# Regio- and stereoselective *tert*-butylthiolation of internal alkynes with thioethers initiated and maintained by silylium-ion catalysis

**DOI:** 10.1039/d5sc09722c

**Published:** 2026-01-02

**Authors:** Dáiríne M. Morgan, Hendrik F. T. Klare, Martin Oestreich

**Affiliations:** a Institut für Chemie, Technische Universität Berlin Strasse des 17. Juni 115 10623 Berlin Germany martin.oestreich@tu-berlin.de

## Abstract

A two-component protocol for the regio- and *trans*-selective addition of aryl tertiary alkyl (especially *tert*-butyl) thioethers across internal C≡C triple bonds is reported. This carbothiolation is initiated by the catalytic formation of a silylated sulfonium ion as a tertiary carbenium ion (*tert*-butyl cation) source. Competing loss of a proton by β-elimination of that carbenium-ion intermediate and as such a potential hydrothiolation pathway are efficiently suppressed by the substoichiometric addition of an arylsilane as a “proton-into-silylium ion” generator. Through this, the silylium-ion activation is restored, thereby maintaining the carbothiolation pathway. The method enables the synthesis of sterically crowded, fully substituted aryl vinyl sulfides as well as the sulfoxides and sulfones.

## Abstract 2

A silylium-ion-promoted carbothiolation of internal C≡C triple bonds is presented. Competing hydrothiolation is mitigated by the addition of substoichiometric amounts of an arylsilane as a “proton-into-silylium ion” generator.

## Graphical abstract

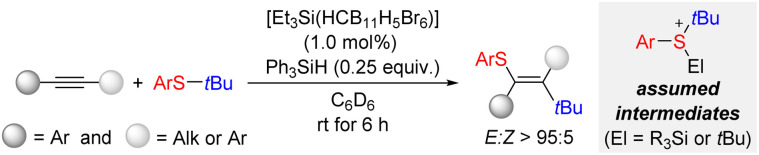

## Introduction

Alkenes are widely used in the synthesis of complex organic molecules and are themselves commonly found in natural products and bioactive compounds.^1^ Despite their ubiquity, the formation of alkenes is non-trivial with the necessity of considering both stereo- and (depending on the method) regioselectivity factors. This is particularly true for the delicate challenging synthesis of highly substituted alkenes, this being C=C double bonds with three or even four carbon substitutents.^2,3^ One possible approach to the synthesis of sterically encumbered alkenes is the selective difunctionalization of alkynes.^4,5^ A wide range of functional groups and carbon substituents have been added across C≡C triple bonds by either three- or two-component reaction systems.^6–12^ Three-component reactions involve the use of a separate nucleophile and electrophile component for sequential addition to the alkyne unit.^13,14^ From an atom-economy perspective the more attractive option however is a two-component reaction in which both units for the addition to the alkyne come from a single reactant.^4,15^ A carbon substituent that has proven particularly challenging to install is a quaternary carbon atom emerging from a tertiary reactant. Although a number of examples have been published where addition of a tertiary carbon center to a terminal alkyne is achieved,^16–22^ examples involving the addition to an internal alkyne are more limited and usually involve activation of the reactants.^23–27^

**Scheme 1:**
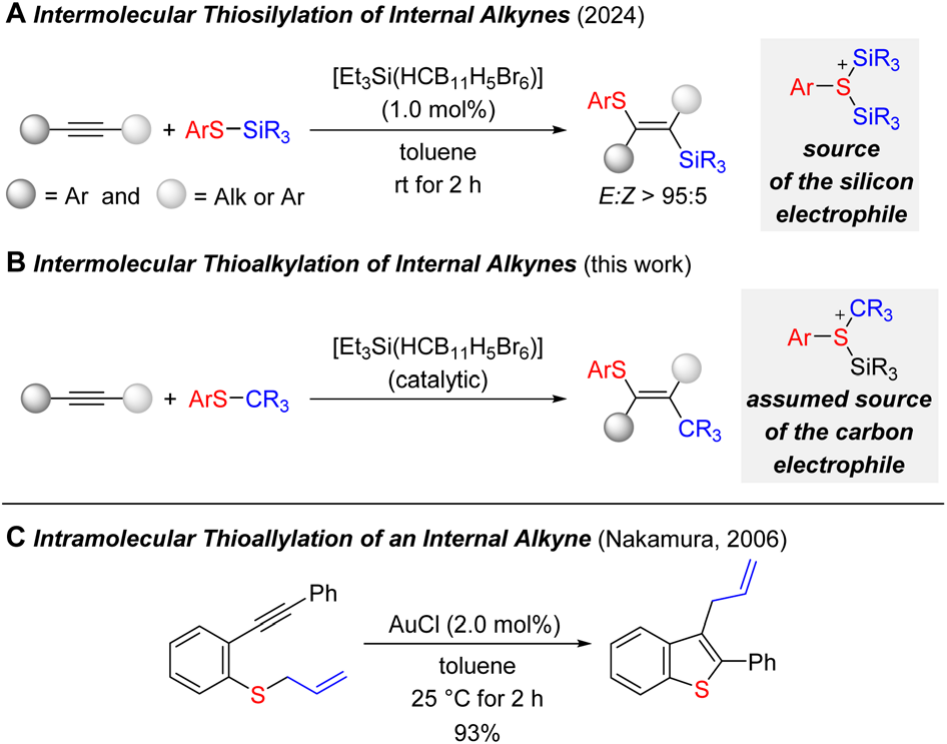
Silylium-ion-promoted thiosilylation and thioalkylation (carbothiolation) of internal alkynes.

Our group recently showed that S–Si bonds can be selectively added across an internal triple bond in a silylium-ion^28^ promoted reaction (Scheme 1A).^15^ This two-component difunctionalization provided a reliable access to fully substituted double bonds with two synthetic handles for further functional-group manipulation. Given the intermediacy of various cationic species, we asked ourselves whether that strategy would allow for the release and transfer of tertiary carbenium ions from thioethers (Scheme 1B). This would correspond to an intermolecular carbothiolation of alkynes. We are only aware of a single related example where Nakamura and co-workers disclosed an intramolecular gold-catalyzed carbothiolation by starting from an allylated thiophenyl derivative (Scheme 1C).^29^ Since then, a number of metal-catalyzed carbothiolation reactions of alkynes have been developed^29–36^ but, to the best of our knowledge, none of these involved simple tertiary alkyl groups.

## Results and discussion

We began our optimization using *tert*-butyl(phenyl)sulfane (1a) and but-1-*yn*-1-ylbenzene (2a) as model compounds (Table 1 and for full details see Tables S1–S5 in the SI). Beginning with the reaction conditions from the aforementioned thiosilylation,^15^ the desired carbothiolation product 3aa was obtained as a single stereoisomer in toluene with 1.0 mol% of [Me_3_Si(HCB_11_H_5_Br_6_)] in a 28% yield along with 18% yield of the hydrothiolation byproduct 4aa (entry 1). A survey of solvents revealed that (deuterated) benzene is an optimal choice, providing 3aa in a 43% yield with 17% of 4aa (entry 2). The reaction time could be reduced from 16 to 6 h with the yield maintained (entry 3). The equivalents of the starting material used played a significant role: Increasing the amount of the alkyne to two equivalents improved the yield of 3aa to 63% with 13% of 4aa (entry 4). This was likely due to the increased concentration of the alkyne accelerating the rate of the bimolecular process. If the volume of solvent used was increased, the overall yield reduced further pointing towards the importance of substrate concentration in this reaction (see Table S3 in the SI). A number of different initiators were then tested and, for example, the trityl salt [Ph_3_C][HCB_11_H_5_Br_6_] failed to initiate the reaction (entry 5) but the silylium salt [Et_3_Si(HCB_11_H_5_Br_6_)] improved the yield to 75% 3aa with 14% of 4aa (entry 6). We presumed that the hydrothiolation product 4aa was being generated by a proton released from the tertiary carbenium ion by β-elimination. We therefore added an arylsilane in order to trap this proton by proton-into-silylium ion interconversion.^37–41^ Ph_4_Si as an additive was almost completely insoluble, hence not bringing about any improvement (entry 7). Using 0.50 equiv. of Ph_3_SiH, it was possible to reduce the amount of 4aa to 5% while maintaining a 73% yield of the desired product 3aa (entry 8). Use of 1.0 equiv. of the alkyne led to a reduction in the yield of 3aa to 51% (entry 9).

**Table 1:** Optimization of the *trans*-selective alkyne carbothiolation^a^^,^^b^

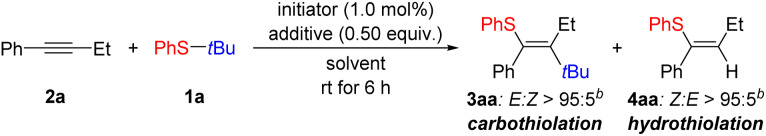							
Entry	Initiator	Solvent	2a (equiv.)	Additive	Yield of 3aa (%)^c^	Yield of 4aa (%)^c^	Ratio of 3aa : 4aa^d^
1^e^	[Me_3_Si(HCB_11_H_5_Br_6_)]	Toluene	1.0		28	18	61 : 39
2^e^	[Me_3_Si(HCB_11_H_5_Br_6_)]	C_6_D_6_	1.0		43	17	72 : 28
3	[Me_3_Si(HCB_11_H_5_Br_6_)]	C_6_D_6_	1.0		46	18	72 : 28
4	[Me_3_Si(HCB_11_H_5_Br_6_)]	C_6_D_6_	2.0		63	13	83 : 17
5	[Ph_3_C][HCB_11_H_5_Br_6_]	C_6_D_6_	2.0		n.r.	n.r.	
6	[Et_3_Si(HCB_11_H_5_Br_6_)]	C_6_D_6_	2.0		75	14	84 : 16
7	[Et_3_Si(HCB_11_H_5_Br_6_)]	C_6_D_6_	2.0	Ph_4_Si	36	9	80 : 20
8	[Et_3_Si(HCB_11_H_5_Br_6_)]	C_6_D_6_	2.0	Ph_3_SiH	73	5	94 : 6
9	[Et_3_Si(HCB_11_H_5_Br_6_)]	C_6_D_6_	1.0	Ph_3_SiH	51	7	88 : 12

a All reactions were performed on a 0.20 mmol scale in a glovebox under an argon atmosphere in 0.5 mL of the indicated solvent.

b *E* : *Z* ratios estimated by ^1^H NMR spectroscopy of the crude reaction mixture.

c Yield determined by ^1^H NMR spectroscopy of the crude reaction mixture using CH_2_Br_2_ as an internal standard.

d 3aa : 4aa ratios determined by ^1^H NMR spectroscopy of the crude reaction mixture.

e Reaction time 16 h. n.r. = no reaction.

**Scheme 2:**
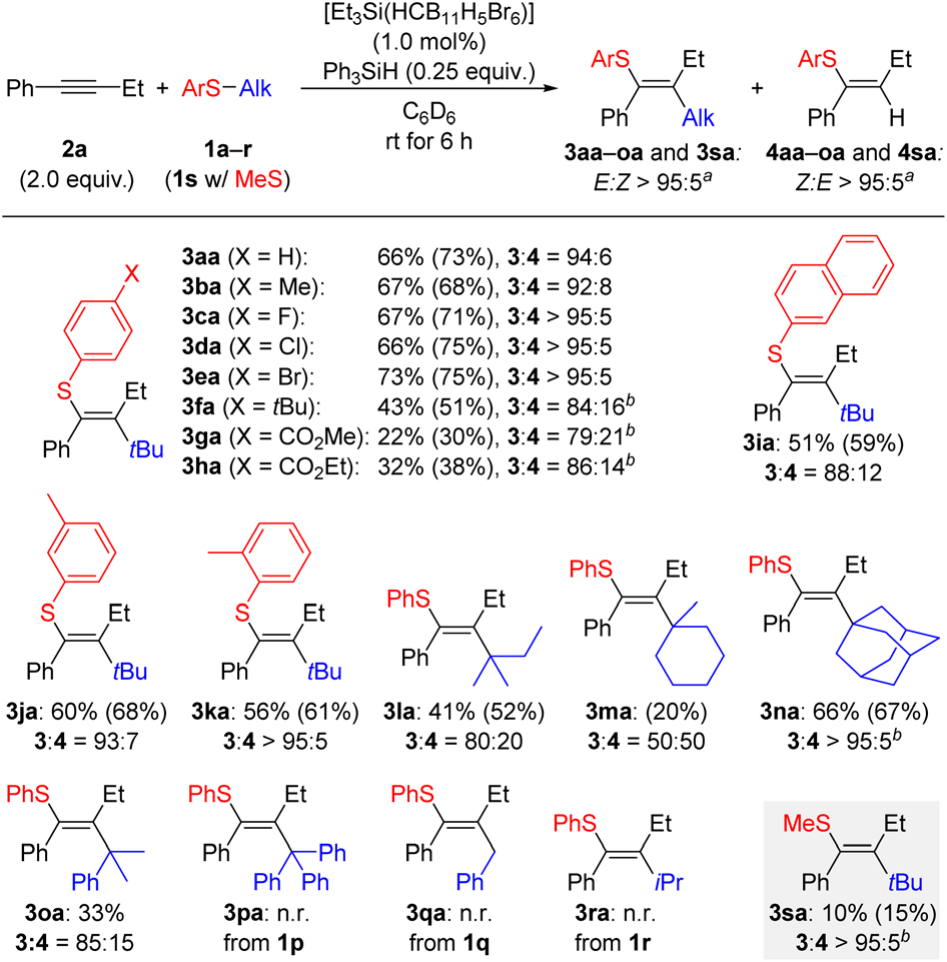
Substrate scope I: variation of the thioether. All reactions were performed using the indicated thioether 1 (0.20 mmol), the alkyne 2a (0.40 mmol, 2.0 equiv.), Ph_3_SiH (0.050 mmol, 0.25 equiv.), and the initiator [Et_3_Si(HCB_11_H_5_Br_6_)] (2.0 µmol, 1.0 mol%) in a glovebox under an argon atmosphere in C_6_D_6_ (0.5 mL) at rt. Isolated yields refer to analytically pure material (combined 3 and 4) after flash chromatography on silica gel; yields in parentheses determined by ^1^H NMR spectroscopy of the crude reaction mixture using CH_2_Br_2_ as an internal standard. ^*a*^*E* : *Z* ratios estimated by ^1^H NMR spectroscopy of the crude reaction mixture. ^*b*^ Reaction performed at 60 °C.

With the optimized conditions in hand, we turned to the substrate scope of the reaction. In many cases, separation of the product from unreacted Ph_3_SiH proved difficult, so the amount used was reduced from 0.50 to 0.25 equiv. We began gauging the substrate scope by varying the aryl ring on the thioether reagent 1 (Scheme 2). Methyl and halogen groups were well tolerated at the *para* position of the aryl group with only a small impact on the yield observed for 3ba–ea. When a bulkier group such as *tert*-butyl as in 1f was present, the activity of the reaction reduced significantly but a yield of 43% for 3fa could be obtained when the reaction temperature was increased to 60 °C. Although the yields remained low even at increased temperatures, products 3ga and 3ha bearing an ester group could also be obtained. The configuration of the double bond was assigned for 3ha by an nOe measurement (see Fig. S2 in the SI). A more sterically bulky β-naphthyl group in 1i resulted in a moderate yield for 3ia. Likewise, 1j and 1k with *meta*- and *ortho*-tolyl groups led to yields for 3ja and 3ka in the same range. Variation of the tertiary alkyl group was next looked at. When the *tert*-butyl group was replaced by a *tert*-amyl residue, a yield of 41% was obtained for 3la along with a substantial amount of the hydrothiolation product 4la. The hydrothiolation pathway became even more pronounced when a cyclic tertiary carbenium ion was present, resulting in a 1 : 1 ratio of 3ma and 4ma. An adamantyl worked equally well as a *tert*-butyl group, leading to the formation of 3na in 66% yield with hardly any hydrothiolation byproduct. Any benzylic carbocation was either transferred sluggishly (1o → 3oa) or did not react as planned (trityl as in 1p and benzyl as in 1q). A secondary alkyl group as potentially released from isopropyl-substituted thioether 1r did not engage in the reaction. A dialkyl thioether such as 1s furnished the carbothiolation product 3sa only in poor yield (gray box).

**Scheme 3:**
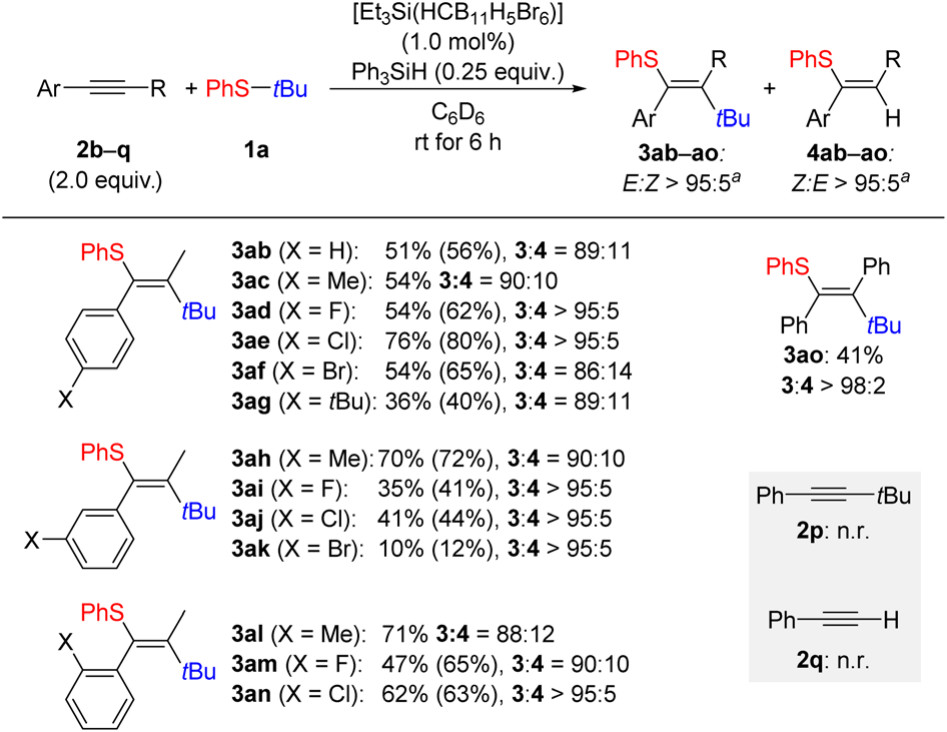
Substrate scope II: variation of the internal alkyne. All reactions were performed using the thioether 1a (0.20 mmol), the indicated alkyne 2 (0.40 mmol, 2.0 equiv.), Ph_3_SiH (0.050 mmol, 0.25 equiv.), and the initiator [Et_3_Si(HCB_11_H_5_Br_6_)] (2.0 µmol, 1.0 mol%) in a glovebox under an argon atmosphere in C_6_D_6_ (0.5 mL) at rt. Isolated yields refer to analytically pure material (combined 3 and 4) after flash chromatography on silica gel; yields in parentheses determined by ^1^H NMR spectroscopy of the crude reaction mixture using CH_2_Br_2_ as an internal standard. ^*a*^*E* : *Z* ratios estimated by ^1^H NMR spectroscopy of the crude reaction mixture.

Following this, the aryl-substituted alkyne used was varied systematically (Scheme 3). Aliphatic alkynes were not compatible with this reaction with the aryl ring necessary for reaction initiation. Substitution at the *para* position of the aryl substituent in alkyne 2 was tolerated with moderate to good yields for 3ab–af. The exception to this was again a *tert*-butyl group which led to a reduction in yield to 36% for 3ag. Substitution at the *meta* position proved to be more challenging although a good yield was obtained for the tolyl-substituted derivative 3ah. The three halogenated products 3ai–ak were all obtained in low yields. In turn, substitution at the *ortho* position had little effect on the overall yield of the product 3al–an. It was possible to utilize diphenylacetylene (2o) in the reaction, and a moderate yield of 41% was found for the carbothiolation product 3ao. Conversely, no reaction occurred with an alkyne bearing a *tert*-butyl group as in 2p or with a terminal alkyne as for phenylacetylene (2q), respectively (gray box).

**Scheme 4:**
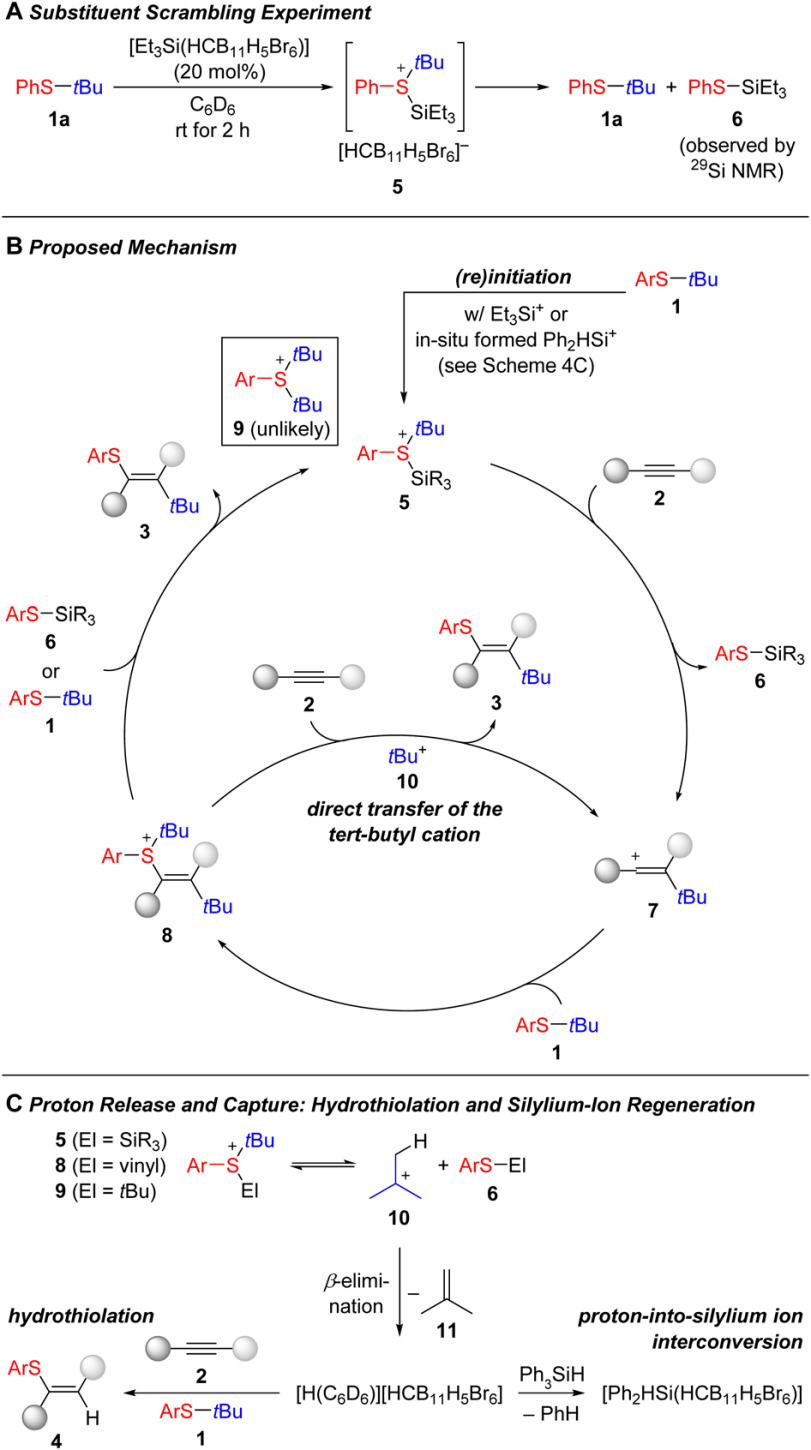
Discussion of the mechanism. The [HCB_11_H_5_Br_6_]^−^ counterion is omitted for clarity in the catalytic cycle.

We believe that the mechanism of this carbothiolation reaction exhibits similarities to that of the silylium-ion-promoted thiosilylation of alkynes (see Scheme 1A).^15^ Interaction of the initiator [Et_3_Si(HCB_11_H_5_Br_6_)] with the aryl *tert*-butyl thioether was verified by a substituent exchange experiment (Scheme 4A). When thioether 1a was reacted with 20 mol% of the counteranion-stabilized silylium ion, the formation of the thiosilane 6 was detected by ^29^Si NMR spectroscopy, thereby suggesting the intermediacy of the silylsulfonium ion 5. Intermediate 5 is the starting point of the catalytic cycle (Scheme 4B). It transfers the *tert*-butyl cation onto the alkyne 2 to form the vinyl cation 7,^42–48^ which in turn reacts with excess thioether 1 to yield another sulfonium-ion intermediate 8. Although it is obvious that this sulfonium ion will release the *tert*-butyl cation to eventually liberate the carbothiolation product 3, its actual fate remains unclear. The role of sulfides 1 and 6 as carbenium-ion shuttles is a possibility but *tert*-butyl-substituted 1 to form bis-*tert*-butylated arylsulfonium ion 9 is unlikely for steric considerations and thiosilane 6 is only available at low concentration. Hence, we assume that the *tert*-butyl group is directly transferred from intermediate 8 to alkyne 2 thereby closing the catalytic cycle. The observation of the hydrothiolation product 4 lends further evidence for this pathway (Scheme 4C). Dissociation of any of the sulfonium ions 5, 8, or 9 gives access to a free *tert*-butyl cation 10, that suffers β-elimination by loss of a proton to give isobutene 11. That proton can be accepted by the alkyne 2 (or thioether 1 and silylated thiophenol 6) opening the door to the hydrothiolation channel. To suppress this side reaction, we decided to exploit the ability of [H(C_6_D_6_)][HCB_11_H_5_Br_6_] to convert arylsilanes into counteranion-stabilzed silylium ions by dearylation.^37–41^ That proton-into-silylium ion interconversion not only sequesters the strong Brønsted acid but also makes available [Ph_2_HSi(HCB_11_H_5_Br_6_)] (from Ph_3_SiH) for further sulfonium-formation. The arylsilane additive thereby enhances the overall performance of the catalysis.^37,49^ We also examined whether the carbothiolation is reversible but a scrambling experiment between product 3aa (Ar = Ph) and thioether 1b (Ar = 4-Tolyl) under the standard reaction conditions showed no exchange; the vinyl sulfide 3aa was recovered exclusively and a small amount of degradation was seen for 1b.

**Scheme 5:**
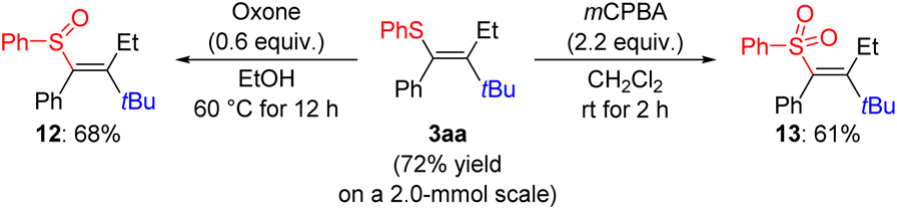
Oxidation of the fully substituted vinyl sulfide. Yields are isolated and refer to analytically pure material after flash chromatography on silica gel.

To explore the utility of this reaction, we performed the model reaction on a 2.0-mmol scale, furnishing the vinyl sulfide 3aa in a slightly improved isolated yield of 72% (Scheme 5). In an effort to prime the carbon–sulfur bond in 3aa for further functional-group manipulation, we oxidized sulfur atom to the sulfoxide 12 with oxone in ethanol,^50^ and to the sulfone 13 with *m*CPBA.^15^ At this stage, attempts to engage any of these three vinyl components in a transition-metal-catalyzed cross-coupling have been unsuccessful in our hands.^51–53^

## Conclusions

We have here disclosed a new method for the regio- and *trans*-selective carbothiolation (thioalkylation) of internal alkynes that specifically enables the installation of a *tert*-butyl group at an alkene. A *tert*-butyl-substituted arylsulfide serves as the *tert*-butyl electrophile and at the same time as the sulfur nucleophile. This is made possible by initiation of the reaction with a counteranion-stabilized silylium ion to form a sulfonium ion as the actual carbenium-ion carrier. The reaction displays a good substrate scope with functional-group tolerance similar to that typically seen in other catalyses under superacidic conditions.

## Author contributions

D. M. M., H. F. T. K. and M. O. conceptualized this work. D. M. M. performed and analyzed the experiments. H. F. T. K. and M. O. supervised the research and acquired funding. All authors contributed to the writing and editing of the manuscript.

## Conflicts of interest

There are no conflicts to declare.

## Supplementary Material

SC-OLF-D5SC09722C-s001

## Data Availability

The data supporting this article has been included as part of the supplementary information (SI). Supplementary information: reaction optimizations, experimental procedures, full characterization data and copies of NMR spectra. See DOI: https://doi.org/10.1039/d5sc09722c.
