# Supplementary material for: Regio- and stereoselective tert-butylthiolation of internal alkynes with thioethers initiated and maintained by silylium-ion catalysis
Source: Chem Sci. 2026 Jan 2;17(8):4307–11. doi: 10.1039/d5sc09722c (PMC12794294; doi:10.1039/d5sc09722c)
Supplement: SC-017-D5SC09722C-s001 [file SC-017-D5SC09722C-s001.pdf]

Supplementary Information (SI) for Chemical Science.
This journal is © The Royal Society of Chemistry 2026

Supplementary Information for

Regio- and stereoselective *tert*-butylthiolation of internal alkynes with thioethers initiated and maintained by silylium-ion catalysis

Dáiríne M. Morgan, Hendrik F. T. Klare and Martin Oestreich*

Institut für Chemie, Technische Universität Berlin, Strasse des 17. Juni 115, 10623 Berlin, Germany

Supplementary Information

Table of Contents

1. General Information S3

2. Optimization of the Reaction Conditions S4

3. Experimental Details for Substrate Synthesis S7

3.1 Synthesis of Arylsulfanes from *tert*-Butyl Alcohol (GP 1) S7

3.2 Synthesis of Arylsulfanes from *tert*-Butyl Thiol (GP 2) S7

3.3 Characterisation Data for Arylsulfanes S8

4. Experimental Details for Thioalkylation of Alkynes S16

4.1 General procedure for the thioalkylation of alkynes (GP 3) S16

4.2 Characterisation data for products **3** S16

5. Substituent Scrambling Experiment S31

6. Synthetic Transformations of 3 .... S32

7. Assignment of Alkene Configuration S34

8. NMR Spectra S35

9. References S84

1. General Information

All reactions were performed in flame-dried glassware using an MBraun glovebox (O2 < 0.5 ppm, H2O < 1.0 ppm) or conventional Schlenk techniques under a static pressure of argon (glovebox) or nitrogen (fume hood) unless otherwise stated. Standard solvents and reagents were obtained from commercial suppliers and used as received unless otherwise stated. Glassware was dried overnight at 150 °C or flame dried using a heat gun. All plastic syringes and needles used in the glovebox were dried overnight at 60 °C. Liquids and solutions were transferred with syringes. Technical grade solvents for extraction and chromatography were distilled prior to use. Tetrahydrofuran (THF) was dried over sodium and freshly distilled prior to use. Dry benzene (C_6H_6), and n-pentane were obtained from an MBraun solvent purification system (SPS-800), degassed by three freeze-pump-thaw cycles, and stored in a glovebox over thermally activated 4 Å molecular sieves. Dichloromethane (CH_2Cl_2), toluene (C_7H_8), fluorobenzene (C_6H_5F), chlorobenzene (C_6H_5Cl) and 1,2-dichlorobenzene (1,2-$C_6H_4Cl_2$) were dried over CaH2, distilled, degassed by three freeze-pump-thaw cycles, and stored in a glovebox over thermally activated 4 Å molecular sieves. Silylium carborates [$Me_3Si(HCB_{11}H_5Br_6)$],[S1] [$Et_3Si(HCB_{11}H_5Br_6)$],[S2] and [*i*-$Pr_3Si(HCB_{11}H_5Br_6)$] were synthesized according to reported procedures. Thin-layer chromatography was performed on Macherey-Nagel Alugram® Xtra SIL G/UV254 silica gel 60 pre-coated aluminum-backed plates (200 µm layer thickness). Product spots were visualized under UV light (λmax = 254 nm) and with a ceric ammonium molybdate stain. Column chromatography was performed on Grace 60 (40–63 µm, 230– 400 mesh, ASTM) silica gel. 1H, 13C, 19F and 29Si NMR spectra were recorded in CDCl3 on a Bruker AV400 or AV500 instruments. Chemical shifts are reported in parts per million (ppm) and are referenced to the residual solvent resonance as the internal standard ($CHCl_3$: δ = 7.26 ppm for ^1H NMR and $CDCl_3$: δ = 77.16 ppm for ^{13}C NMR). ^{19}F NMR spectra are referenced in compliance with the unified scale for NMR chemical shifts as recommended by the IUPAC stating the chemical shift relative to CCl_3F.[S3] Data are reported as follows: chemical shift, multiplicity (s = singlet, d = doublet, t = triplet, q = quartet, sept = septet, m = multiplet, br = broad), coupling constants (Hz), and integration. Infrared (IR) spectra were recorded on an Agilent Technologies Cary 630, and the signals are reported in wavenumbers (cm^{-1}). Data for the single crystal structure determination were collected with an Agilent SuperNova diffractometer equipped with a CCD area Atlas detector and a mirror monochromator by utilizing Cu-K_α radiation (λ = 1.5418 Å). Melting points (m.p.) were determined with a Stuart Scientific SMP20 melting point apparatus and were not corrected. High resolution mass spectra (HRMS) were obtained from the Laboratory of Mass Spectrometry at the Institut für Chemie, Technische Universität Berlin on a Thermo Fisher Scientific LTQ Orbitrap XL apparatus using APCI, ESI or LIFDI techniques with a linear ion trap analyzer.

2. Optimization of the Reaction Conditions[a]

Table S1: Optimization of the reaction solvent.

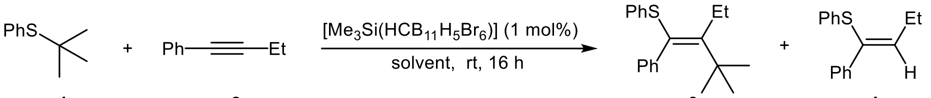

1a (1.2 equiv) **2a** **3aa** **4aa**

Entry	Solvent	Yield **3aa** (%)[b]	Yield **4aa** (%)[b]	Ratio **3:4**
1.	PhMe	28	18	61:39
2.	PhH	38	16	70:30
3.	o-DFB	22	14	61:39
4.	PhCl	32	16	67:33
5.	o-DCB	19	16	54:46
6.	PhF	41	19	68:32
7.	Hexane	5	4	56:44
8.	CH_2Cl_2	28	13	68:32
9.	C_6D_6	43	17	72:28

[a]All reactions were performed on a 0.20 mmol scale under argon atmosphere in 0.5 mL of indicated solvent. *E:Z* > 95:5 in all cases, as verified by ^{1}H NMR spectroscopy of the crude reaction mixture. [b]Yields were determined by ^{1}H NMR spectroscopy using CH_2Br_2 as an internal standard.

Table S2: Optimization of the reaction time.

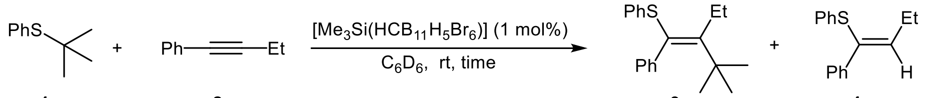

1a (1.2 equiv) **2a** **3aa** **4aa**

Entry	Time	Yield **3aa** (%)[b]	Yield **4aa** (%)[b]	Ratio **3:4**
2.	1 h	18	7	72:28
3.	2 h	24	9	73:27
4.	4 h	43	16	73:27
5.	6 h	46	18	72:28

[a]All reactions were performed on a 0.20 mmol scale under argon atmosphere in 0.5 mL of indicated solvent. *E:Z* > 95:5 in all cases, as verified by ^{1}H NMR spectroscopy of the crude reaction mixture. [b]Yields were determined by ^{1}H NMR spectroscopy using CH_2Br_2 as an internal standard.

Table S3: Optimization of the reactant equivalents.

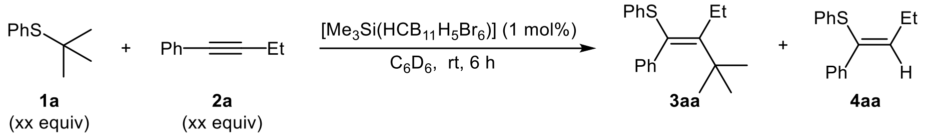

Entry	Equiv **1a**	Equiv **2a**	Yield **3aa** (%)[b]	Yield **4aa** (%)[b]	Ratio **3:4**
1.	1.2	1.0	46	18	72:28
2.	1.5	1.0	43	20	68:32
3.	2.0	1.0	42	24	64:36
4.	2.5	1.0	42	24	64:36
5.	1.0	1.5	57	14	80:20
6.	1.0	2.0	63	13	83:17
7.	1.0	2.5	60	10	86:14
8.[c]	1.0	2.0	60	10	86:14
9.[d]	1.0	2.0	49	10	83:17
10.[e]	1.0	2.0	44	10	81:19

[a]All reactions were performed on a 0.20 mmol scale under argon atmosphere in 0.5 mL of indicated solvent. *E:Z* > 95:5 in all cases, as verified by ^{1}H NMR spectroscopy of the crude reaction mixture. [b]Yields were determined by ^{1}H NMR spectroscopy using CH_2Br_2 as an internal standard. [c] 0.25 mL C_6D_6 used. [d] 1.0 mL C_6D_6 used. [e] 1.5 mL C_6D_6 used.

Table S4: Optimization of the initiator used.

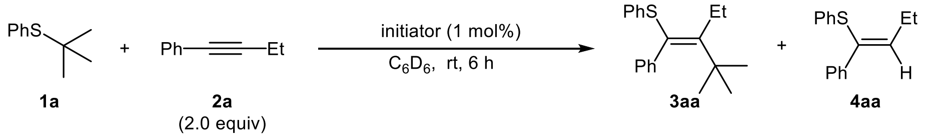

Entry	Initiator	Yield **3aa** (%)[b]	Yield **4aa** (%)[b]	Ratio **3:4**
1.	$[Me_3Si(HCB_{11}H_5Br_6)]$	63	13	83:17
2.	$[Et_3Si(HCB_{11}H_5Br_6)]$	75	14	84:16
3.	[*i*-$Pr_3Si(HCB_{11}H_5Br_6)$]	67	15	82:18
4.	$[Ph_3C][HCB_{11}H_5Br_6]$	N.R.	-	-
5.	$AlCl_3$ (5 mol%)	N.R.	-	-
6.	$InCl_3$ (5 mol%)	N.R.	-	-
7.	TfOH (5 mol%)	N.R.	-	-

[a]All reactions were performed on a 0.20 mmol scale under argon atmosphere in 0.5 mL of indicated solvent. *E:Z* > 95:5 in all cases, as verified by ^{1}H NMR spectroscopy of the crude reaction mixture. [b]Yields were determined by ^{1}H NMR spectroscopy using CH_2Br_2 as an internal standard.

Table S5: Optimization of additive used.

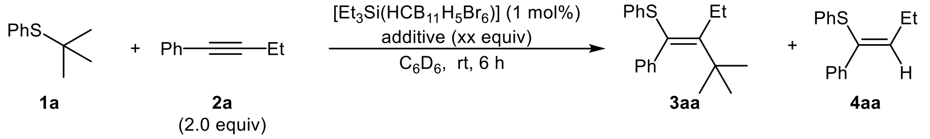

Entry	Additive	Yield **3aa** (%)[b]	Yield **4aa** (%)[b]	Ratio **3:4**
1.	-	75	14	84:16
2.	Et_3SiH (1.0 equiv)	67	16	81:19
3.	Et_3SiPh (1.0 equiv)	74	15	83:17
4.	Ph_3SiH (1.0 equiv)	69	4	95:5
5.	Ph_3Si*t*-Bu (1.0 equiv)	45	8	85:15
6.	$PhMe_2SiH$ (1.0 equiv)	44	<2	>95:5
7.	*i*Pr_3SiH (1.0 equiv)	44	3	94:6
8.	*t*Bu_2MeSiH (1.0 equiv)	51	6	89:11
9.	allyl$SiEt_3$ (1.0 equiv)	28	<2	>95:5
10.	Ph_3SiH (1.5 equiv)	62	4	94:6
11.	Ph_3SiH (0.5 equiv)	73	5	94:6
12.	Ph_3SiH (0.2 equiv)	76	8	90:10

[a]All reactions were performed on a 0.20 mmol scale under argon atmosphere in 0.5 mL of indicated solvent. *E:Z* > 95:5 in all cases, as verified by ^1H NMR spectroscopy of the crude reaction mixture. [b]Yields were determined by ^1H NMR spectroscopy using CH_2Br_2 as an internal standard.

3. Experimental Details for Substrate Synthesis

3.1 Synthesis of Arylsulfanes from *tert*-Butyl Alcohol (GP 1)

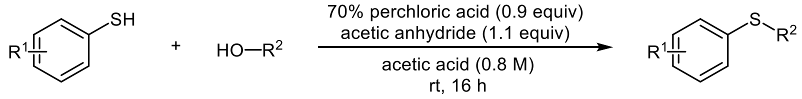

According to a literature procedure,[S4] a 25 mL round-bottom flask with a stir bar is charged with thiophenol (5.0 mmol), alcohol (6.0 mmol, 1.2 equiv) and acetic acid (5.0 mL, 0.80 M). The reaction is then cooled to 0 °C and acetic anhydride (0.52 mL, 5.5 mmol, 1.1 equiv) and perchloric acid (0.27 mL, 4.5 mmol, 0.90 equiv) are added sequentially. Following this the reaction is allowed warm to room temperature and left stirring for 16 h. The solution is then diluted with water (10 mL) and extracted with Et_2O (3 x 25 mL). The combined organic phases are then neutralised and washed with saturated aqueous sodium bicarbonate solution (3 x 25 mL). The organic layer is then dried over $MgSO_4$, filtered, and the solvent is removed *in vacuo*. Purification of the residue by flash column chromatography on silica gel using *n*-pentane as the eluent affords the arylsulfane in analytically pure form.

3.2 Synthesis of Arylsulfanes from *tert*-Butyl Thiol (GP 2)

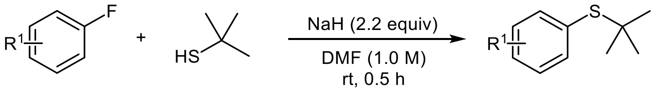

According to a literature procedure,[S5] an oven dried 25 mL Schlenk tube is evacuated and backfilled with nitrogen three times. DMF (5.0 mL) is added. Sodium hydride (60% in mineral oil) (11 mmol, 2.2 equiv) is then added portion wise and the reaction is stirred for 20 min. *tert*-Butyl thiol (5.5 mmol, 1.1 equiv) is added dropwise. The reaction is cooled to 0 °C and fluorobenzene (5.0 mmol) is carefully added dropwise. The reaction is allowed warm to room temperature and stirred for 30 min. Water (15 mL) is slowly added and the reaction is stirred for 15 min. The solution is extracted with ethyl acetate (3 x 20 mL). The combined organic phases are dried over $MgSO_4$, filtered and the solvent is removed *in vacuo*. Purification of the residue by flash column chromatography on silica gel (*n*-pentane/EtOAc 25:1) affords the arylsulfane in analytically pure form.

3.3 Characterisation Data for Arylsulfanes

tert-Butyl(phenyl)sulfane (1a)

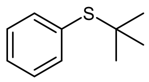

1a
$C_{10}H_{14}S$
M = 166.28 g/mol

Prepared from benzenethiol (550.9 mg, 5.0 mmol) and *tert*-butanol (444.7 mg, 6.0 mmol) according to **GP 1**. Flash column chromatography on silica gel using *n*-pentane afforded substrate **1a** as a colourless oil (565.2 mg, 3.4 mmol, 68% yield).

^{1}H NMR (500 MHz, $CDCl_3$, 298 K) δ = 7.63 – 7.49 (m, 2H), 7.43 – 7.30 (m, 3H), 1.32 (s, 9H).

^{13}C{^{1}H} NMR (126 MHz, $CDCl_3$, 298 K) δ = 137.6, 132.9, 128.8, 128.6, 46.0, 31.1.

The NMR spectroscopic data are in accordance with those reported.[S4]

tert-Butyl(*p*-tolyl)sulfane (1b)

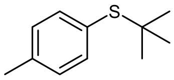

1b
$C_{11}H_{16}S$
M = 180.31 g/mol

Prepared from 4-methylbenzenethiol (621.0 mg, 5.0 mmol) and *tert*-butanol (444.7 mg, 6.0 mmol) according to **GP 1**. Flash column chromatography on silica gel using *n*-pentane afforded substrate **1b** as a colourless oil (663.1 mg, 3.7 mmol, 74% yield).

^{1}H NMR (500 MHz, $CDCl_3$, 298 K) δ = 7.41 (d, J = 7.9 Hz, 2H), 7.14 (d, J = 7.7 Hz, 2H), 2.36 (s, 3H), 1.27 (s, 9H).

^{13}C{^{1}H} NMR (126 MHz, $CDCl_3$, 298 K) δ = 138.9, 137.6, 129.4, 129.4, 45.7, 31.0, 21.4.

The NMR spectroscopic data are in accordance with those reported.[S6]

tert-Butyl(4-fluorophenyl)sulfane (1c)

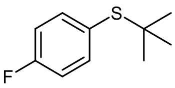

1c
$C_{10}H_{13}FS$
M = 184.27 g/mol

Prepared from 4-fluorobenzenethiol (640.8 mg, 5.0 mmol) and *tert*-butanol (444.7 mg, 6.0 mmol) according to **GP 1**. Flash column chromatography on silica gel using *n*-pentane afforded substrate **1c** as a colourless oil (721.0 mg, 3.9 mmol, 78% yield).

^{1}H NMR (500 MHz, $CDCl_3$, 298 K) δ = 7.53 – 7.46 (m, 2H), 7.02 (t, *J* = 8.7 Hz, 2H), 1.27 (d, *J* = 2.0 Hz, 9H).

^{13}C{^{1}H} NMR (126 MHz, $CDCl_3$, 298 K) δ = 163.6 (d, *J* = 249.1 Hz), 139.4 (d, *J* = 8.3 Hz), 128.3 (d, *J* = 3.4 Hz), 115.7 (d, *J* = 21.6 Hz), 45.9, 31.0.

The NMR spectroscopic data are in accordance with those reported.[S6]

tert-Butyl(4-chlorophenyl)sulfane (1d)

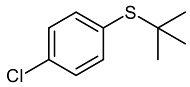

1d
$C_{10}H_{13}ClS$
M = 200.72 g/mol

Prepared from 4-chlorobenzenethiol (723.1 mg, 5.0 mmol) and *tert*-butanol (444.7 mg, 6.0 mmol) according to **GP 1**. Flash column chromatography on silica gel using *n*-pentane afforded substrate **1d** as a colourless oil (589.0 mg, 2.9 mmol, 59% yield).

^{1}H NMR (500 MHz, $CDCl_3$, 298 K) δ = 7.45 (d, *J* = 8.0 Hz, 2H), 7.30 (d, *J* = 8.0 Hz, 2H), 1.28 (s, 9H).

^{13}C{^{1}H} NMR (126 MHz, $CDCl_3$, 298 K) δ = 138.8, 135.3, 131.5, 128.8, 46.3, 31.0.

The NMR spectroscopic data are in accordance with those reported.[S6]

tert-Butyl(4-bromophenyl)sulfane (1e)

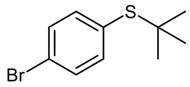

1e
$C_{10}H_{13}BrS$
M = 245.18 g/mol

Prepared from 4-bromobenzenethiol (945.4 mg, 5.0 mmol) and *tert*-butanol (444.7 mg, 6.0 mmol) according to **GP 1**. Flash column chromatography on silica gel using *n*-pentane afforded substrate **1e** as white solid (1076 mg, 4.4 mmol, 88% yield).

^{1}H NMR (500 MHz, $CDCl_3$, 298 K) δ = 7.45 (d, J = 8.1 Hz, 2H), 7.38 (d, J = 8.1 Hz, 2H), 1.28 (s, 9H).

^{13}C{^{1}H} NMR (126 MHz, $CDCl_3$, 298 K) δ = 139.0, 132.1, 131.8, 123.6, 46.2, 31.0.

The NMR spectroscopic data are in accordance with those reported.[S7]

tert-Butyl(4-(_tert_-butyl)phenyl)sulfane (1f)

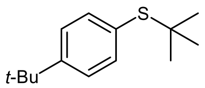

1f
$C_{14}H_{22}S$
M = 222.39 g/mol

Prepared from 4-*tert*-butylbenzenethiol (831.4 mg, 5.0 mmol) and *tert*-butanol (444.7 mg, 6.0 mmol) according to **GP 1**. Flash column chromatography on silica gel using *n*-pentane afforded substrate **1f** as a colourless oil (901.3 mg, 4.1 mmol, 81% yield).

^{1}H NMR (500 MHz, $CDCl_3$, 298 K) δ = 7.45 (d, *J* = 8.2 Hz, 2H), 7.34 (d, *J* = 8.2 Hz, 2H), 1.32 (s, 9H), 1.29 (s, 9H).

^{13}C{^{1}H} NMR (126 MHz, $CDCl_3$, 298 K) δ = 152.0, 137.3, 129.4, 125.6, 45.8, 34.8, 31.4, 31.1.

The NMR spectroscopic data are in accordance with those reported.[S8]

Methyl-4-(*tert*-butylthio)benzoate (1g)

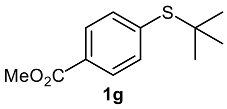

1g

$C_{12}H_{16}O_2S$

M = 224.32 g/mol

Prepared from methyl-4-flurorobenzoate (770.7 mg, 5.0 mmol) and *tert*-butylthiol (496.0 mmol, 5.5 mmol) according to **GP 2.** Flash column chromatography on silica gel (*n*-pentane/EtOAc 25:1) afforded the substrate **1g** as a white solid (274.0 mg, 1.2 mmol, 24% yield).

^{1}H NMR (500 MHz, $CDCl_3$, 298 K) δ = 8.00 – 7.95 (m, 2H), 7.62 – 7.56 (m, 2H), 3.92 (s, 3H), 1.31 (s, 9H).

^{13}C{^{1}H} NMR (126 MHz, $CDCl_3$, 298 K) δ = 166.9, 139.2, 136.9, 130.3, 129.6, 52.3, 46.9, 31.3.

The NMR spectroscopic data are in accordance with those reported.[S9]

Ethyl-4-(*tert*-butylthio)benzoate (1h)

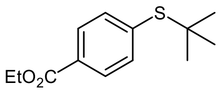

1h

$C_{13}H_{18}O_2S$

M = 238.35 g/mol

Prepared from ethyl-4-flurorobenzoate (840.8 mg, 5.0 mmol) and *tert*-butylthiol (496.0 mmol, 5.5 mmol) according to **GP 2.** Flash column chromatography on silica gel (*n*-pentane/EtOAc 25:1) afforded the substrate **1h** as a white solid (562.0 mg, 2.4 mmol, 47% yield).

^{1}H NMR (500 MHz, $CDCl_3$, 298 K) δ = 8.01 – 7.95 (m, 2H), 7.61 – 7.55 (m, 2H), 4.38 (q, *J* = 7.1 Hz, 2H), 1.39 (t, *J* = 7.1, 3H), 1.31 (s, 10H).

^{13}C{^{1}H} NMR (126 MHz, $CDCl_3$, 298 K) δ = 166.4, 139.0, 136.9, 130.6, 129.5, 61.2, 46.8, 31.2, 14.4.

The NMR spectroscopic data are in accordance with those reported.[S10]

***tert*-Butyl(naphthale*n*-2-yl)sulfane (1i)**

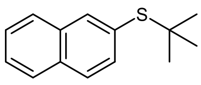

1i
$C_{14}H_{16}S$
M = 216.34 g/mol

Prepared from naphthalene-2-thiol (801.2 mg, 5.0 mmol) and *tert*-butanol (444.7 mg, 6.0 mmol) according to **GP 1**. Flash column chromatography on silica gel using *n*-pentane afforded substrate **1k** as a white solid (883.0 mg, 4.1 mmol, 82% yield).

^{1}H NMR (500 MHz, $CDCl_3$, 298 K) δ = 8.08 (d, *J* = 1.7 Hz, 1H), 7.88 – 7.82 (m, 2H), 7.79 (d, *J* = 8.5 Hz, 1H), 7.63 – 7.58 (m, 1H), 7.55 – 7.48 (m, 2H), 1.35 (s, 9H).

^{13}C{^{1}H} NMR (126 MHz, $CDCl_3$, 298 K) δ = 137.2, 134.4, 133.6, 133.3, 130.4, 128.1, 127.9, 127.8, 126.8, 126.4, 46.5, 31.2.

The NMR spectroscopic data are in accordance with those reported.[S6]

***tert*-Butyl(*m*-tolyl)sulfane (1j)**

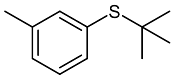

1j
$C_{11}H_{16}S$
M = 180.31 g/mol

Prepared from 3-methylbenzenethiol (621.0 mg, 5.0 mmol) and *tert*-butanol (444.7 mg, 6.0 mmol) according to **GP 1**. Flash column chromatography on silica gel using *n*-pentane afforded substrate **1j** as a colourless oil (735.0 mg, 4.1 mmol, 82% yield).

^{1}H NMR (500 MHz, $CDCl_3$, 298 K) δ = 7.38 – 7.31 (m, 2H), 7.25 – 7.14 (m, 2H), 2.36 (s, 3H), 1.30 (s, 9H).

^{13}C{^{1}H} NMR (126 MHz, $CDCl_3$, 298 K) δ = 138.3, 138.2, 134.6, 132.6, 129.6, 128.4, 45.8, 31.2, 21.4.

The NMR spectroscopic data are in accordance with those reported.[S11]

***tert*-Butyl(*o*-tolyl)sulfane (1k)**

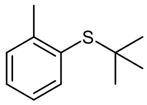

1k
$C_{11}H_{16}S$
M = 180.31 g/mol

Prepared from 2-methylbenzenethiol (621.0 mg, 5.0 mmol) and *tert*-butanol (444.7 mg, 6.0 mmol) according to **GP 1**. Flash column chromatography on silica gel using *n*-pentane afforded substrate **1k** as a colourless oil (747.0 mg, 4.1 mmol, 83% yield).

^{1}**H NMR** (500 MHz, $CDCl_3$, 298 K) δ = 7.56 – 7.51 (m, 1H), 7.31 – 7.22 (m, 2H), 7.18 – 7.11 (m, 1H), 2.53 (s, 3H), 1.31 (s, 9H).

^{13}**C{^{1}H} NMR** (126 MHz, $CDCl_3$, 298 K) δ = 144.0, 139.1, 132.4, 130.5, 129.0, 125.9, 47.4, 31.3, 22.0.

The NMR spectroscopic data are in accordance with those reported.[S12]

tert-Pentyl(phenyl)sulfane (1l)

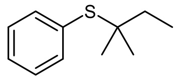

1l
$C_{11}H_{16}S$
M = 180.31 g/mol

Prepared from benzenethiol (550.9 mg, 5.0 mmol) and 2-methylbutan-2-ol (528.9 mg, 6.0 mmol) according to **GP 1**. Flash column chromatography on silica gel using *n*-pentane afforded substrate **1l** as a colourless oil (758.0 mg, 4.2 mmol, 84% yield).

^{1}**H NMR** (500 MHz, $CDCl_3$, 298 K) δ = 7.30 – 7.24 (m, 2H), 7.13 – 7.03 (m, 3H), 1.26 (q, J = 7.3 Hz, 2H), 0.97 (s, 6H), 0.76 (t, J = 7.4 Hz, 3H).

^{13}**C{^{1}H} NMR** (126 MHz, $CDCl_3$, 298 K) δ = 137.7, 132.7, 128.7, 128.5, 49.9, 35.0, 28.4, 9.4.

The NMR spectroscopic data are in accordance with those reported.[S13]

(1-Methylcyclohexyl)(phenyl)sulfane (1m)

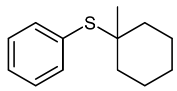

1m
$C_{13}H_{18}S$
M = 206.35 g/mol

Prepared from benzenethiol (550.9 mg, 5.0 mmol) and 3-ethylpentan-3-ol (697.2 mg, 6.0 mmol) according to **GP 1**. Flash column chromatography on silica gel using *n*-pentane afforded substrate **1m** as a colourless oil (937.2 mg, 4.5 mmol, 90% yield).

^{1}**H NMR** (500 MHz, $CDCl_3$, 298 K) δ = 7.55 – 7.49 (m, 2H), 7.38 – 7.28 (m, 3H), 1.84 – 1.72 (m, 2H), 1.71 – 1.61 (m, 2H), 1.54 – 1.41 (m, 5H), 1.39 – 1.30 (m, 1H), 1.22 (s, 3H).

^{13}**C{^{1}H} NMR** (126 MHz, $CDCl_3$, 298 K) δ = 137.8, 132.3, 128.7, 128.5, 50.3, 38.5, 28.9, 26.0, 22.7.

The NMR spectroscopic data are in accordance with those reported.[S14]

Adamantan-1-yl(phenyl)sulfane (1n)

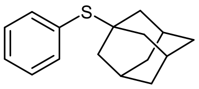

1n
$C_{16}H_{20}S$
M = 244.40 g/mol

Prepared from benzenethiol (550.9 mg, 5.0 mmol) and adamantal-1-ol (913.4 mg, 6.0 mmol) according to **GP 1**. Flash column chromatography on silica gel using *n*-pentane afforded substrate **1n** as a colourless oil (354.3 mg, 1.5 mmol, 29% yield).

^{1}H NMR (500 MHz, $CDCl_3$, 298 K) δ = 7.53 – 7.47 (m, 2H), 7.39 – 7.28 (m, 3H), 2.03 – 1.99 (m, 3H), 1.82 (d, *J* = 3.0 Hz, 6H), 1.68 – 1.56 (m, 6H).

^{13}C{^{1}H} NMR (126 MHz, $CDCl_3$, 298 K) δ = 137.8, 130.8, 128.7, 128.4, 48.0, 43.8, 36.3, 30.2.

The NMR spectroscopic data are in accordance with those reported.[S15]

Phenyl(2-phenylpropan-2-yl)sulfane (1o)

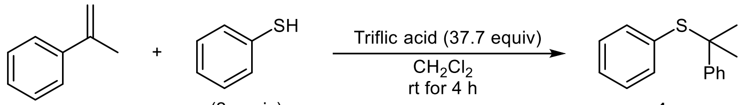

1o
$C_{15}H_{16}S$
M = 228.35 g/mol

According to a literature procedure,[S16] a 50 mL Schlenk-tube with a stir bar was charged with isopropenylbenzene (0.59 g, 5.0 mmol, 1.0 equiv), thiophenol (1.10 g, 10.0 mmol, 2.0 equiv) and CH_2Cl_2 (30 mL). The reaction was cooled to −10 °C and trifluoroacetic acid (4.56 g, 40.0 mmol, 37.7 equiv) was added dropwise to the solution. The reaction was allowed warm to room temperature and stirred for 4 h. The reaction was then cooled to 0 °C and quenched by the slow addition of a saturated aqueous ammonium chloride solution (10 mL). The resulting solution was separated, and the organic layer was washed with 2 M NaOH (2 x 20 mL) followed by brine (2 x 20 mL). The organic layer was then dried over $MgSO_4$, filtered, and the solvent was removed *in vacuo*. Purification of the residue by flash column chromatography on silica gel using *n*-pentane as the eluent afforded phenyl(2-phenylpropan-2-yl)sulfane **1o** in analytically pure form as a colourless oil (900.0 mg, 3.9 mmol, 79% yield).

^{1}H NMR (500 MHz, $CDCl_3$, 298 K) δ = 7.46 (d, *J* = 7.7 Hz, 2H), 7.35 – 7.27 (m, 3H), 7.27 – 7.16 (m, 5H), 1.74 (s, 6H).

^{13}C{^{1}H} NMR (126 MHz, $CDCl_3$, 298 K) δ = 146.6, 136.6, 133.1, 128.6, 128.4, 128.0, 126.70, 126.66, 51.1, 29.9.

The NMR spectroscopic data are in accordance with those reported.[S16]

Phenyl(trityl)sulfane (1p)

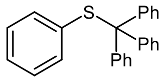

1p
$C_{25}H_{20}S$
M = 352.50 g/mol

Prepared from benzenethiol (550.9 mg, 5.0 mmol) and triphenylmethanol (1302 mg, 6.0 mmol) according to **GP 1**. Flash column chromatography on silica gel using *n*-pentane afforded substrate **1p** as a white solid (852.0 mg, 2.4 mmol, 48% yield).

^{1}H NMR (500 MHz, $CDCl_3$, 298 K) δ = 7.49 – 7.44 (m, 6H), 7.31 – 7.19 (m, 9H), 7.18 – 7.10 (m, 1H), 7.07 – 6.99 (m, 4H).

^{13}C{^{1}H} NMR (126 MHz, $CDCl_3$, 298 K) δ = 144.7, 134.68, 134.66, 130.2, 128.2, 128.1, 127.8, 126.8, 70.9.

The NMR spectroscopic data are in accordance with those reported.[S17]

Benzyl(phenyl)sulfane (1q)

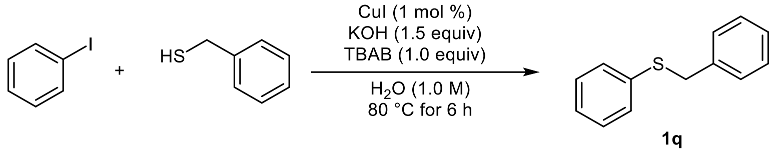

1q
$C_{13}H_{12}S$
M = 200.30 g/mol

According to a literature procedure,[S18] a round bottom flask was charged with water (5 mL), iodobenzene (0.62 mL, 5.5 mmol, 1.1 equiv), benzyl mercaptane (0.59 mL, 5.0 mmol, 1.0 equiv), TBAB (1.61 g, 5.0 mmol, 1.0 equiv) and potassium hydroxide (0.42 g, 7.5 mmol, 1.5 equiv). Copper iodide (9.5 mg, 0.05 mmol, 0.01 equiv) was added and the reaction was heated to 80 °C. The reaction was stirred for 6 h. The reaction was cooled to room temperature and diethyl ether (20 mL) was added. The aqueous layer was separated and extracted with diethyl ether (3 x 10 mL). The combined organic layers were washed with water (10 mL) and brine (10 mL). The organic layer was dried over $MgSO_4$ and concentrated *in vacuo*. Purification of the residue by flash column chromatography on silica gel using *n*-pentane as the eluent afforded substrate **1q** in analytically pure form as a white solid (433.0 mg, 2.2 mmol, 43% yield).

^{1}H NMR (500 MHz, $CDCl_3$, 298 K) δ = 7.36 – 7.13 (m, 10H), 4.13 (s, 2H).

^{13}C{^{1}H} NMR (126 MHz, $CDCl_3$, 298 K) δ = 137.6, 136.5, 130.0, 129.0, 128.6, 128.6, 127.3, 126.5, 39.2.

The NMR spectroscopic data are in accordance with those reported.[S18]

Isopropyl(phenyl)sulfane (1r)

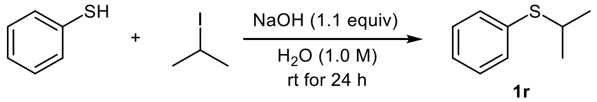

1r
$C_9H_{12}S$
M = 152.26 g/mol

According to a literature procedure,[S19] a round bottom flask was charged with water (5.5 mL) and NaOH (0.22 g, 5.5 mmol, 1.1 equiv). Thiophenol (0.51 mL, 5 mmol, 1.0 equiv) was added dropwise to the reaction mixture. The reaction was stirred for 30 min. Isopropyl iodide (0.5 mL, 5 mmol, 1.0 equiv) in ethanol (1 mL) was added slowly to the reaction. The reaction was stirred at room temperature for 24 h. The product was extracted with CH_2Cl_2 (20 mL), and the combined organic extracts were washed with 1 M NaOH (20 mL) followed by water (20 mL). The organic layer was dried over $MgSO_4$ and concentrated *in vacuo*. Purification of the residue by flash column chromatography on silica gel using *n*-pentane as the eluent afforded substrate **1r** in analytically pure form as a colourless oil (105.0 mg, 0.7 mmol, 14% yield).

^{1}H NMR (500 MHz, $CDCl_3$, 298 K) δ = 7.35 – 7.30 (m, 2H), 7.24 – 7.18 (m, 2H), 7.17 – 7.11 (m, 1H), 3.30 (hept, *J* = 6.8 Hz, 1H), 1.22 (d, *J* = 6.7 Hz, 6H).

^{13}C{^{1}H} NMR (126 MHz, $CDCl_3$, 298 K) δ = 135.7, 132.1, 128.9, 126.82 38.4, 23.3.

The NMR spectroscopic data are in accordance with those reported.[S19]

4. Experimental Details for Thioalkylation of Alkynes

4.1 General procedure for the thioalkylation of alkynes (GP 3)

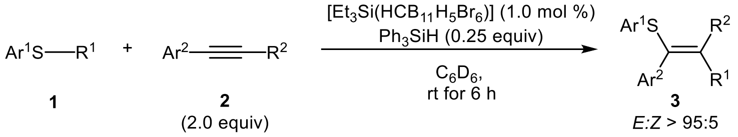

In an argon-filled glovebox, the thioether **1** (0.20 mmol), the alkyne **2** (0.40 mmol, 2.0 equiv), and triphenylsilane (0.050 mmol, 0.25 equiv) are added to a 2-mL vial equipped with a stirbar followed by the addition of C_6D_6 (0.5 mL). [$Et_3Si(HCB_{11}H_5Br_6)$] (1.5 mg, 2.0 μmol, 1.0 mol %) is subsequently added. The resulting mixture is stirred at room temperature for 6 h. Upon completion, the reaction mixture is removed from the glovebox, and CH_2Br_2 (34.8 mg, 0.20 mmol, 1.0 equiv) is then added as an internal standard to determine the yield by ^{1}H NMR spectroscopy. Purification by flash column chromatography on silica gel using *n*-pentane as the eluent affords the carboalkylation product **3** in analytically pure form.

4.2 Characterisation data for products 3

(E)-(2-Ethyl-3,3-dimethyl-1-phenylbut-1-en-1-yl)(phenyl)sulfane (3aa)

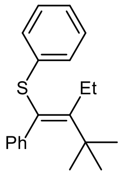

3aa
$C_{20}H_{24}S$
M = 296.47 g/mol

Prepared from **1a** (33.3 mg, 0.2 mmol) and but-1-ynylbenzene (**2a**) (52.1 mg, 0.4 mmol) according to **GP 3.** Flash column chromatography on silica gel using *n*-pentane afforded product **3aa** as a colourless oil (39.2 mg, 0.13 mmol, 66% yield). 2 mmol scale; (426.8 mg, 1.44 mmol, 72% yield). $\mathbf{R_f}$ = 0.52 (pentane).

IR (ATR): ṽ = 3072, 2961, 2869, 1579, 1476, 1238, 1068, 1023, 877, 759, 639.

^{1}H NMR (500 MHz, $CDCl_3$, 298 K) δ = 7.40 – 7.32 (m, 5H), 7.29 – 7.25 (m, 3H), 7.23 – 7.18 (m, 2H), 2.96 (q, *J* = 7.4 Hz, 2H), 1.55 (t, *J* = 7.4 Hz, 3H), 1.24 (s, 9H).

^{13}C{^{1}H} NMR (126 MHz, $CDCl_3$, 298 K) δ = 153.4, 142.4, 134.9, 132.7, 130.2, 128.8, 128.4, 126.9, 126.7, 126.3, 39.2, 31.8, 27.5, 15.8.

HRMS (APCI): calculated for $C_{20}H_{24}S^+$ [M]$^+$: 296.1593; Found 296.1596.

(E)-(2-Ethyl-3,3-dimethyl-1-phenylbut-1-en-1-yl)(p-tolyl)sulfane (3ba)

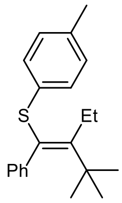

3ba

$C_{21}H_{26}S$

M = 310.50 g/mol

Prepared from **1b** (36.1 mg, 0.2 mmol) and but-1-ynylbenzene (**2a**) (52.1 mg, 0.4 mmol) according to **GP 3.** Flash column chromatography on silica gel using *n*-pentane afforded product **3ba** as a colourless oil (41.6 mg, 0.13 mmol, 67% yield). $\mathbf{R_f}$ = 0.53 (pentane).

IR (ATR): ṽ = 3053, 2962, 2869, 1597, 1487, 1238, 1017, 804, 757, 647.

^{1}H NMR (500 MHz, $CDCl_3$, 298 K) δ = 7.07 – 6.99 (m, 5H), 6.99 – 6.89 (m, 4H), 2.74 (q, *J* = 7.4 Hz, 2H), 2.26 (s, 3H), 1.32 (t, *J* = 7.4 Hz, 3H), 1.00 (s, 8H).

^{13}C{^{1}H} NMR (126 MHz, $CDCl_3$, 298 K) δ = 152.4, 142.4, 136.0, 133.1, 130.3, 130.0, 129.1, 128.2, 126.9, 126.2, 39.1, 31.8, 27.4, 21.2, 15.8.

HRMS (APCI): calculated for $C_{21}H_{26}S^+$ $[M]^+$: 310.1749; Found 310.1749.

(E)-(2-Ethyl-3,3-dimethyl-1-phenylbut-1-en-1-yl)(4-fluorophenyl)sulfane (3ca)

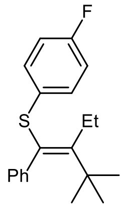

3ca

$C_{20}H_{23}FS$

M = 314.46 g/mol

Prepared from **1c** (36.9 mg, 0.2 mmol) and but-1-ynylbenzene (**2a**) (52.1 mg, 0.4 mmol) according to **GP 3**. Flash column chromatography on silica gel using *n*-pentane afforded product **3ca** as a colourless oil (42.2 mg, 0.13 mmol, 67% yield). $\mathbf{R_f}$ = 0.56 (pentane).

IR (ATR): ṽ = 3055, 2962, 2870, 1588, 1486, 1222, 1087, 826, 758, 698.

^{1}H NMR (500 MHz, $CDCl_3$, 298 K) δ = 7.59 – 7.44 (m, 5H), 7.40 – 7.33 (m, 2H), 7.29 – 7.19 (m, 2H), 3.19 (q, J = 7.4 Hz, 2H), 1.77 (t, J = 7.4 Hz, 3H), 1.45 (s, 9H).

^{13}C{^{1}H} NMR (126 MHz, $CDCl_3$, 298 K) δ = 162.4 (d, *J* = 246.9 Hz), 151.5, 141.8, 135.8 (d, *J* = 8.2 Hz), 131.4, 130.4, 127.7 (d, *J* = 11.1 Hz), 127.0, 126.3, 115.3 (d, *J* = 21.8 Hz), 39.1, 31.8, 27.2, 15.7.

HRMS (APCI): calculated for $C_{20}H_{23}FS^+$ $[M]^+$: 314.1499; Found 314.1497.

(E)-(4-Chlorophenyl)(2-ethyl-3,3-dimethyl-1-phenylbut-1-en-1-yl)sulfane (3da)

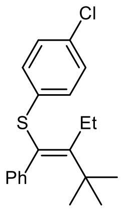

3da
$C_{20}H_{23}ClS$
M = 330.91 g/mol

Prepared from **1d** (40.2 mg, 0.2 mmol) and but-1-ynylbenzene (**2a**) (52.1 mg, 0.4 mmol) according to **GP 3**. Flash column chromatography on silica gel using *n*-pentane afforded product **3da** as a colourless oil (43.5 mg, 0.13 mmol, 66% yield). $\mathbf{R_f}$ = 0.58 (pentane).

IR (ATR): ṽ = 3054, 2962, 2870, 1573, 1472, 1238, 1091, 815, 758, 697.

^{1}H NMR (500 MHz, $CDCl_3$, 298 K) δ = 7.28 – 7.21 (m, 6H), 7.15 (dd, *J* = 6.7, 3.0 Hz, 2H), 2.90 (q, *J* = 7.5 Hz, 2H), 1.49 (t, *J* = 7.5 Hz, 4H), 1.19 (s, 9H).

^{13}C{^{1}H} NMR (126 MHz, $CDCl_3$, 298 K) δ = 153.7, 142.1, 134.0, 133.5, 133.0, 130.2, 128.9, 128.5, 127.1, 126.5, 39.2, 31.8, 27.5, 15.8.

HRMS (APCI): calculated for $C_{20}H_{23}{}^{35}ClS^+$ $[M]^+$: 330.1203; Found 330.1203; calculated for $C_{20}H_{23}{}^{37}ClS^+$ $[M]^+$: 332.1174; Found 332.1173.

(E)-(4-Bromophenyl)(2-ethyl-3,3-dimethyl-1-phenylbut-1-en-1-yl)sulfane (3ea)

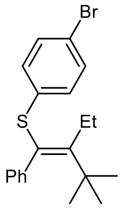

3ea
$C_{20}H_{23}BrS$
M = 375.37 g/mol

Prepared from **1e** (49.0 mg, 0.2 mmol) and but-1-ynylbenzene (**2a**) (52.1 mg, 0.4 mmol) according to **GP 3**. Flash column chromatography on silica gel using *n*-pentane afforded product **3ea** as a colourless oil (54.7 mg, 0.15 mmol, 73% yield). $\mathbf{R_f}$ = 0.62 (pentane).

IR (ATR): ṽ = 3054, 2962, 2869, 1562, 1469, 1237, 1007, 810, 758, 697.

^{1}H NMR (500 MHz, $CDCl_3$, 298 K) δ = 7.45 – 7.40 (m, 2H), 7.31 – 7.23 (m, 3H), 7.18 (ddd, *J* = 11.0, 6.4, 2.1 Hz, 4H), 2.91 (q, *J* = 7.4 Hz, 2H), 1.50 (t, *J* = 7.4 Hz, 4H), 1.21 (s, 9H).

^{13}C{^{1}H} NMR (126 MHz, $CDCl_3$, 298 K) δ = 154.1, 142.1, 134.1, 131.4, 130.2, 129.7, 128.4, 127.1, 126.5, 120.9, 39.2, 31.8, 27.5, 15.8.

HRMS (APCI): calculated for $C_{20}H_{23}{}^{79}BrS^{+}$ [M]$^+$: 374.0698; Found 374.0698; calculated for $C_{20}H_{23}{}^{81}BrS^{+}$ [M]$^+$: 376.0678; Found 376.0677.

***(E)*-(4-(*tert*-Butyl)phenyl)(2-ethyl-3,3-dimethyl-1-phenylbut-1-en-1-yl)sulfane (3fa)**

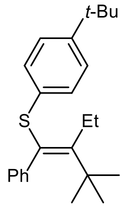

3fa
$C_{24}H_{32}S$
M = 352.58 g/mol

Prepared from **1f** (44.5 mg, 0.2 mmol) and but-1-ynylbenzene (**2a**) (52.1 mg, 0.4 mmol) according to **GP 3** at 60 °C. Flash column chromatography on silica gel using *n*-pentane afforded product **3fa** as a colourless oil (30.4 mg, 0.09 mmol, 43% yield). **R$_f$** = 0.56 (pentane).

IR (ATR): ṽ = 3074, 2958, 2868, 1596, 1479, 1193, 1013, 824, 758, 697.

^{1}H NMR (500 MHz, $CDCl_3$, 298 K) δ = 7.13 – 7.07 (m, 2H), 7.05 – 6.96 (m, 5H), 6.93 – 6.88 (m, 2H), 2.71 (q, *J* = 7.3 Hz, 2H), 1.30 (t, *J* = 7.3 Hz, 5H), 1.25 (s, 9H), 0.98 (s, 9H).

^{13}C{^{1}H} NMR (126 MHz, $CDCl_3$, 298 K) δ = 152.4, 150.1, 142.5, 136.0, 132.8, 131.1, 130.3, 126.8, 126.1, 125.4, 39.1, 34.5, 31.8, 31.4, 27.3, 15.8.

HRMS (APCI): calculated for $C_{24}H_{32}S^{+}$ [M]$^+$: 352.2219; Found 352.2217.

Methyl *(E)*-4-((2-ethyl-3,3-dimethyl-1-phenylbut-1-en-1-yl)thio)benzoate (3ga)

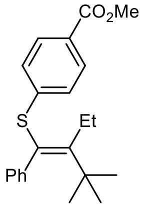

3ga
$C_{22}H_{26}O_2S$
M = 354.51 g/mol

Prepared from **1g** (44.9 mg, 0.2 mmol) and but-1-ynylbenzene (**2a**) (52.1 mg, 0.4 mmol) according to **GP 3** at 60 °C. Flash column chromatography on silica gel using *n*-pentane afforded product **3ga** as a colourless oil (15.6 mg, 0.04 mmol, 22% yield). **R$_f$** = 0.3 (pentane/EtOAc 20:1).

IR (ATR): ṽ = 2952, 1717, 1591, 1484, 1433, 1270, 1174, 1106, 1014, 848, 757, 695.

^{1}H NMR (500 MHz, $CDCl_3$, 298 K) δ = 7.82 – 7.78 (m, 2H), 7.21 – 7.18 (m, 2H), 7.07 – 7.04 (m, 5H), 3.86 (s, 3H), 2.65 (q, *J* = 7.4 Hz, 2H), 1.27 (t, *J* = 7.4 Hz, 3H), 1.02 (s, 9H).

$^{13}C\{^1H\}$ NMR (126 MHz, $CDCl_3$, 298 K) δ = 167.0, 157.8, 142.7, 142.4, 129.9, 129.8, 129.6, 128.5, 127.4, 127.3, 126.7, 52.1, 39.4, 31.8, 27.9, 15.9.

HRMS (APCI): calculated for $C_{22}H_{26}O_2S^+$ $[M]^+$: 354.1648; Found 254.1647.

Ethyl *(E)*-4-((2-ethyl-3,3-dimethyl-1-phenylbut-1-en-1-yl)thio)benzoate (3ha)

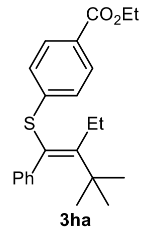

3ha
$C_{23}H_{28}O_2S$
M = 368.54 g/mol

Prepared from **1h** (47.7 mg, 0.2 mmol) and but-1-ynylbenzene (**2a**) (52.1 mg, 0.4 mmol) according to **GP 3** at 60 °C. Flash column chromatography on silica gel using *n*-pentane afforded product **3ha** as a colourless oil (23.6 mg, 0.06 mmol, 32% yield). **R_f** = 0.34 (pentane/EtOAc 20:1).

IR (ATR): ṽ = 3075, 2963, 1713, 1591, 1477, 1364, 1266, 1103, 1016, 849, 758, 697.

^1H NMR (500 MHz, $CDCl_3$, 298 K) δ = 7.84 – 7.78 (m, 2H), 7.22 – 7.17 (m, 2H), 7.10 – 7.01 (m, 5H), 4.33 (q, *J* = 7.1 Hz, 2H), 2.65 (q, *J* = 7.4 Hz, 2H), 1.36 (t, *J* = 7.1 Hz, 3H), 1.27 (t, *J* = 7.4 Hz, 3H), 1.01 (s, 9H).

$^{13}C\{^1H\}$ NMR (126 MHz, $CDCl_3$, 298 K) δ = 166.5, 157.9, 142.8, 142.2, 136.9, 129.9, 129.7, 129.6, 127.9, 127.3, 126.7, 61.0, 39.4, 31.8, 27.9, 15.9, 14.5.

HRMS (APCI): calculated for $C_{23}H_{29}O_2S^+$ $[M-H]^+$: 369.1882; Found 369.1882.

***(E)*-(2-Ethyl-3,3-dimethyl-1-phenylbut-1-en-1-yl)(naphthalen-2-yl)sulfane (3ia)**

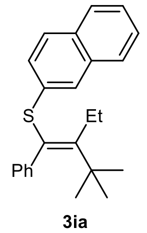

3ia
$C_{24}H_{26}S$
M = 346.53 g/mol

Prepared from **1i** (43.3 mg, 0.2 mmol) and but-1-ynylbenzene (**2a**) (52.1 mg, 0.4 mmol) according to **GP 3**. Flash column chromatography on silica gel using *n*-pentane afforded product **3ia** as a colourless oil (35.4 mg, 0.10 mmol, 51% yield). **R_f** = 0.45 (pentane).

IR (ATR): ṽ = 3051, 2960, 2869, 1586, 1477, 1236, 1130, 1069, 850, 810, 758, 697.

^{1}**H NMR** (500 MHz, $CDCl_3$, 298 K) δ = 7.74 – 7.70 (m, 1H), 7.66 – 7.61 (m, 2H), 7.60 – 7.57 (m, 2H), 7.44 – 7.37 (m, 2H), 7.24 (dd, *J* = 8.5, 1.6 Hz, 1H), 7.03 – 6.95 (m, 4H), 2.77 (q, *J* = 7.4 Hz, 2H), 1.34 (t, *J* = 7.6 Hz, 3H), 1.03 (s, 9H).

^{13}**C{^{1}H} NMR** (126 MHz, $CDCl_3$, 298 K) δ = 154.6, 142.6, 133.6, 132.6, 132.2, 130.7, 130.1, 129.8, 127.8, 127.7, 127.4, 127.0, 126.4, 126.2, 125.8, 39.3, 31.8, 27.6, 15.9.

HRMS (APCI): calculated for $C_{24}H_{26}S^+$ $[M]^+$: 346.1749; Found 346.1747.

(E)-(2-Ethyl-3,3-dimethyl-1-phenylbut-1-en-1-yl)(*m*-tolyl)sulfane (3ja)

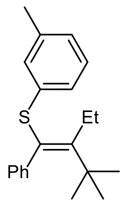

3ja
$C_{21}H_{26}S$
M = 310.50 g/mol

Prepared from **1j** (36.1 mg, 0.2 mmol) and but-1-ynylbenzene (**2a**) (52.1 mg, 0.4 mmol) according to **GP 3**. Flash column chromatography on silica gel using *n*-pentane afforded product **3ja** as a colourless oil (37.4 mg, 0.12 mmol, 60% yield). **R$_f$** = 0.58 (pentane).

IR (ATR): ṽ = 3056, 2961, 2869, 1589, 1467, 1238, 1060, 855, 747, 698.

^{1}**H NMR** (500 MHz, $CDCl_3$, 298 K) δ = 7.30 – 7.17 (m, 7H), 7.16 – 7.10 (m, 2H), 2.94 (q, *J* = 7.4 Hz, 2H), 2.43 (s, 3H), 1.54 (t, *J* = 7.4 Hz, 3H), 1.24 (s, 9H).

^{13}**C{^{1}H} NMR** (126 MHz, $CDCl_3$, 298 K) δ = 153.2, 142.5, 137.9, 136.0, 134.5, 133.3, 130.2, 129.5, 128.2, 127.5, 126.9, 126.2, 39.1, 31.8, 27.4, 21.3, 15.8.

HRMS (APCI): calculated for $C_{21}H_{26}S^+$ $[M]^+$: 310.1749; Found 310.1748.

(E)-(2-Ethyl-3,3-dimethyl-1-phenylbut-1-en-1-yl)(o-tolyl)sulfane (3ka)

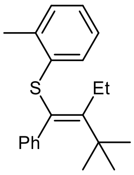

3ka
$C_{21}H_{26}S$
M = 310.50 g/mol

Prepared from **1k** (36.1 mg, 0.2 mmol) and but-1-ynylbenzene (**2a**) (52.1 mg, 0.4 mmol) according to **GP 3**. Flash column chromatography on silica gel using *n*-pentane afforded product **3ka** as a colourless oil (34.7 mg, 11 mmol, 56% yield). **R$_f$** = 0.52 (pentane).

IR (ATR): ṽ = 3057, 2957, 2869, 1589, 1459, 1238, 1060, 854, 747, 697.

^{1}H NMR (500 MHz, $CDCl_3$, 298 K) δ = 7.10 (d, *J* = 7.7 Hz, 1H), 7.04 – 6.95 (m, 5H), 6.94 – 6.85 (m, 3H), 2.74 (q, *J* = 7.4 Hz, 2H), 2.26 (s, 3H), 1.31 (t, *J* = 7.4 Hz, 3H), 0.99 (s, 9H).

$^{13}C\{^1H\}$ NMR (126 MHz, $CDCl_3$, 298 K) δ = 152.4, 142.3, 140.9, 134.4, 133.7, 129.9, 129.8, 128.2, 127.3, 126.8, 126.2, 125.8, 39.1, 31.8, 27.4, 20.8, 15.8.

HRMS (APCI): calculated for $C_{21}H_{26}S^+$ $[M]^+$: 310.1749; Found 310.1748.

***(E)*-(2-Ethyl-3,3-dimethyl-1-phenylpent-1-en-1-yl)(phenyl)sulfane (3la)**

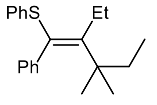

3la

$C_{21}H_{26}S$

M = 310.50 g/mol

Prepared from **1l** (36.1 mg, 0.2 mmol) and but-1-ynylbenzene (**2a**) (52.1 mg, 0.4 mmol) according to **GP 3**. Flash column chromatography on silica gel using *n*-pentane afforded product **3la** as a colourless oil (25.2 mg, 0.08 mmol, 41% yield). **R_f** = 0.57 (pentane).

IR (ATR): ṽ = 3056, 2962, 2928, 2871, 1579, 1475, 1069, 1024, 801, 741, 697.

^{1}H NMR (500 MHz, $CDCl_3$, 298 K) δ = 7.14 – 7.03 (m, 5H), 6.99 – 6.94 (m, 3H), 6.93 – 6.88 (m, 2H), 2.64 (q, *J* = 7.4 Hz, 2H), 1.29 (dt, *J* = 19.5, 7.4 Hz, 5H), 0.89 (s, 5H), 0.79 (t, *J* = 7.4 Hz, 3H).

$^{13}C\{^1H\}$ NMR (126 MHz, $CDCl_3$, 298 K) δ = 151.5, 142.2, 134.8, 133.1, 131.6, 130.2, 128.3, 126.8, 126.8, 126.1, 42.8, 35.4, 29.7, 27.1, 15.6, 9.9.

HRMS (APCI): calculated for $C_{21}H_{26}S^+$ $[M]^+$: 310.1749; Found 310.1748.

(*(E)*-2-((3r,5r,7r)-Adamantan-1-yl)-1-phenylbut-1-en-1-yl)(phenyl)sulfane (3na)

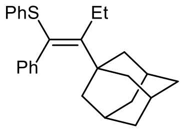

3na

$C_{26}H_{30}S$

M = 374.59 g/mol

Prepared from **1n** (48.9 mg, 0.2 mmol) and but-1-ynylbenzene (**2a**) (52.1 mg, 0.4 mmol) according to **GP 3** at 60 °C. Flash column chromatography on silica gel using *n*-pentane afforded product **3na** as a colourless oil (49.7 mg, 0.13 mmol, 66% yield). **R_f** = 0.52 (pentane).

IR (ATR): ṽ = 3068, 2898, 2846, 1578, 1439, 1312, 1068, 800, 728, 689.

^{1}H NMR (500 MHz, $CDCl_3$, 298 K) δ = 7.16 – 7.06 (m, 4H), 7.02 – 6.96 (m, 3H), 6.96 – 6.90 (m, 2H), 2.70 (q, *J* = 7.4 Hz, 2H), 1.86 – 1.81 (m, 3H), 1.68 (d, *J* = 3.1 Hz, 6H), 1.60 – 1.48 (m, 6H), 1.28 (t, *J* = 7.4 Hz, 3H).

^{13}C{^{1}H} NMR (126 MHz, $CDCl_3$, 298 K) δ = 153.6, 142.5, 136.0, 134.9, 132.7, 130.1, 128.3, 126.8, 126.7, 126.1, 42.3, 42.0, 36.8, 29.0, 26.2, 16.0.

HRMS (APCI): calculated for $C_{26}H_{30}S^+$ $[M]^+$: 374.2062; Found 374.2065.

***(E)*-(2-Ethyl-3-methyl-1,3-diphenylbut-1-en-1-yl)(phenyl)sulfane (3oa)**

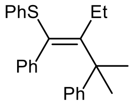

3oa
$C_{25}H_{26}S$
M = 358.54 g/mol

Prepared from **1o** (45.7 mg, 0.2 mmol) and but-1-ynylbenzene (**2a**) (52.1 mg, 0.4 mmol) according to **GP 3**. Flash column chromatography on silica gel using *n*-pentane afforded product **3oa** as a colourless oil (24.0 mg, 0.07 mmol, 33% yield). $\mathbf{R_f}$ = 0.57 (pentane).

IR (ATR): ṽ = 3057, 3017, 2959, 2926, 2864, 1597, 1458, 1071, 1022, 752, 698.

^{1}H NMR (500 MHz, $CDCl_3$, 298 K) δ = 7.39 – 7.21 (m, 6H), 7.19 – 6.97 (m, 4H), 2.32 (q, *J* = 7.6 Hz, 2H), 1.29 (s, 6H), 0.95 (t, *J* = 7.6 Hz, 3H).

^{13}C{^{1}H} NMR (126 MHz, $CDCl_3$, 298 K) δ = 155.1, 153.1, 144.1, 136.5, 136.2, 129.2, 128.8, 128.5, 128.3, 127.1, 126.5, 124.6, 121.2, 119.7, 50.8, 24.7, 18.8, 14.6.

HRMS (APCI): calculated for $C_{19}H_{21}^+$ $[M{-}SPh]^+$: 249.1637; Found 249.1637.

***(E)*-(2-Ethyl-3,3-dimethyl-1-phenylbut-1-en-1-yl)(methyl)sulfane (3sa)**

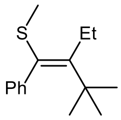

3sa
$C_{15}H_{22}S$
M = 234.40 g/mol

Prepared from 2-methyl-2-propanethiol (20.8 mg, 0.2 mmol) and but-1-ynylbenzene (**2a**) (52.1 mg, 0.4 mmol) according to **GP 3**. Flash column chromatography on silica gel using *n*-pentane afforded product **3sa** as a colourless oil (4.6 mg, 0.02 mmol, 10% yield). $\mathbf{R_f}$ = 0.68 (pentane).

IR (ATR): ṽ = 3055, 2959, 2920, 1600, 1477, 1239, 1069, 880, 760, 699.

^{1}H NMR (500 MHz, $CDCl_3$, 298 K) δ = 7.22 – 7.17 (m, 2H), 7.16 – 7.11 (m, 1H), 7.07 – 7.03 (m, 2H), 2.46 (q, *J* = 7.5 Hz, 2H), 1.55 (s, 3H), 1.12 (t, *J* = 7.3 Hz, 3H), 0.84 (s, 9H).

^{13}C{^{1}H} NMR (126 MHz, $CDCl_3$, 298 K) δ = 147.5, 141.0, 130.7, 128.5, 127.6, 126.7, 38.9, 31.7, 26.7, 15.5, 14.9.

HRMS (APCI): calculated for $C_{15}H_{22}S^+$ $[M]^+$: 234.1436; Found 234.1437.

(E)-Phenyl(2,3,3-trimethyl-1-phenylbut-1-en-1-yl)sulfane (3ab)

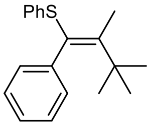

3ab
$C_{19}H_{22}S$
M = 282.45 g/mol

Prepared from **1a** (33.3 mg, 0.2 mmol) and prop-1-ynylbenzene (**2b**) (46.5 mg, 0.4 mmol) according to **GP 3**. Flash column chromatography on silica gel using *n*-pentane afforded product **3ab** as a colourless oil (29.0 mg, 0.10 mmol, 51% yield). $\mathbf{R_f}$ = 0.52 (pentane).

IR (ATR): ṽ = 3055, 2955, 2906, 1579, 1474, 1257, 1024, 885, 738, 698.

^{1}H NMR (500 MHz, $CDCl_3$, 298 K) δ = 7.19 – 7.11 (m, 4H), 7.10 – 7.01 (m, 6H), 2.24 (s, 3H), 0.99 (s, 9H).

^{13}C{^{1}H} NMR (126 MHz, $CDCl_3$, 298 K) δ = 149.5, 143.1, 135.7, 130.9, 129.9, 129.1, 128.5, 127.2, 126.5, 126.2, 39.0, 31.4, 21.0.

HRMS (APCI): calculated for $C_{19}H_{22}S^+$ $[M]^+$: 282.1437; Found 282.1436.

(E)-Phenyl(2,3,3-trimethyl-1-(p-tolyl)but-1-en-1-yl)sulfane (3ac)

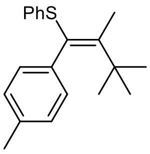

3ac
$C_{20}H_{24}S$
M = 296.47 g/mol

Prepared from **1a** (33.3 mg, 0.2 mmol) and 1-methyl-4-prop-1-ynylbenzene (**2c**) (52.1 mg, 0.4 mmol) according to **GP 3**. Flash column chromatography on silica gel using *n*-pentane afforded product **3ac** as a colourless oil (32.0 mg, 0.11 mmol, 54% yield). $\mathbf{R_f}$ = 0.55 (pentane).

IR (ATR): ṽ = 3053, 2953, 2916, 2863, 1580, 1474, 1193, 893, 810, 735, 689.

^{1}H NMR (500 MHz, $CDCl_3$, 298 K) δ = 7.29 – 7.23 (m, 4H), 7.22 – 7.12 (m, 2H), 7.04 (d, *J* = 7.9 Hz, 2H), 7.00 (d, *J* = 7.7 Hz, 2H), 2.34 (s, 3H), 2.32 (s, 3H), 1.09 (s, 9H).

^{13}C{^{1}H} NMR (126 MHz, $CDCl_3$, 298 K) δ = 150.2, 140.4, 137.6, 136.13, 136.06, 130.2, 129.7, 128.5, 128.0, 125.9, 38.9, 31.4, 21.3, 21.1.

HRMS (APCI): calculated for $C_{20}H_{24}S^+$ $[M]^+$: 296.1593; Found 296.1591.

(E)-(1-(4-Fluorophenyl)-2,3,3-trimethylbut-1-en-1-yl)(phenyl)sulfane (3ad)

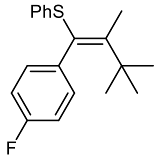

3ad
$C_{19}H_{21}FS$
M = 300.44 g/mol

Prepared from **1a** (33.3 mg, 0.2 mmol) and 1-fluoro-4-prop-1-ynylbenzene (**2d**) (53.7 mg, 0.4 mmol) according to **GP 3**. Flash column chromatography on silica gel using *n*-pentane afforded product **3ad** as a colourless oil (32.0 mg, 0.11 mmol, 54% yield). $\mathbf{R_f}$ = 0.55 (pentane).

IR (ATR): ṽ = 3061, 2958, 2909, 1581, 1501, 1475, 1221, 1090, 826, 735, 689.

^{1}H NMR (500 MHz, $CDCl_3$, 298 K) δ = 7.17 – 7.08 (m, 5H), 7.01 – 6.94 (m, 2H), 6.80 – 6.73 (m, 2H), 2.24 (s, 3H), 0.98 (s, 9H).

^{13}C{^{1}H} NMR (126 MHz, $CDCl_3$, 298 K) δ = 161.6 (d, *J* = 245.5 Hz), 149.5, 138.9 (d, *J* = 3.6 Hz), 135.2, 131.5, 131.4, 128.6, 128.4 (d, *J* = 9.5 Hz), 126.6, 114.1 (d, *J* = 21.5 Hz), 38.9, 31.4, 21.0.

HRMS (APCI): calculated for $C_{19}H_{21}FS^+$ $[M]^+$: 300.1342; Found 300.1342.

(E)-(1-(4-Chlorophenyl)-2,3,3-trimethylbut-1-en-1-yl)(phenyl)sulfane (3ae)

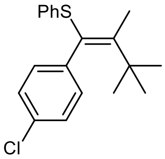

3ae
$C_{19}H_{21}ClS$
M = 316.89 g/mol

Prepared from **1a** (33.3 mg, 0.2 mmol) and 1-chloro-4-prop-1-ynylbenzene (**2e**) (60.2 mg, 0.4 mmol) according to **GP 3**. Flash column chromatography on silica gel using *n*-pentane afforded product **3ae** as a colourless oil (48.3 mg, 0.15 mmol, 76% yield). $\mathbf{R_f}$ = 0.53 (pentane).

IR (ATR): ṽ = 3053, 2958, 2906, 1578, 1472, 1257, 1086, 1011, 892, 815, 735, 688.

^{1}H NMR (500 MHz, $CDCl_3$, 298 K) δ = 7.08 – 7.00 (m, 5H), 6.98 – 6.93 (m, 2H), 6.88 – 6.83 (m, 2H), 2.15 (s, 3H), 0.89 (s, 9H).

^{13}C{^{1}H} NMR (126 MHz, $CDCl_3$, 298 K) δ = 150.2, 141.6, 135.2, 132.3, 131.18, 131.15, 128.9, 128.7, 127.4, 126.6, 39.0, 31.5, 21.1.

HRMS (APCI): calculated for $C_{19}H_{21}{}^{35}ClS^+$ $[M]^+$: 316.1047; Found 316.1044; calculated for $C_{19}H_{21}{}^{37}ClS^+$ $[M]^+$: 318.1017; Found 318.1017.

(E)-(1-(4-bromophenyl)-2,3,3-trimethylbut-1-en-1-yl)(phenyl)sulfane (3af)

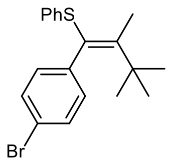

3af
$C_{19}H_{21}BrS$
M = 361.34 g/mol

Prepared from **1a** (33.3 mg, 0.2 mmol) and 1-bromo-4-prop-1-ynylbenzene (**2f**) (78.0 mg, 0.4 mmol) according to **GP 3**. Flash column chromatography on silica gel using *n*-pentane afforded product **3af** as a colourless oil (39.3 mg, 0.11 mmol, 54% yield). $\mathbf{R_f}$ = 0.57 (pentane).

IR (ATR): ṽ = 3058, 2960, 2909, 1577,1471, 1258, 1068, 1009, 887, 812, 738, 686.

^{1}H NMR (500 MHz, $CDCl_3$, 298 K) δ = 7.12 (d, *J* = 8.3 Hz, 2H), 7.09 – 6.99 (m, 5H), 6.80 (d, *J* = 8.3 Hz, 2H), 2.15 (s, 3H), 0.89 (s, 9H).

^{13}C{^{1}H} NMR (126 MHz, $CDCl_3$, 298 K) δ = 150.4, 142.1, 135.2, 131.5, 131.1, 130.4, 128.7, 128.0, 126.6, 120.5, 39.0, 31.5, 21.1.

HRMS (APCI): calculated for $C_{19}H_{21}{}^{79}BrS^+$ $[M]^+$: 360.0541; Found 360.0540; calculated for $C_{19}H_{21}{}^{81}BrS^+$ $[M]^+$: 362.0521; Found 362.0518.

(E)-(1-(4-(*tert*-Butyl)phenyl)-2,3,3-trimethylbut-1-en-1-yl)(phenyl)sulfane (3ag)

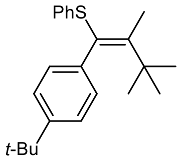

3ag
$C_{23}H_{30}S$
M = 338.55 g/mol

Prepared from **1a** (33.3 mg, 0.2 mmol) and 1-tert-butyl-4-prop-1-ynylbenzene (**2g**) (68.9 mg, 0.4 mmol) according to **GP 3**. Flash column chromatography on silica gel using *n*-pentane afforded product **3ag** as a colourless oil (24.5 mg, 0.07 mmol, 36% yield). $\mathbf{R_f}$ = 0.53 (pentane).

IR (ATR): ṽ = 3028, 2964, 1558, 1457, 1260 1091, 1019, 845, 797, 699.

^{1}H NMR (500 MHz, $CDCl_3$, 298 K) δ = 7.20 – 7.03 (m, 7H), 6.97 – 6.91 (m, 2H), 2.19 (s, 3H), 1.23 (s, 9H), 0.98 (s, 9H).

^{13}C{^{1}H} NMR (126 MHz, $CDCl_3$, 298 K) δ = 149.5, 140.2, 136.0, 130.5, 130.0, 129.4, 128.5, 128.2, 125.9, 124.1, 38.9, 34.5, 31.5, 31.4, 30.5, 20.9.

HRMS (APCI): calculated for $C_{23}H_{30}S^+$ $[M]^+$: 338.2062; Found 338.2062.

(E)-Phenyl(2,3,3-trimethyl-1-(m-tolyl)but-1-en-1-yl)sulfane (3ah)

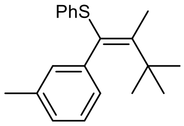

3ah
$C_{20}H_{24}S$
M = 296.47 g/mol

Prepared from **1a** (33.3 mg, 0.2 mmol) and 1-methyl-3-prop-1-ynylbenzene (**2h**) (52.1 mg, 0.4 mmol) according to **GP 3**. Flash column chromatography on silica gel using *n*-pentane afforded product **3ah** as a colourless oil (42.8 mg, 0.14 mmol, 72% yield). **R**$_f$ = 0.47 (pentane).

IR (ATR): ṽ = 3053, 2953, 2915, 1580, 1475, 1023, 858, 779, 737, 689.

^{1}H NMR (500 MHz, $CDCl_3$, 298 K) δ = 7.19 – 7.10 (m, 5H), 6.98 (t, *J* = 7.9 Hz, 1H), 6.92 – 6.81 (m, 3H), 2.22 (s, 3H), 2.21 (s, 3H), 1.00 (s, 9H).

^{13}C{^{1}H} NMR (126 MHz, $CDCl_3$, 298 K) δ = 149.4, 143.1, 136.7, 135.9, 130.63, 130.59, 129.0, 128.5, 127.2, 127.1, 126.9, 126.1, 39.0, 31.4, 21.4, 21.0.

HRMS (APCI): calculated for $C_{20}H_{24}S^+$ $[M]^+$: 296.1593; Found 296.1594.

(E)-(1-(3-Fluorophenyl)-2,3,3-trimethylbut-1-en-1-yl)(phenyl)sulfane (3ai)

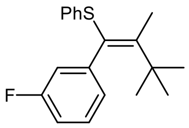

3ai
$C_{19}H_{21}FS$
M = 300.44 g/mol

Prepared from **1a** (33.3 mg, 0.2 mmol) and 1-fluoro-3-prop-1-ynylbenzene (**2i**) (53.7 mg, 0.4 mmol) according to **GP 3**. Flash column chromatography on silica gel using *n*-pentane afforded product **3ai** as a colourless oil (20.8 mg, 0.07 mmol, 35% yield). **R**$_f$ = 0.52 (pentane).

IR (ATR): ṽ = 3072, 2961, 2925, 1580, 1476, 1261, 1149, 1024, 809, 741, 691.

^{1}H NMR (500 MHz, $CDCl_3$, 298 K) δ = 7.18 – 7.08 (m, 5H), 7.05 – 6.98 (m, 1H), 6.82 – 6.70 (m, 3H), 2.24 (s, 3H), 0.99 (s, 9H).

^{13}C{^{1}H} NMR (126 MHz, $CDCl_3$, 298 K) δ = 162.1 (d, *J* = 245.4 Hz), 150.0, 145.1 (d, *J* = 7.7 Hz), 135.1, 131.2, 128.9, 128.7, 128.5 (d, *J* = 8.4 Hz), 126.6, 125.9 (d, *J* = 2.9 Hz), 116.9 (d, *J* = 21.2 Hz), 113.4 (d, *J* = 21.0 Hz), 39.0, 31.3, 21.0.

HRMS (APCI): calculated for $C_{19}H_{21}FS^+$ $[M]^+$: 300.1342; Found 300.1340.

(E)-(1-(3-Chlorophenyl)-2,3,3-trimethylbut-1-en-1-yl)(phenyl)sulfane (3aj)

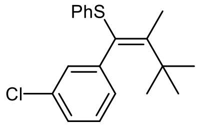

3aj
$C_{19}H_{21}ClS$
M = 316.89 g/mol

Prepared from **1a** (33.3 mg, 0.2 mmol) and 1-chloro-3-prop-1-ynylbenzene (**2j**) (60.2 mg, 0.4 mmol) according to **GP 3**. Flash column chromatography on silica gel using *n*-pentane afforded product **3aj** as a colourless oil (25.8 mg, 0.08 mmol, 41% yield). $\mathbf{R_f}$ = 0.52 (pentane).

IR (ATR): ṽ = 3059, 2958, 2908, 1561, 1473, 1194, 1077, 855, 783, 738, 689.

^{1}H NMR (500 MHz, $CDCl_3$, 298 K) δ = 7.20 – 7.07 (m, 5H), 7.06 – 6.93 (m, 3H), 6.88 (dt, *J* = 7.5, 1.6 Hz, 1H), 2.23 (d, *J* = 2.0 Hz, 3H), 0.99 (s, 9H).

^{13}C{^{1}H} NMR (126 MHz, $CDCl_3$, 298 K) δ = 150.0, 144.7, 135.0, 133.1, 131.4, 131.3, 130.1, 130.0, 128.7, 128.1, 127.9, 126.6, 39.0, 31.4, 21.0.

HRMS (APCI): calculated for $C_{19}H_{21}{}^{35}ClS^+$ $[M]^+$: 316.1047; Found 316.1046; calculated for $C_{19}H_{21}{}^{37}ClS^+$ $[M]^+$: 318.1017; Found 318.1016.

(E)-(1-(3-Bromophenyl)-2,3,3-trimethylbut-1-en-1-yl)(phenyl)sulfane (3ak)

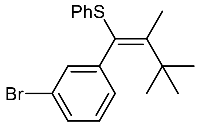

3ak
$C_{19}H_{21}BrS$
M = 361.34 g/mol

Prepared from **1a** (33.3 mg, 0.2 mmol) and 1-bromo-3-prop-1-ynylbenzene (**2k**) (78.0 mg, 0.4 mmol) according to **GP 3**. Flash column chromatography on silica gel using *n*-pentane afforded product **3ak** as a colourless oil (7.5 mg, 0.02 mmol, 10% yield). $\mathbf{R_f}$ = 0.48 (pentane).

IR (ATR): ṽ = 3056, 2956, 1471, 1398, 1256, 1192, 1069, 855, 781, 735, 688.

^{1}H NMR (500 MHz, $CDCl_3$, 298 K) δ = 7.21 – 7.05 (m, 7H), 6.99 – 6.89 (m, 2H), 2.22 (s, 3H), 0.98 (s, 9H).

^{13}C{^{1}H} NMR (126 MHz, $CDCl_3$, 298 K) δ = 149.9, 144.9, 135.7, 134.9, 132.9, 131.5, 129.5, 128.7, 128.6, 128.2, 126.7, 121.3, 39.1, 31.5, 20.9.

HRMS (APCI): calculated for $C_{19}H_{21}{}^{79}BrS^+$ $[M]^+$: 360.0541; Found 360.0539; calculated for $C_{19}H_{21}{}^{81}BrS^+$ $[M]^+$: 362.0521; Found 362.0521.

(E)-Phenyl(2,3,3-trimethyl-1-(o-tolyl)but-1-en-1-yl)sulfane (3al)

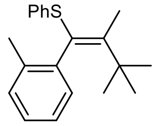

3al
$C_{20}H_{24}S$
M = 296.47 g/mol

Prepared from **1a** (33.3 mg, 0.2 mmol) and 1-methyl-2-prop-1-ynylbenzene (**2l**) (52.1 mg, 0.4 mmol) according to **GP 3**. Flash column chromatography on silica gel using *n*-pentane afforded product **3al** as a colourless oil (42.3 mg, 0.14 mmol, 71% yield). **R$_f$** = 0.53 (pentane).

IR (ATR): ṽ = 3058, 2954, 2908, 1580, 1475, 1257, 1190, 1023, 890, 730, 689.

^{1}H NMR (500 MHz, $CDCl_3$, 298 K) δ = 7.07 – 6.99 (m, 5H), 6.89 (d, *J* = 4.3 Hz, 2H), 6.76 – 6.70 (m, 1H), 6.65 (d, *J* = 7.5 Hz, 1H), 2.17 (s, 3H), 2.14 (s, 3H), 0.86 (s, 9H).

^{13}C{^{1}H} NMR (126 MHz, $CDCl_3$, 298 K) δ = 146.6, 141.5, 136.1, 132.4, 130.3, 129.7, 128.3, 126.9, 126.7, 124.4, 39.1, 30.7, 20.3, 20.1.

HRMS (APCI): calculated for $C_{20}H_{24}S^+$ $[M]^+$: 296.1593; Found 296.1591.

(E)-(1-(2-Fluorophenyl)-2,3,3-trimethylbut-1-en-1-yl)(phenyl)sulfane (3am)

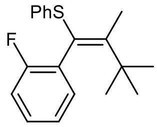

3am
$C_{19}H_{21}FS$
M = 300.44 g/mol

Prepared from **1a** (33.3 mg, 0.2 mmol) and 1-fluoro-2-prop-1-ynylbenzene (**2m**) (53.7 mg, 0.4 mmol) according to **GP 3**. Flash column chromatography on silica gel using *n*-pentane afforded product **3am** as a colourless oil (28.4 mg, 0.09 mmol, 47% yield). **R$_f$** = 0.44 (pentane).

IR (ATR): ṽ = 3057, 2958, 1578, 1479, 1440, 1192, 1024, 805, 754, 689.

^{1}H NMR (500 MHz, $CDCl_3$, 298 K) δ = 7.23 – 7.17 (m, 2H), 7.15 – 7.08 (m, 3H), 7.07 – 7.01 (m, 1H), 6.99 – 6.90 (m, 1H), 6.86 – 6.76 (m, 2H), 2.24 (s, 3H), 1.00 (s, 9H).

^{13}C{^{1}H} NMR (126 MHz, $CDCl_3$, 298 K) δ = 159.5 (d, *J* = 244.6 Hz), 150.3, 135.0 (d, *J* = 41.1 Hz), 131.84, 131.75 (d, *J* = 3.2 Hz), 128.7 (d, *J* = 8.1 Hz), 128.5, 127.8, 126.7, 123.0 (d, *J* = 3.5 Hz), 122.4, 115.1 (d, *J* = 22.5 Hz), 39.0, 30.6, 20.5.

HRMS (APCI): calculated for $C_{19}H_{21}FS^+$ $[M]^+$: 300.1342; Found 300.1342.

(*E*)-(1-(2-Chlorophenyl)-2,3,3-trimethylbut-1-en-1-yl)(phenyl)sulfane (3an)

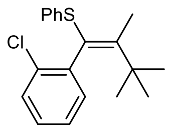

3an
$C_{19}H_{21}ClS$
M = 316.89 g/mol

Prepared from **1a** (33.3 mg, 0.2 mmol) and 1-chloro-2-prop-1-ynylbenzene (**2n**) (60.2 mg, 0.4 mmol) according to **GP 3**. Flash column chromatography on silica gel using *n*-pentane afforded product **3an** as a colourless oil (39.5 mg, 0.12 mmol, 62% yield). $\mathbf{R_f}$ = 0.47 (pentane).

IR (ATR): ṽ = 3065, 2956, 2906, 1580, 1465, 1427, 1194, 1057, 894, 801, 736, 690.

^{1}H NMR (500 MHz, $CDCl_3$, 298 K) δ = 7.23 – 7.18 (m, 3H), 7.16 – 7.09 (m, 3H), 7.05 – 6.97 (m, 1H), 6.93 – 6.84 (m, 2H), 2.25 (s, 3H), 1.00 (s, 9H).

^{13}C{^{1}H} NMR (126 MHz, $CDCl_3$, 298 K) δ = 149.2, 141.1, 136.0, 132.2, 131.4, 130.0, 129.3, 128.5, 128.2, 128.0, 126.8, 125.4, 39.2, 30.5, 20.2.

HRMS (APCI): calculated for $C_{19}H_{21}{}^{35}ClS^+$ $[M]^+$: 316.1047; Found 316.1046; calculated for $C_{19}H_{21}{}^{37}ClS^+$ $[M]^+$: 318.1017; Found 318.1017.

(*Z*)-(3,3-Dimethyl-1,2-diphenylbut-1-en-1-yl)(phenyl)sulfane (3ao)

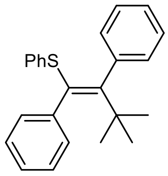

3ao
$C_{24}H_{24}S$
M = 344.52 g/mol

Prepared from **1a** (33.3 mg, 0.2 mmol) and diphenylacetylene (**2o**) (71.3 mg, 0.4 mmol) according to **GP 3**. Flash column chromatography on silica gel using *n*-pentane afforded product **3ao** as a colourless oil (28.1 mg, 0.08 mmol, 41% yield). $\mathbf{R_f}$ = 0.25 (pentane/1% NEt_3).

IR (ATR): ṽ = 3054, 2952, 1576, 1474, 1192, 1069, 1024, 875, 785, 752, 697.

^{1}H NMR (500 MHz, $CDCl_3$, 298 K) δ = 7.71 – 7.65 (m, 2H), 7.62 – 7.57 (m, 1H), 7.56 – 7.51 (m, 2H), 7.34 – 7.23 (m, 10H), 1.19 (s, 9H).

^{13}C{^{1}H} NMR (126 MHz, $CDCl_3$, 298 K) δ = 150.0, 143.7, 140.4, 134.9, 134.6, 134.3, 130.5, 129.4, 128.1, 128.0, 127.2, 127.1, 126.61, 126.57, 38.1, 32.3.

HRMS (APCI): calculated for $C_{24}H_{24}S^+$ $[M]^+$: 344.1593; Found 344.1592.

5. Substituent Scrambling experiment

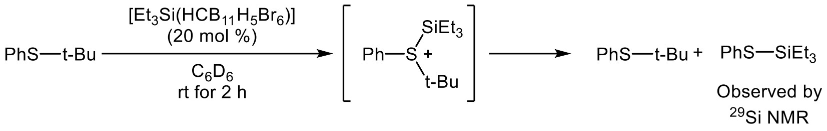

In an argon filled glovebox, in a 2 mL vial equipped with a stirbar *tert*-butyl(phenyl)sulfane (**1a**) was dissolved in C_6D_6 (0.25 mL). [$Et_3Si(HCB_{11}H_5Br_6)$] (14 mg, 0.04 mmol, 0.2 equiv) was added and the reaction was stirred for 2 h. The reaction mixture was transferred to an NMR tube and the presence of triethyl(phenylthio)silane was confirmed by ^{29}Si NMR.[S20]

Figure S1: ^1H ^{29}Si–HMQC 7Hz NMR spectrum of mixture showing ^{29}Si peak of triethyl(phenylthio)silane

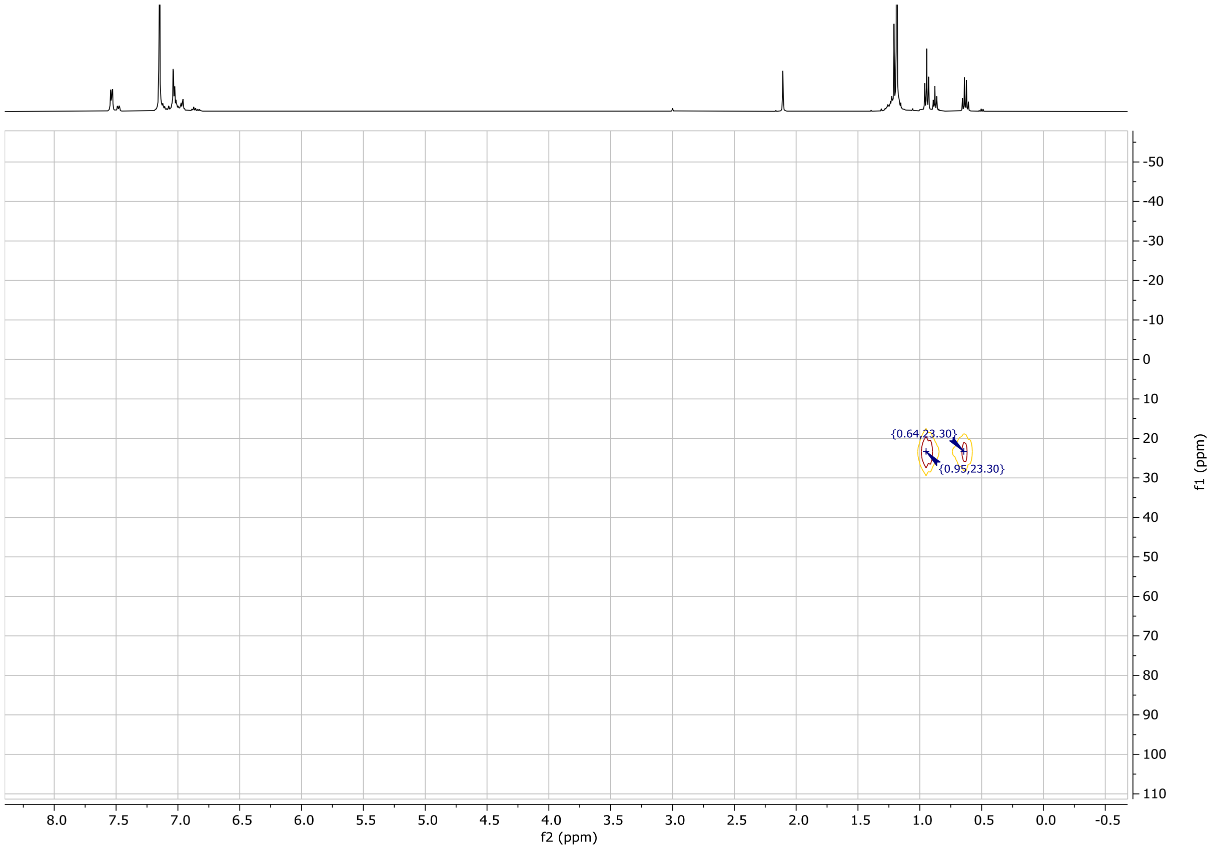

6. Synthetic Transformations of 3

(E)-(2-Ethyl-3,3-dimethyl-1-(phenylsulfinyl)but-1-en-1-yl)benzene (12)

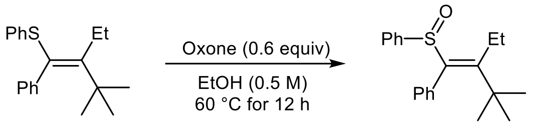

12
$C_{20}H_{24}OS$
M = 312.47 g/mol

According to a literature procedure,[S21] compound **3aa** (59.3 mg, 0.20 mmol, 1.0 equiv), oxone (43.4 mg, 0.12 mmol, 0.6 equiv) and ethanol (1.0 mL) were added to a Schlenk tube. The reaction was heated to 60 °C and stirred for 16 h. The reaction was cooled to room temperature and water (5 mL) was added. The organic layer was extracted with ethyl acetate (3 x 10 mL). The combined organic layer was then dried over $MgSO_4$, filtered, and the solvent was removed *in vacuo*. Purification of the residue by flash column chromatography on silica gel using *n*-pentane/EtOAc (98/2) as the eluent afforded product **12** in analytically pure form as a white solid (42.3 mg, 0.14 mmol, 68% yield). $\mathbf{R_f}$ **=** 0.64 (*n*-pentane/EtOAc = 25/1).

IR (ATR): ṽ = 3051, 2960, 1584, 1476, 1441, 1286, 1191, 1145, 1081, 1033, 873, 758, 699.

^{1}H NMR (500 MHz, $CDCl_3$, 298 K) δ = 7.28 – 7.04 (m, 9H), 6.74 – 6.67 (m, 1H), 5.87 – 5.81 (m, 1H), 2.92 – 2.77 (m, 2H), 1.38 (t, *J* = 7.8 Hz, 3H), 0.89 (d, *J* = 1.5 Hz, 9H).

^{13}C{^{1}H} NMR (126 MHz, $CDCl_3$, 298 K) δ = 158.9, 142.9, 142.4, 132.6, 131.9, 130.9, 130.1, 128.4, 127.5, 126.7, 126.1, 124.8, 39.5, 31.1, 24.6, 17.4.

HRMS (APCI): calculated for $C_{20}H_{25}OS^+$ $[M{-}H]^+$: 313.1620; Found 313.1620

(E)-(2-Ethyl-3,3-dimethyl-1-(phenylsulfonyl)but-1-en-1-yl)benzene (12)

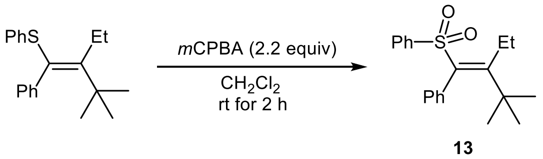

13
$C_{20}H_{24}O_2S$
M = 328.47 g/mol

According to a literature procedure,[S22] compound **3aa** (59.3 mg, 0.20 mmol, 1.0 equiv) was weighed into a Schlenk flask and dissolved in CH_2Cl_2 (1.0 mL). Then, mCPBA (75.9 mg, 0.44 mmol, 2.2 equiv) was added in one portion, and the resulting mixture was stirred at room temperature for 2 h. Upon completion (monitored by TLC), all volatiles were evaporated under

vacuum. Flash column chromatography on silica gel using *n*-pentane/EtOAc (98/2) afforded product **12** as a white solid (40.4 mg, 0.12 mmol, 61% yield). **R_f =** 0.53 (*n*-pentane/EtOAc = 25/1).

IR (ATR): ṽ = 3060, 2960, 1477, 1446, 1363, 1287, 1193, 1143, 1078, 872, 766, 704.

^{1}H NMR (500 MHz, $CDCl_3$, 298 K) δ = 7.45 – 7.39 (m, 2H), 7.37 – 7.29 (m, 1H), 7.23 – 7.17 (m, 2H), 7.11 – 7.05 (m, 1H), 6.99 (dd, *J* = 8.5, 6.9 Hz, 2H), 6.90 – 6.84 (m, 2H), 2.93 (q, *J* = 7.3 Hz, 2H), 1.28 (t, *J* = 7.4 Hz, 3H), 0.85 (s, 9H).

^{13}C{^{1}H} NMR (126 MHz, $CDCl_3$, 298 K) δ = 162.5, 141.5, 140.2, 135.7, 133.3, 132.5, 130.3, 128.4, 128.3, 128.2, 127.8, 127.2, 40.3, 31.3, 24.3, 16.4.

HRMS (APCI): calculated for $C_{20}H_{25}O_2S^+$ $[M{-}H]^+$: 329.1569; Found 329.1572.

7. Assignment of Alkene Configuration

Figure S2: $^{1}H,^{1}H$–NOESY of Ethyl *(E)*-4-((2-ethyl-3,3-dimethyl-1-phenylbut-1-en-1-yl)thio)benzoate (**3ha**)

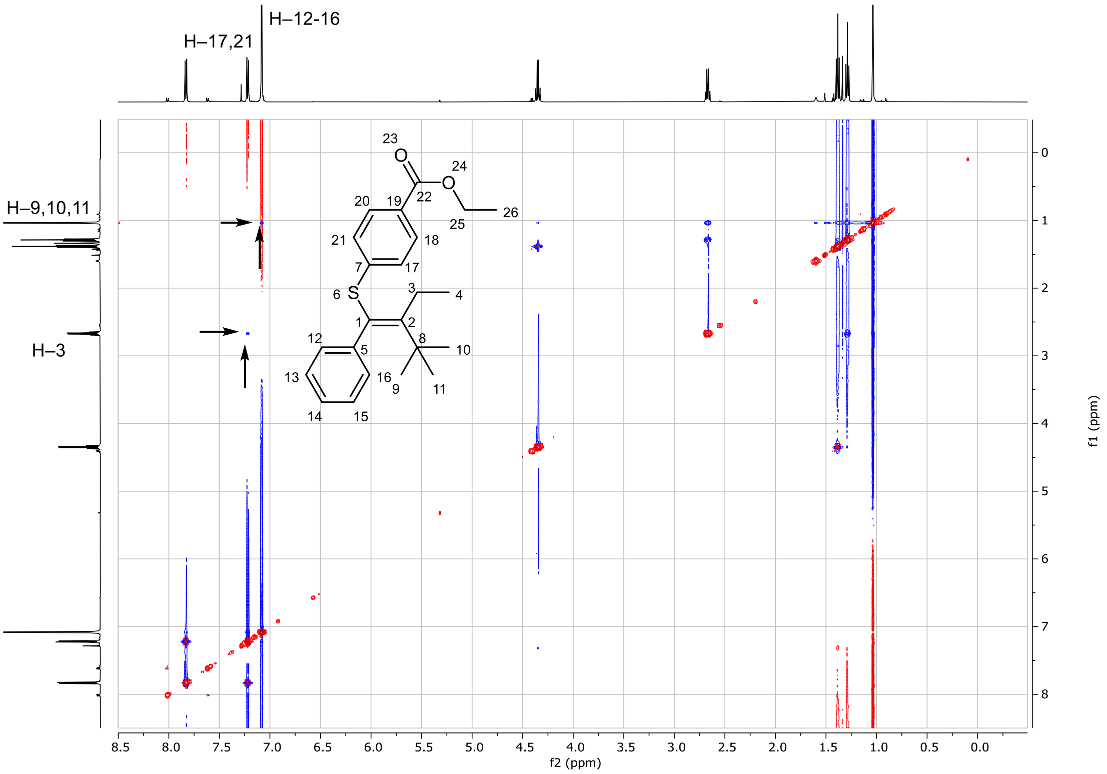

8. NMR Spectra

Figure S3. ^{1}H NMR spectrum (500 MHz, $CDCl_3$, 298 K) of *tert*-Butyl(phenyl)sulfane (**1a**)

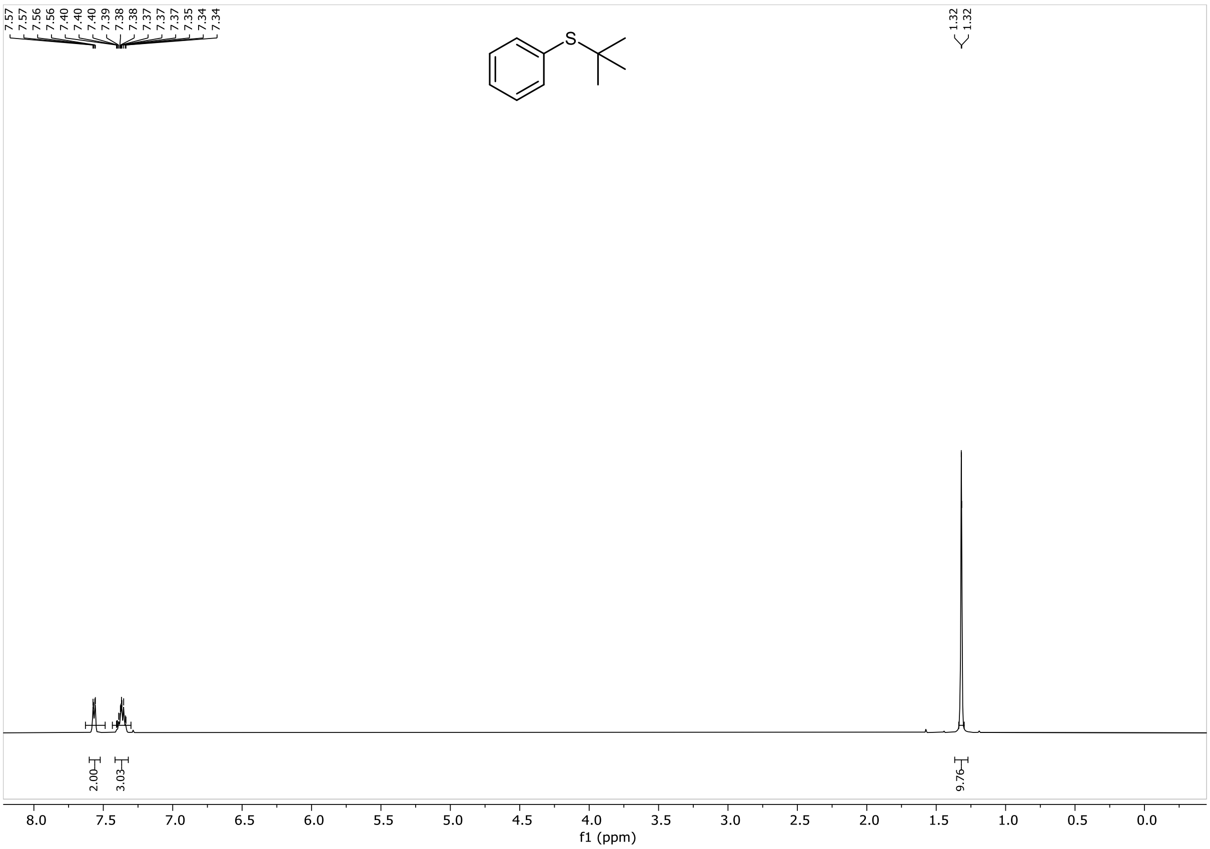

Figure S4. $^{13}C\{^{1}H\}$ NMR spectrum (126 MHz, $CDCl_3$, 298 K) of **1a**

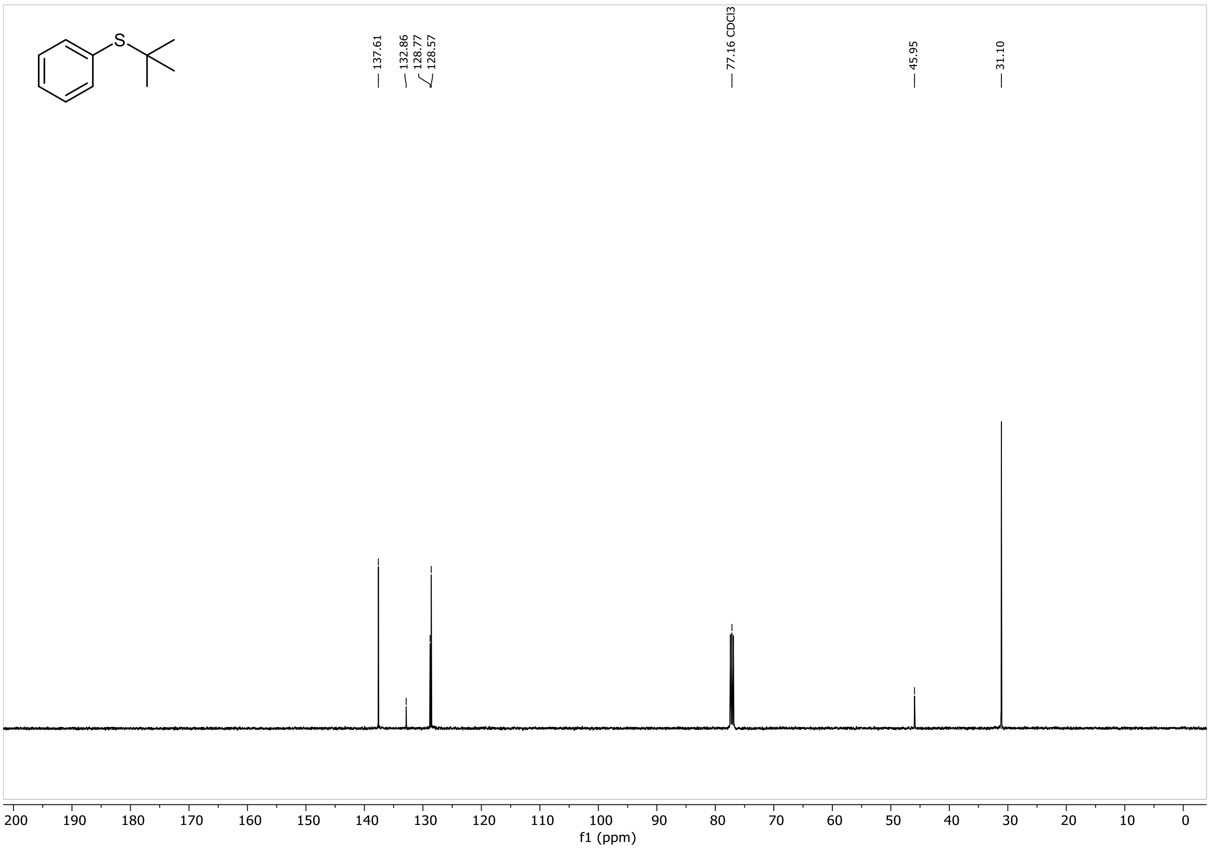

Figure S5. ^{1}H NMR spectrum (500 MHz, $CDCl_3$, 298 K) of *tert*-Butyl(*p*-tolyl)sulfane (**1b**)

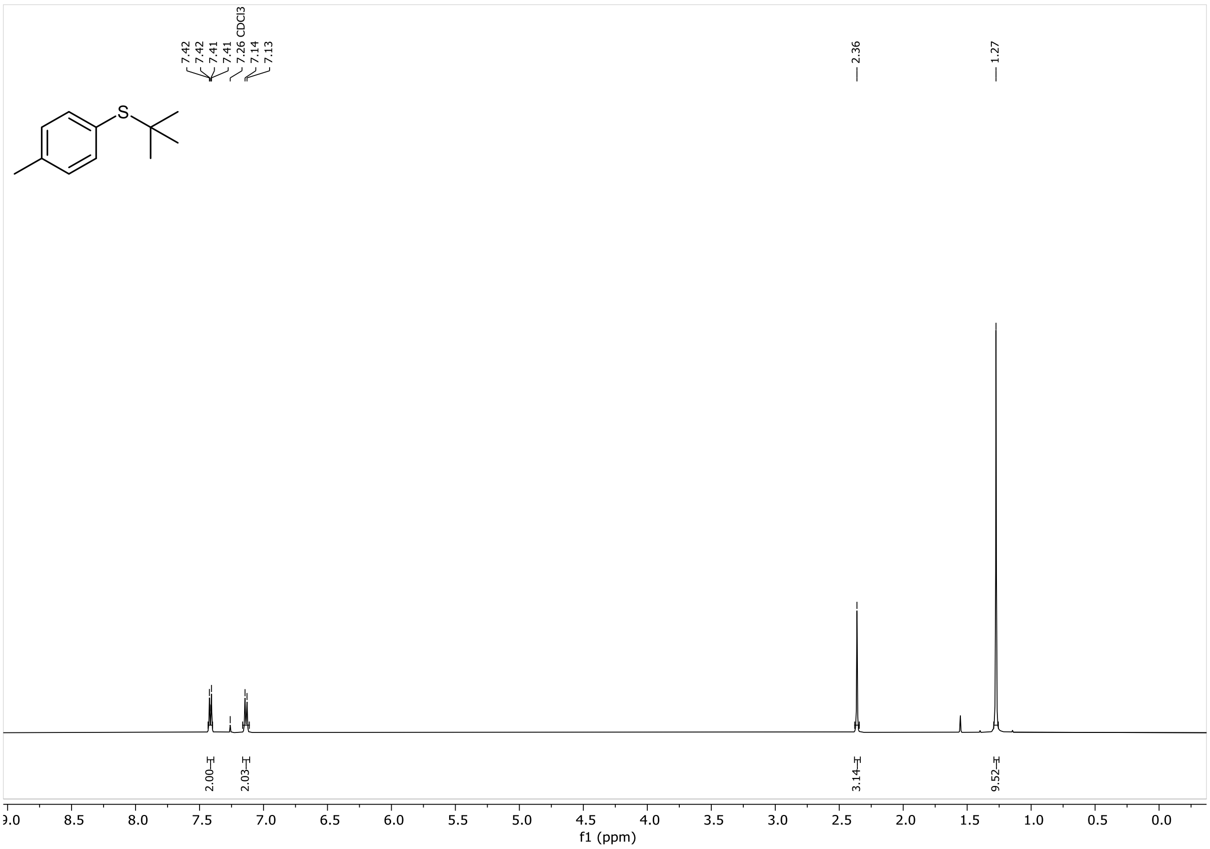

Figure S6. $^{13}C\{^{1}H\}$ NMR spectrum (126 MHz, $CDCl_3$, 298 K) of **1b**

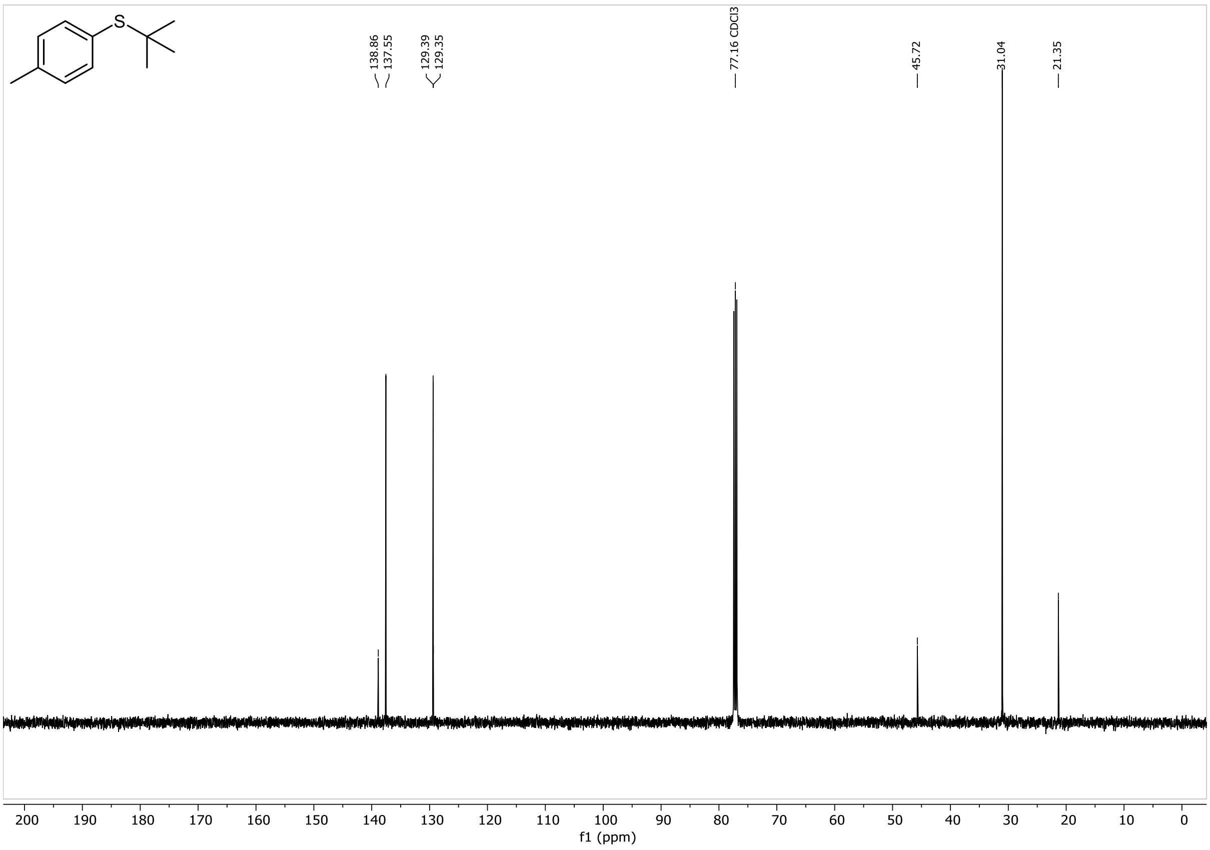

Figure S7. ^{1}H NMR spectrum (500 MHz, $CDCl_3$, 298 K) of *tert*-Butyl(4-fluorophenyl)sulfane (**1c**)

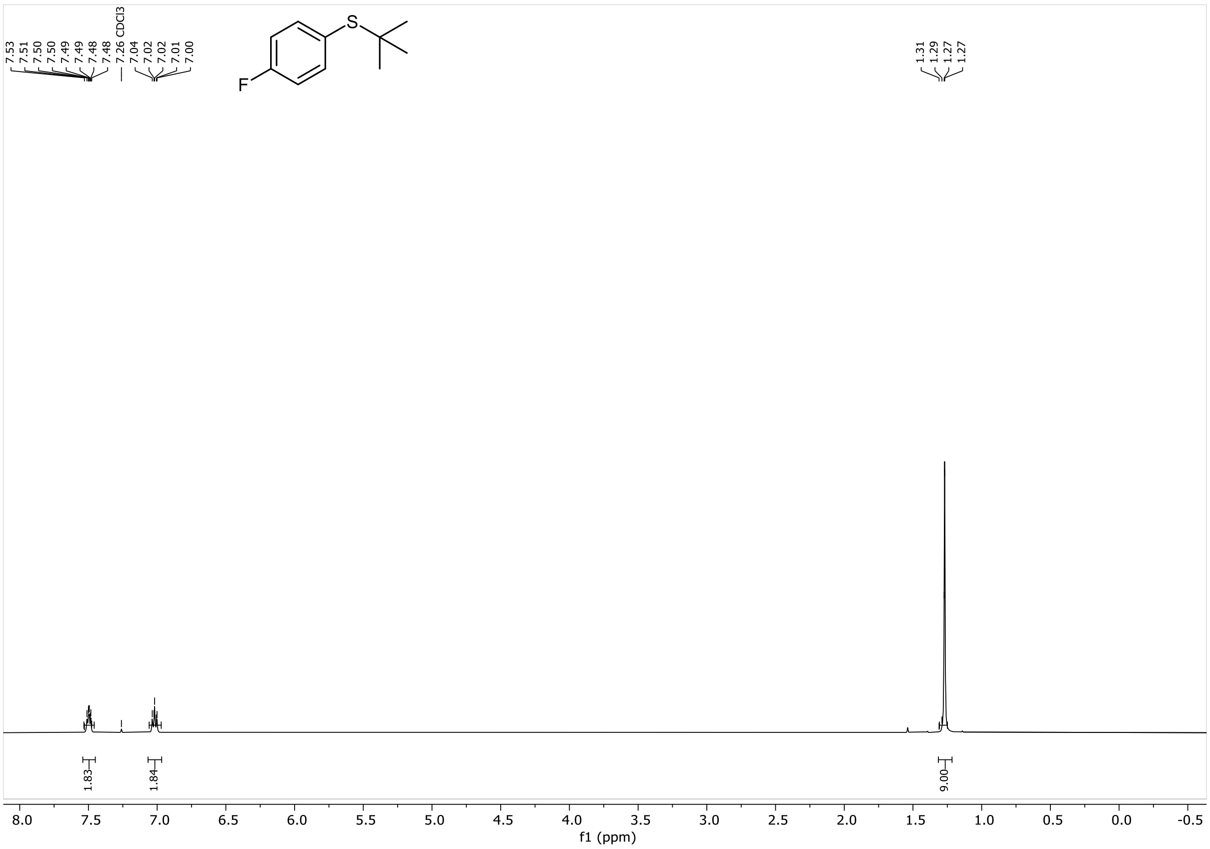

Figure S8. $^{13}C\{^{1}H\}$ NMR spectrum (126 MHz, $CDCl_3$, 298 K) of **1c**

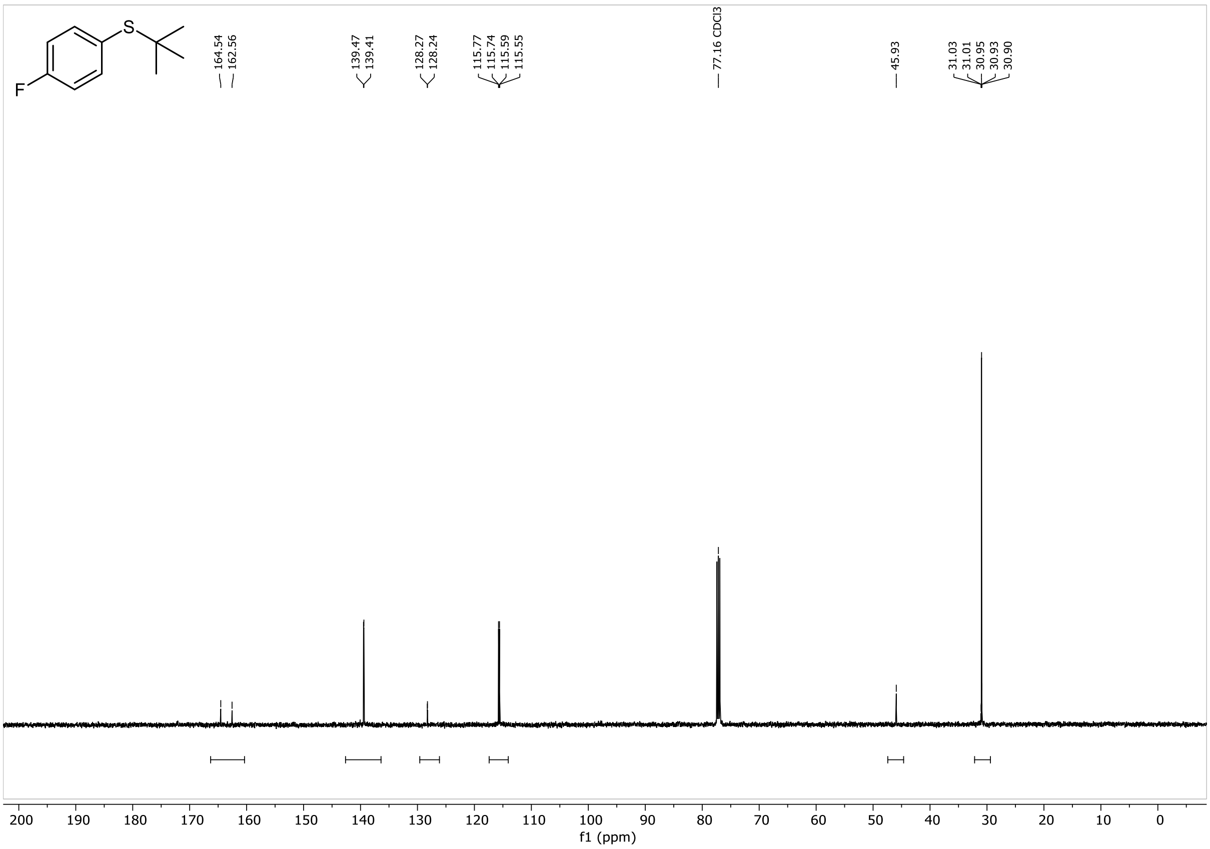

Figure S9. ^{1}H NMR spectrum (500 MHz, $CDCl_3$, 298 K) of *tert*-Butyl(4-chlorophenyl)sulfane (**1d**)

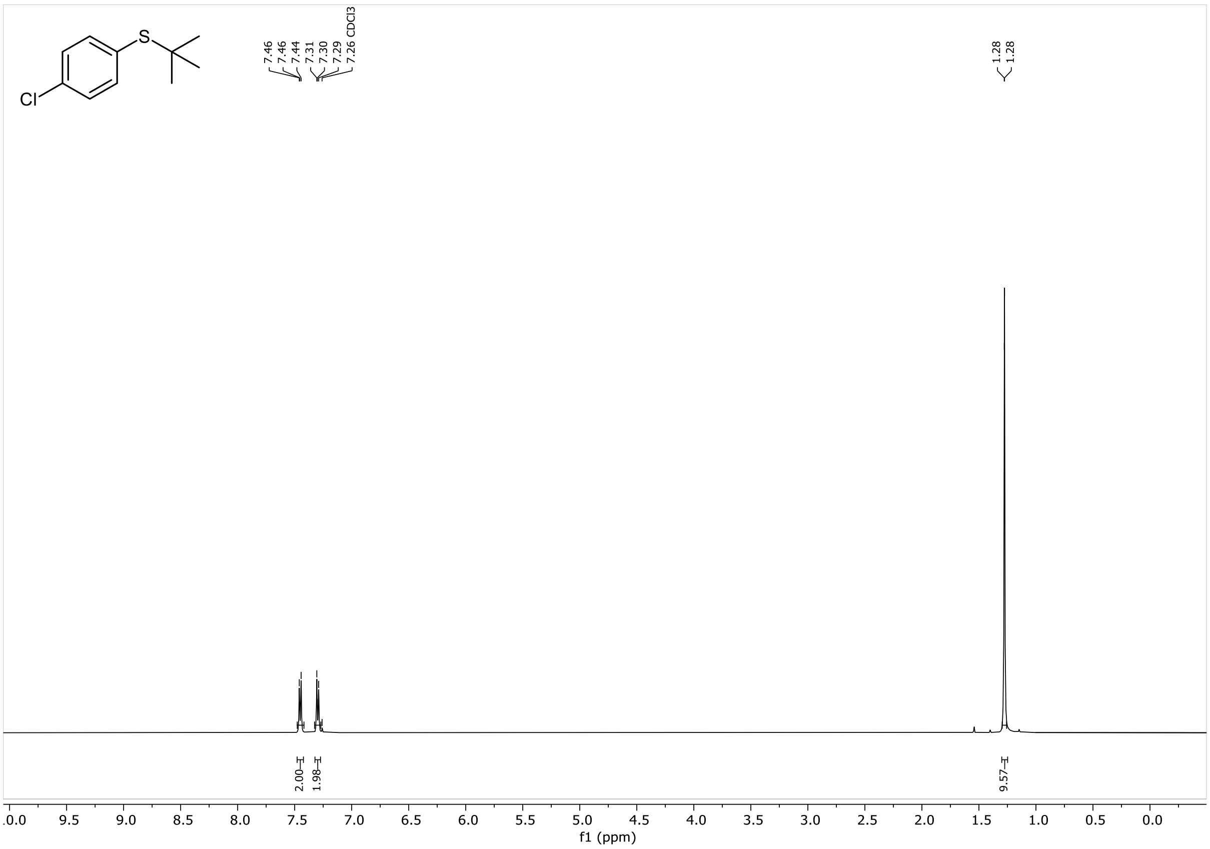

Figure S10. $^{13}C\{^1H\}$ NMR spectrum (126 MHz, $CDCl_3$, 298 K) of **1d**

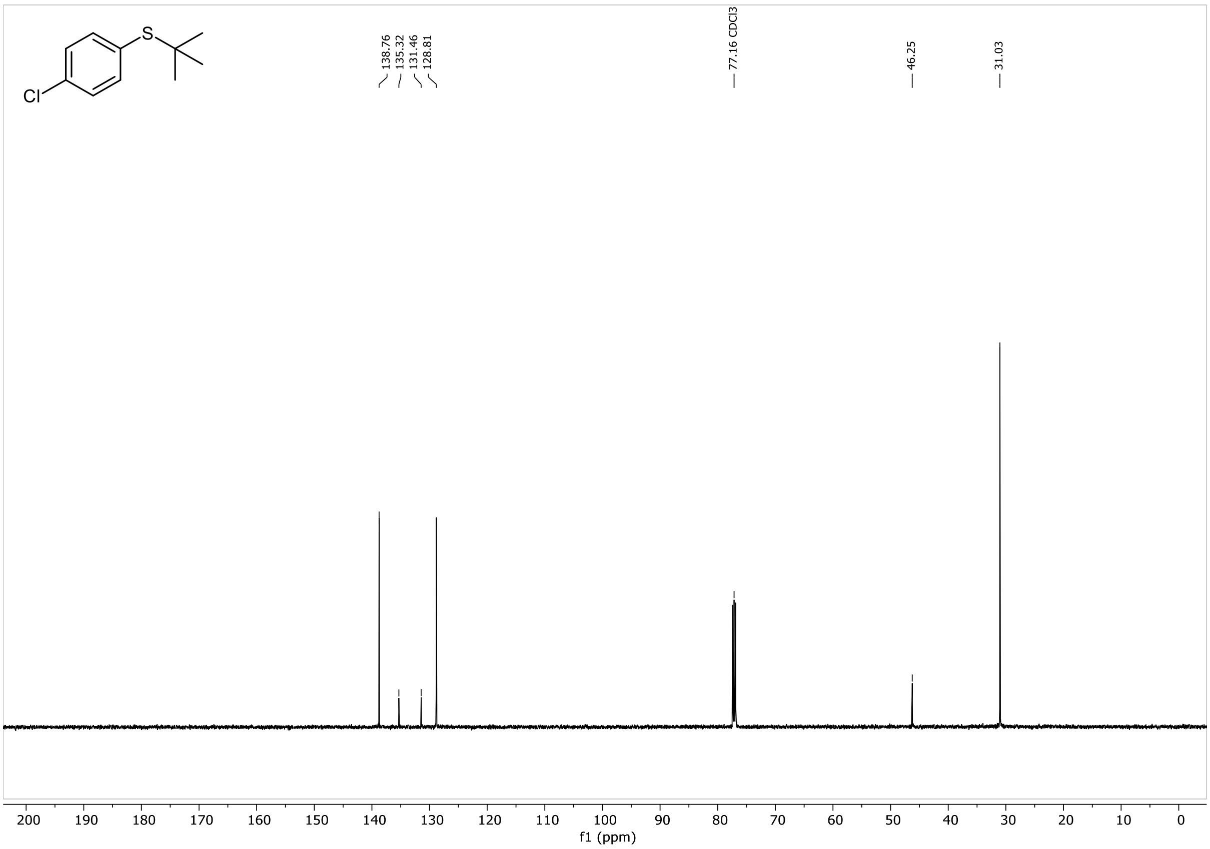

Figure S11. ^{1}H NMR spectrum (500 MHz, $CDCl_3$, 298 K) of *tert*-Butyl(4-bromophenyl)sulfane (**1e**)

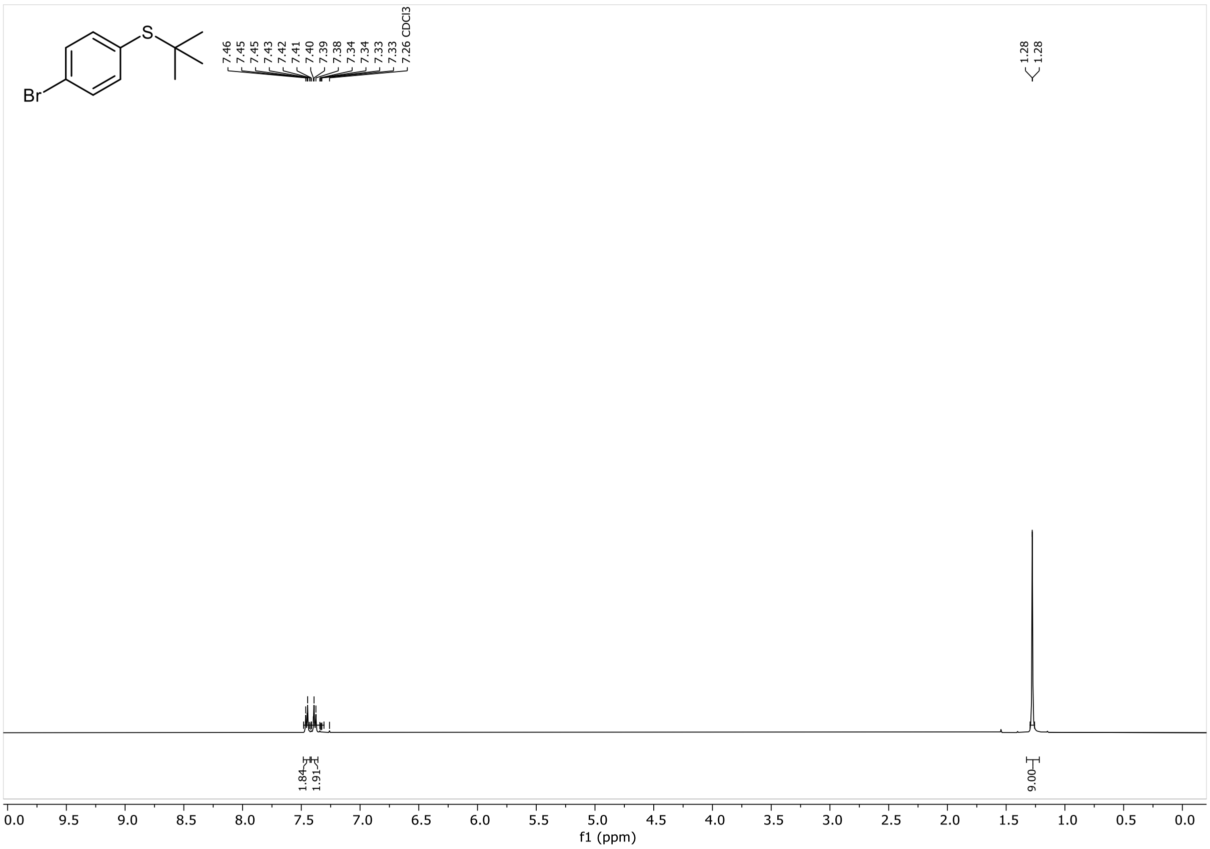

Figure S12. $^{13}C\{^1H\}$ NMR spectrum (126 MHz, $CDCl_3$, 298 K) of **1e**

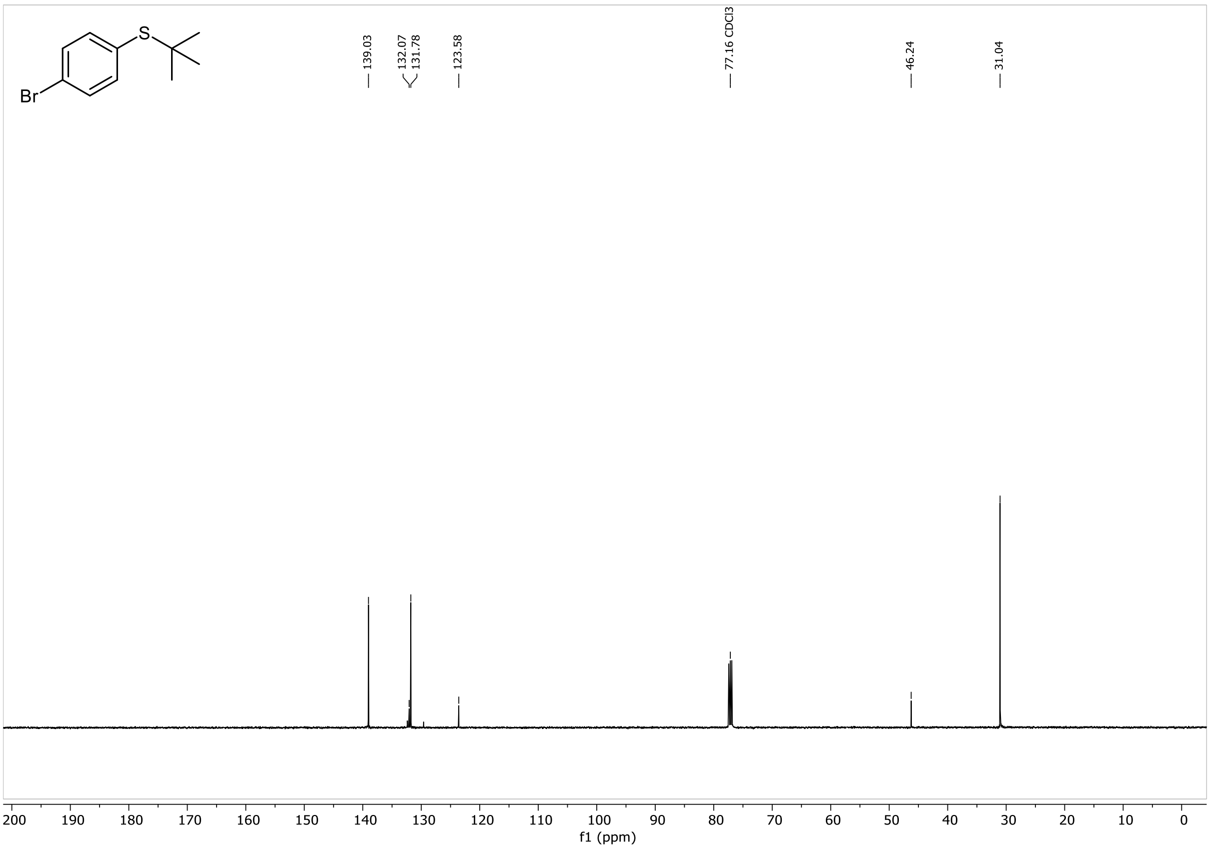

Figure S13. ^{1}H NMR spectrum (500 MHz, $CDCl_3$, 298 K) of *tert*-Butyl(4-(*tert*-butyl)phenyl)sulfane (**1f**)

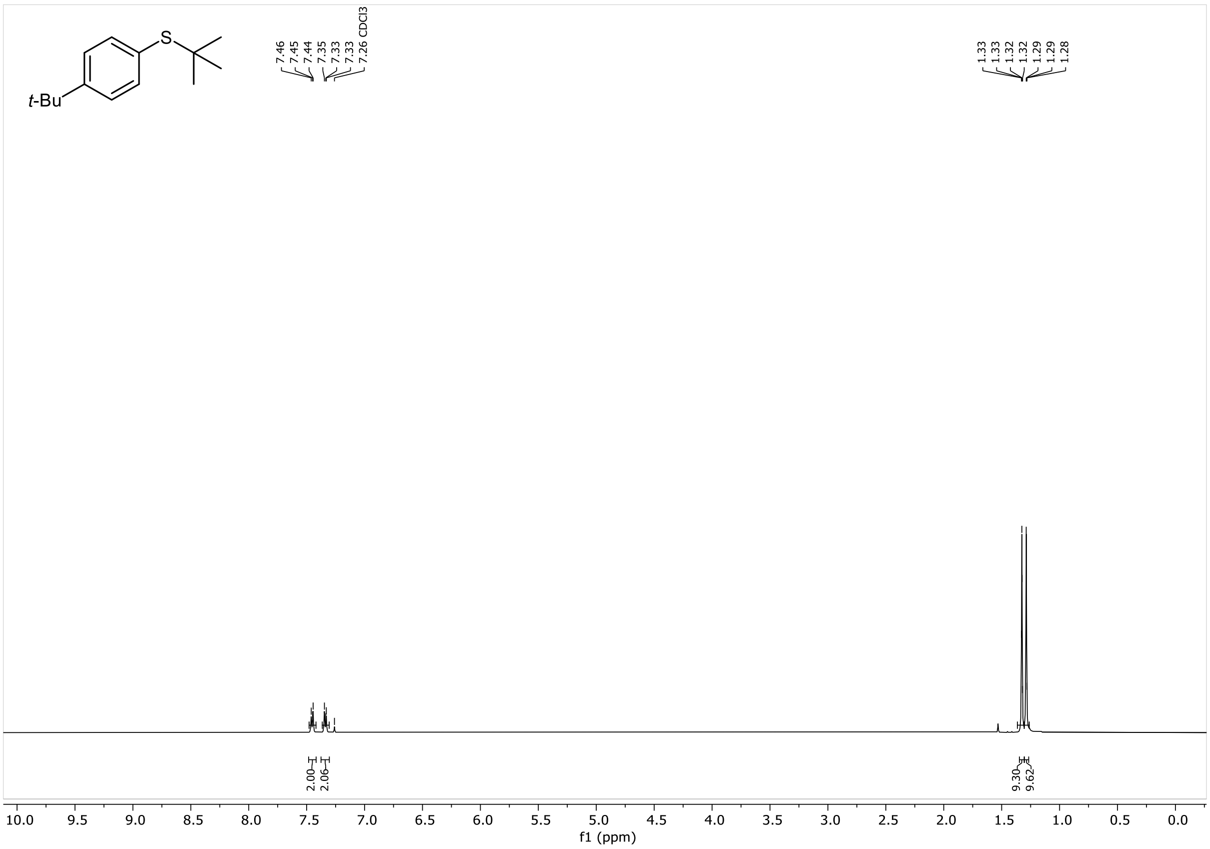

Figure S14. $^{13}C\{^{1}H\}$ NMR spectrum (126 MHz, $CDCl_3$, 298 K) of **1f**

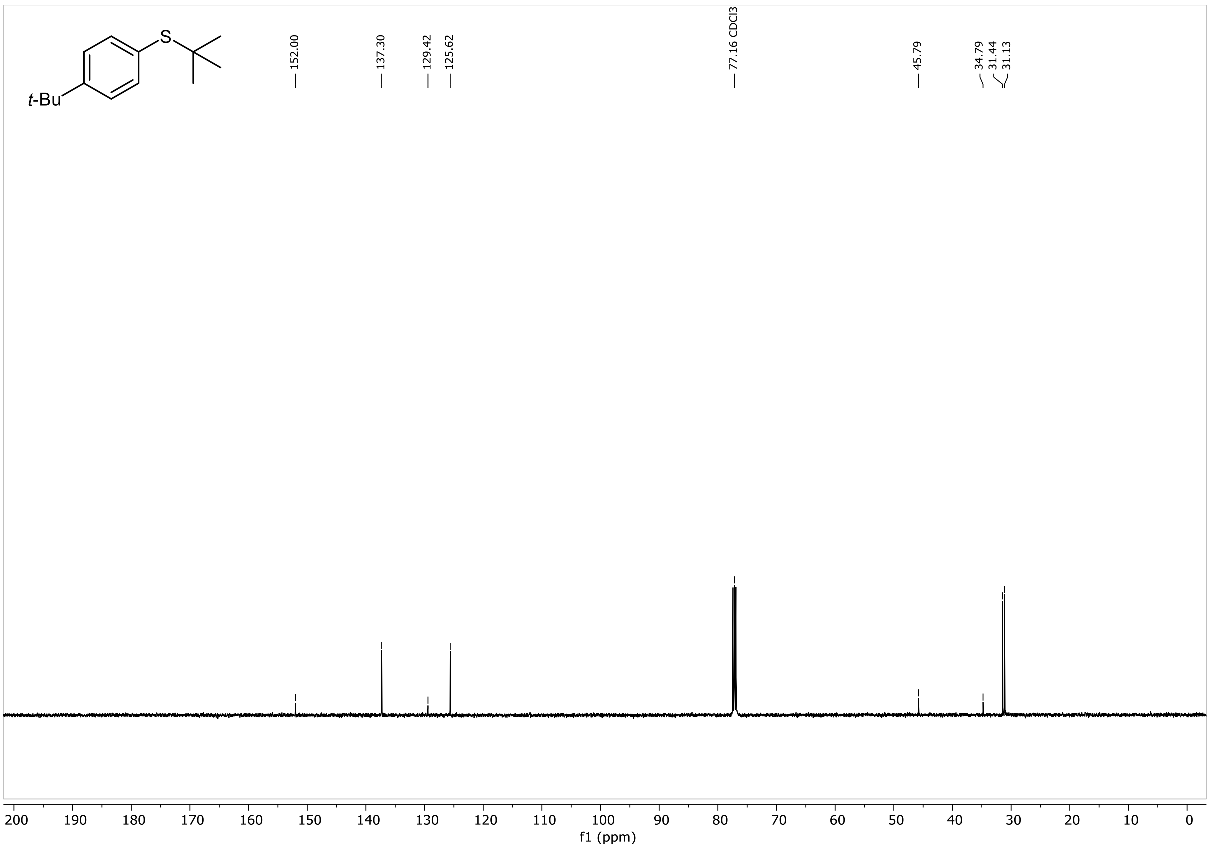

Figure S15. ^{1}H NMR spectrum (500 MHz, $CDCl_3$, 298 K) of methyl-4-(*tert*-butylthio)benzoate (**1g**)

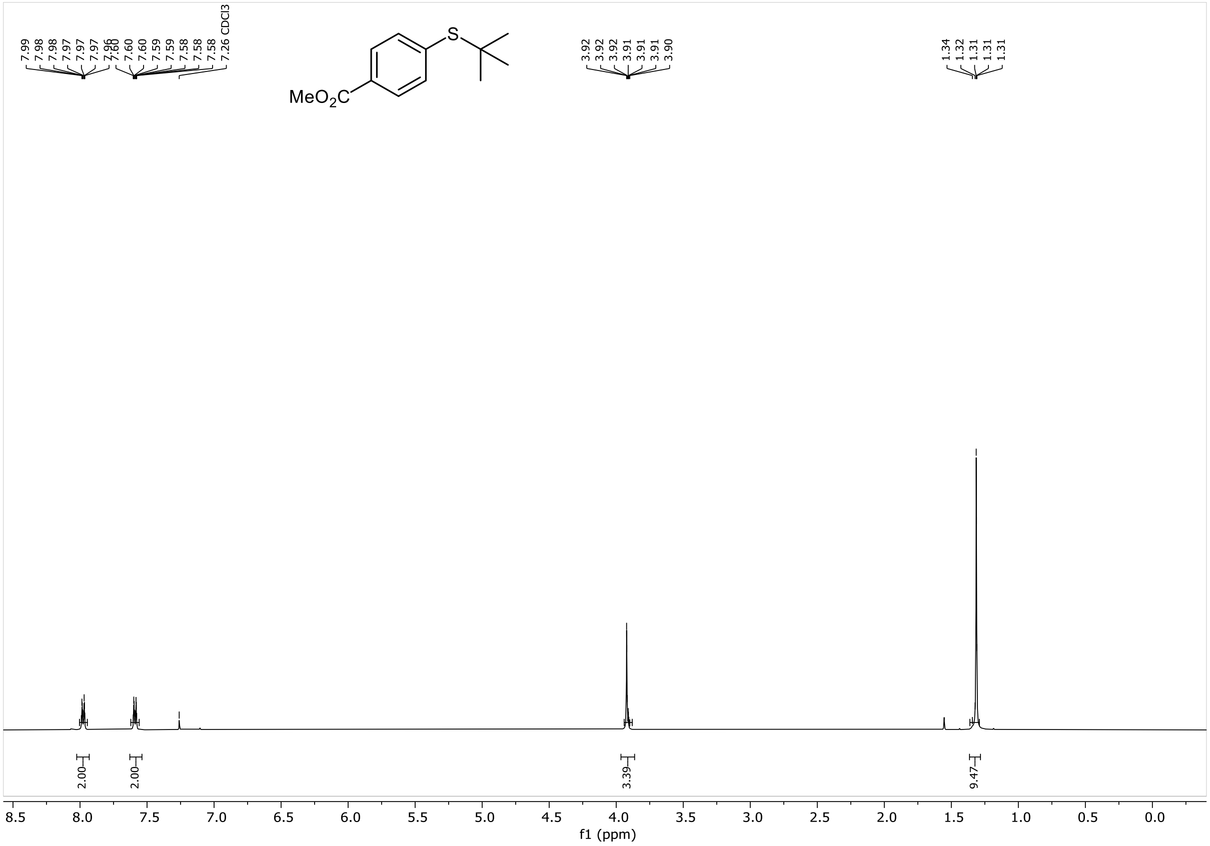

Figure S16. $^{13}C\{^{1}H\}$ NMR spectrum (126 MHz, $CDCl_3$, 298 K) of **1g**

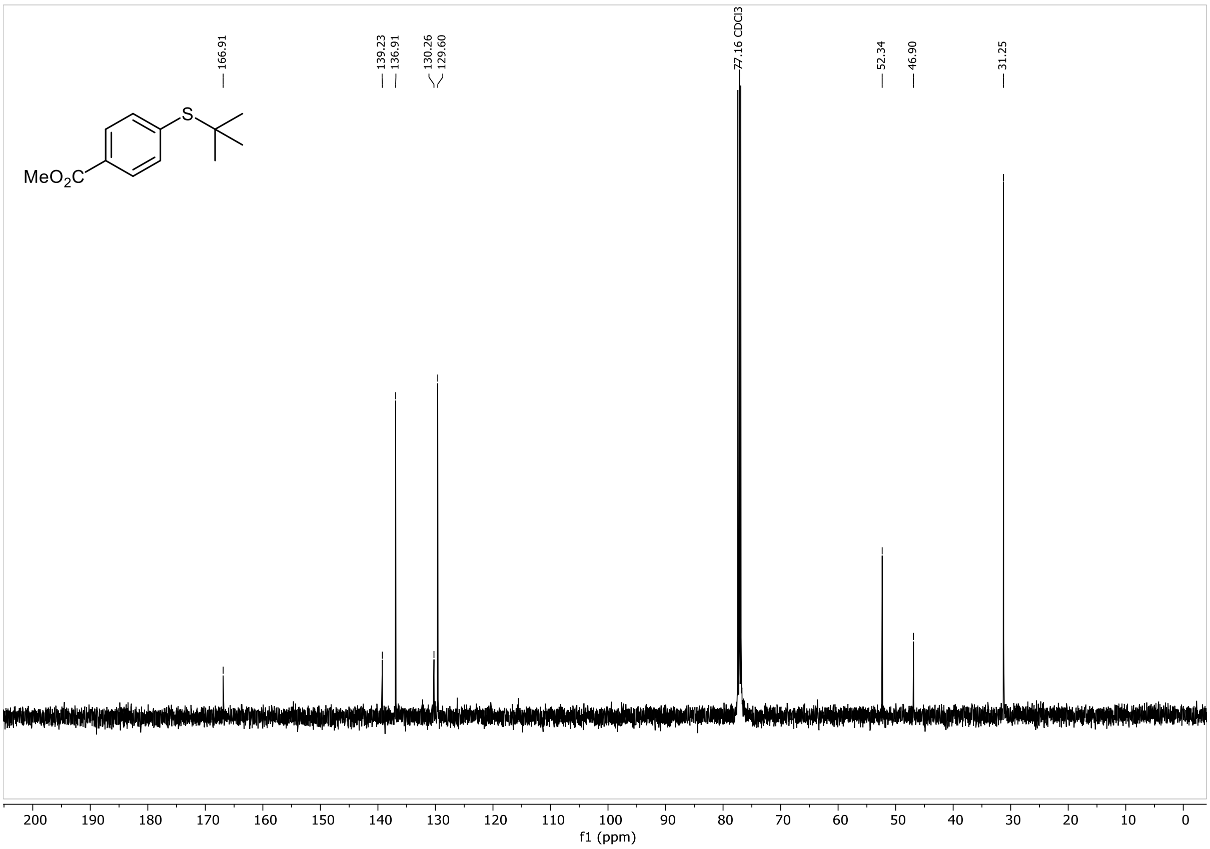

Figure S17. ^1H NMR spectrum (500 MHz, $CDCl_3$, 298 K) of ethyl-4-(*tert*-butylthio)benzoate (**1h**)

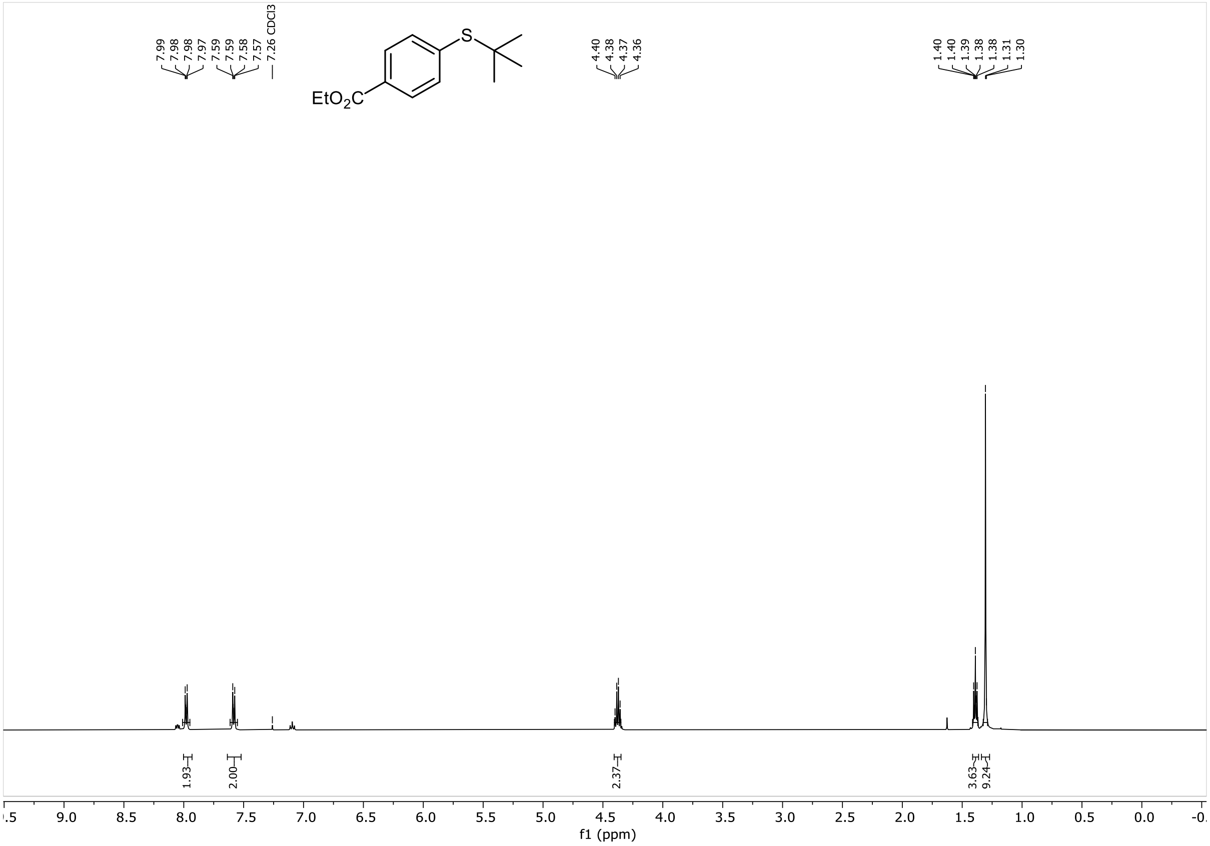

Figure S18. $^{13}C\{^1H\}$ NMR spectrum (126 MHz, $CDCl_3$, 298 K) of **1h**

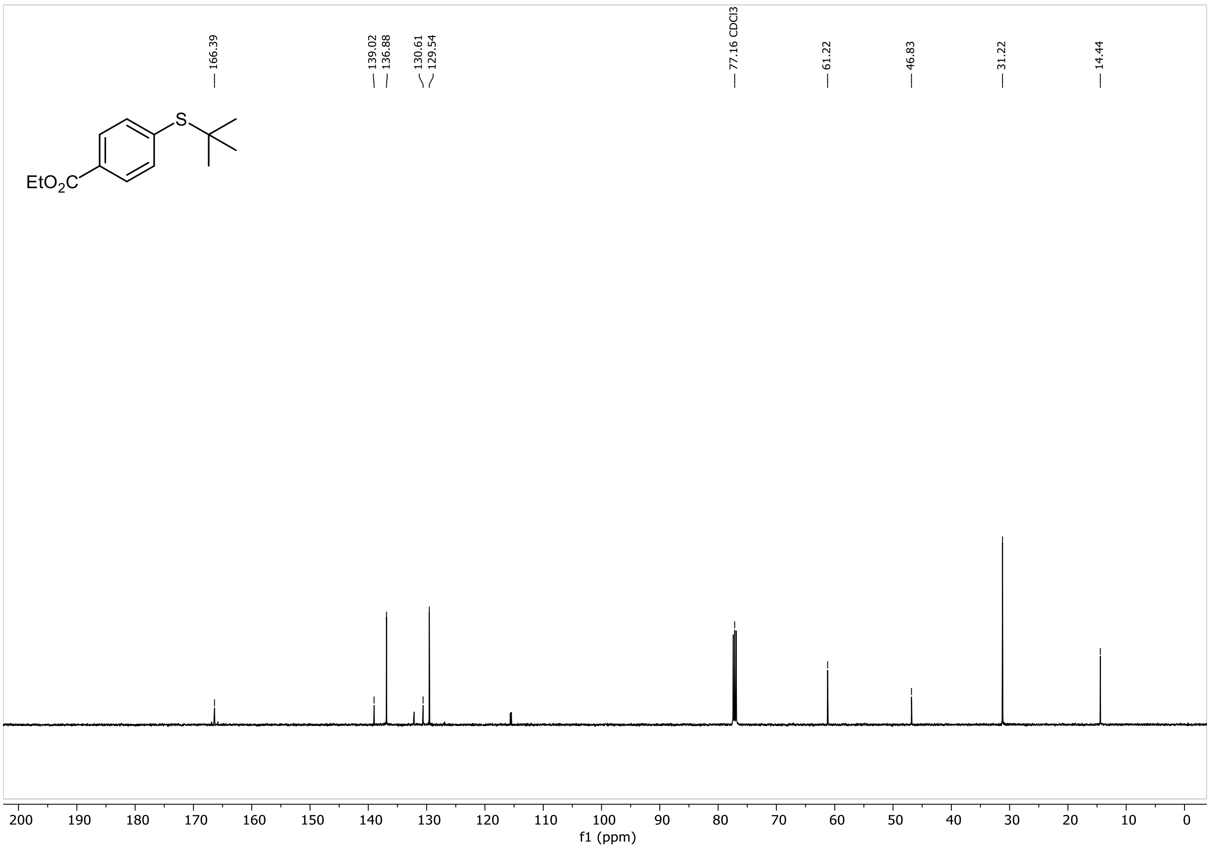

Figure S19. ^{1}H NMR spectrum (500 MHz, $CDCl_3$, 298 K) of *tert*-Butyl(naphthalen-2-yl)sulfane (**1i**)

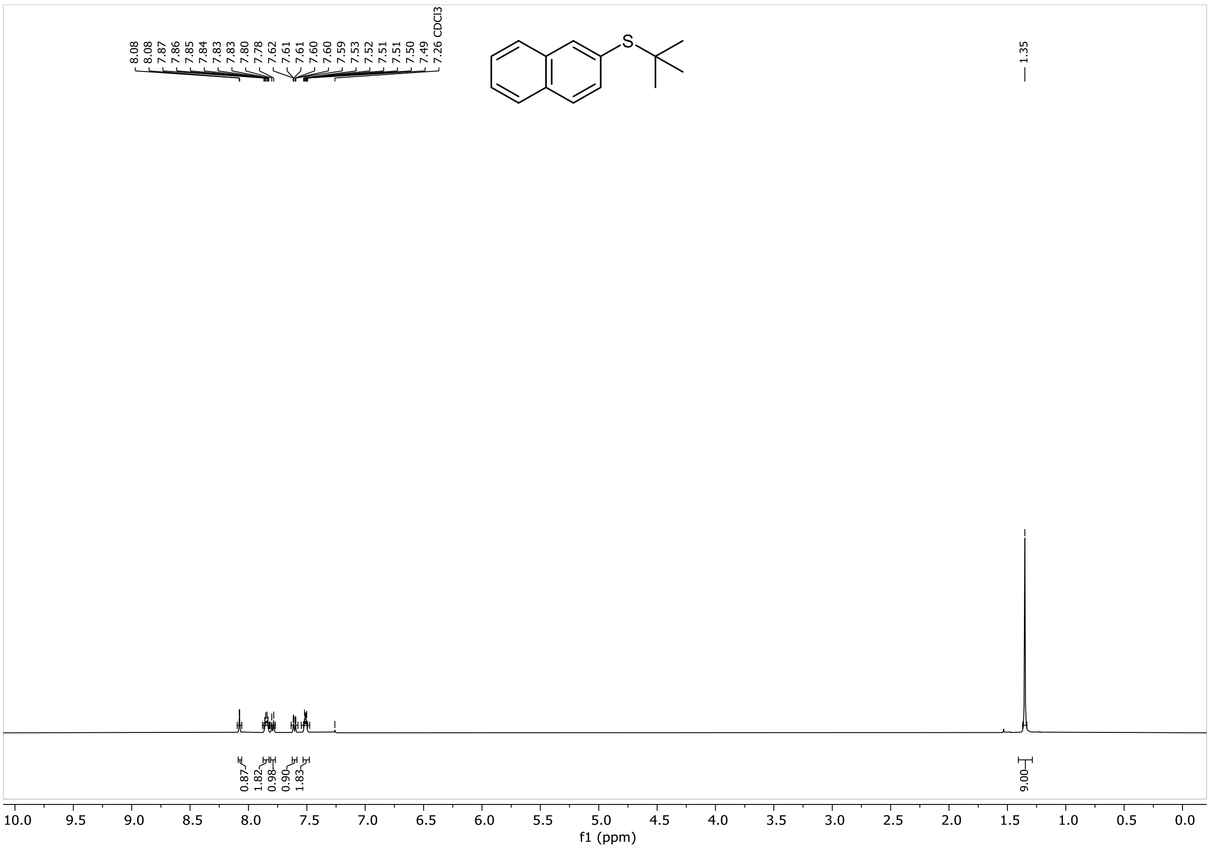

Figure S20. $^{13}C\{^{1}H\}$ NMR spectrum (126 MHz, $CDCl_3$, 298 K) of **1i**

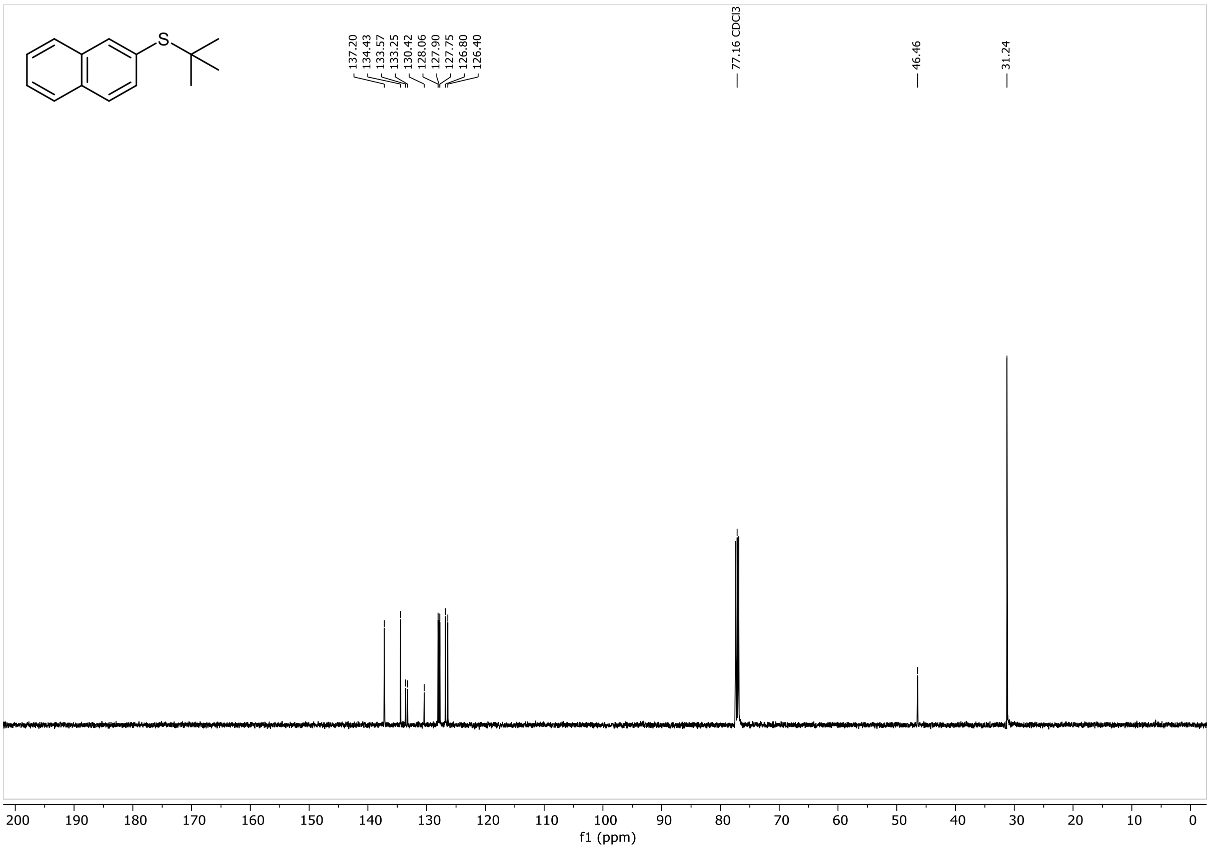

Figure S21. ^{1}H NMR spectrum (500 MHz, $CDCl_3$, 298 K) of *tert*-Butyl(*m*-tolyl)sulfane (**1j**)

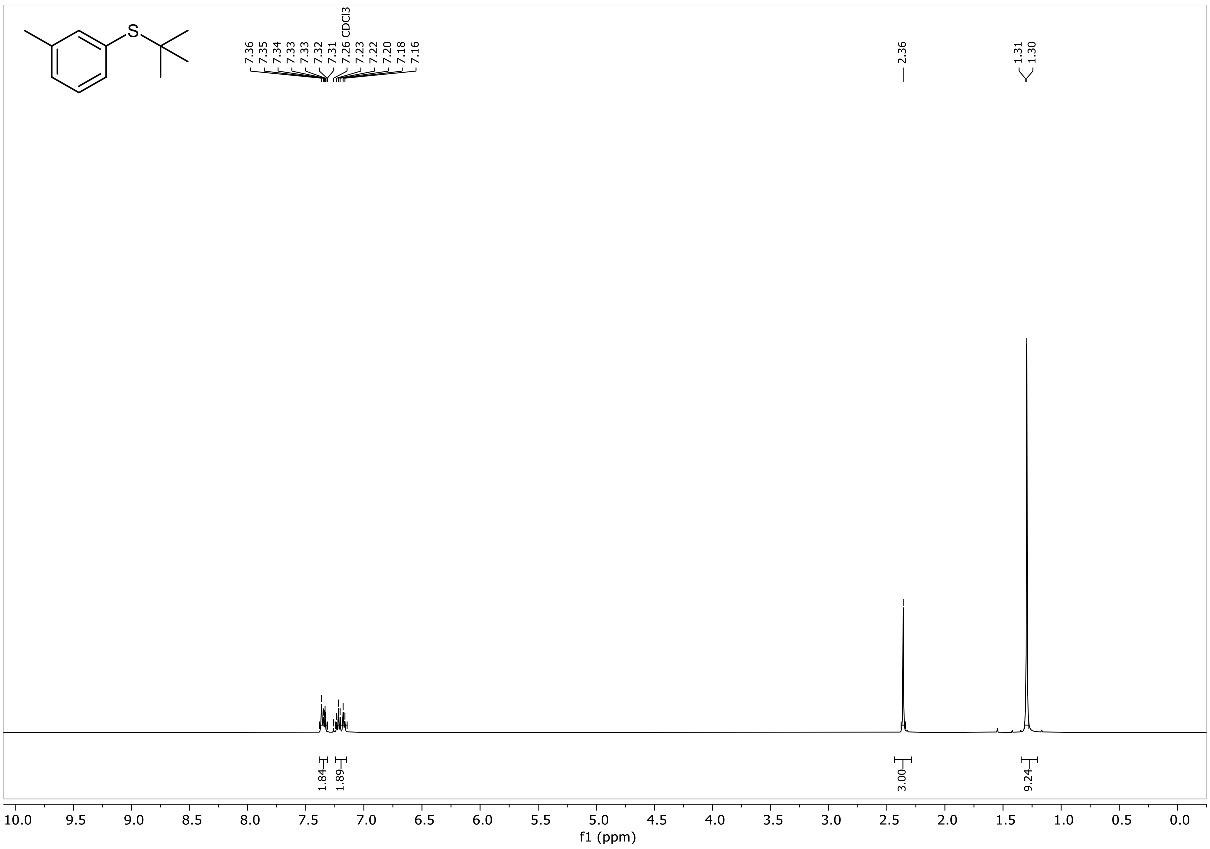

Figure S22. ^{13}C{^{1}H} NMR spectrum (126 MHz, $CDCl_3$, 298 K) of **1j**

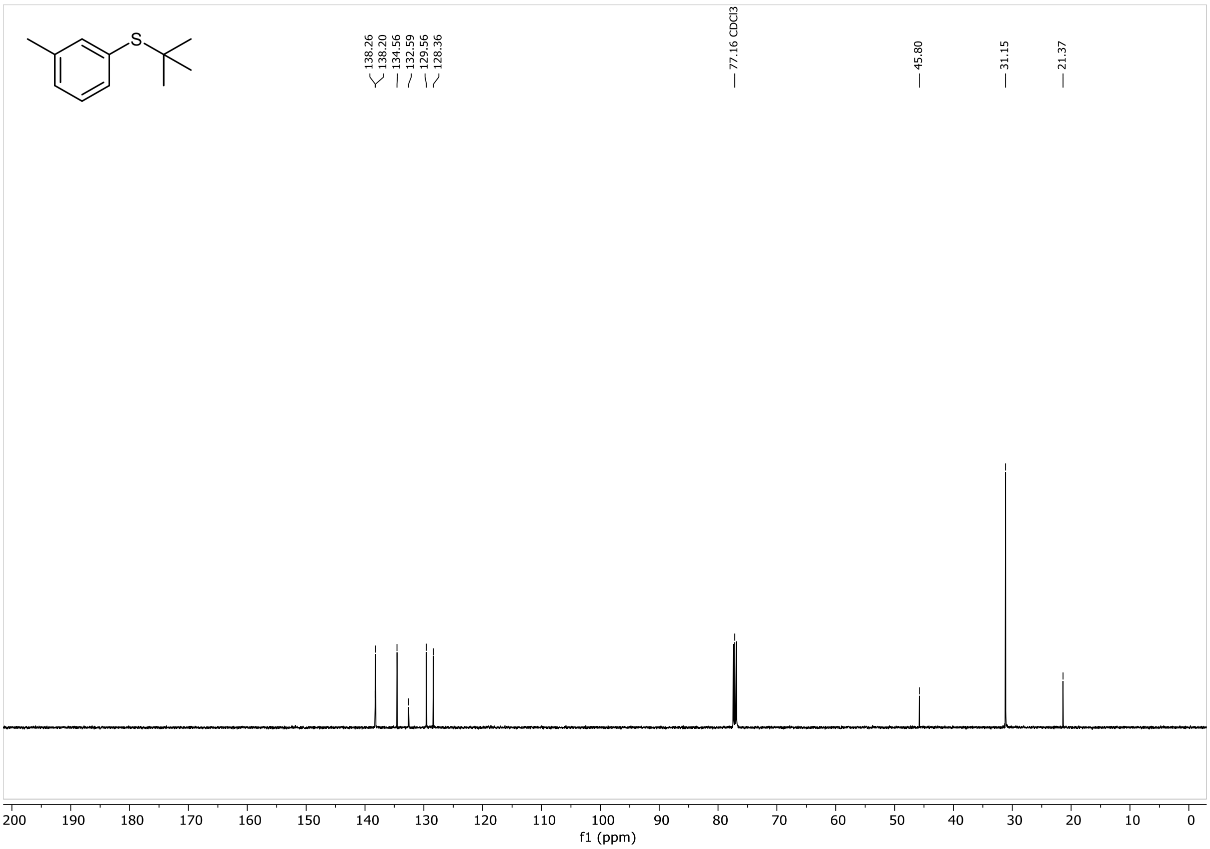

Figure S23. ^{1}H NMR spectrum (500 MHz, $CDCl_3$, 298 K) of *tert*-Butyl(*o*-tolyl)sulfane (**1k**)

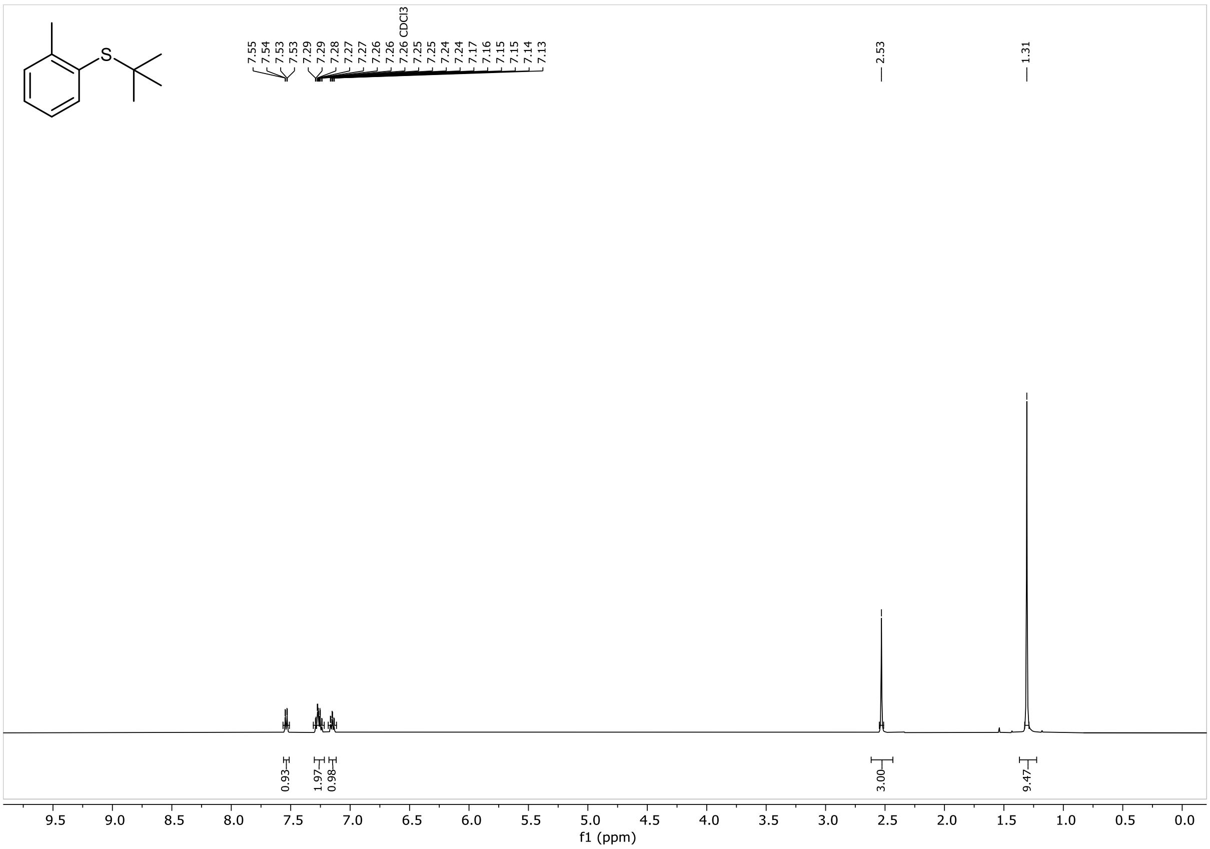

Figure S24. $^{13}C\{^{1}H\}$ NMR spectrum (126 MHz, $CDCl_3$, 298 K) of **1k**

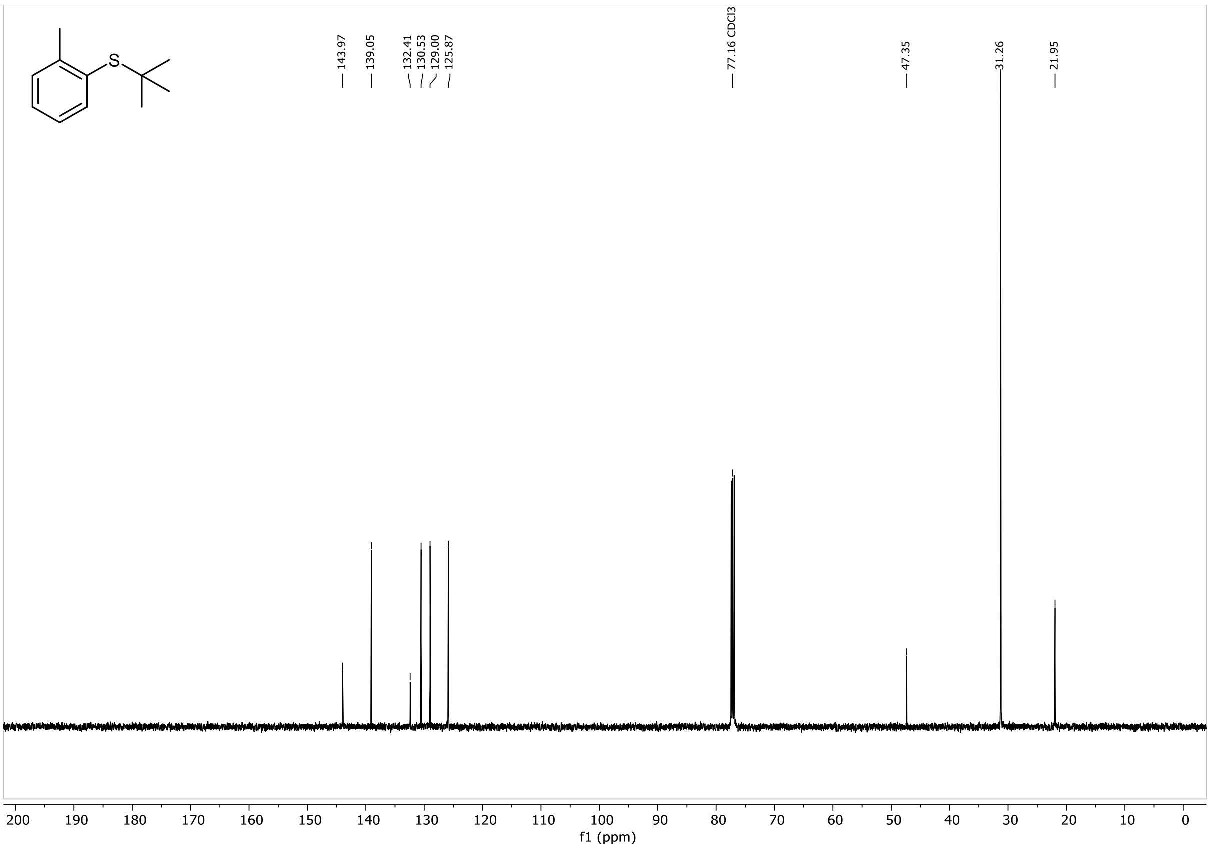

Figure S25. ^{1}H NMR spectrum (500 MHz, $CDCl_3$, 298 K) of *tert*-pentyl(phenyl)sulfane (**1l**)

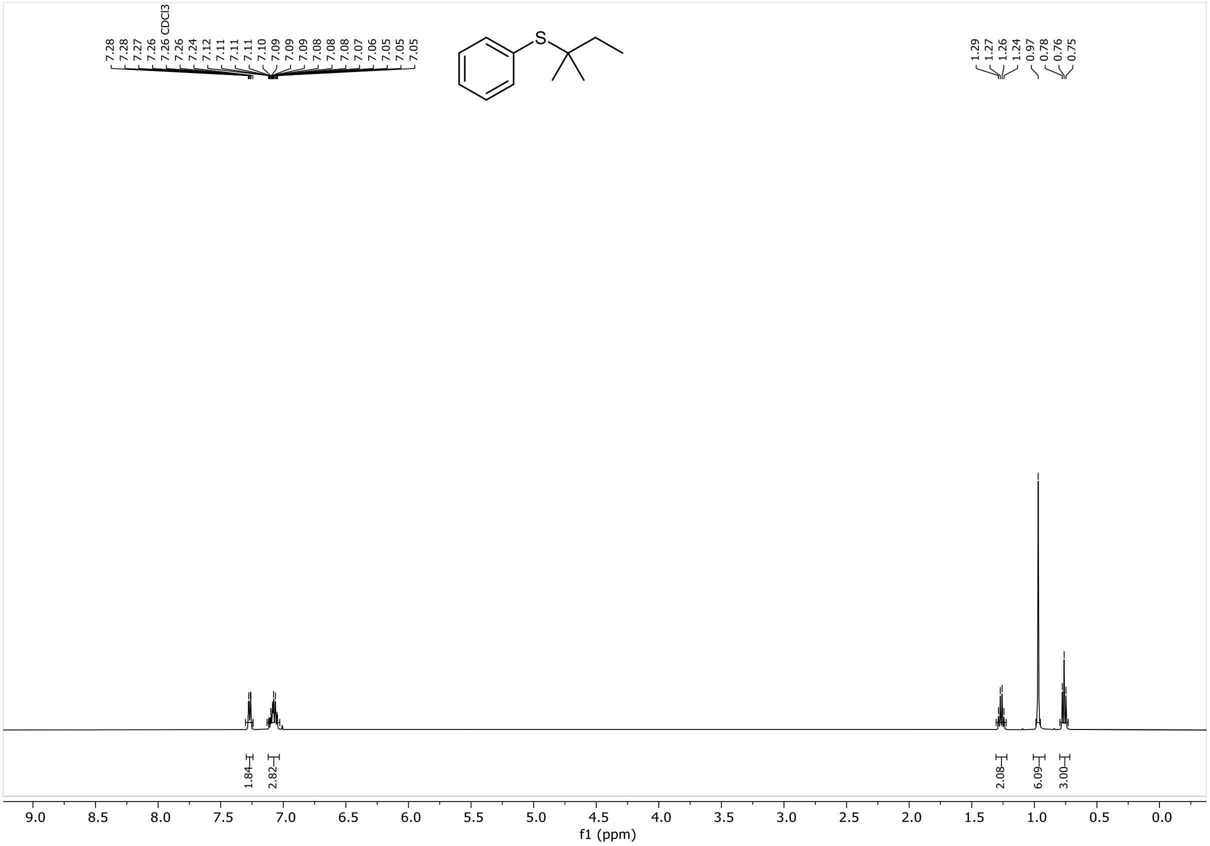

Figure S26. $^{13}C\{^1H\}$ NMR spectrum (126 MHz, $CDCl_3$, 298 K) of **1l**

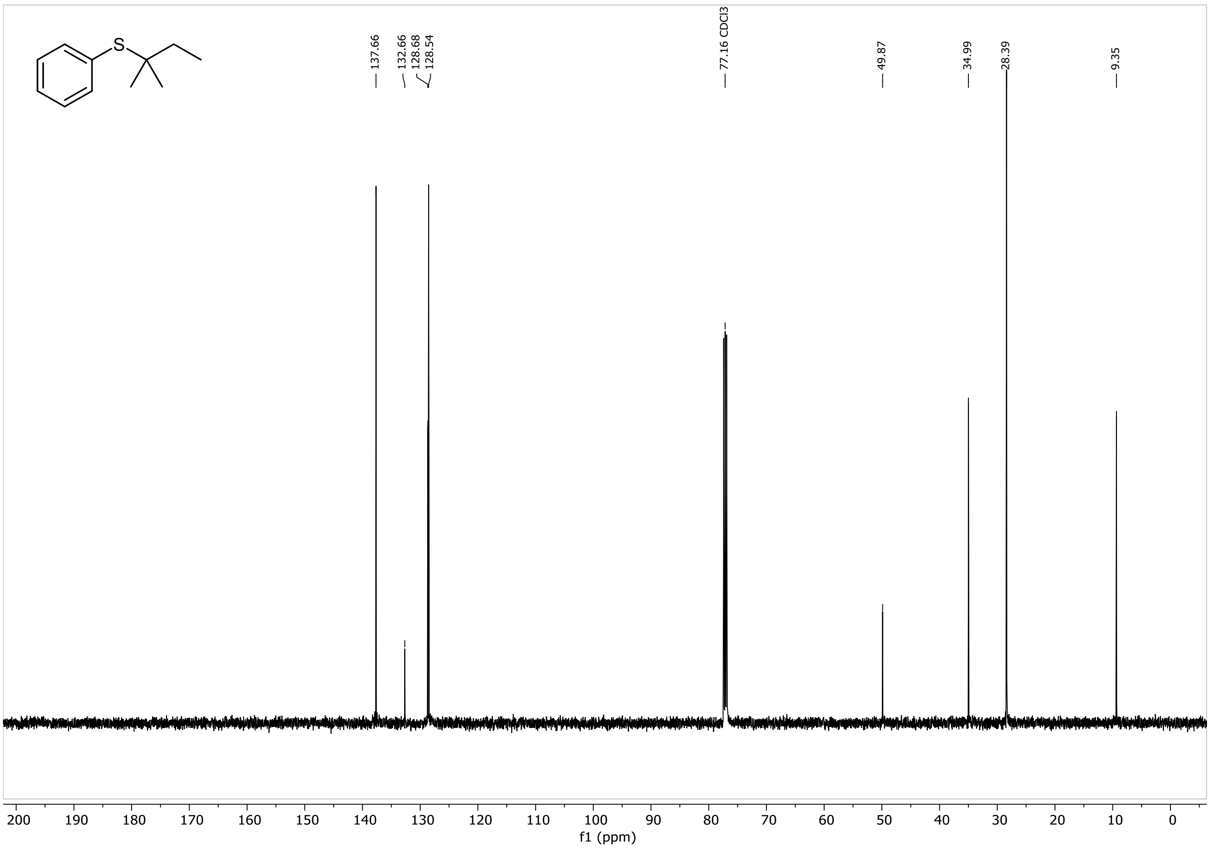

Figure S27. ^{1}H NMR spectrum (500 MHz, $CDCl_3$, 298 K) of (1-methylcyclohexyl)(phenyl)sulfane (**1m**)

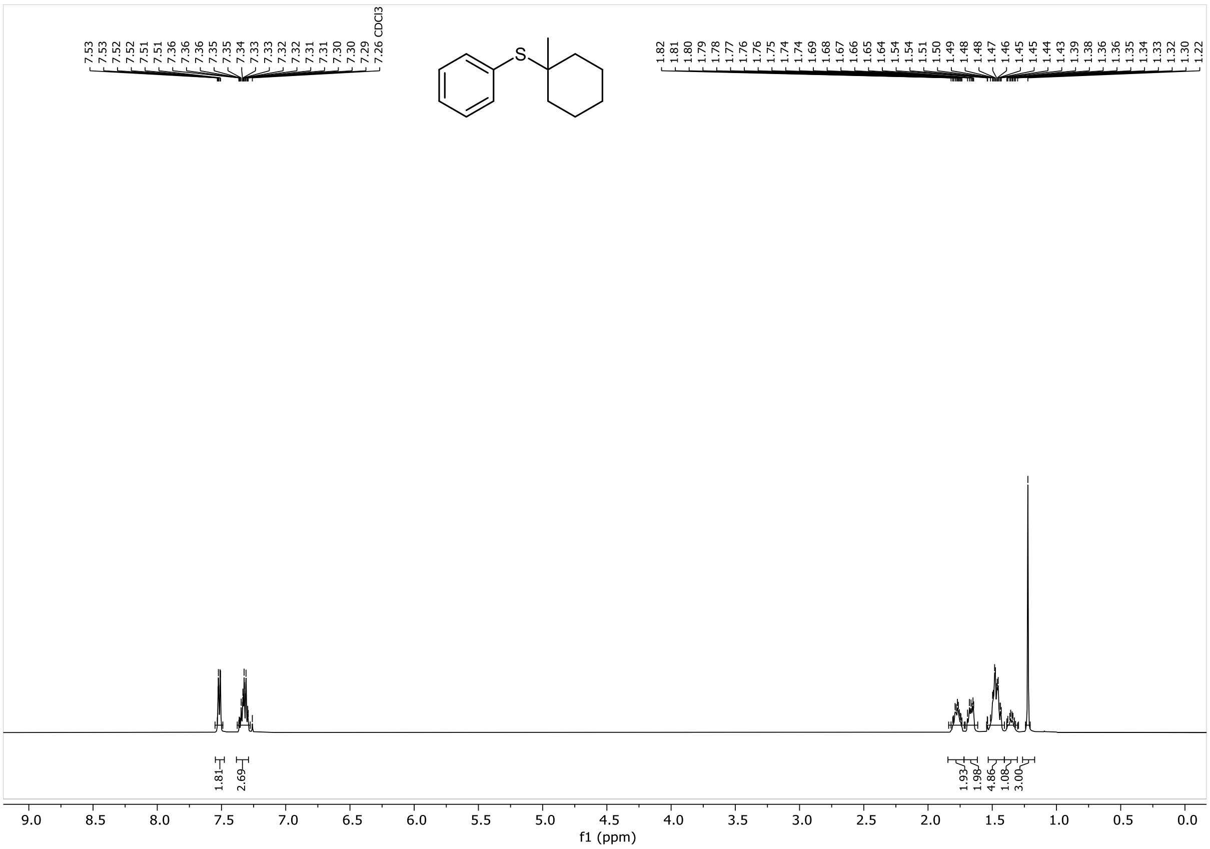

Figure S28. $^{13}C\{^1H\}$ NMR spectrum (126 MHz, $CDCl_3$, 298 K) of **1m**

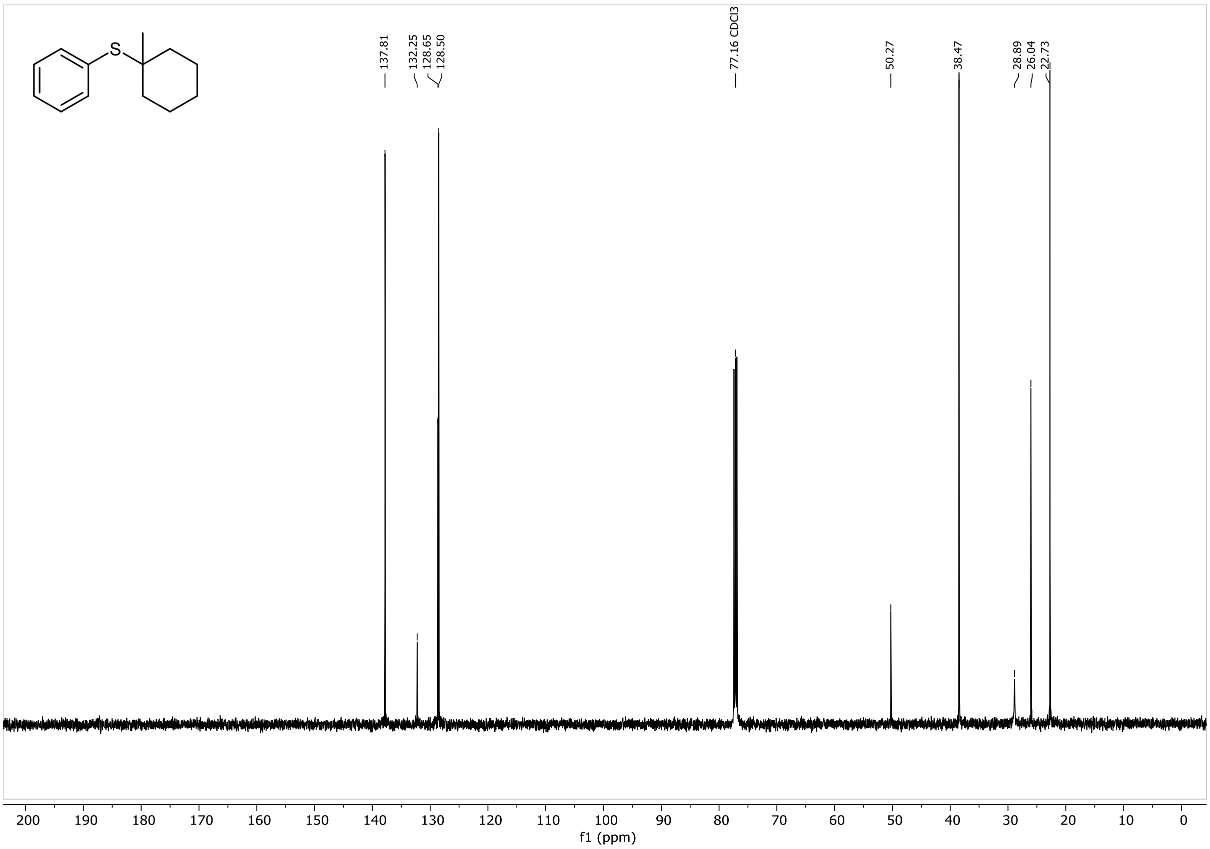

Figure S29. ^{1}H NMR spectrum (500 MHz, $CDCl_3$, 298 K) of adamantan-1-yl(phenyl)sulfane (**1n**)

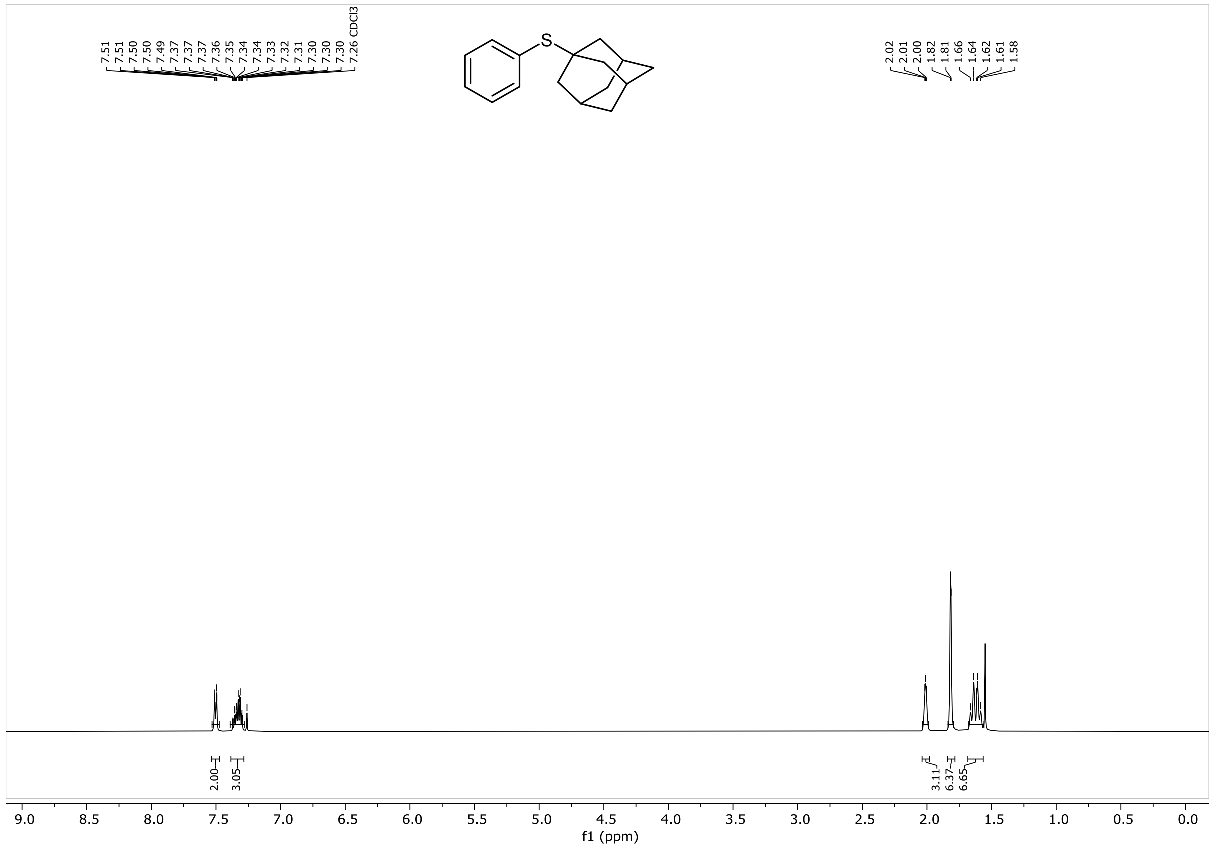

Figure S30. $^{13}C\{^{1}H\}$ NMR spectrum (126 MHz, $CDCl_3$, 298 K) of **1n**

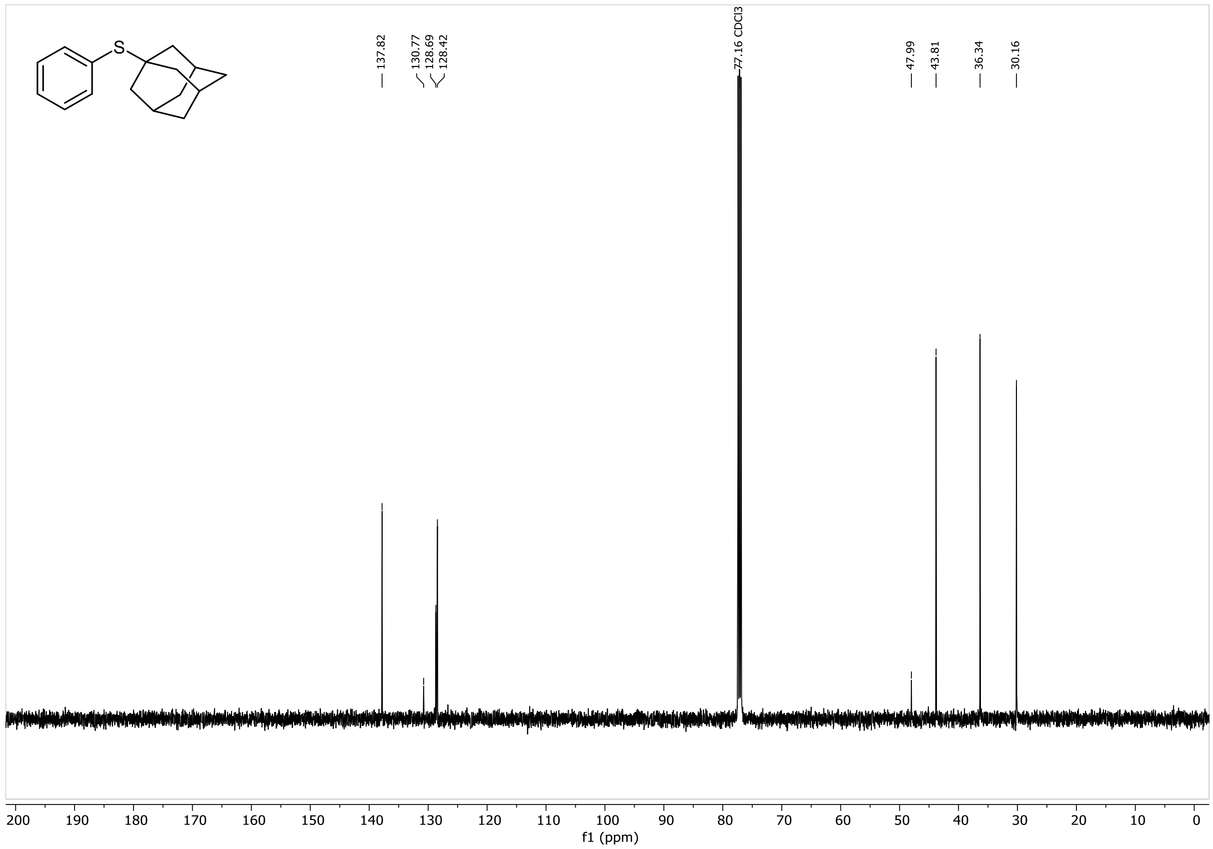

Figure S31. ^{1}H NMR spectrum (500 MHz, $CDCl_3$, 298 K) of phenyl(2-phenylpropan-2-yl)sulfane (**1o**)

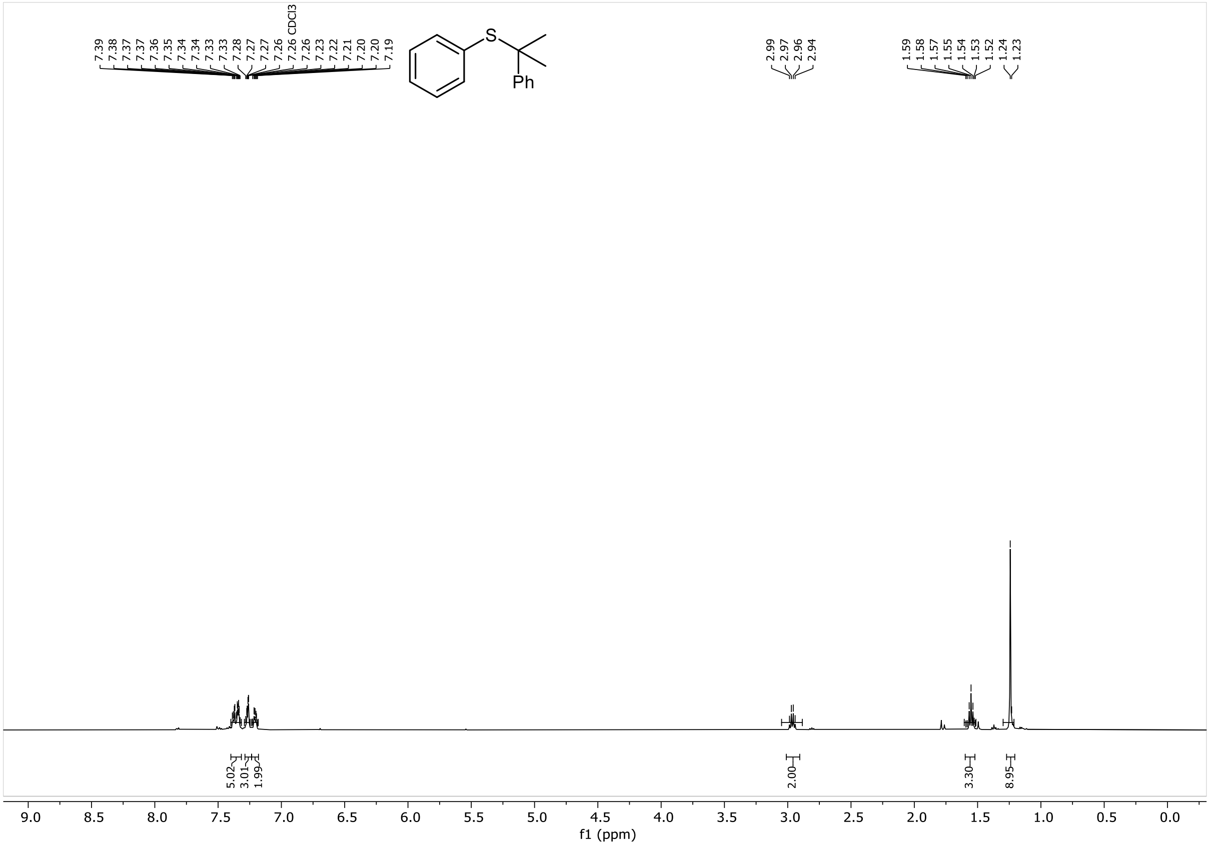

Figure S32. $^{13}C\{^{1}H\}$ NMR spectrum (126 MHz, $CDCl_3$, 298 K) of **1o**

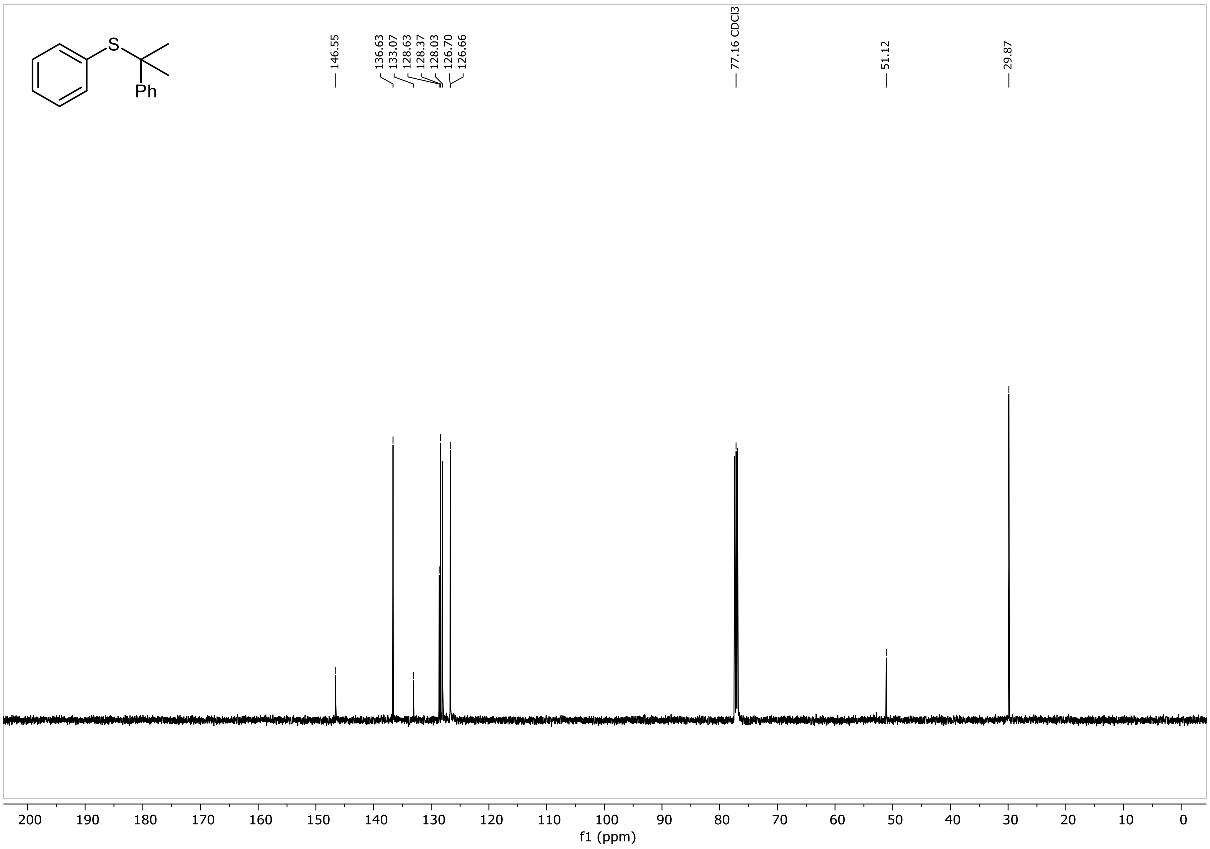

Figure S33. ^{1}H NMR spectrum (500 MHz, $CDCl_3$, 298 K) of phenyl(trityl)sulfane (**1p**)

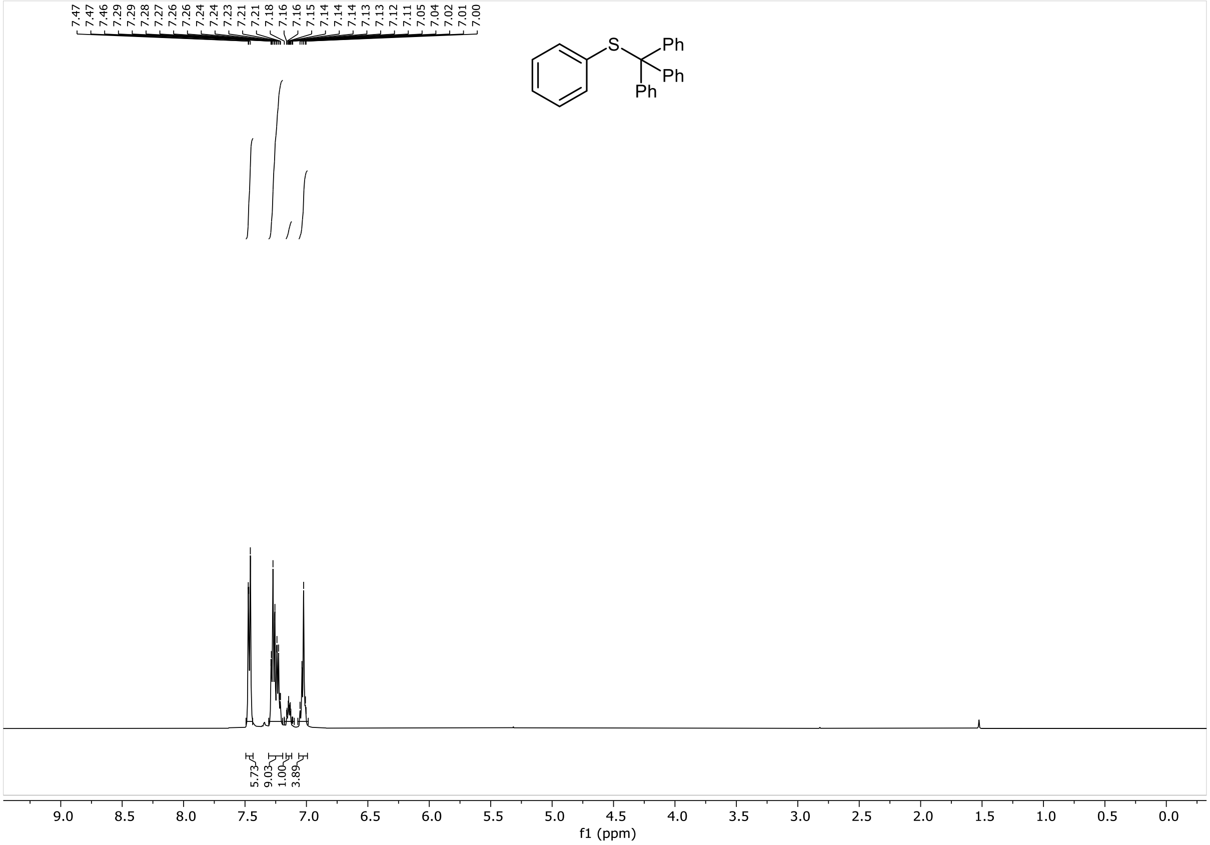

Figure S34. $^{13}C\{^1H\}$ NMR spectrum (126 MHz, $CDCl_3$, 298 K) of **1p**

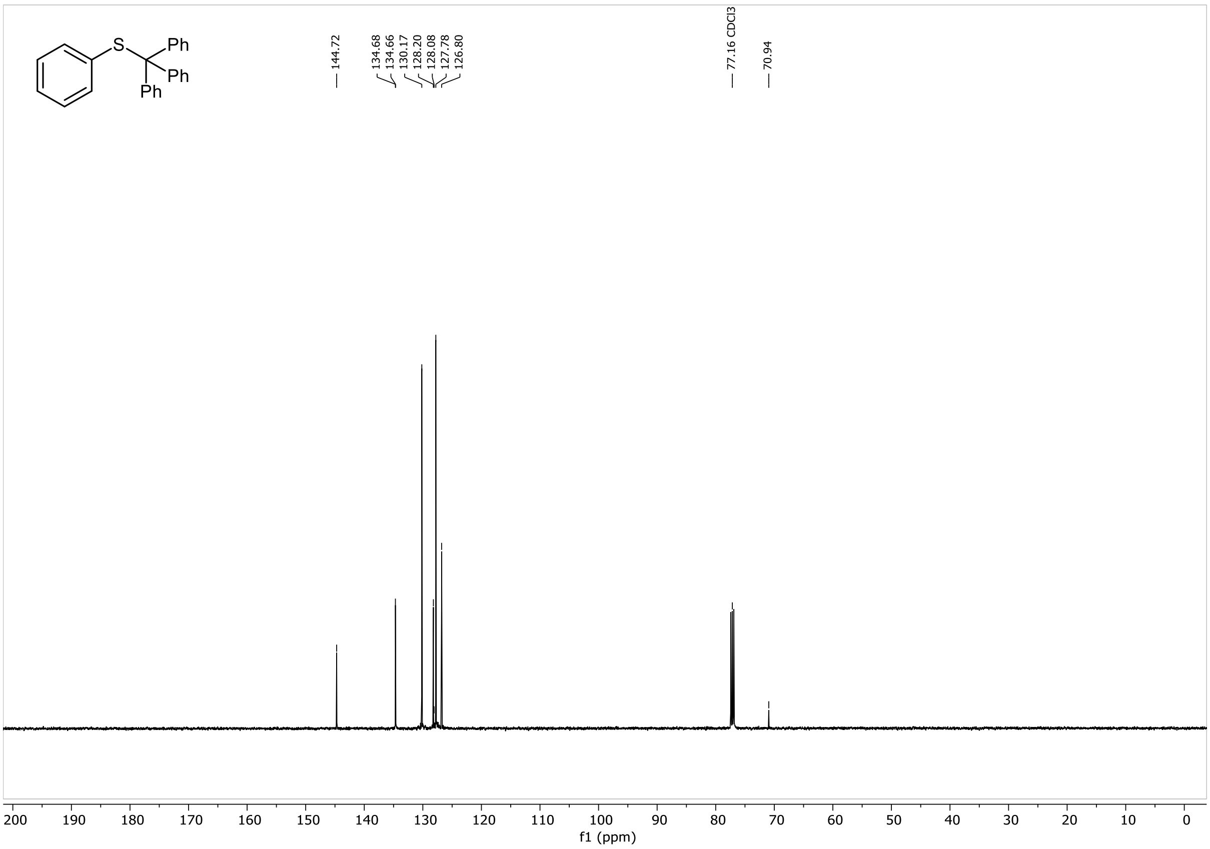

Figure S35. ^{1}H NMR spectrum (500 MHz, $CDCl_3$, 298 K) of benzyl(phenyl)sulfane (**1q**)

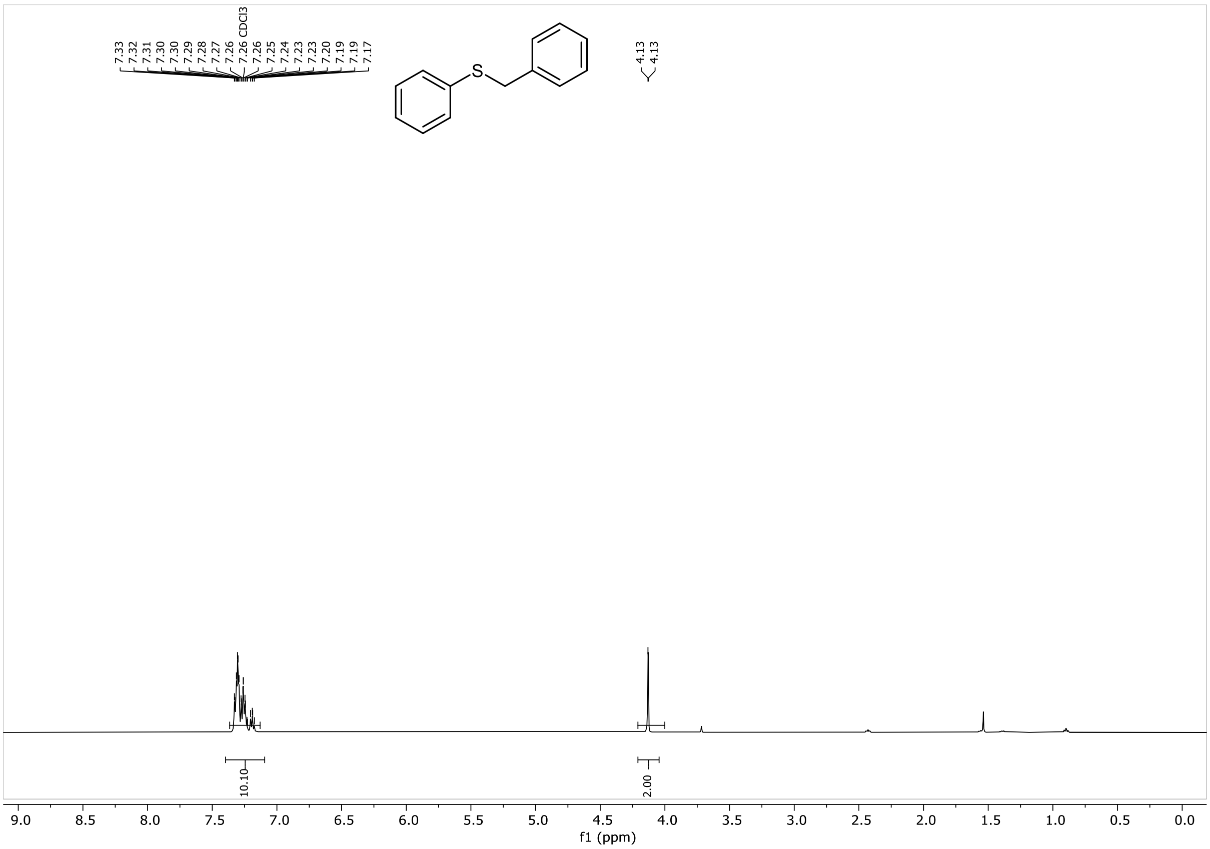

Figure S36. $^{13}C\{^{1}H\}$ NMR spectrum (126 MHz, $CDCl_3$, 298 K) of **1q**

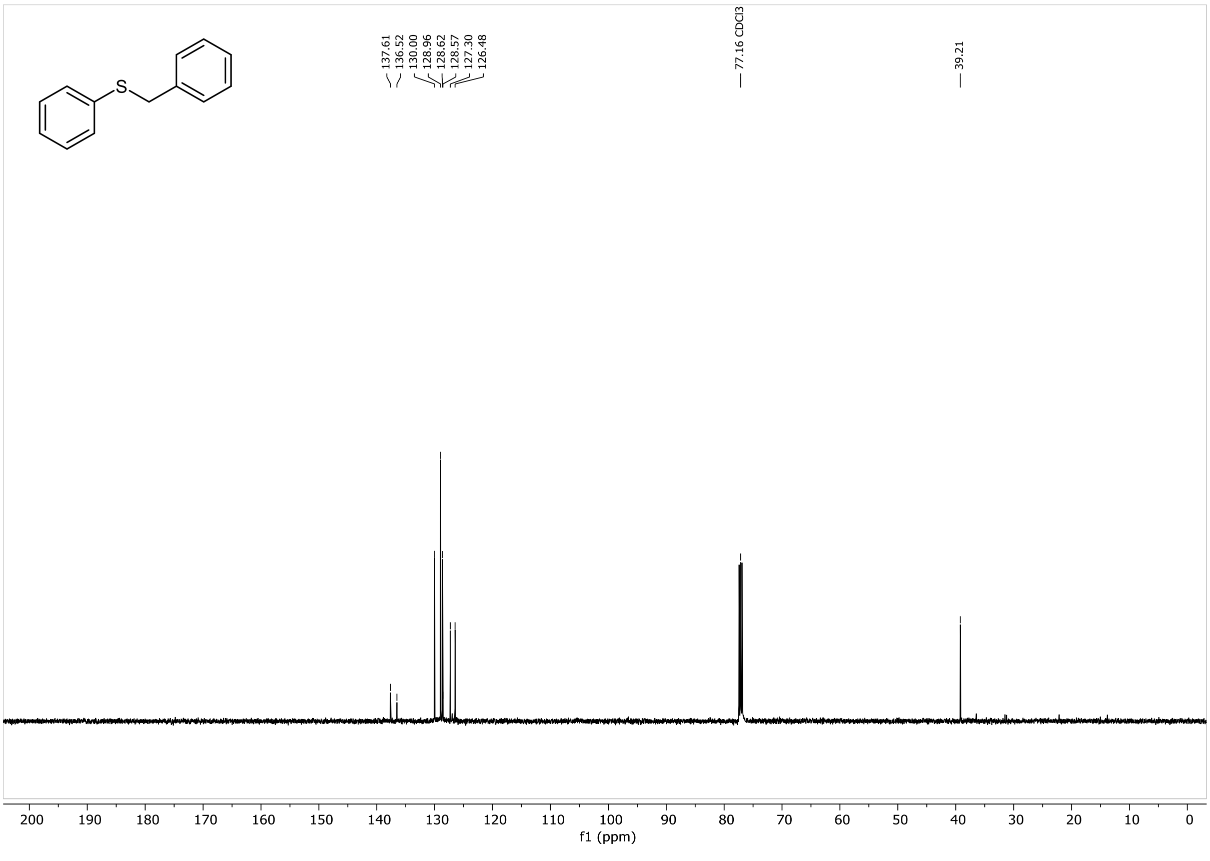

Figure S37. ^{1}H NMR spectrum (500 MHz, $CDCl_3$, 298 K) of isopropyl(phenyl)sulfane (**1r**)

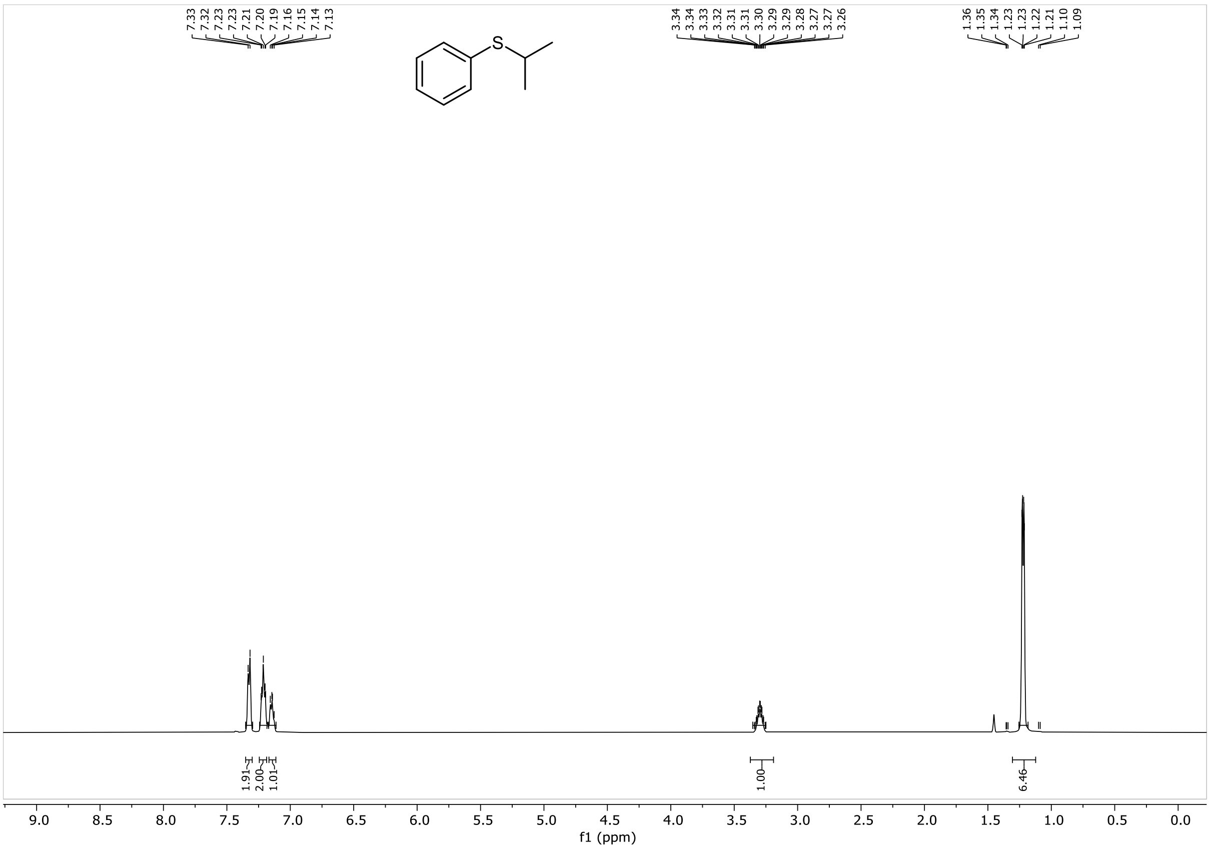

Figure S38. $^{13}C\{^{1}H\}$ NMR spectrum (126 MHz, $CDCl_3$, 298 K) of **1r**

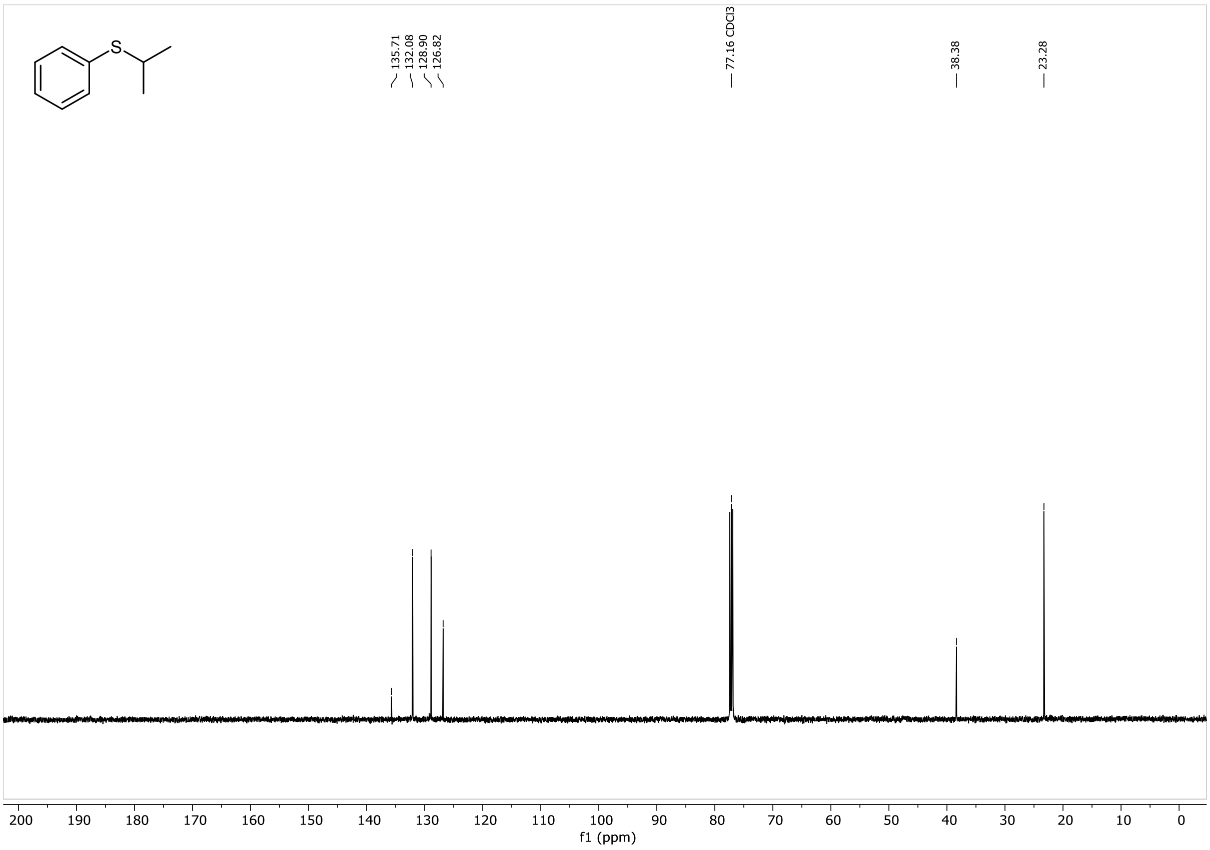

Figure S39. ^{1}H NMR spectrum (500 MHz, $CDCl_3$, 298 K) of *(E)*-(2-ethyl-3,3-dimethyl-1-phenylbut-1-en-1-yl)(phenyl)sulfane (**3aa**)

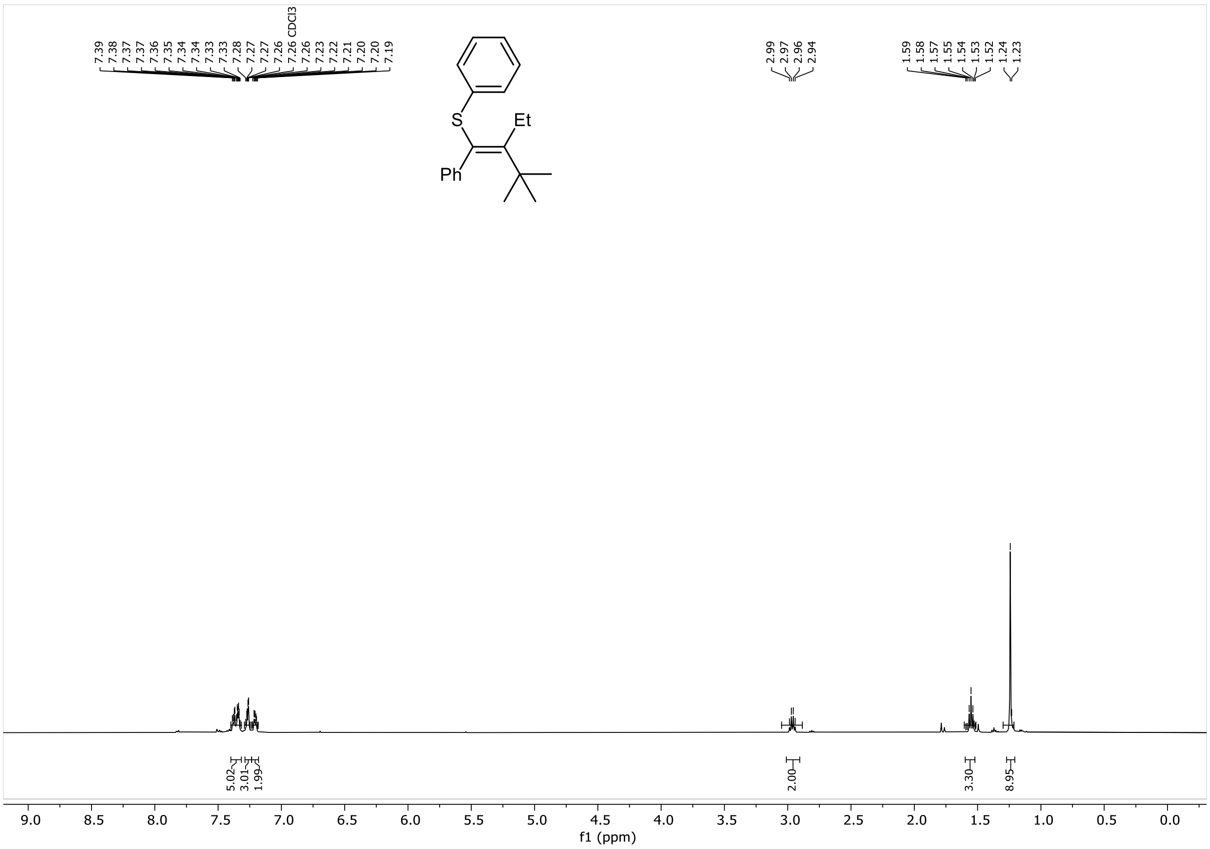

Figure S40. $^{13}C\{^1H\}$ NMR spectrum (126 MHz, $CDCl_3$, 298 K) of **3aa**

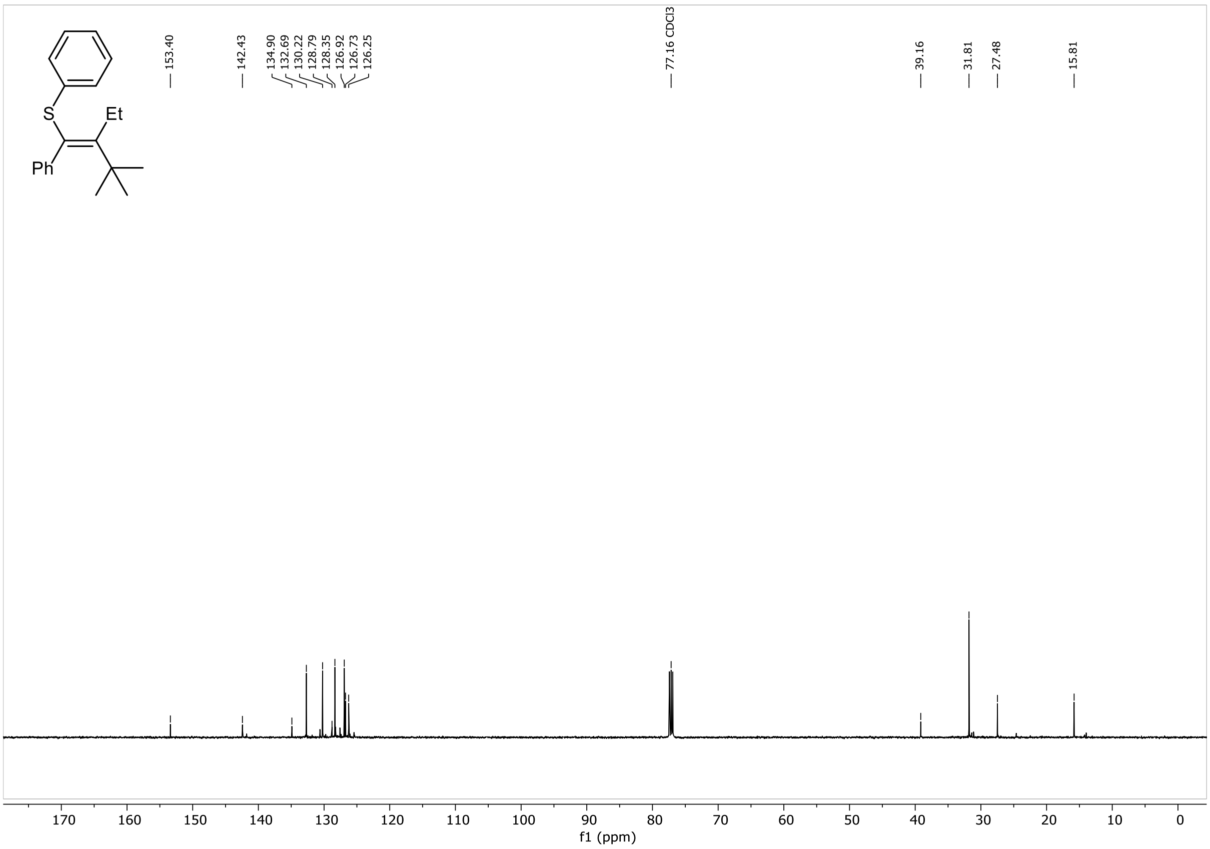

Figure S41. ^{1}H NMR spectrum (500 MHz, $CDCl_3$, 298 K) of *(E)*-(2-ethyl-3,3-dimethyl-1-phenylbut-1-en-1-yl)(p-tolyl)sulfane (**3ba**)

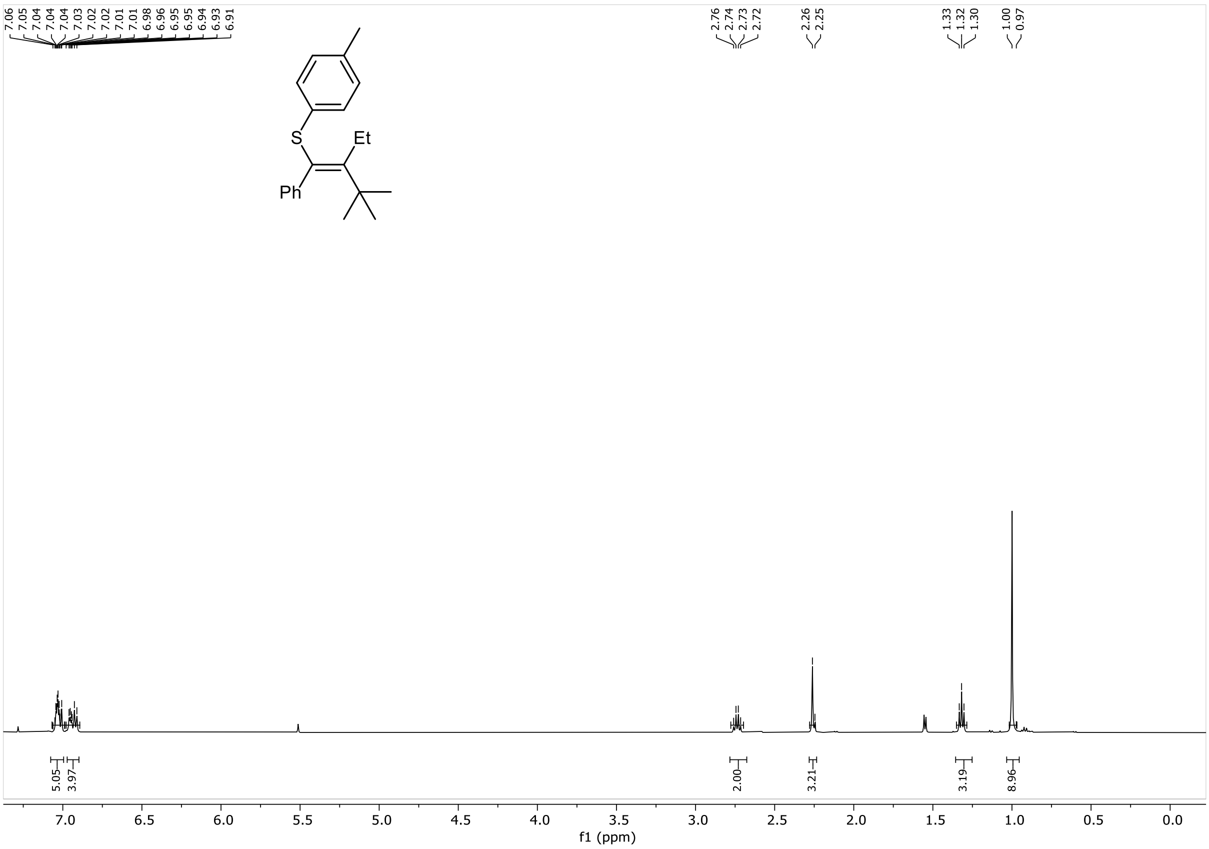

Figure S42. $^{13}C\{^{1}H\}$ NMR spectrum (126 MHz, $CDCl_3$, 298 K) of **3ba**

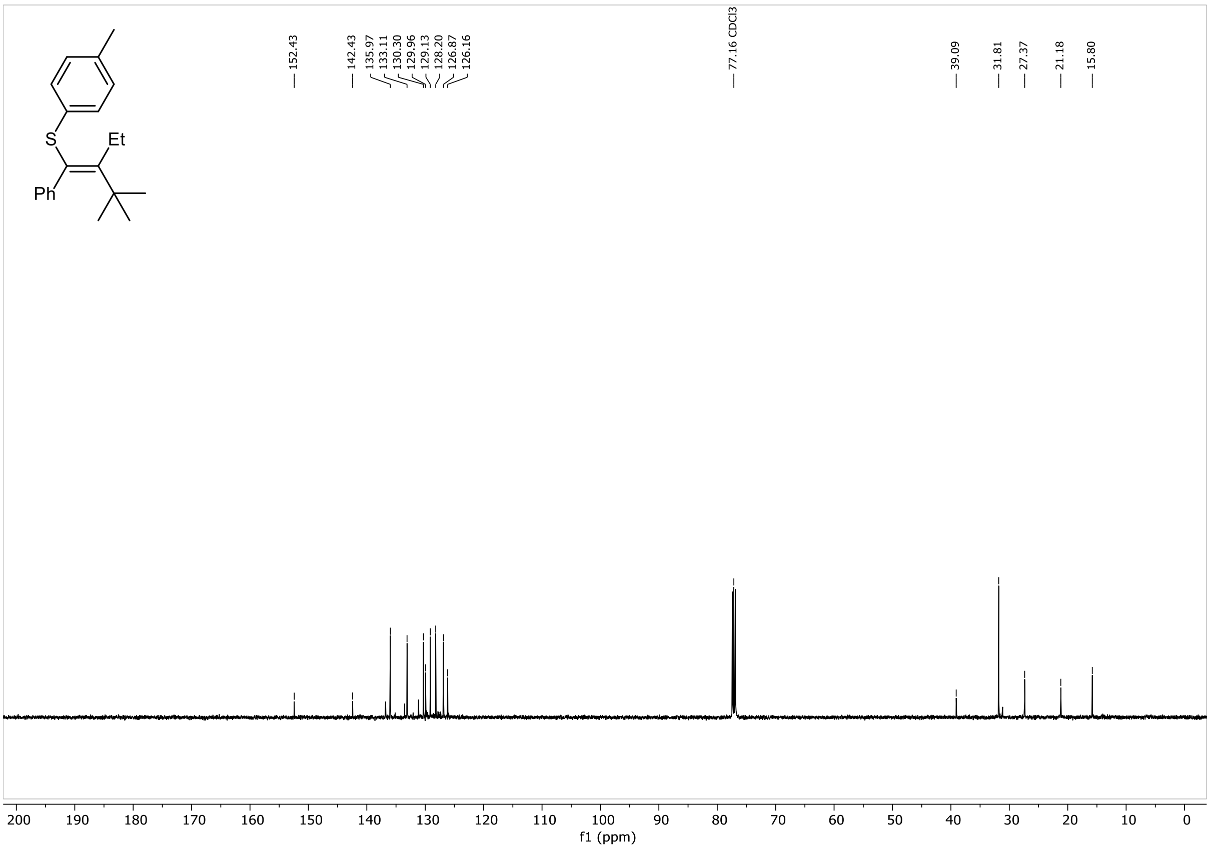

Figure S43. ^{1}H NMR spectrum (500 MHz, $CDCl_3$, 298 K) of *(E)*-(2-ethyl-3,3-dimethyl-1-phenylbut-1-en-1-yl)(4-fluorophenyl)sulfane (**3ca**)

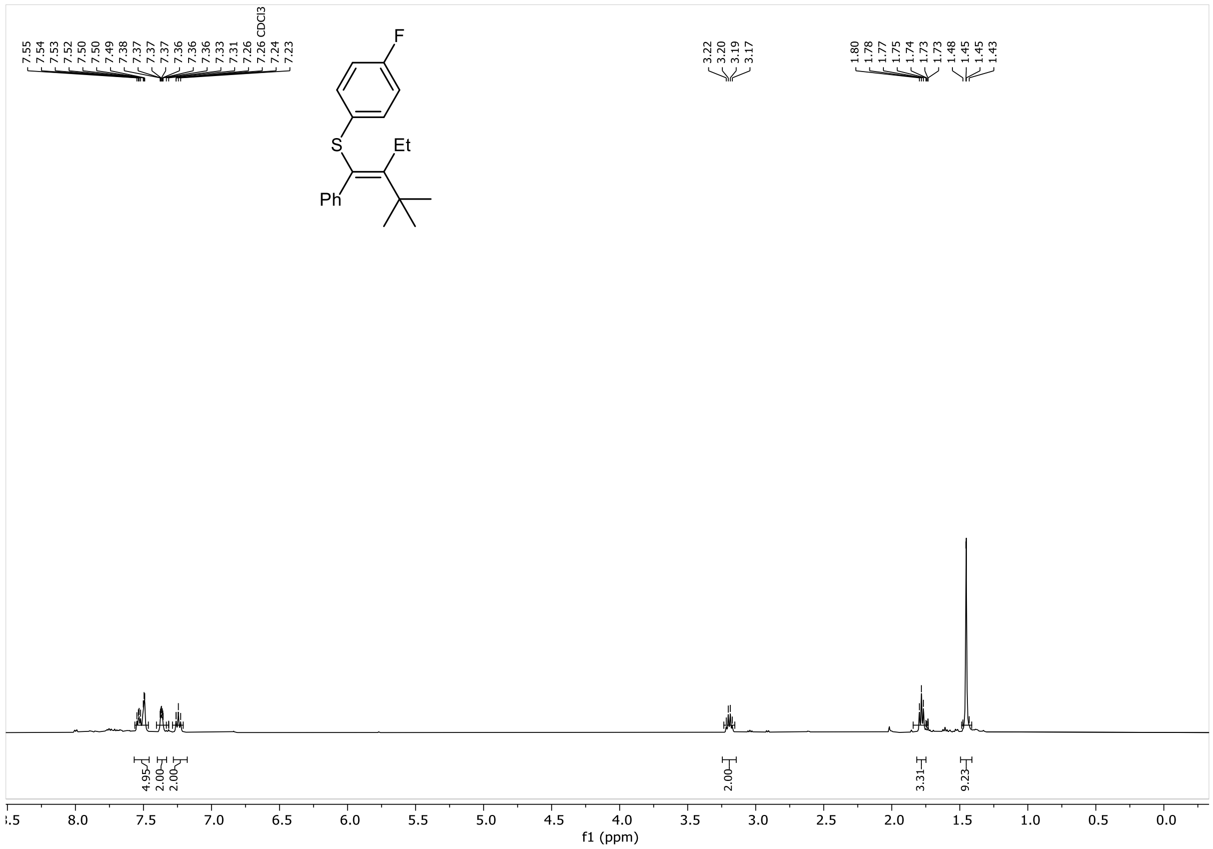

Figure S44. $^{13}C\{^{1}H\}$ NMR spectrum (126 MHz, $CDCl_3$, 298 K) of **3ca**

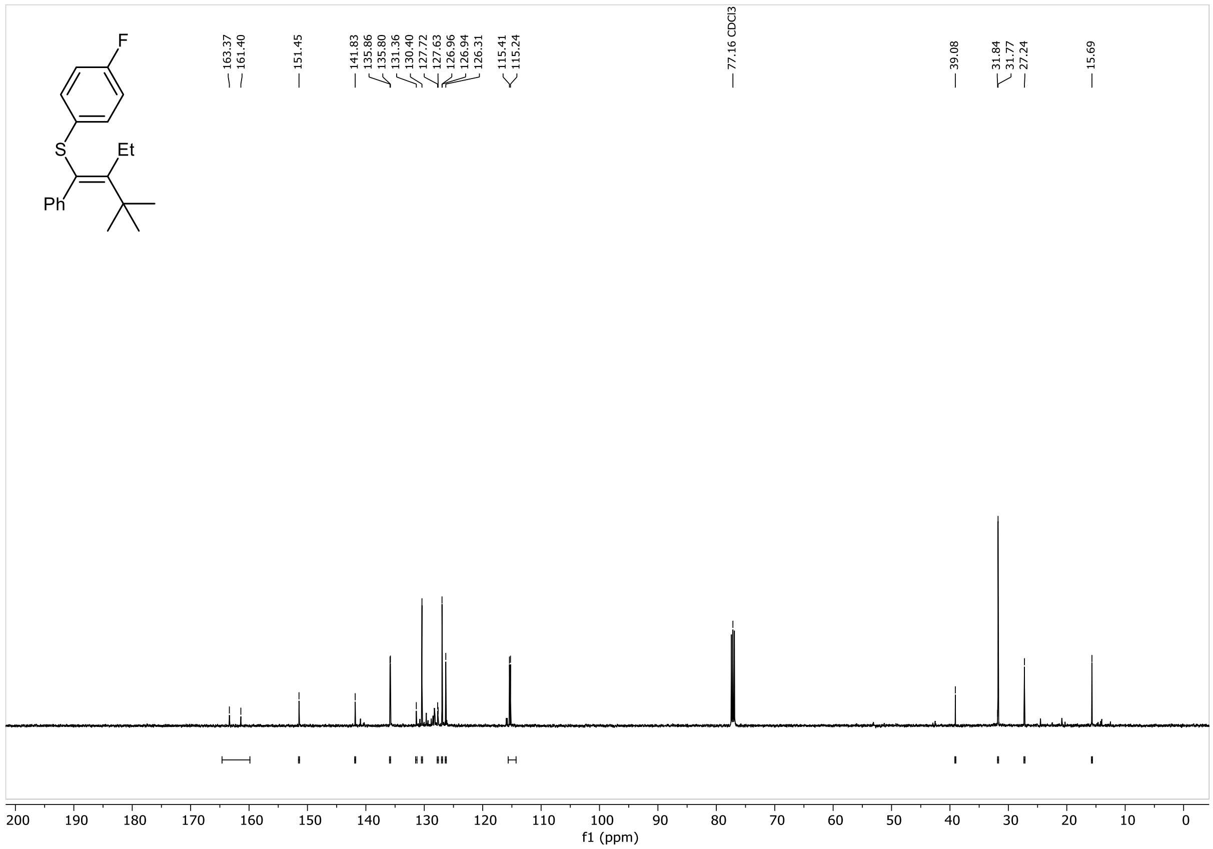

Figure S45. ^{1}H NMR spectrum (500 MHz, $CDCl_3$, 298 K) of *(E)*-(4-chlorophenyl)(2-ethyl-3,3-dimethyl-1-phenylbut-1-en-1-yl)sulfane (**3da**)

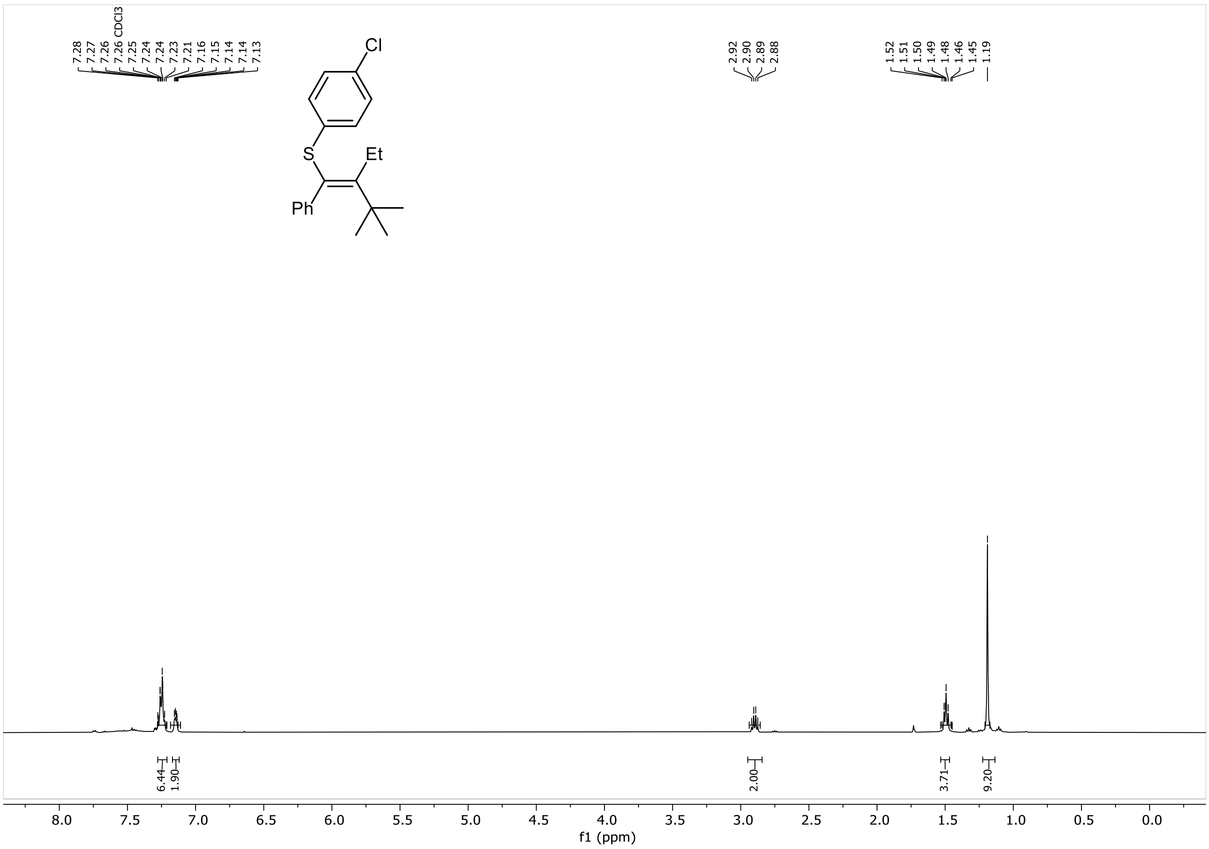

Figure S46. $^{13}C\{^{1}H\}$ NMR spectrum (126 MHz, $CDCl_3$, 298 K) of **3da**

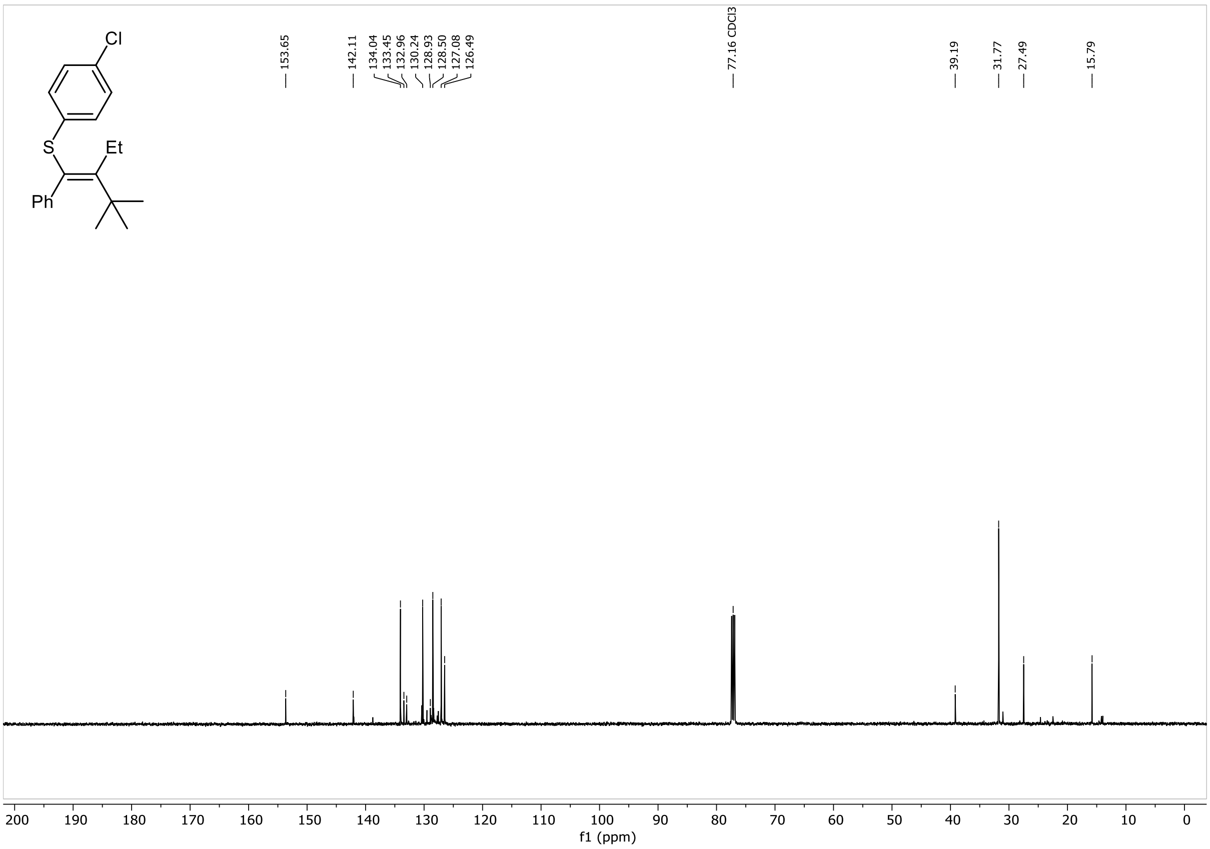

Figure S47. ^{1}H NMR spectrum (500 MHz, $CDCl_3$, 298 K) of *(E)*-(4-bromophenyl)(2-ethyl-3,3-dimethyl-1-phenylbut-1-en-1-yl)sulfane (**3ea**)

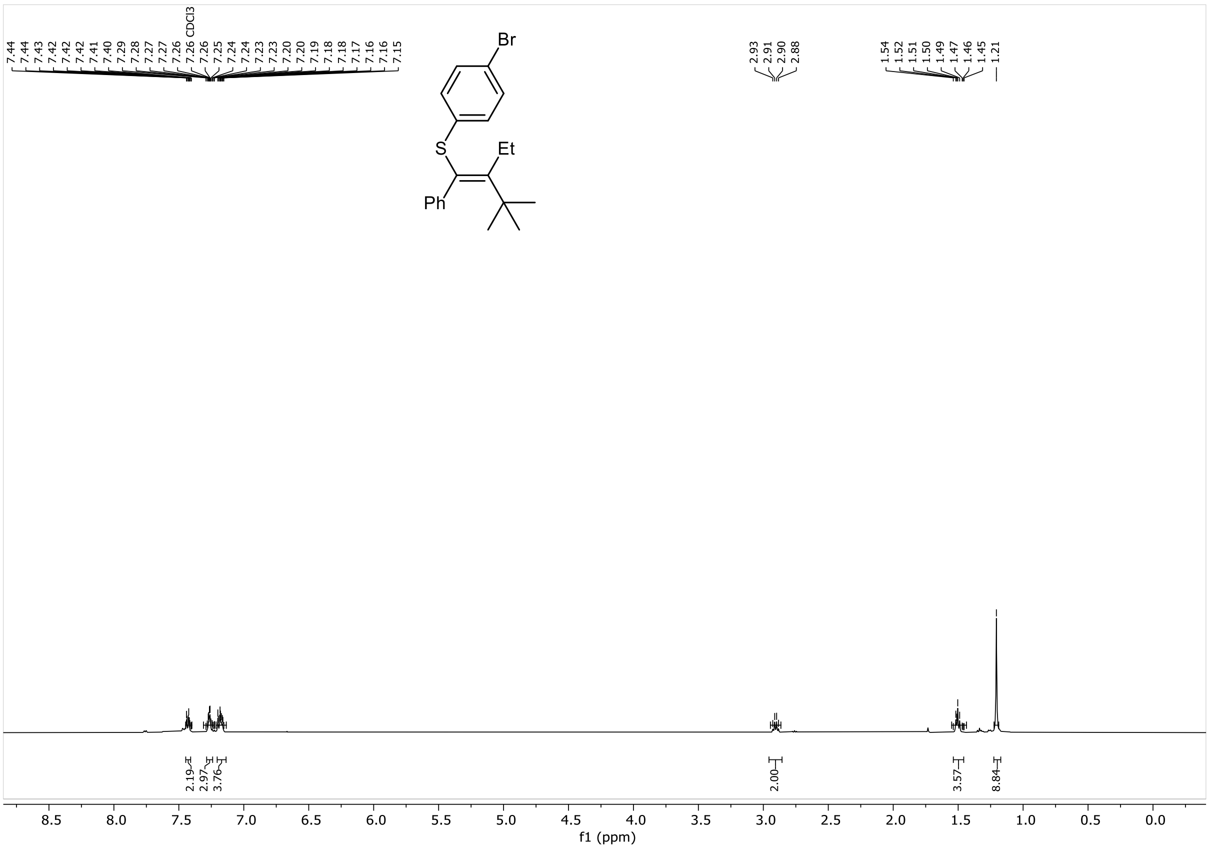

Figure S48. $^{13}C\{^{1}H\}$ NMR spectrum (126 MHz, $CDCl_3$, 298 K) of **3ea**

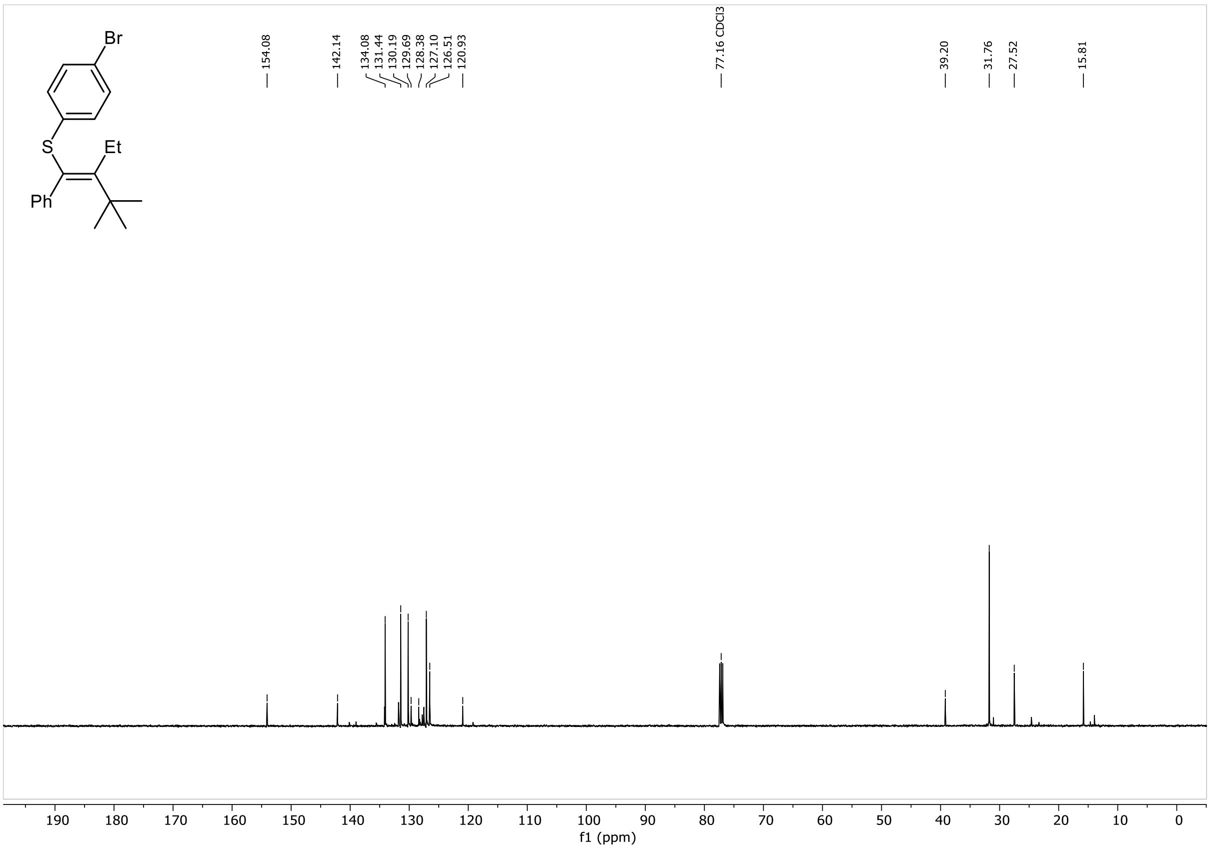

Figure S49. ^{1}H NMR spectrum (500 MHz, $CDCl_3$, 298 K) of *(E)*-(4-(*tert*-butyl)phenyl)(2-ethyl-3,3-dimethyl-1-phenylbut-1-en-1-yl)sulfane (**3fa**)

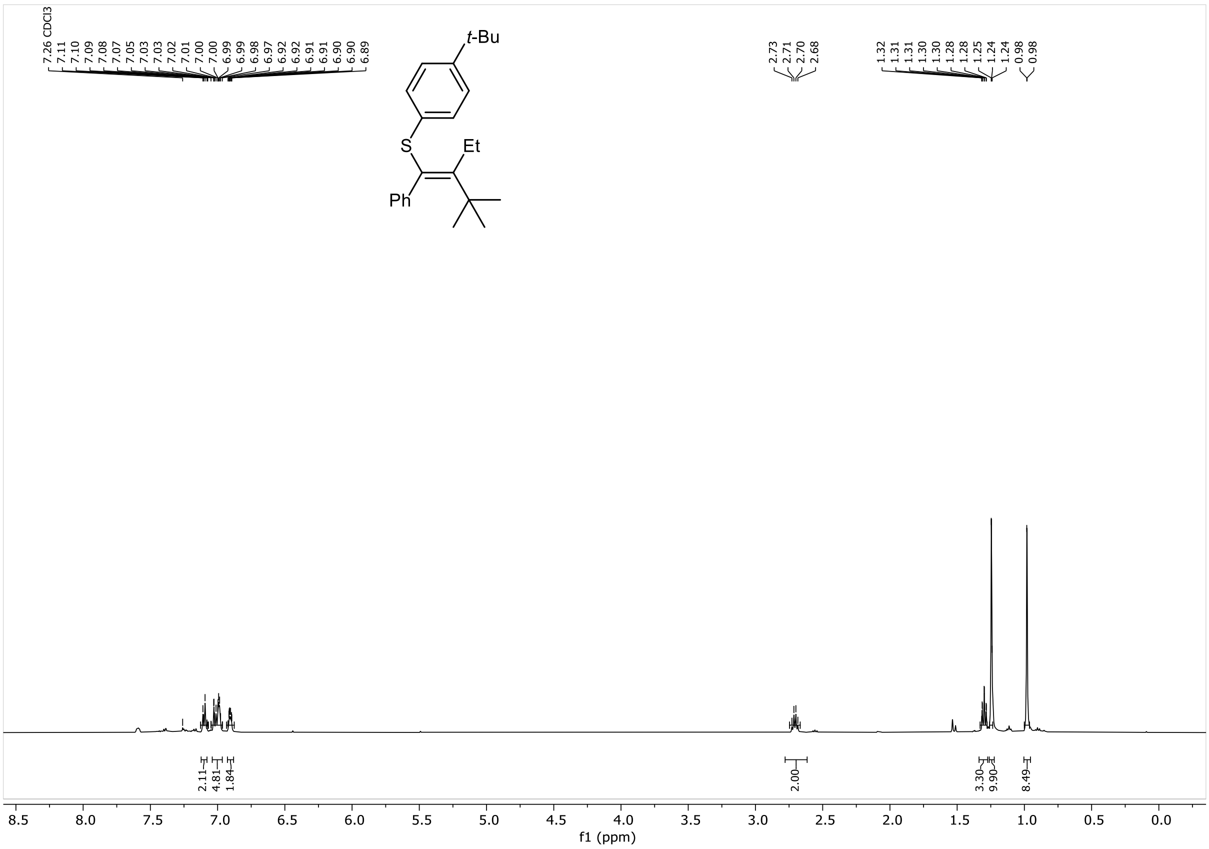

Figure S50. $^{13}C\{^{1}H\}$ NMR spectrum (126 MHz, $CDCl_3$, 298 K) of **3fa**

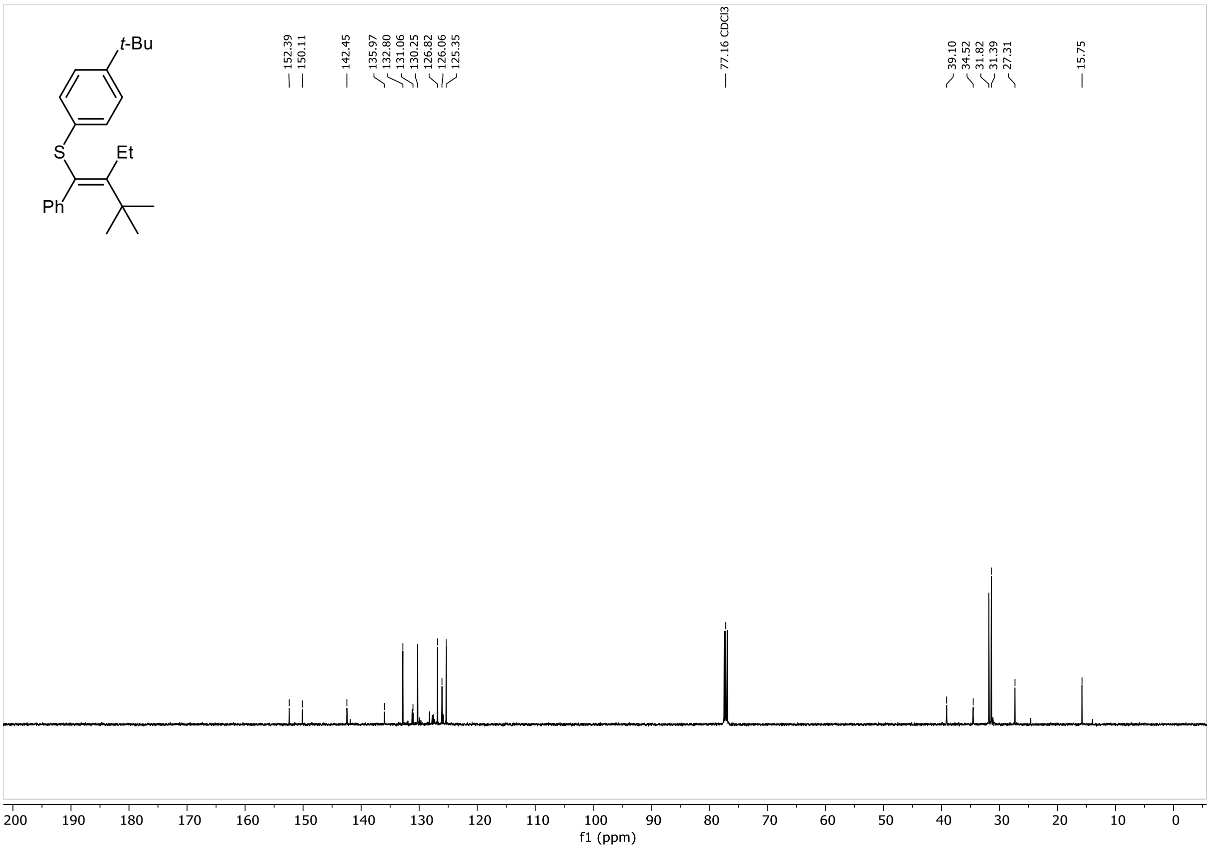

Figure S51. ^{1}H NMR spectrum (500 MHz, $CDCl_3$, 298 K) of methyl *(E)*-4-((2-ethyl-3,3-dimethyl-1-phenylbut-1-en-1-yl)thio)benzoate (**3ga**)

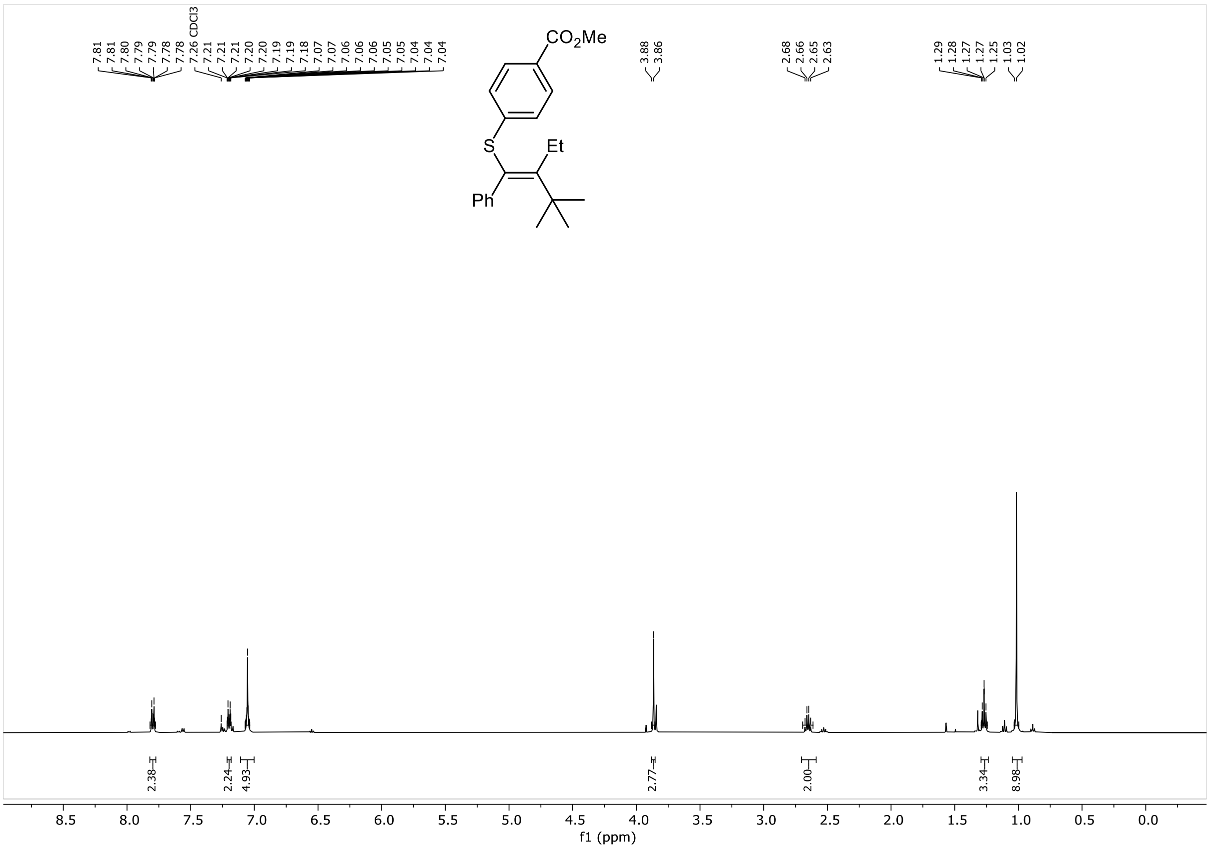

Figure S52. $^{13}C\{^{1}H\}$ NMR spectrum (126 MHz, $CDCl_3$, 298 K) of **3ga**

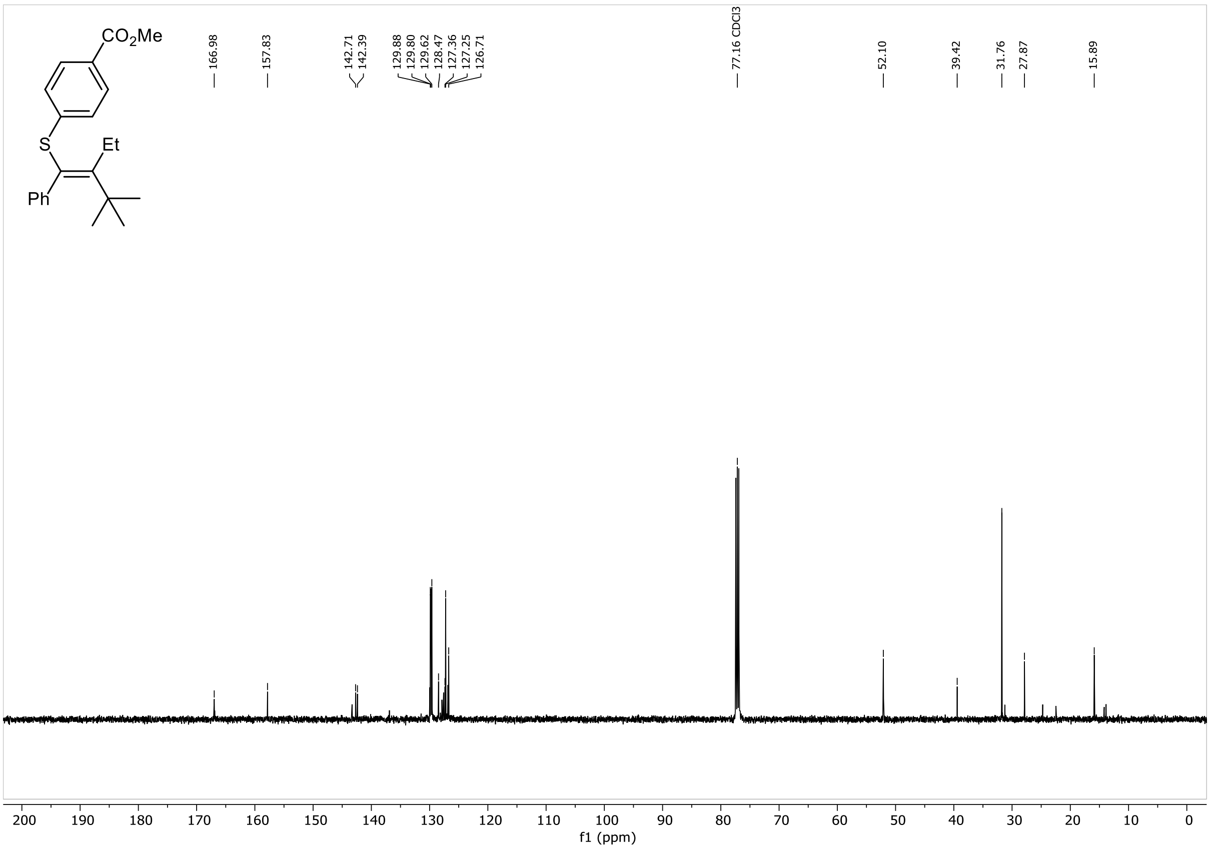

Figure S53. ^{1}H NMR spectrum (500 MHz, $CDCl_3$, 298 K) of ethyl *(E)*-4-((2-ethyl-3,3-dimethyl-1-phenylbut-1-en-1-yl)thio)benzoate (**3ha**)

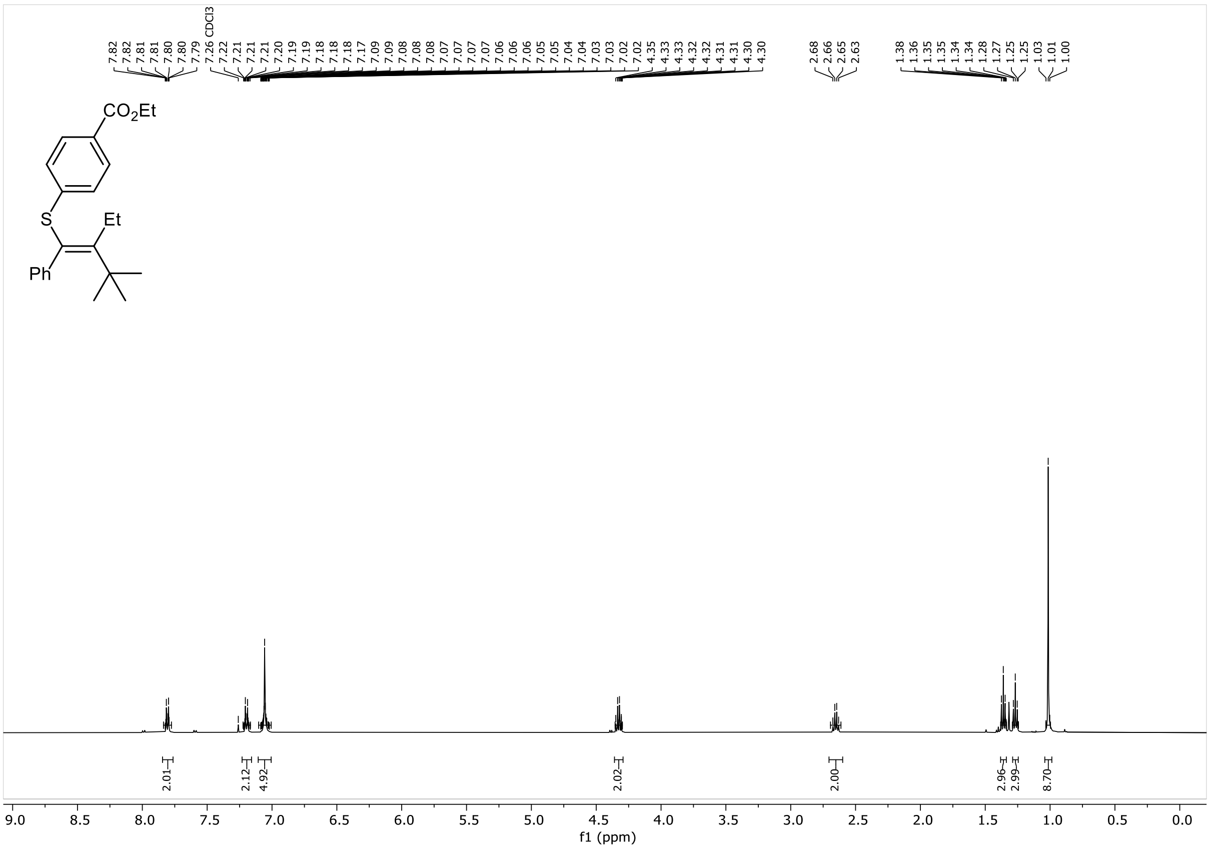

Figure S54. $^{13}C\{^{1}H\}$ NMR spectrum (126 MHz, $CDCl_3$, 298 K) of **3ha**

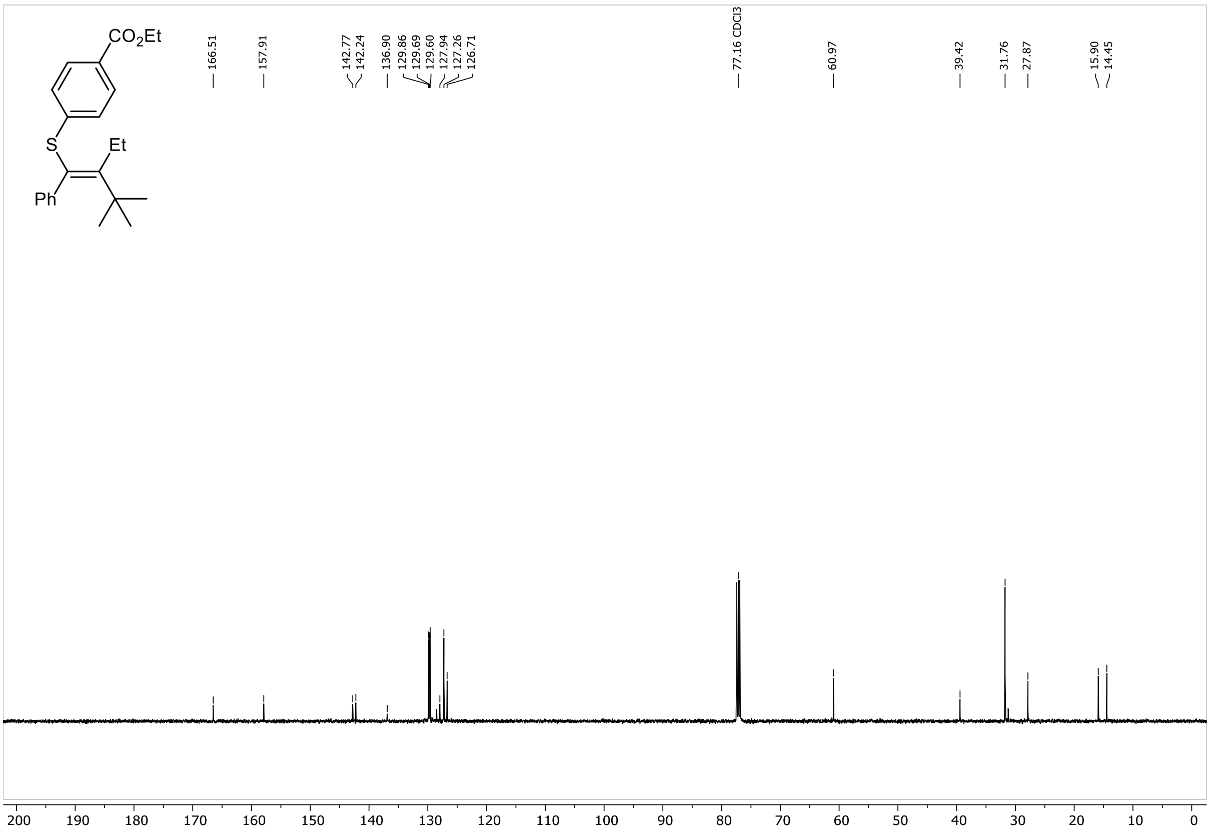

Figure S55. ^{1}H NMR spectrum (500 MHz, $CDCl_3$, 298 K) of *(E)*-(2-ethyl-3,3-dimethyl-1-phenylbut-1-en-1-yl)(naphthalen-2-yl)sulfane (**3ia**)

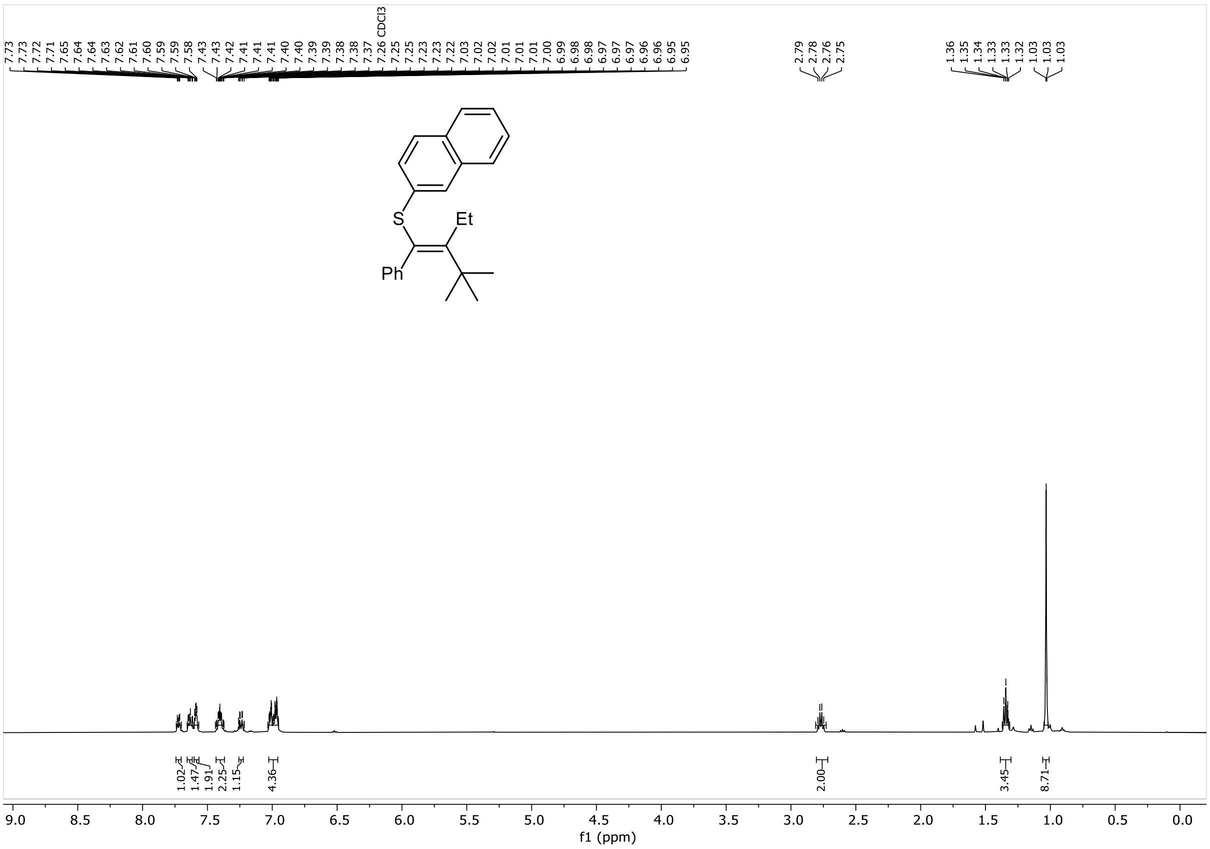

Figure S56. $^{13}C\{^{1}H\}$ NMR spectrum (126 MHz, $CDCl_3$, 298 K) of **3ia**

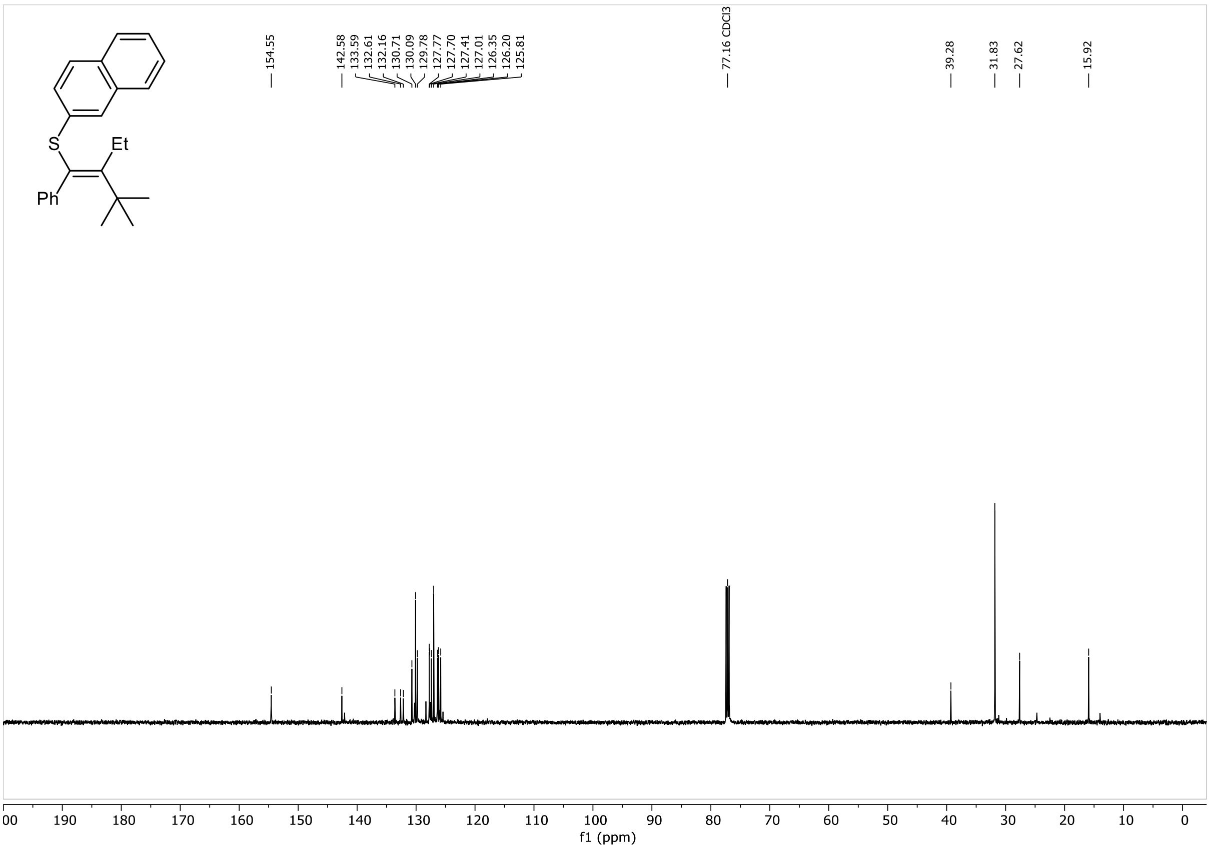

Figure S57. ^{1}H NMR spectrum (500 MHz, $CDCl_3$, 298 K) of *(E)*-(2-ethyl-3,3-dimethyl-1-phenylbut-1-en-1-yl)(*m*-tolyl)sulfane (**3ja**)

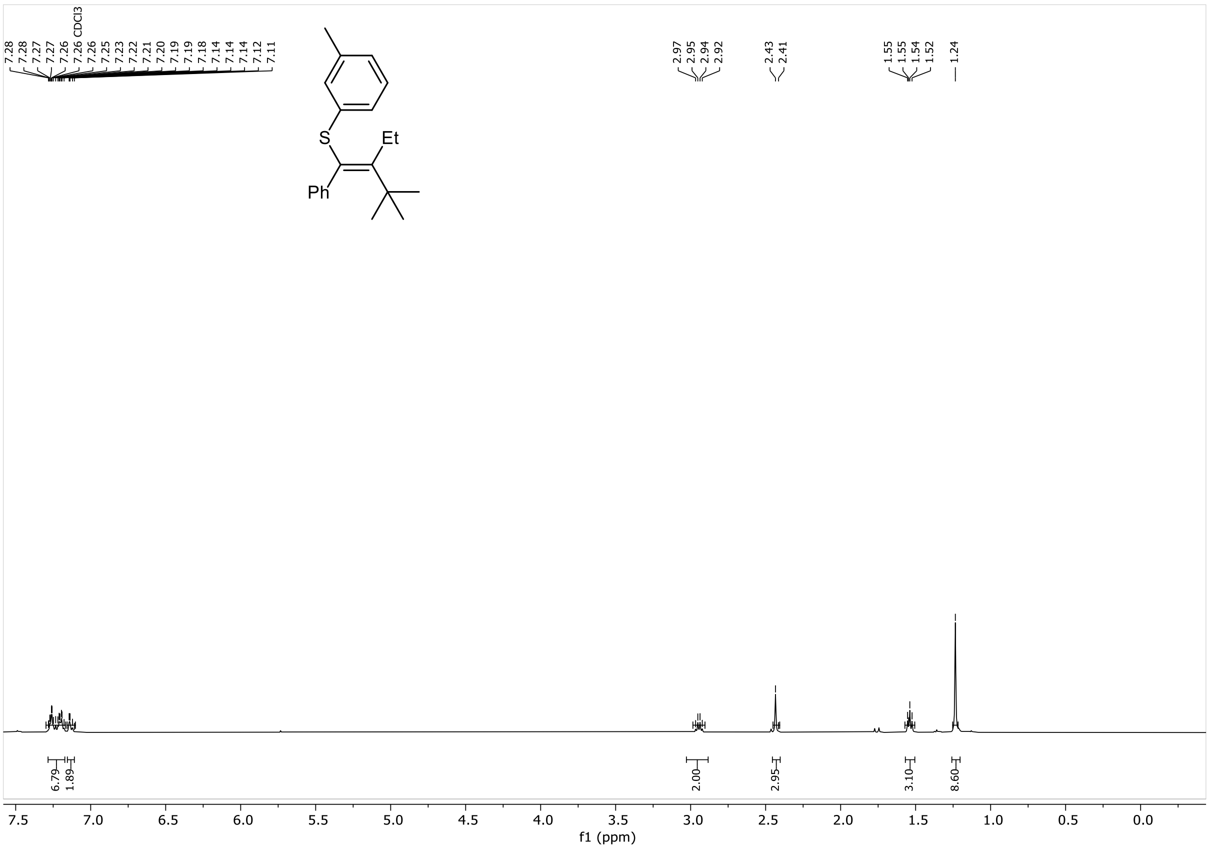

Figure S58. $^{13}C\{^{1}H\}$ NMR spectrum (126 MHz, $CDCl_3$, 298 K) of **3ja**

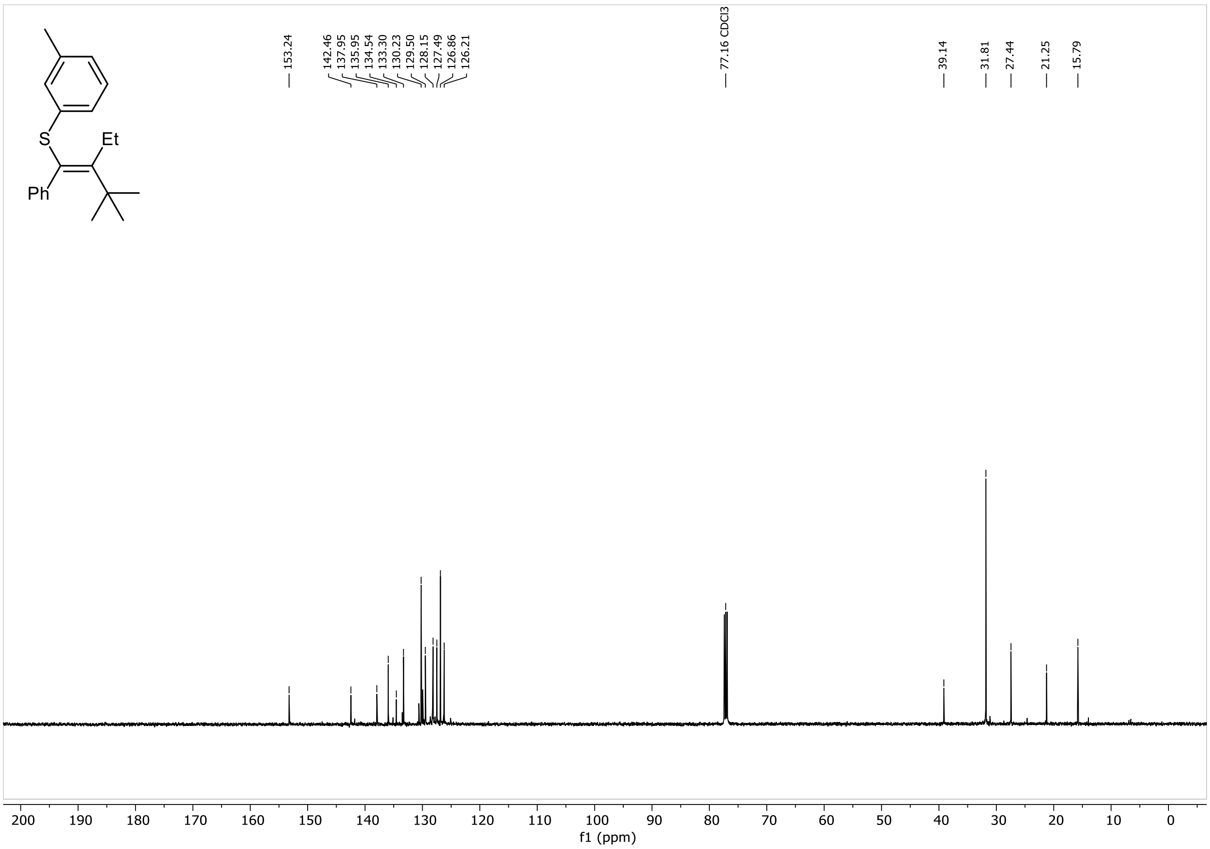

Figure S59. ^{1}H NMR spectrum (500 MHz, $CDCl_3$, 298 K) of *(E)*-(2-ethyl-3,3-dimethyl-1-phenylbut-1-en-1-yl)(o-tolyl)sulfane (**3ka**)

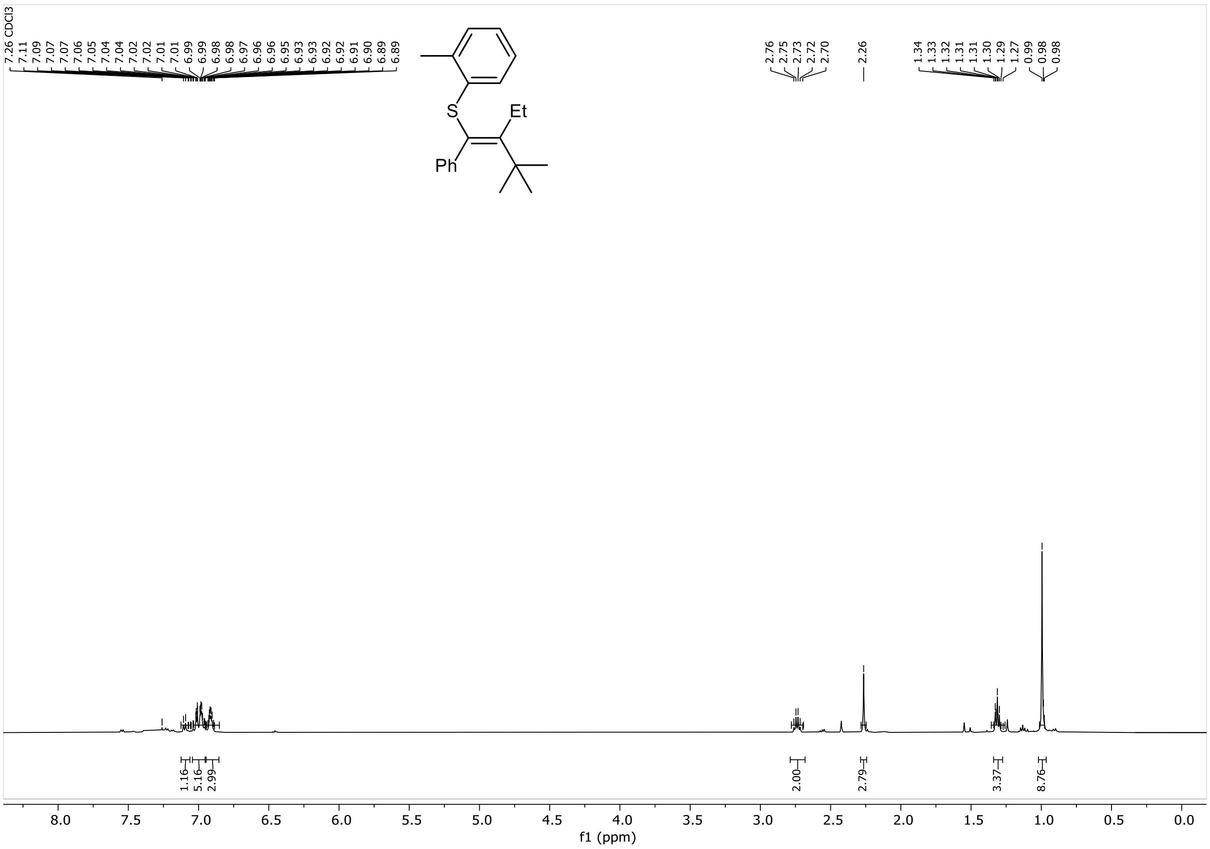

Figure S60. $^{13}C\{^{1}H\}$ NMR spectrum (126 MHz, $CDCl_3$, 298 K) of **3ka**

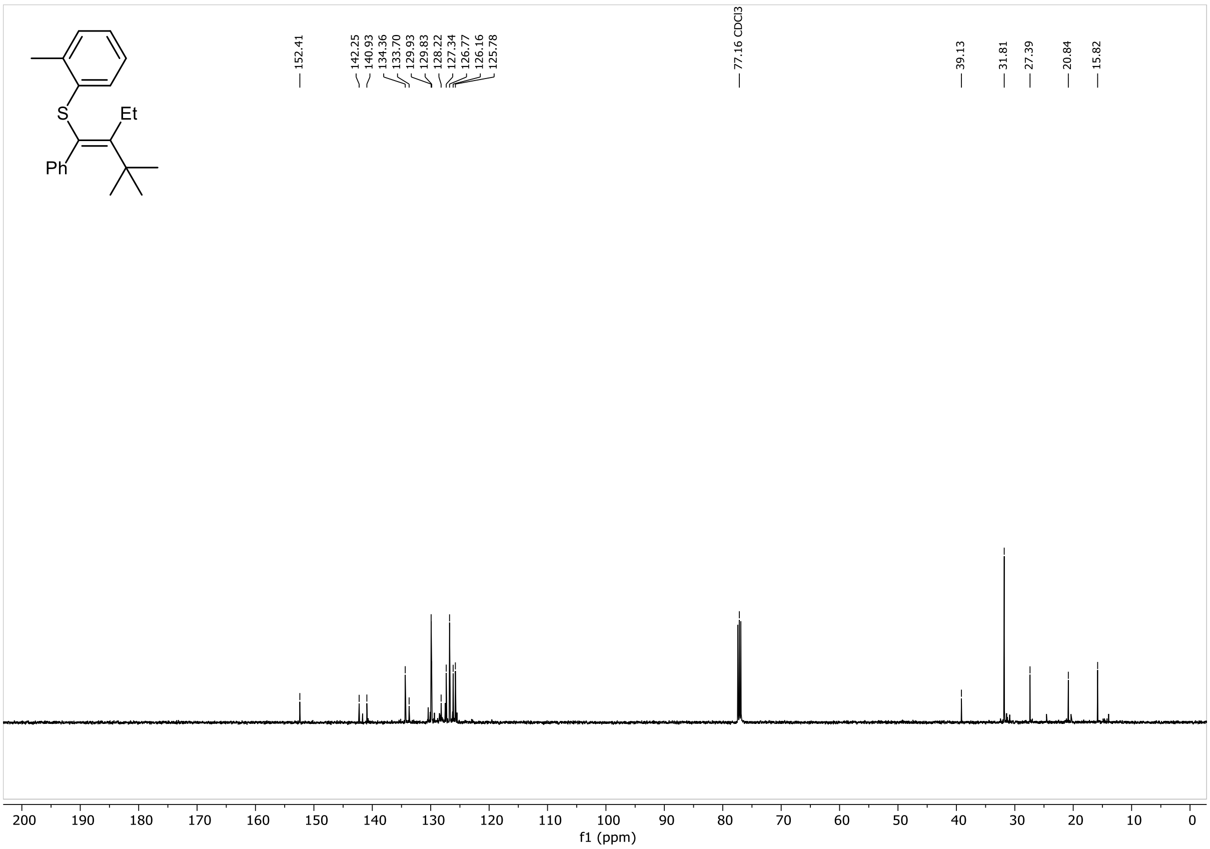

Figure S61. ^{1}H NMR spectrum (500 MHz, $CDCl_3$, 298 K) of *(E)*-(2-ethyl-3,3-dimethyl-1-phenylpent-1-en-1-yl)(phenyl)sulfane (**3la**)

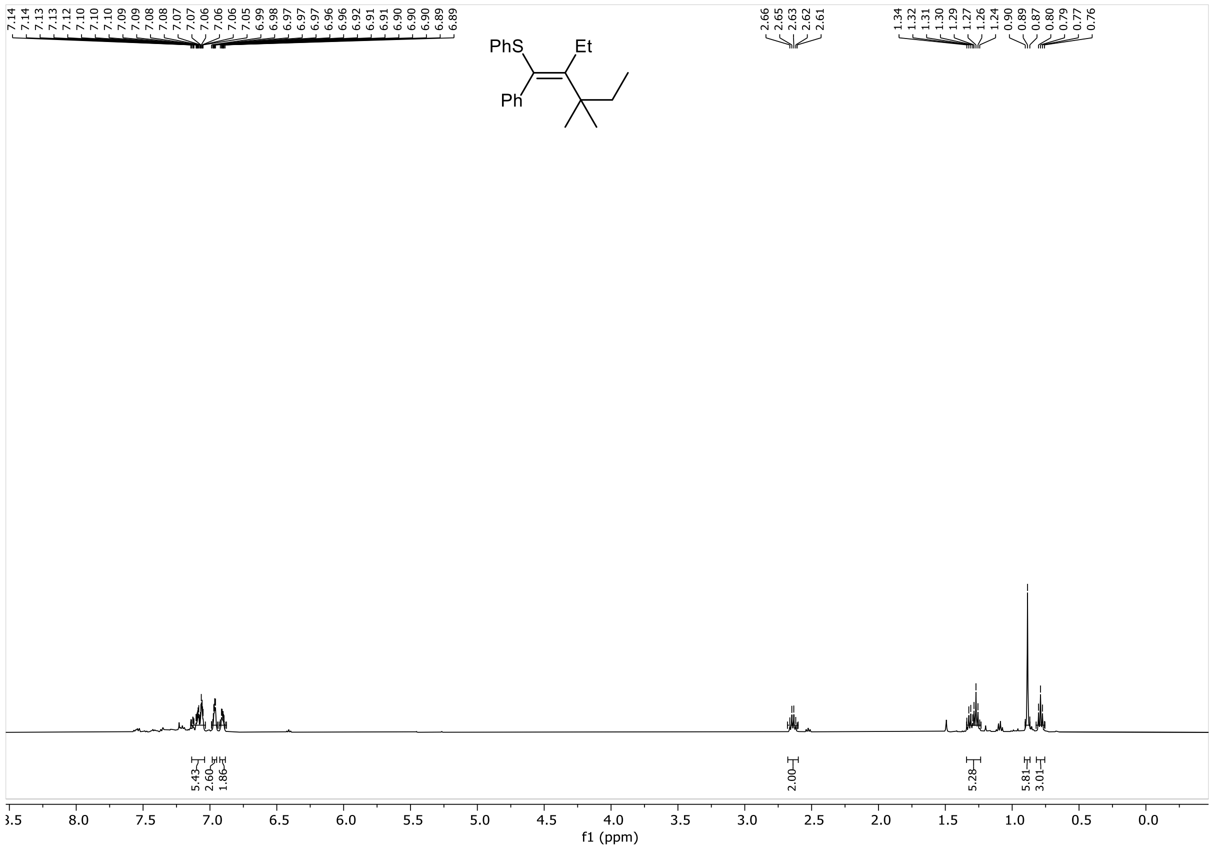

Figure S62. $^{13}C\{^1H\}$ NMR spectrum (126 MHz, $CDCl_3$, 298 K) of **3la**

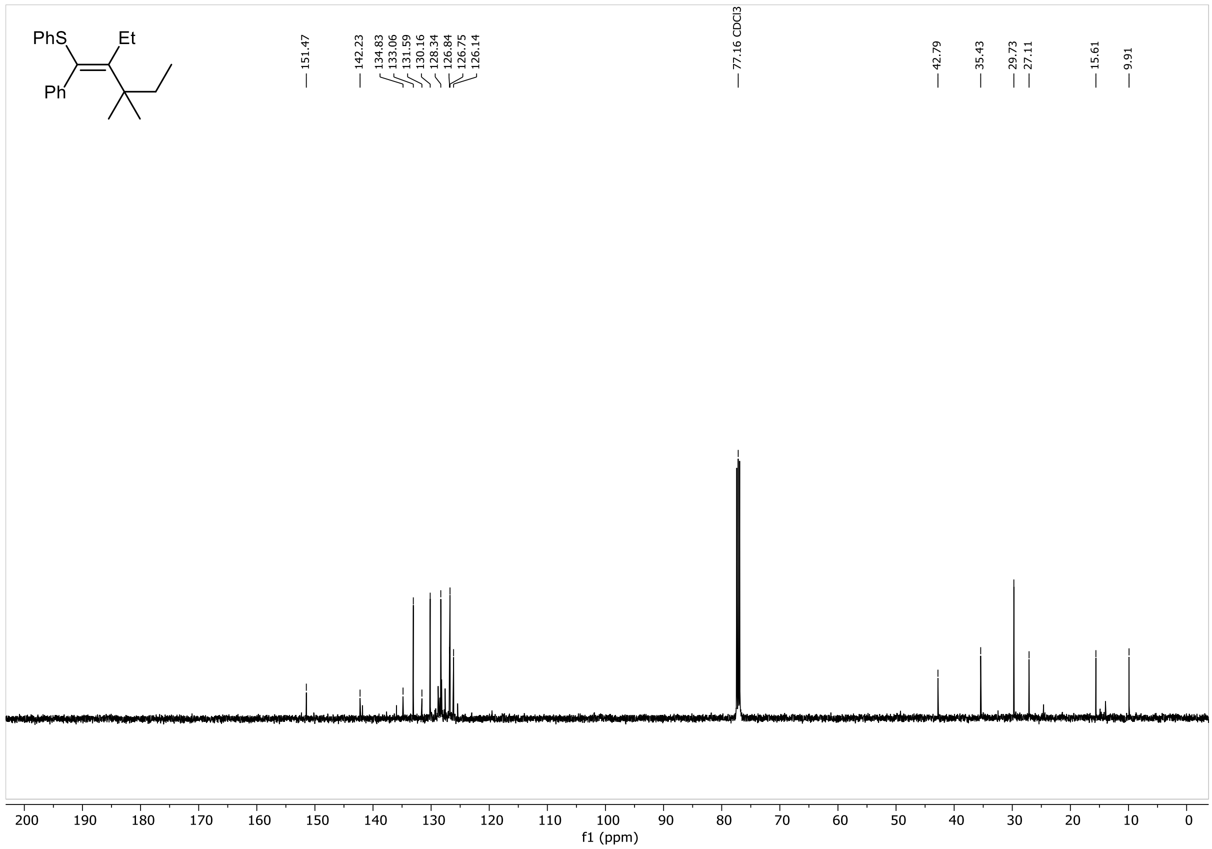

Figure S63. ^{1}H NMR spectrum (500 MHz, $CDCl_3$, 298 K) of (*(E)*-2-((3r,5r,7r)-adamantan-1-yl)-1-phenylbut-1-en-1-yl)(phenyl)sulfane (**3na**)

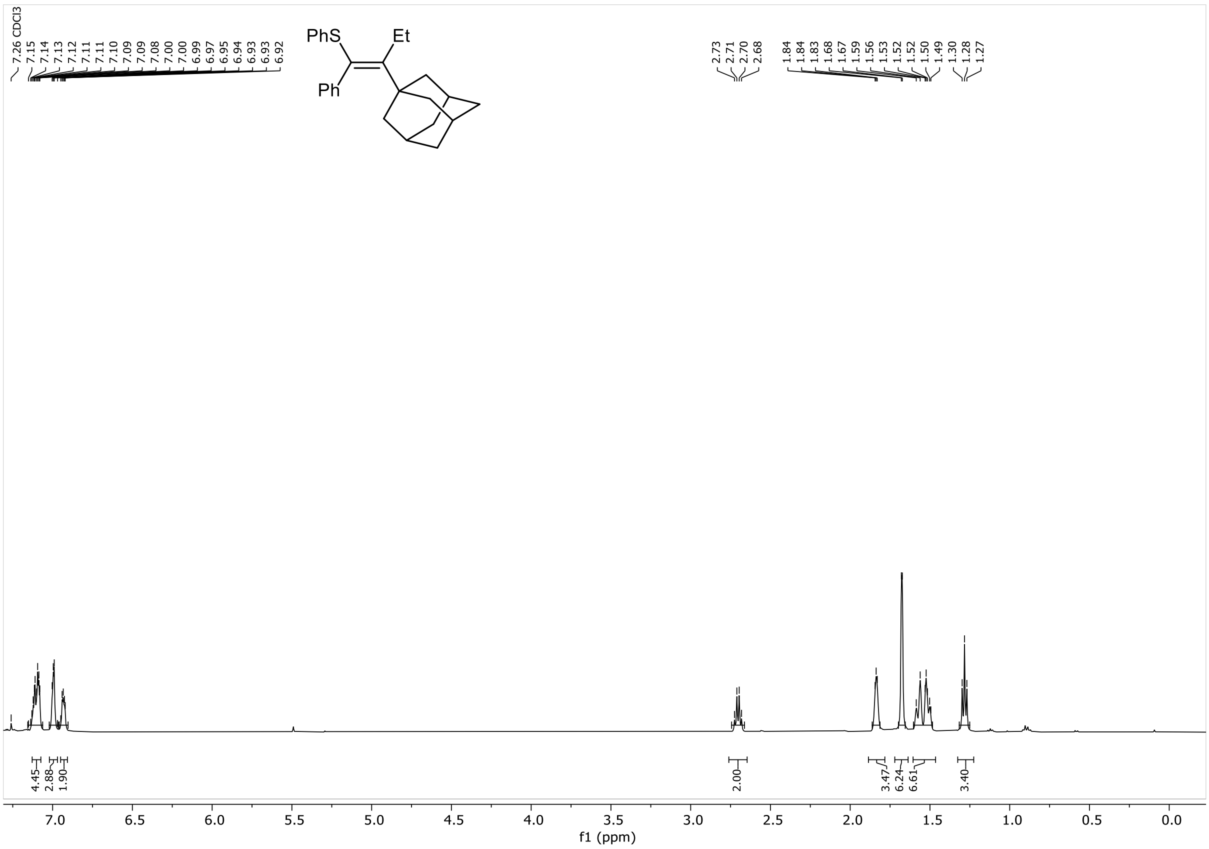

Figure S64. $^{13}C\{^{1}H\}$ NMR spectrum (126 MHz, $CDCl_3$, 298 K) of **3na**

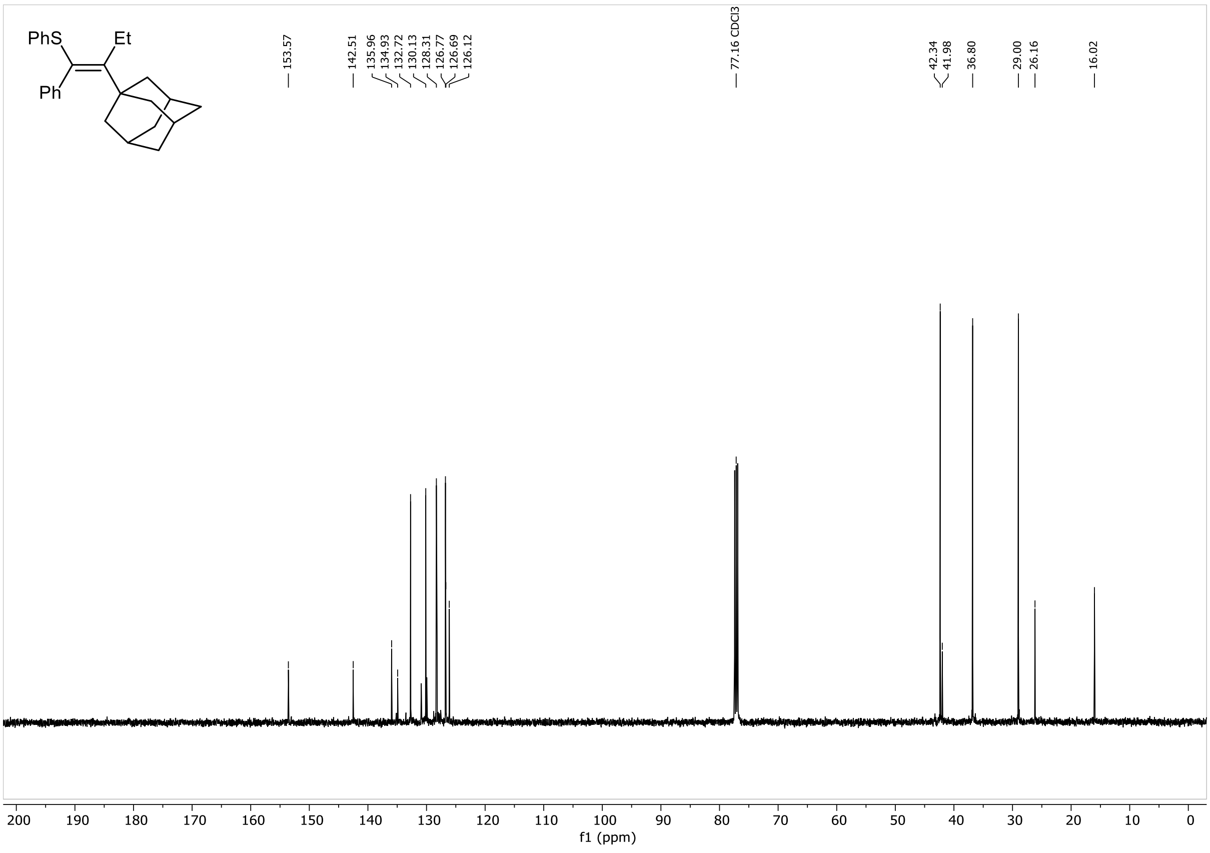

Figure S65. ^{1}H NMR spectrum (500 MHz, $CDCl_3$, 298 K) of *(E)*-(2-ethyl-3-methyl-1,3-diphenylbut-1-en-1-yl)(phenyl)sulfane (**3oa**)

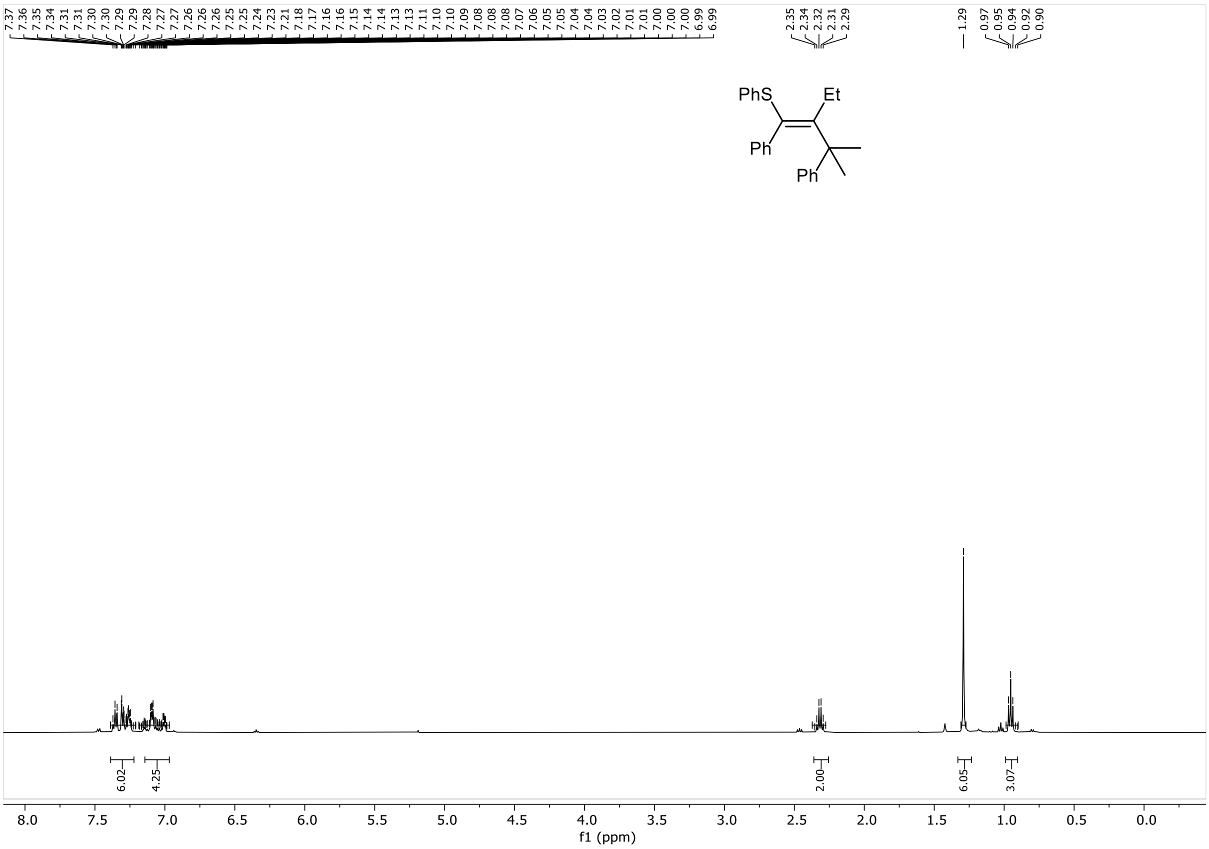

Figure S66. $^{13}C\{^{1}H\}$ NMR spectrum (126 MHz, $CDCl_3$, 298 K) of **3oa**

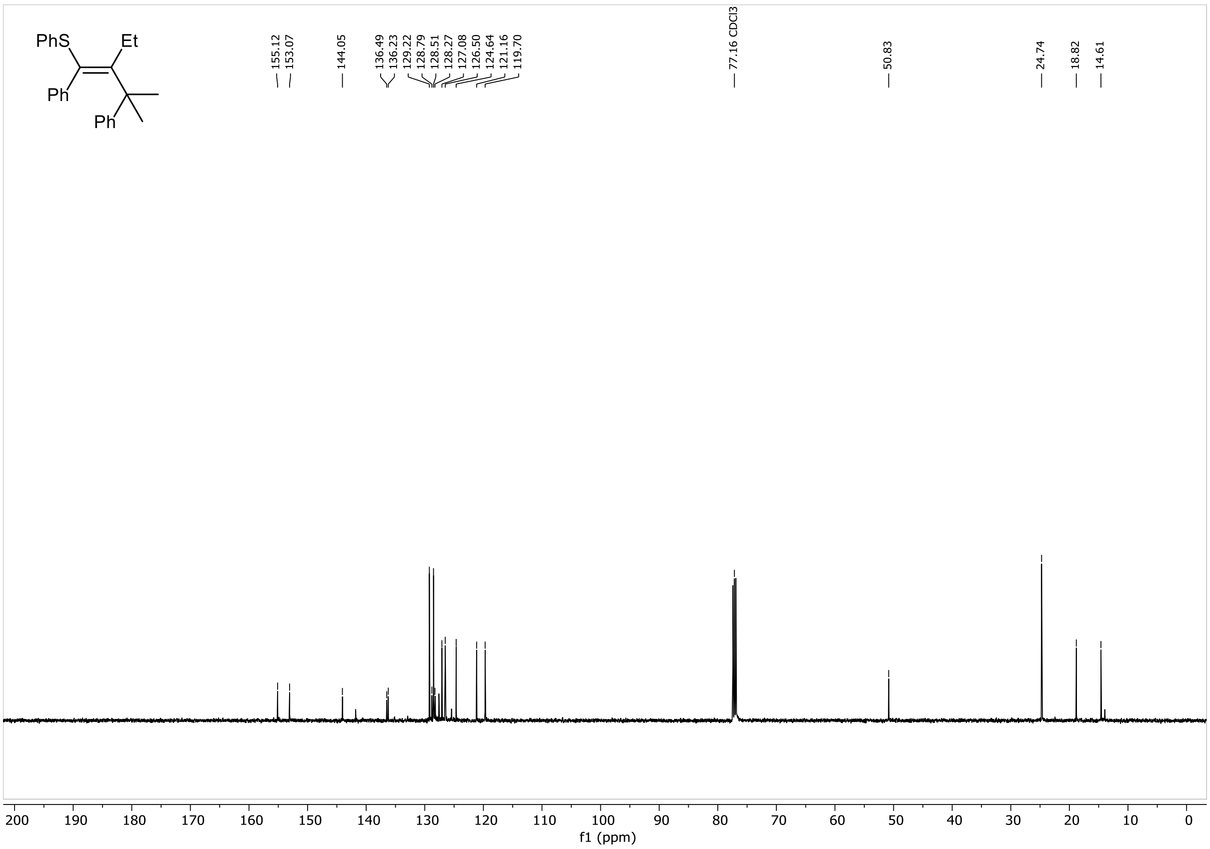

Figure S67. ^{1}H NMR spectrum (500 MHz, $CDCl_3$, 298 K) of *(E)*-(2-ethyl-3,3-dimethyl-1-phenylbut-1-en-1-yl)(methyl)sulfane (**3sa**)

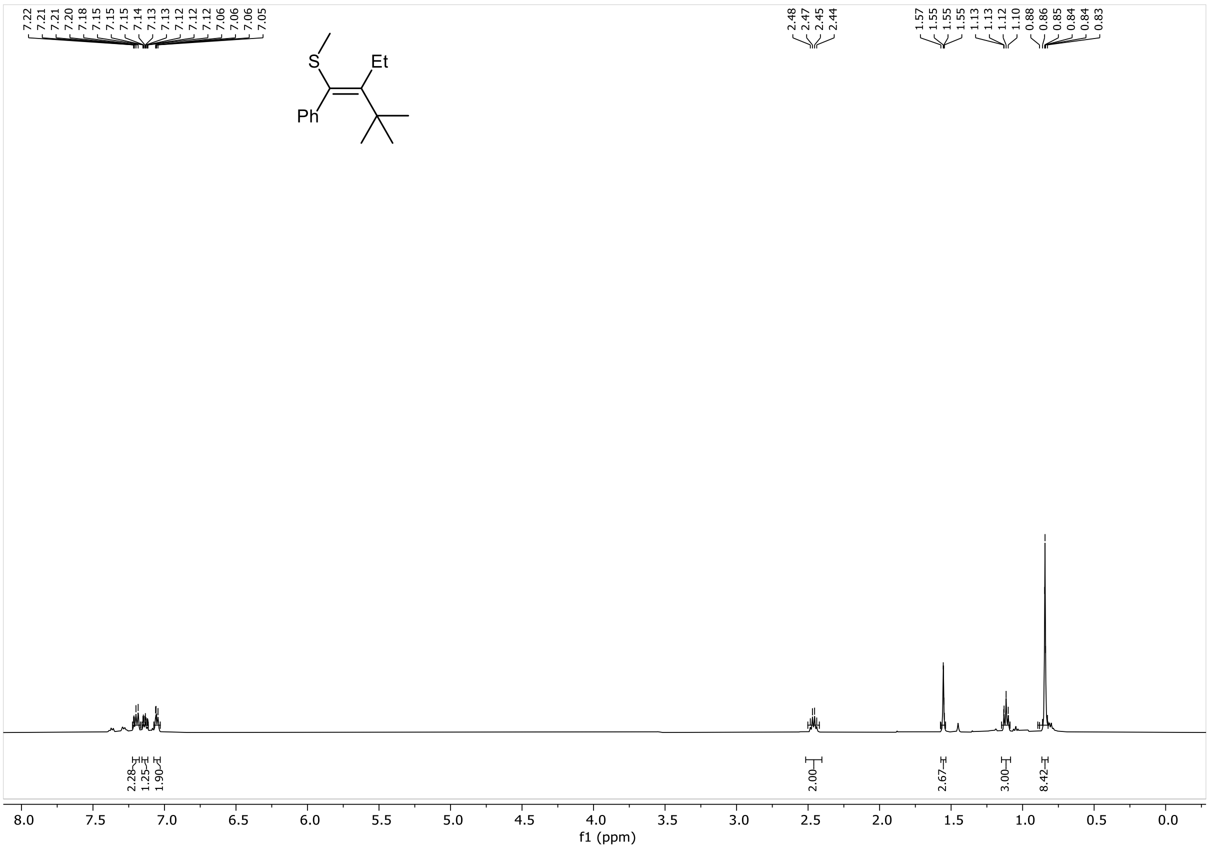

Figure S68. $^{13}C\{^{1}H\}$ NMR spectrum (126 MHz, $CDCl_3$, 298 K) of **3sa**

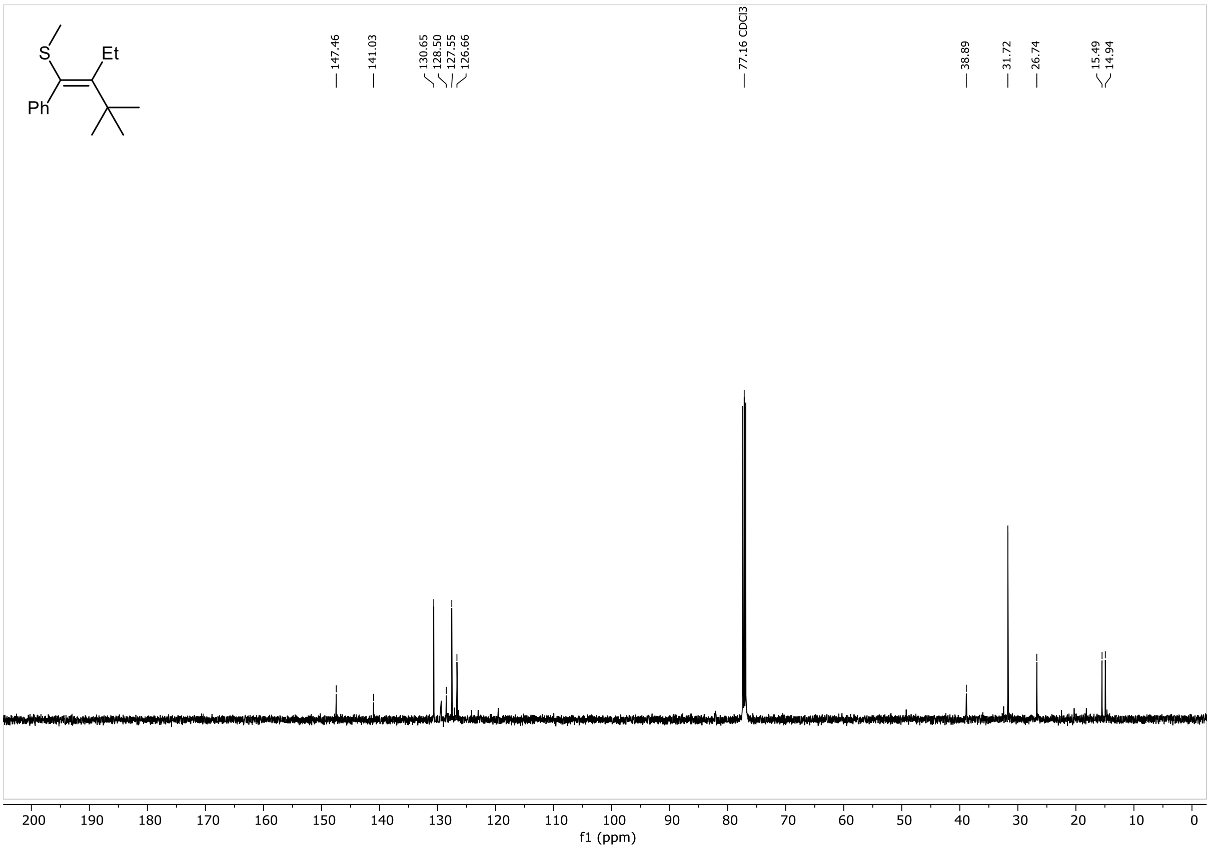

Figure S69. ^{1}H NMR spectrum (500 MHz, $CDCl_3$, 298 K) of *(E)*-phenyl(2,3,3-trimethyl-1-phenylbut-1-en-1-yl)sulfane (**3ab**)

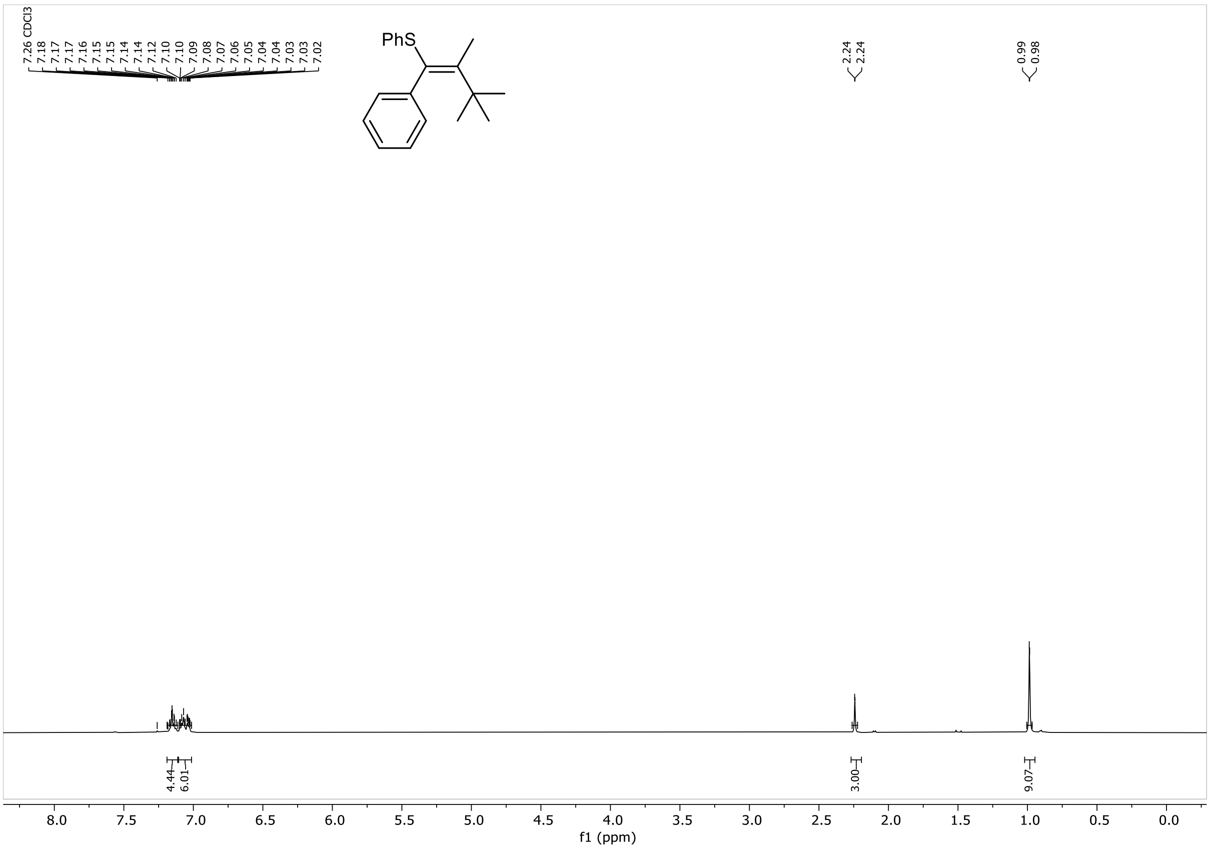

Figure S70. $^{13}C\{^{1}H\}$ NMR spectrum (126 MHz, $CDCl_3$, 298 K) of **3ab**

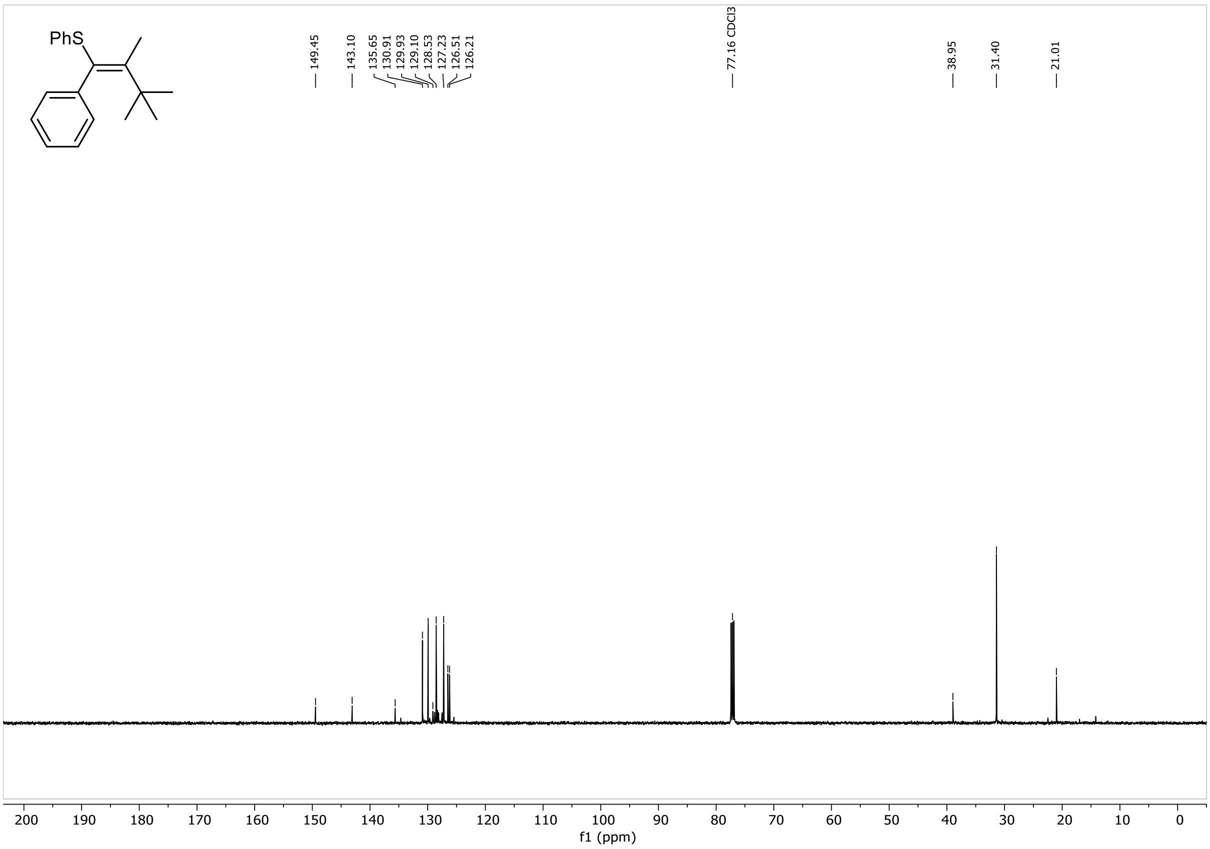

Figure S71. ^{1}H NMR spectrum (500 MHz, $CDCl_3$, 298 K) of *(E)*-phenyl(2,3,3-trimethyl-1-(p-tolyl)but-1-en-1-yl)sulfane (**3ac**)

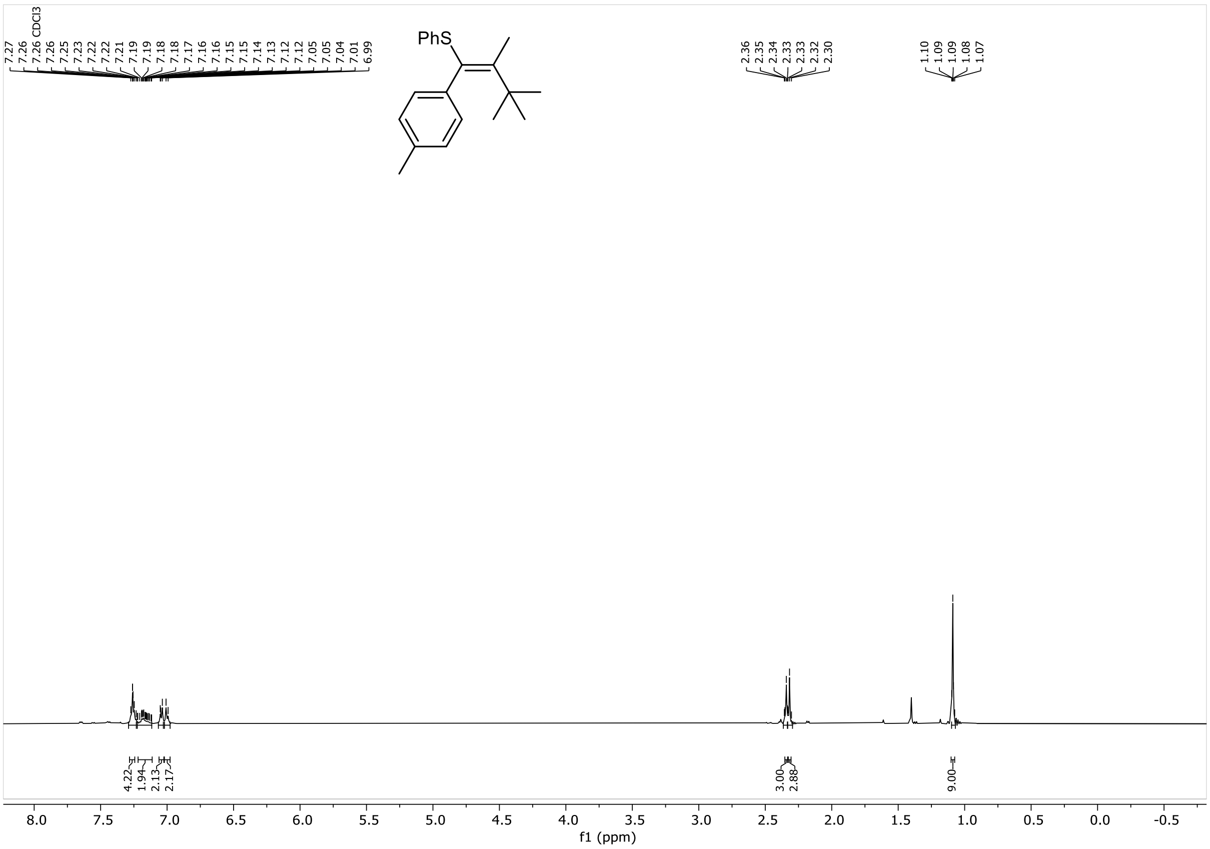

Figure S72. $^{13}C\{^{1}H\}$ NMR spectrum (126 MHz, $CDCl_3$, 298 K) of **3ac**

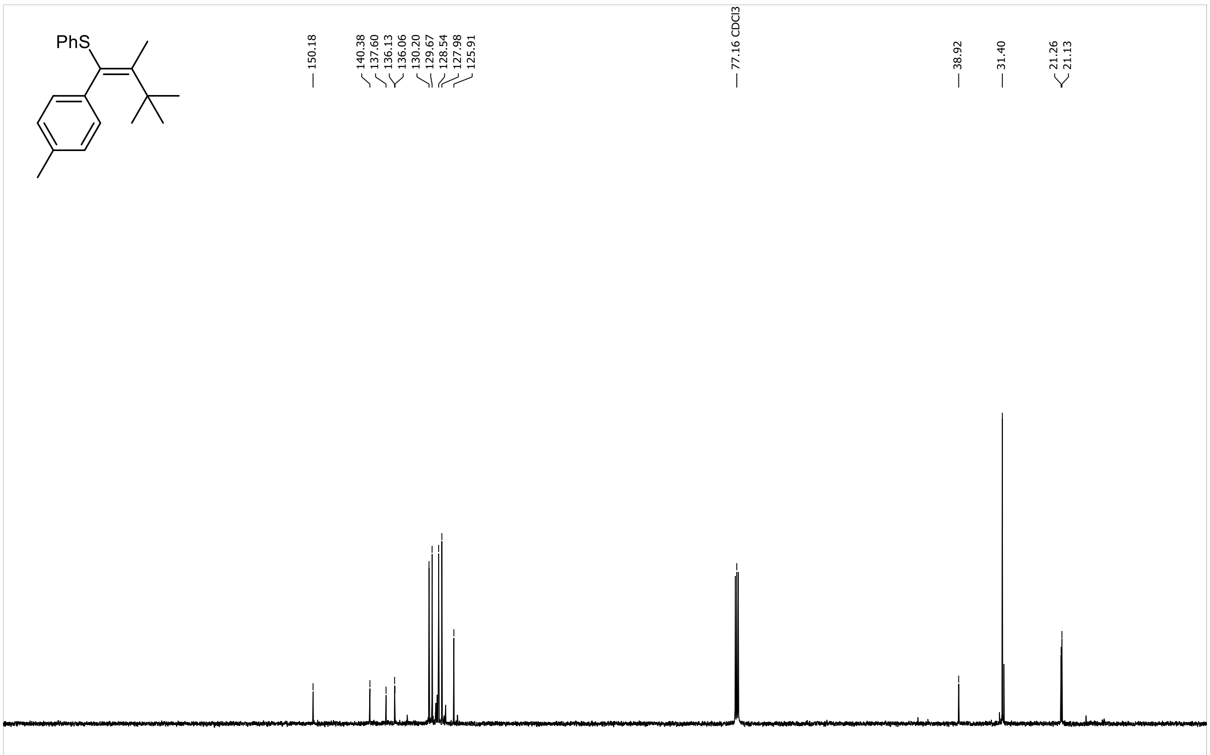

Figure S73. ^{1}H NMR spectrum (500 MHz, $CDCl_3$, 298 K) of *(E)*-(1-(4-fluorophenyl)-2,3,3-trimethylbut-1-en-1-yl)(phenyl)sulfane (**3ad**)

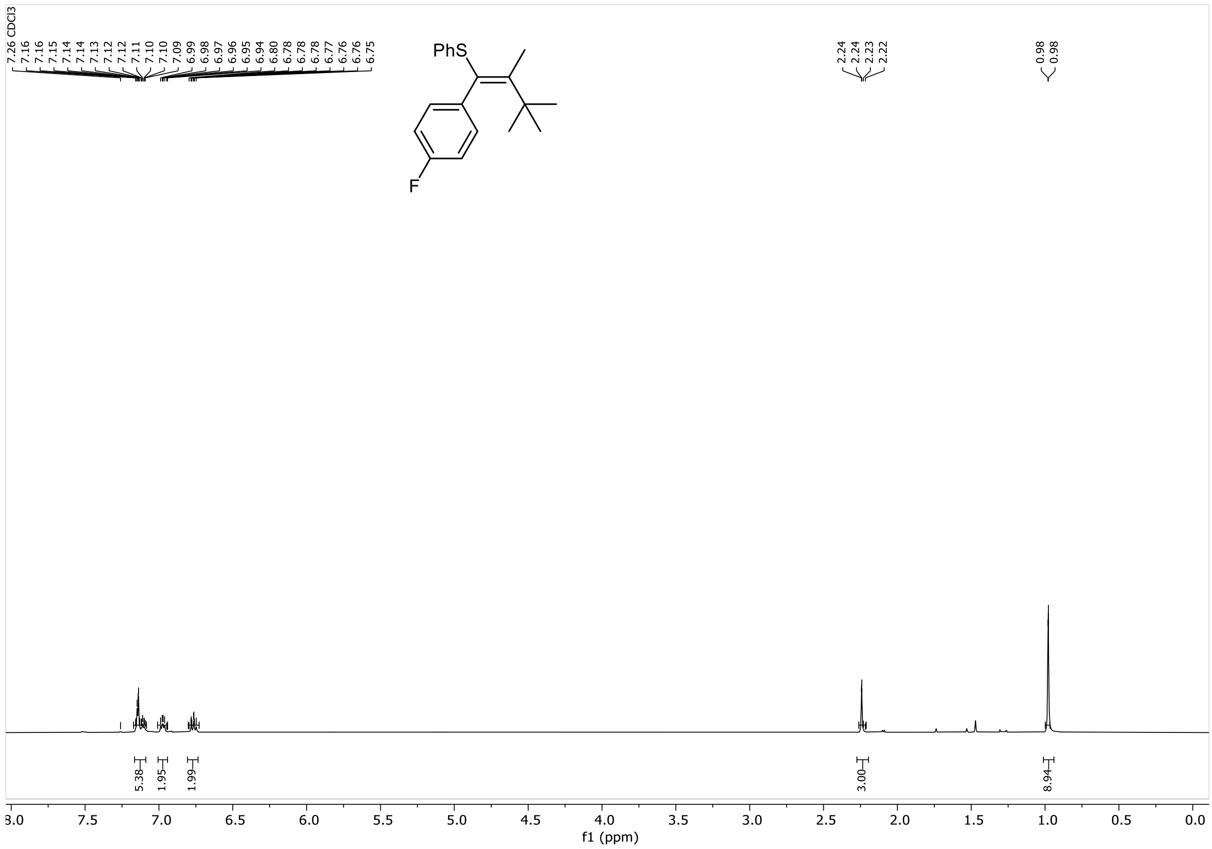

Figure S74. $^{13}C\{^{1}H\}$ NMR spectrum (126 MHz, $CDCl_3$, 298 K) of **3ad**

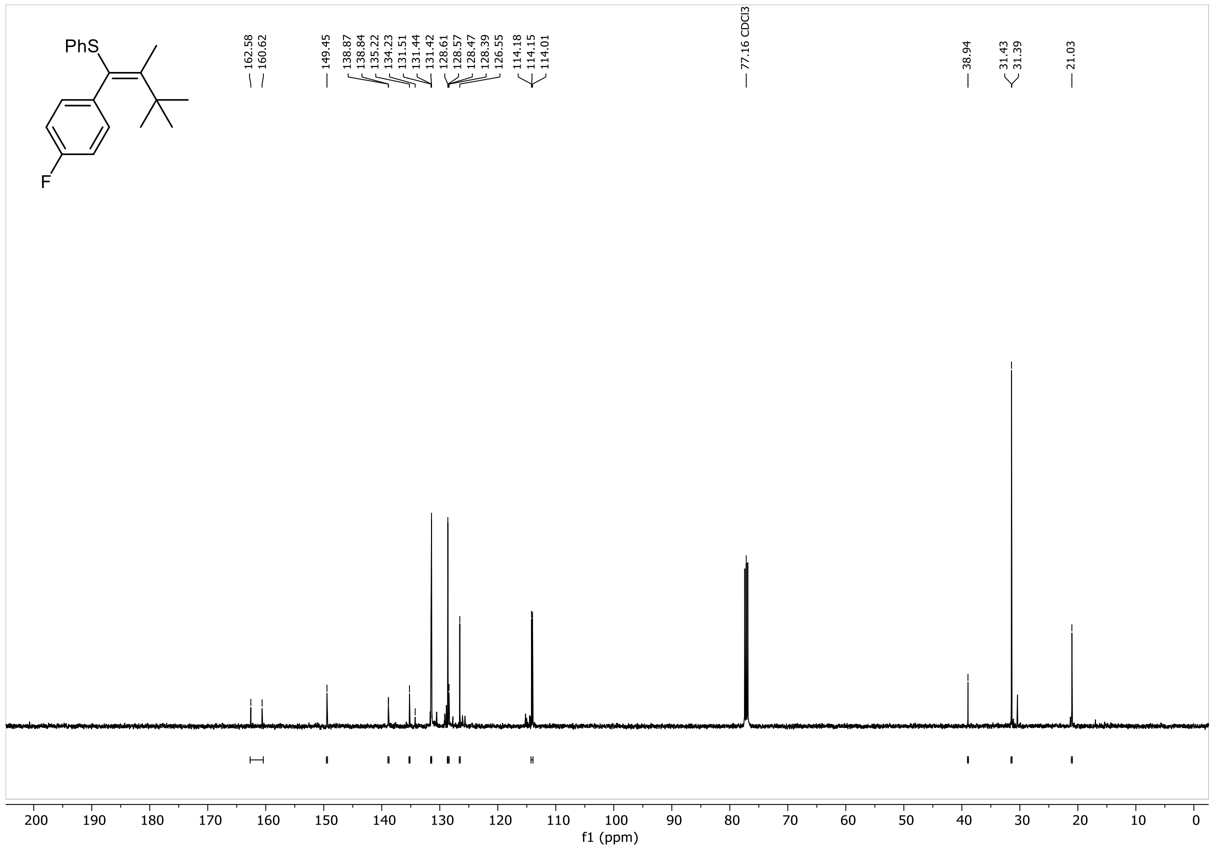

Figure S75. ^{1}H NMR spectrum (500 MHz, $CDCl_3$, 298 K) of *(E)*-(1-(4-Chlorophenyl)-2,3,3-trimethylbut-1-en-1-yl)(phenyl)sulfane (**3ae**)

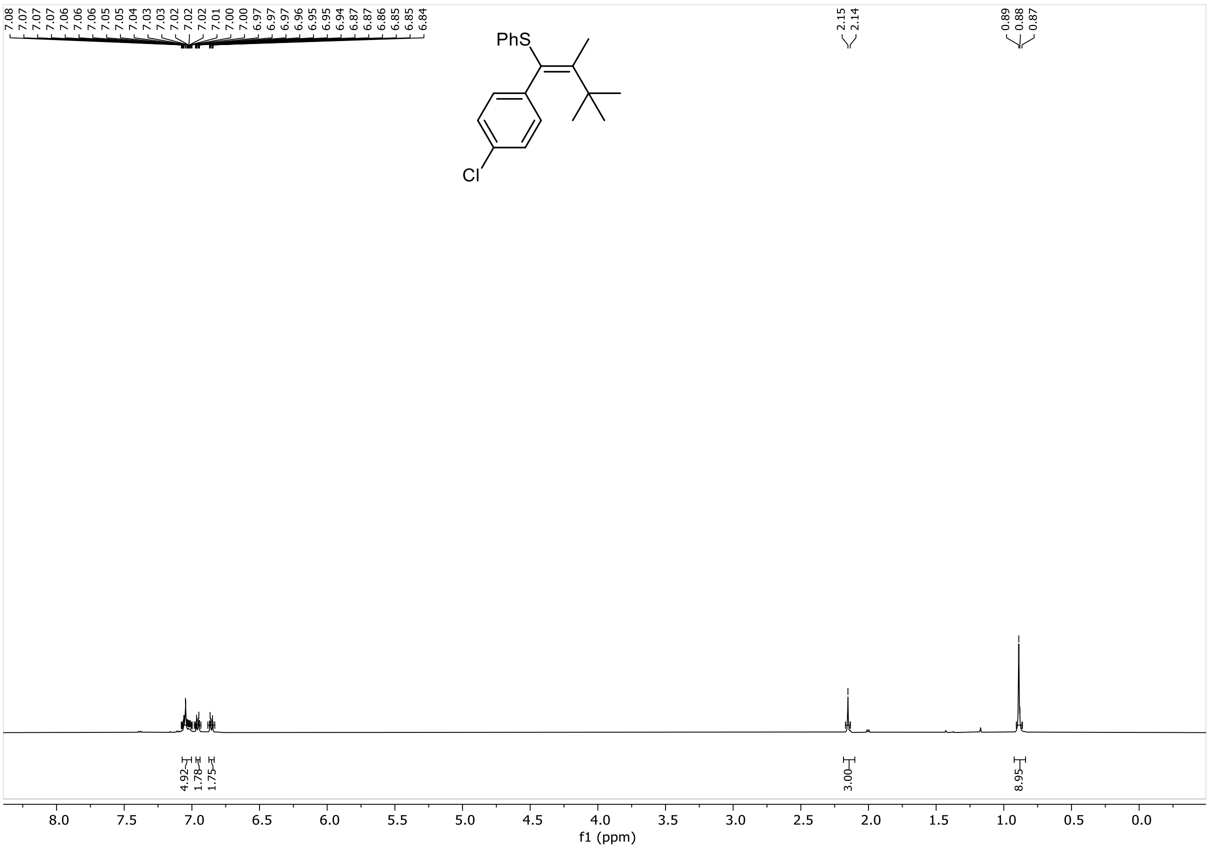

Figure S76. $^{13}C\{^{1}H\}$ NMR spectrum (126 MHz, $CDCl_3$, 298 K) of **3ae**

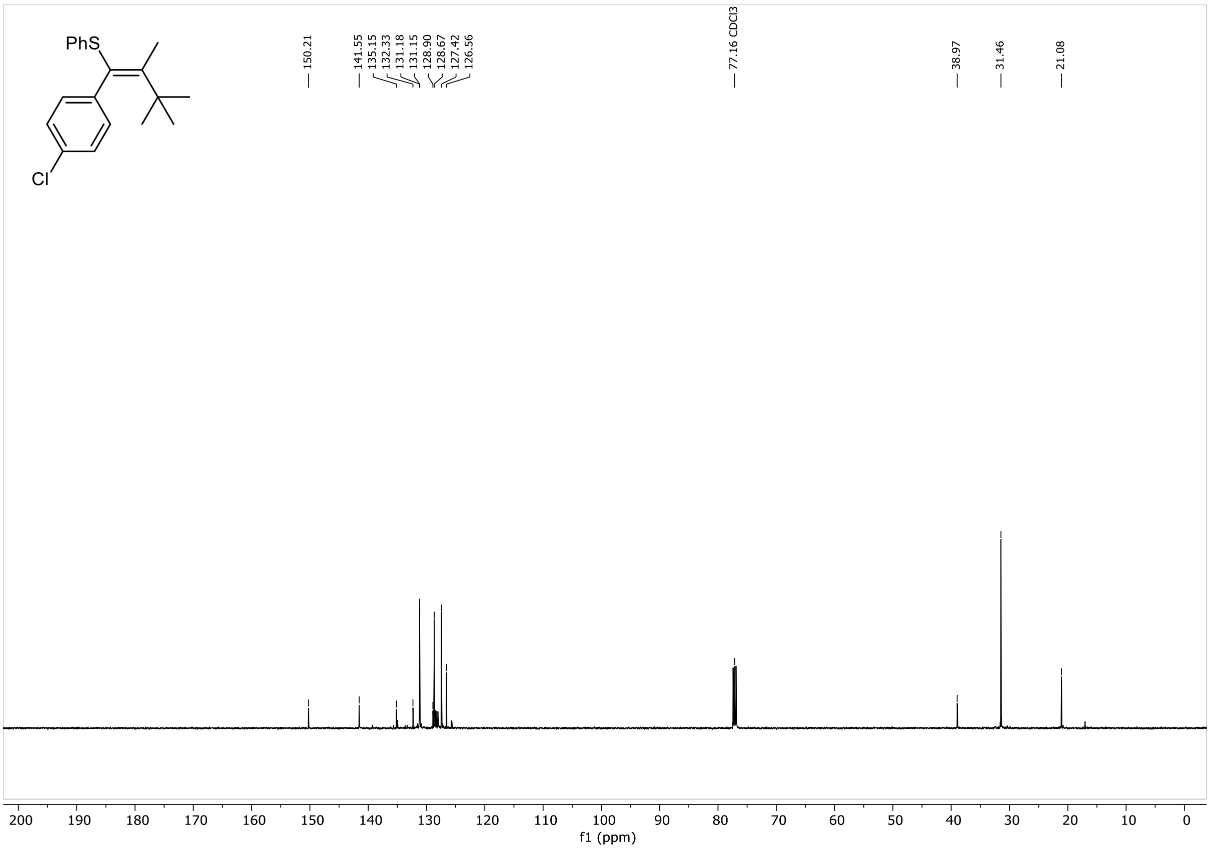

Figure S77. ^{1}H NMR spectrum (500 MHz, $CDCl_3$, 298 K) of *(E)*-(1-(4-bromophenyl)-2,3,3-trimethylbut-1-en-1-yl)(phenyl)sulfane (**3af**)

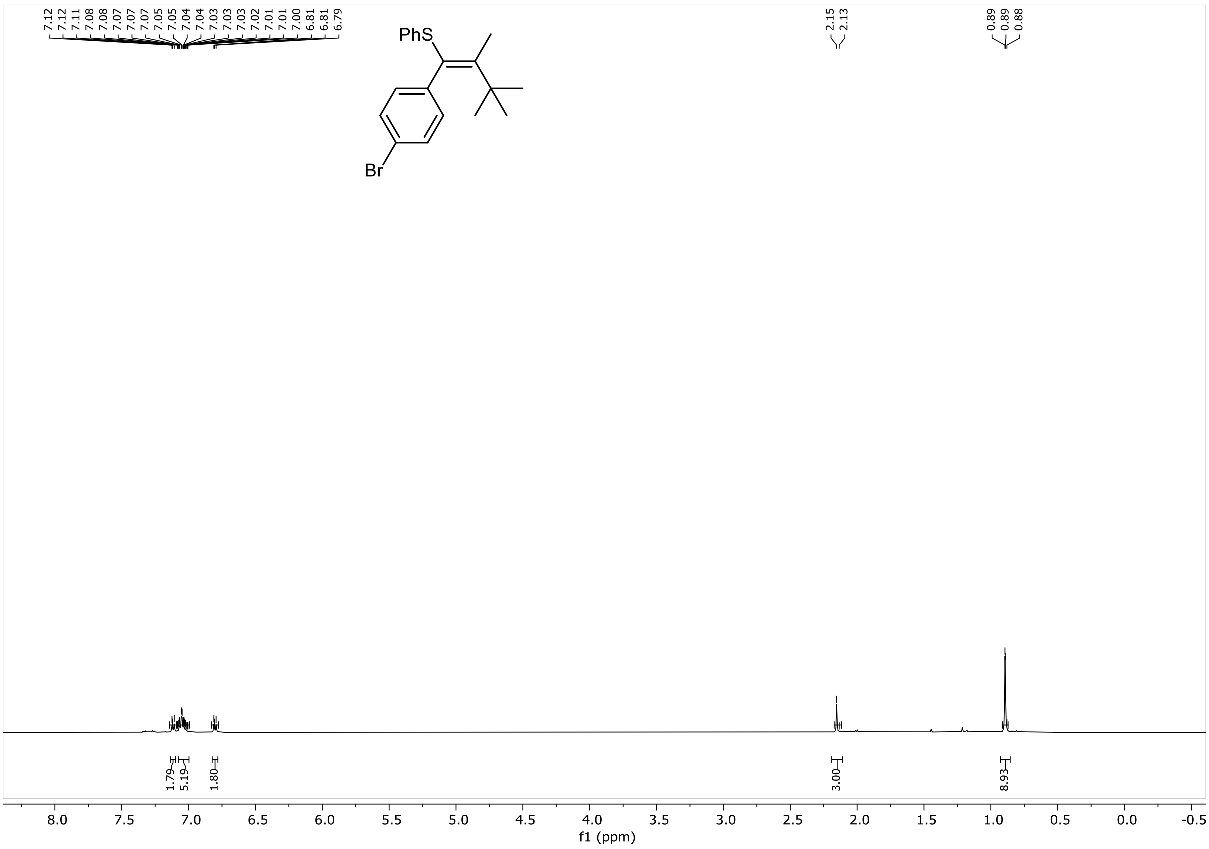

Figure S78. $^{13}C\{^{1}H\}$ NMR spectrum (126 MHz, $CDCl_3$, 298 K) of **3af**

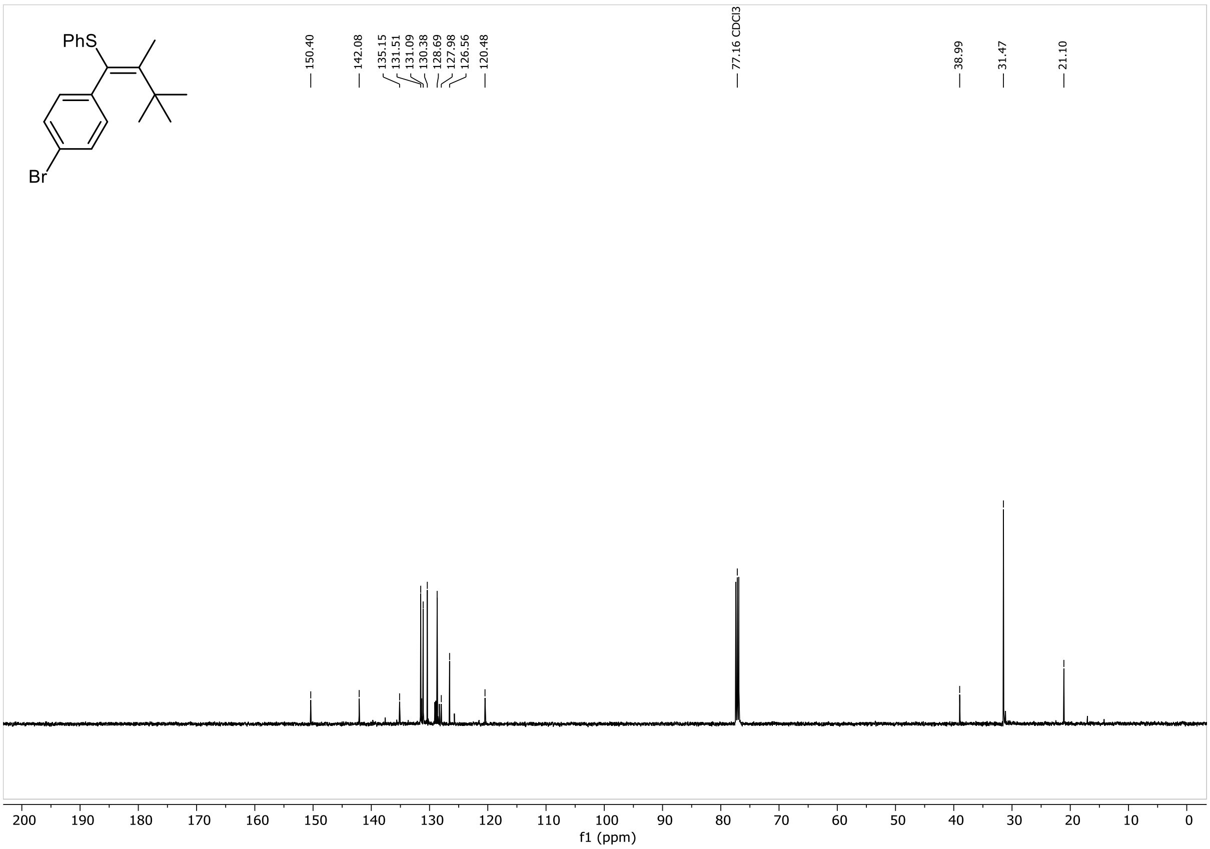

Figure S79. ^{1}H NMR spectrum (500 MHz, $CDCl_3$, 298 K) of *(E)*-(1-(4-(*tert*-butyl)phenyl)-2,3,3-trimethylbut-1-en-1-yl)(phenyl)sulfane (**3ag**)

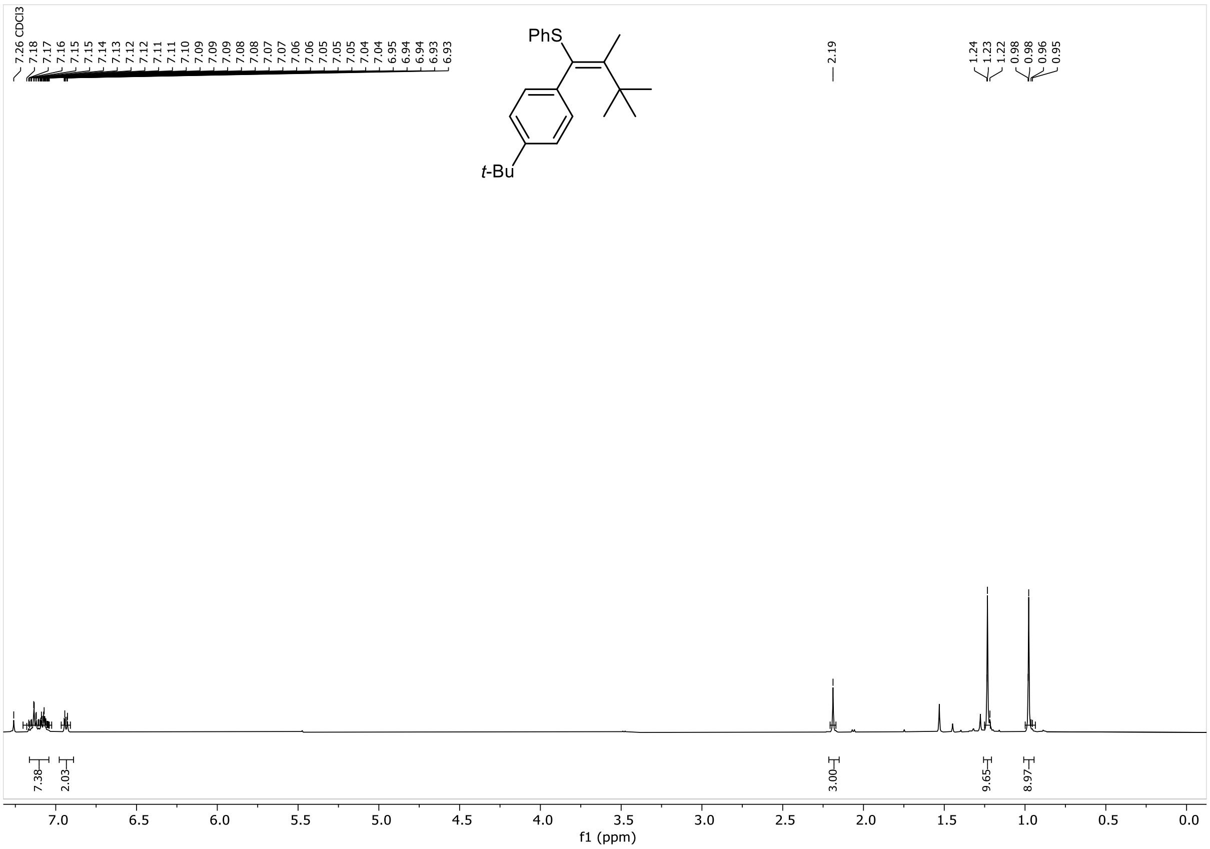

Figure S80. $^{13}C\{^{1}H\}$ NMR spectrum (126 MHz, $CDCl_3$, 298 K) of **3ag**

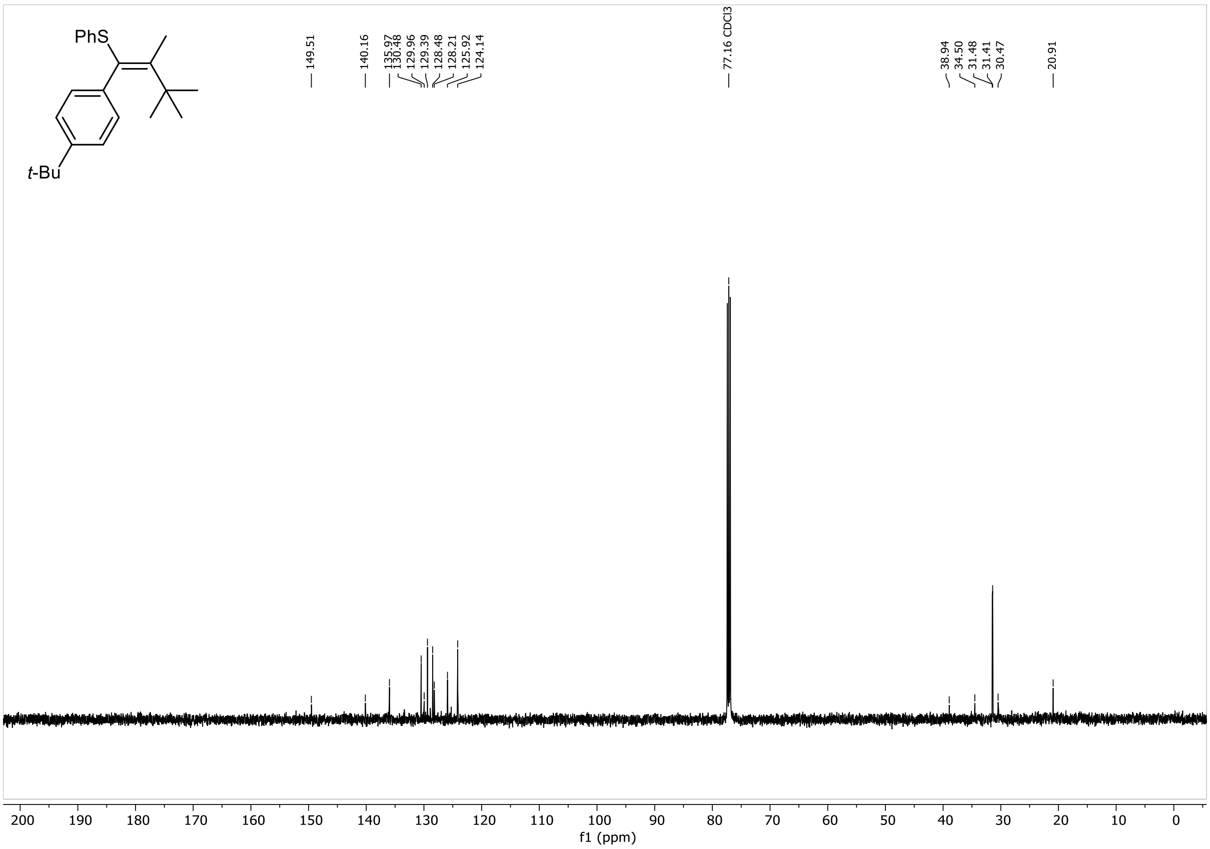

Figure S81. ^{1}H NMR spectrum (500 MHz, $CDCl_3$, 298 K) of *(E)*-phenyl(2,3,3-trimethyl-1-(*m*-tolyl)but-1-en-1-yl)sulfane (**3ah**)

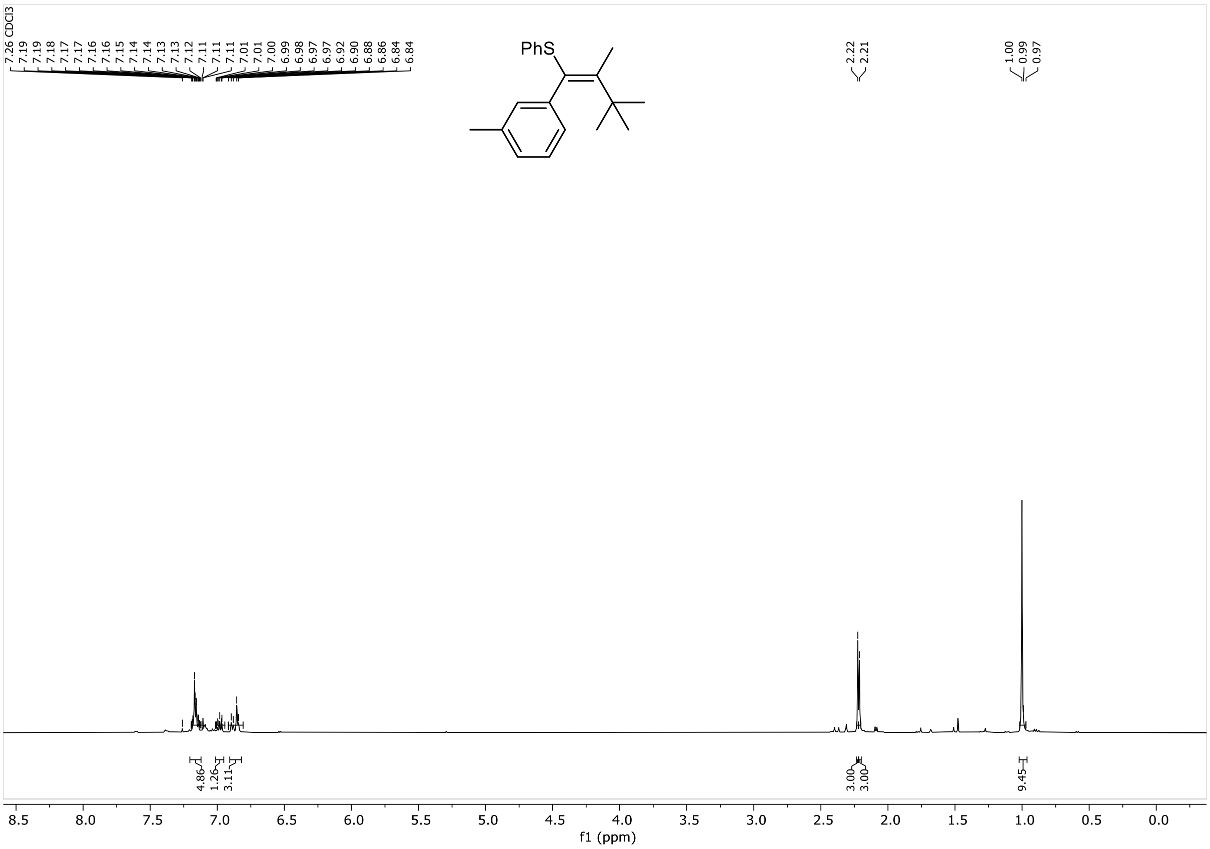

Figure S82. $^{13}C\{^{1}H\}$ NMR spectrum (126 MHz, $CDCl_3$, 298 K) of **3ah**

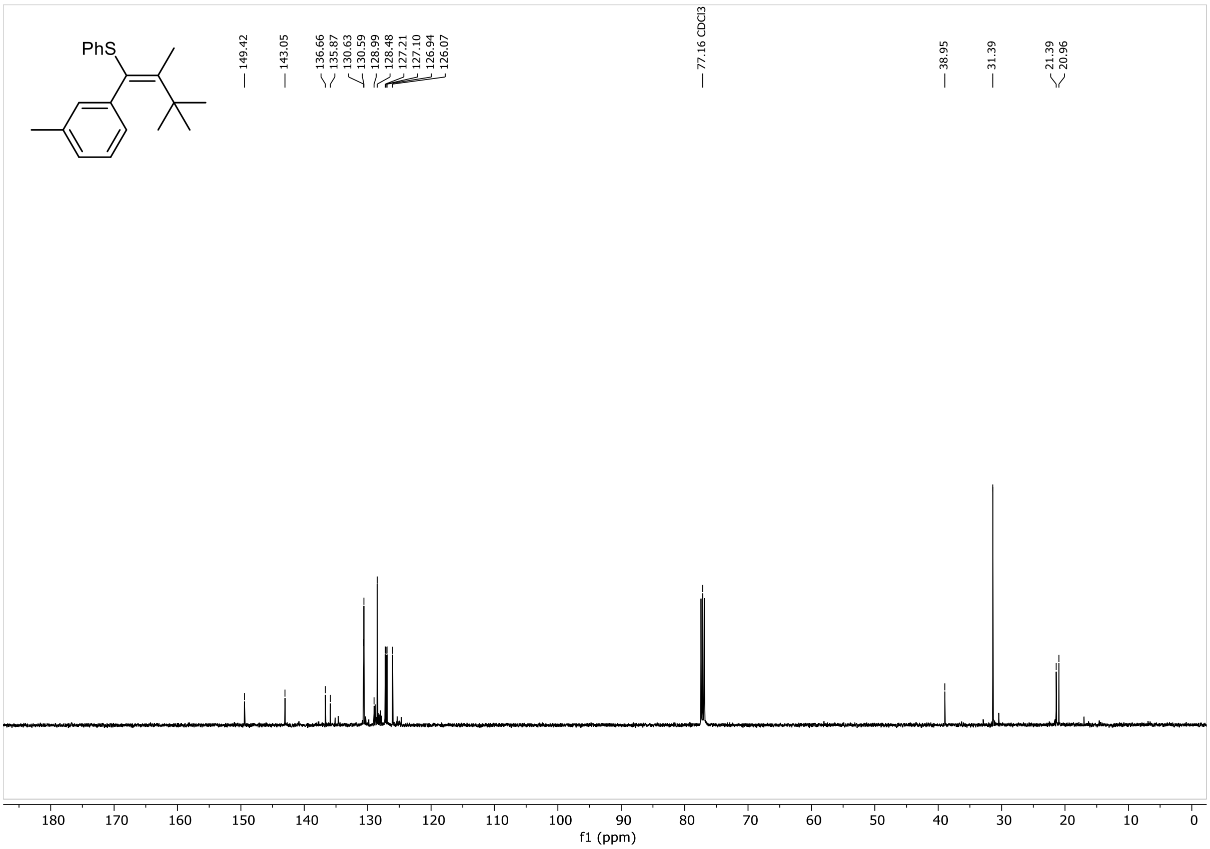

Figure S83. ^{1}H NMR spectrum (500 MHz, $CDCl_3$, 298 K) of *(E)*-(1-(3-fluorophenyl)-2,3,3-trimethylbut-1-en-1-yl)(phenyl)sulfane (**3al**)

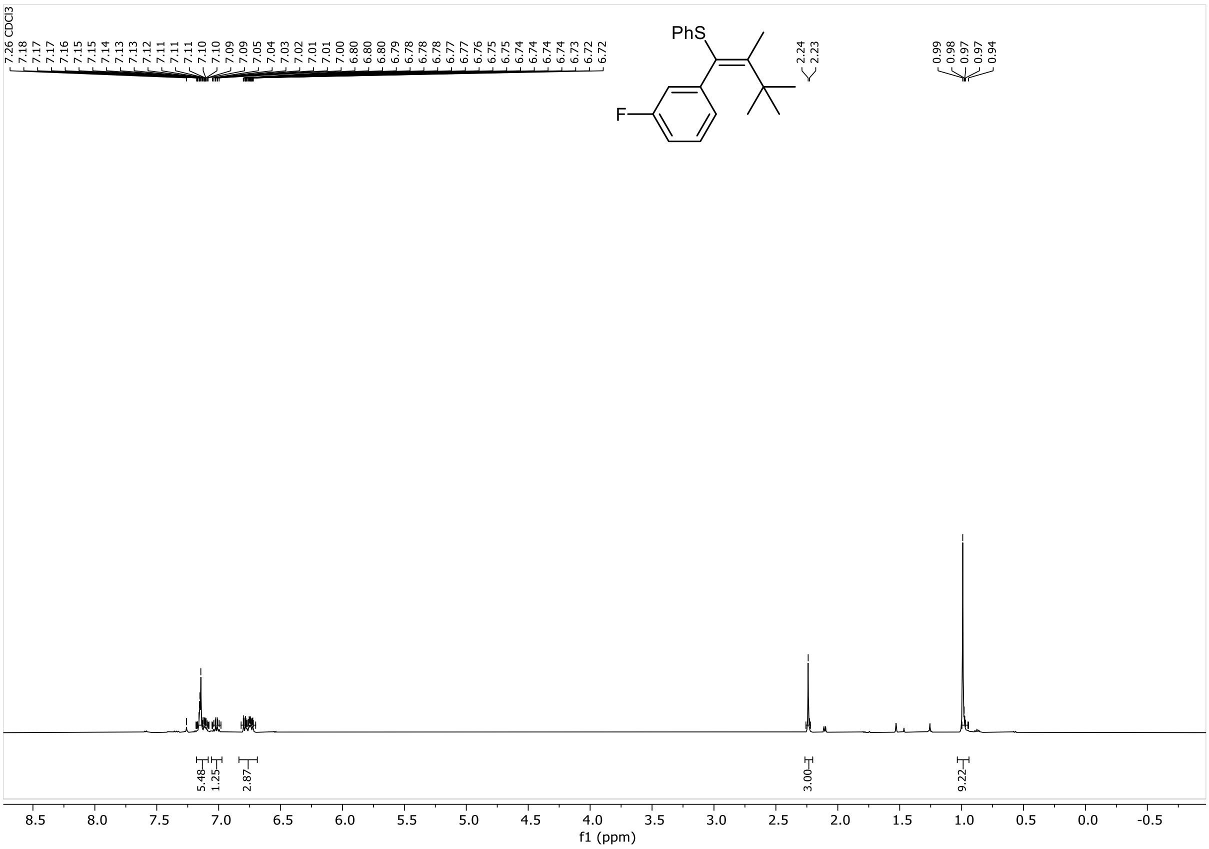

Figure S84. ^{13}C{^{1}H} NMR spectrum (126 MHz, $CDCl_3$, 298 K) of **3ai**

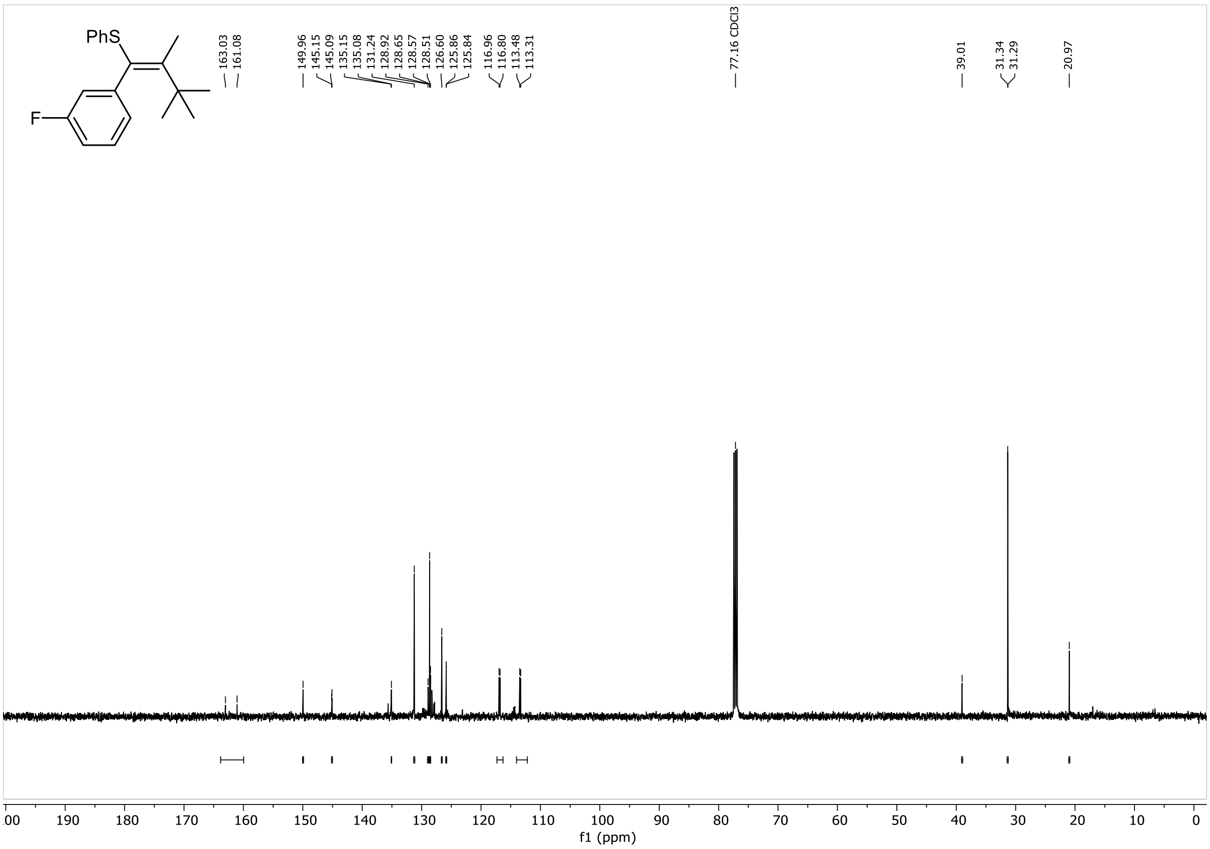

Figure S85. ^{1}H NMR spectrum (500 MHz, $CDCl_3$, 298 K) of *(E)*-(1-(3-chlorophenyl)-2,3,3-trimethylbut-1-en-1-yl)(phenyl)sulfane (**3aj**)

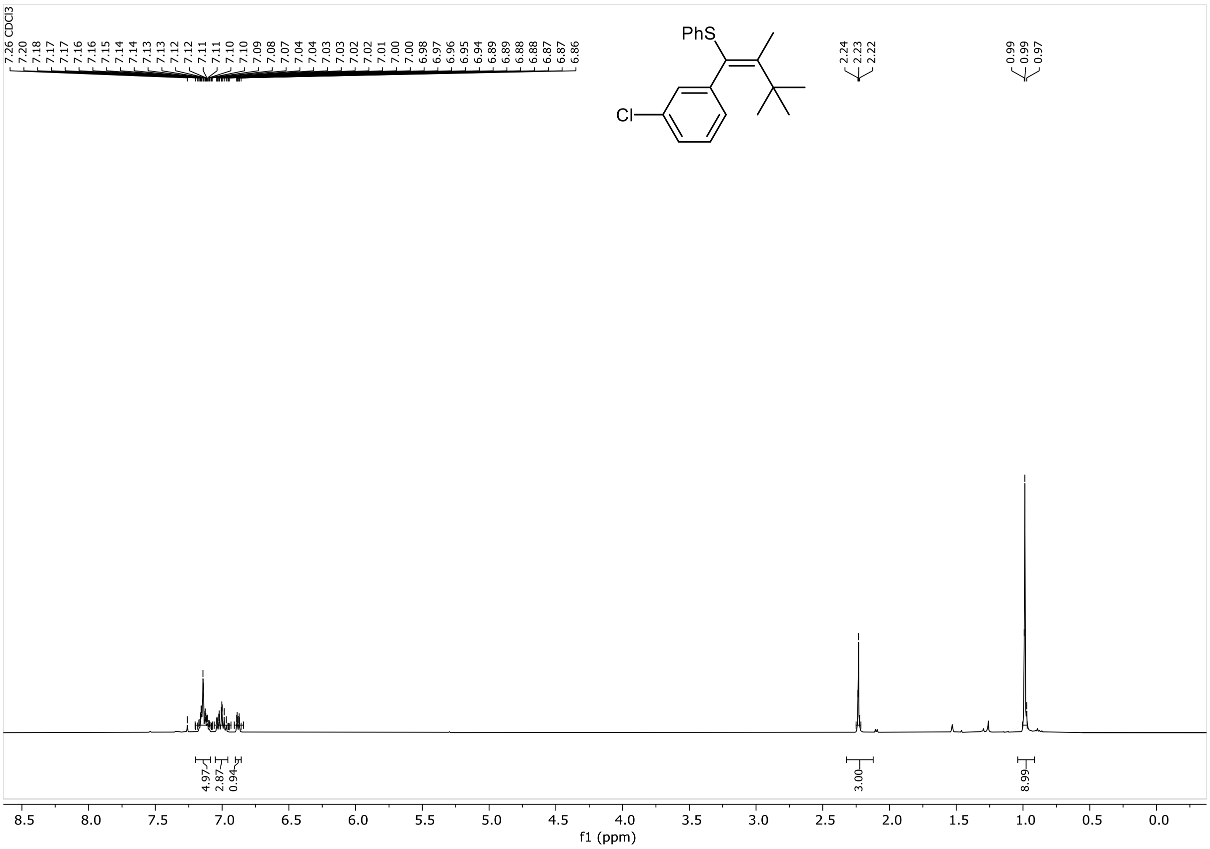

Figure S86. $^{13}C\{^{1}H\}$ NMR spectrum (126 MHz, $CDCl_3$, 298 K) of **3aj**

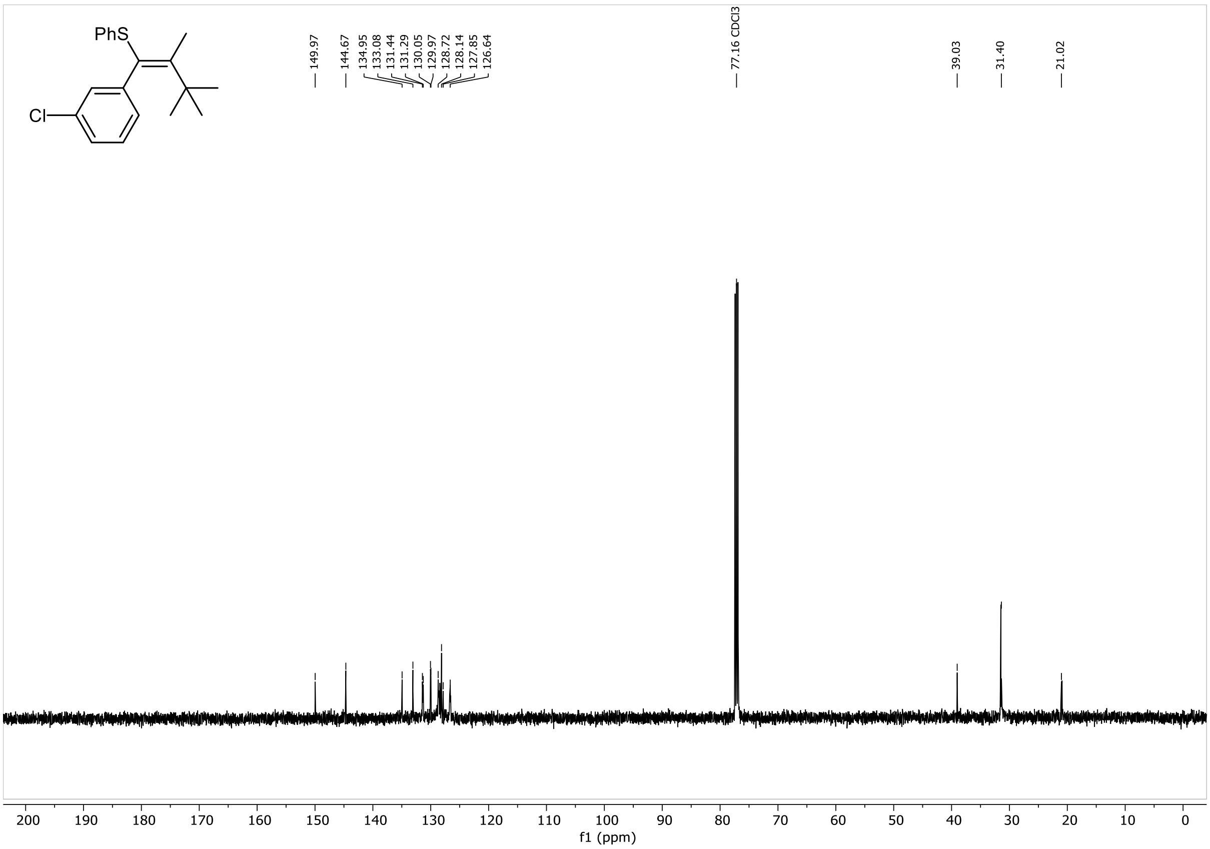

Figure S87. ^1H NMR spectrum (500 MHz, $CDCl_3$, 298 K) of *(E)*-(1-(3-bromophenyl)-2,3,3-trimethylbut-1-en-1-yl)(phenyl)sulfane (**3ak**)

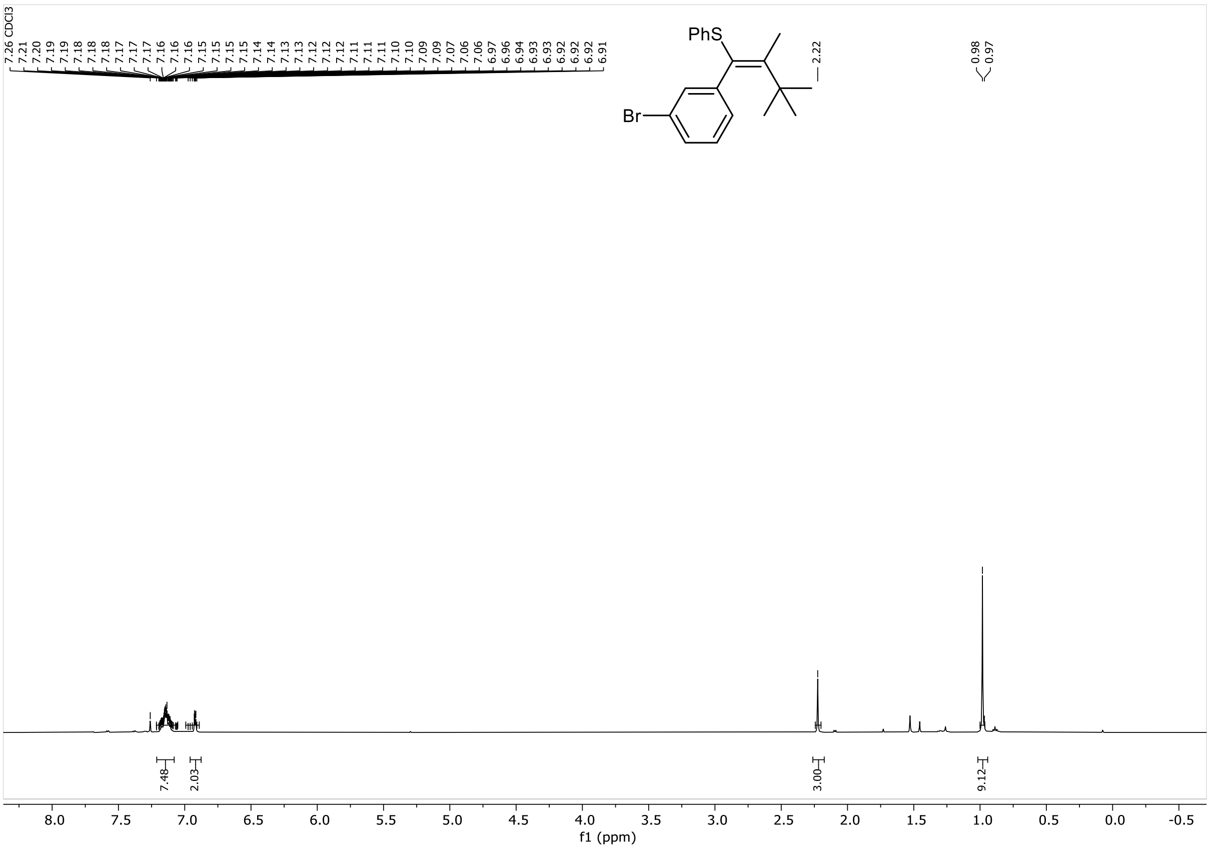

Figure S88. $^{13}C\{^1H\}$ NMR spectrum (126 MHz, $CDCl_3$, 298 K) of **3ak**

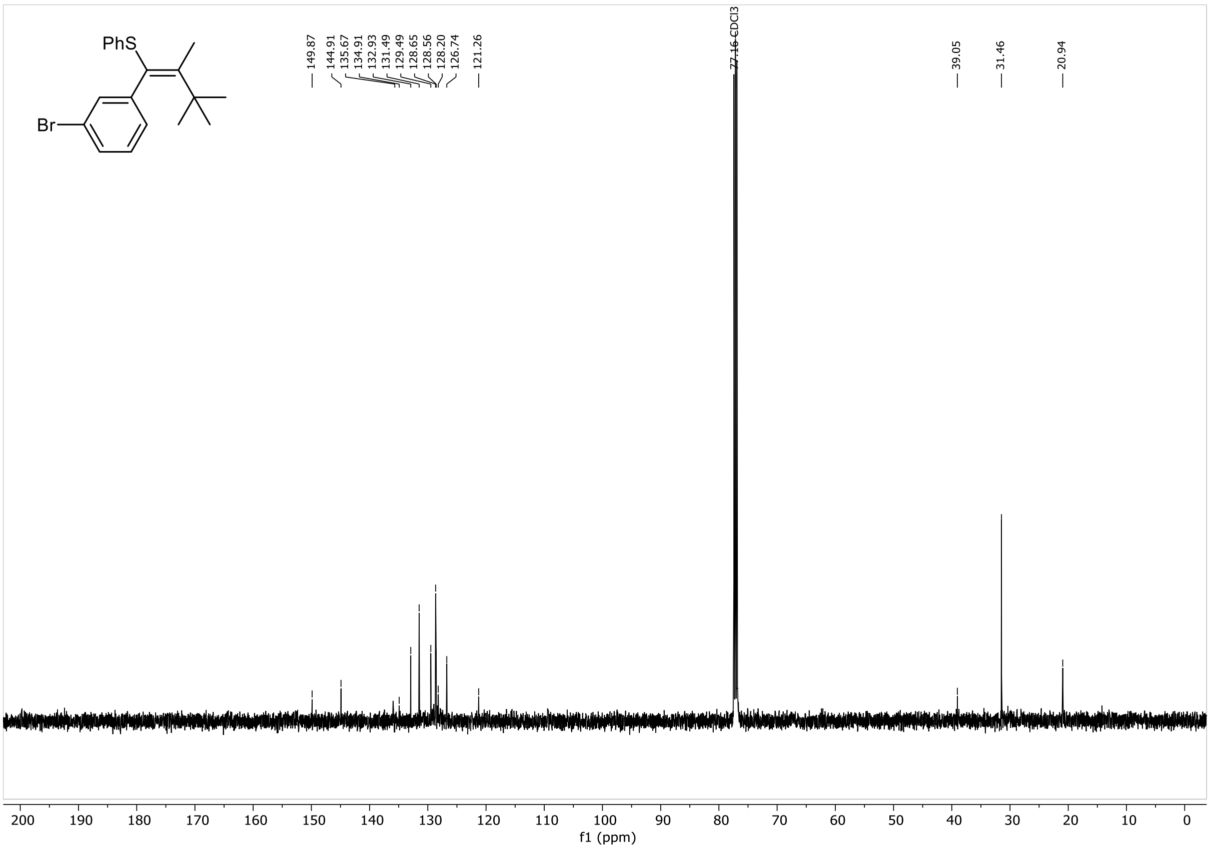

Figure S89. ^{1}H NMR spectrum (500 MHz, $CDCl_3$, 298 K) of *(E)*-phenyl(2,3,3-trimethyl-1-(*o*-tolyl)but-1-en-1-yl)sulfane (**3al**)

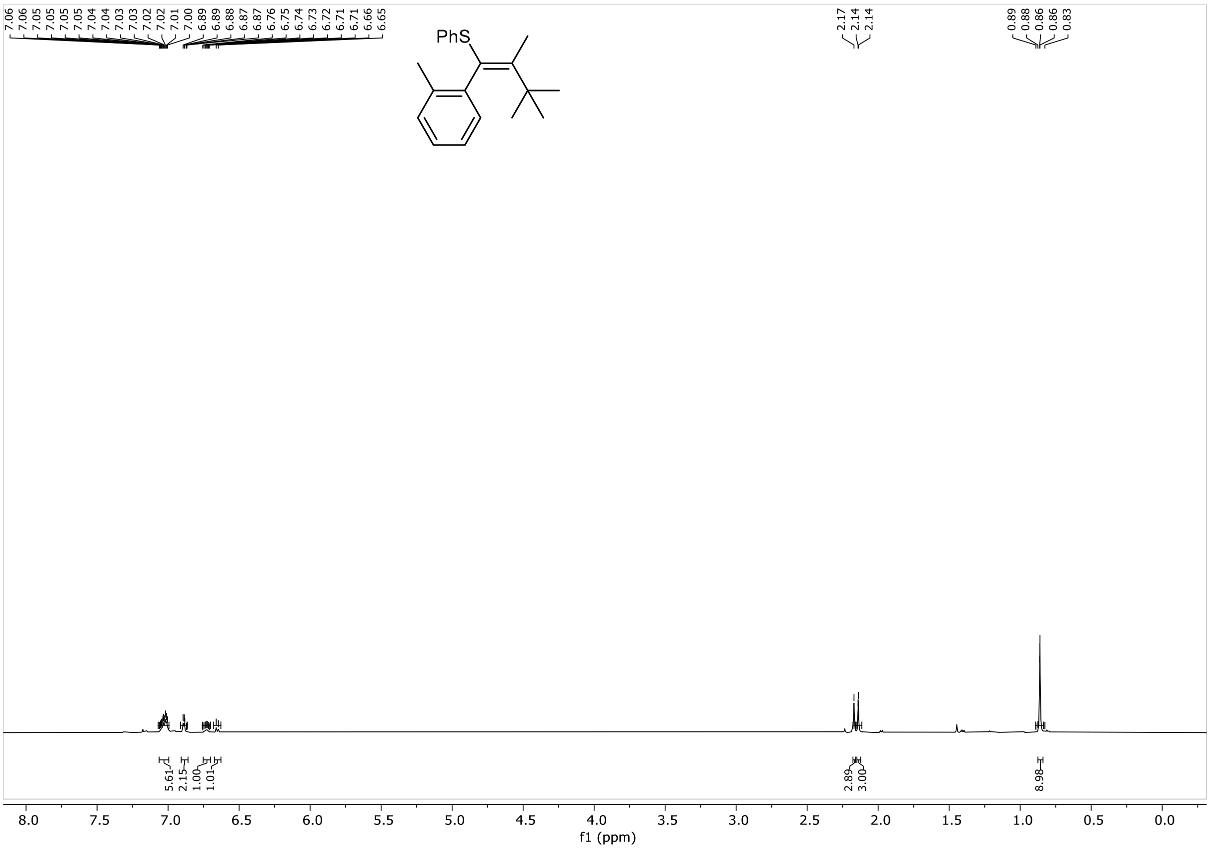

Figure S90. $^{13}C\{^{1}H\}$ NMR spectrum (126 MHz, $CDCl_3$, 298 K) of **3al**

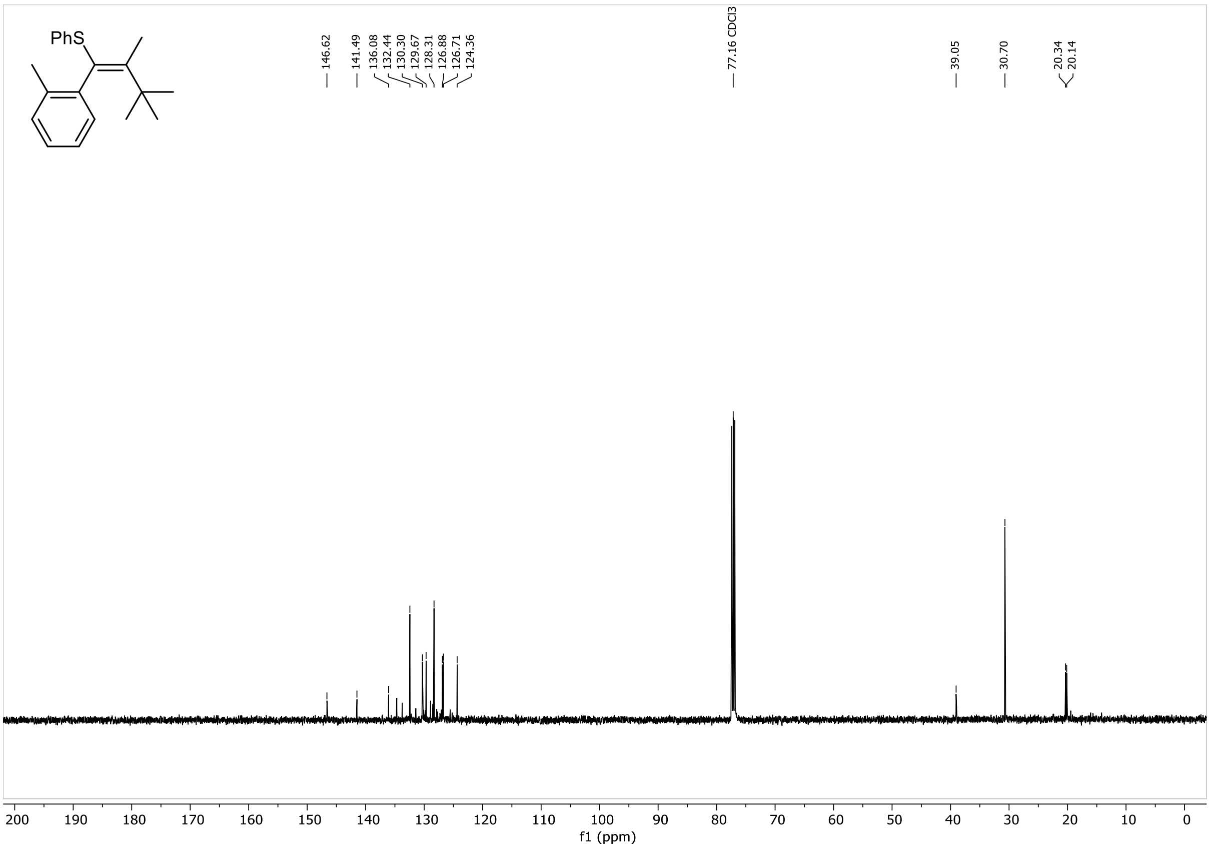

Figure S91. ^{1}H NMR spectrum (500 MHz, $CDCl_3$, 298 K) of *(E)*-(1-(2-fluorophenyl)-2,3,3-trimethylbut-1-en-1-yl)(phenyl)sulfane (**3am**)

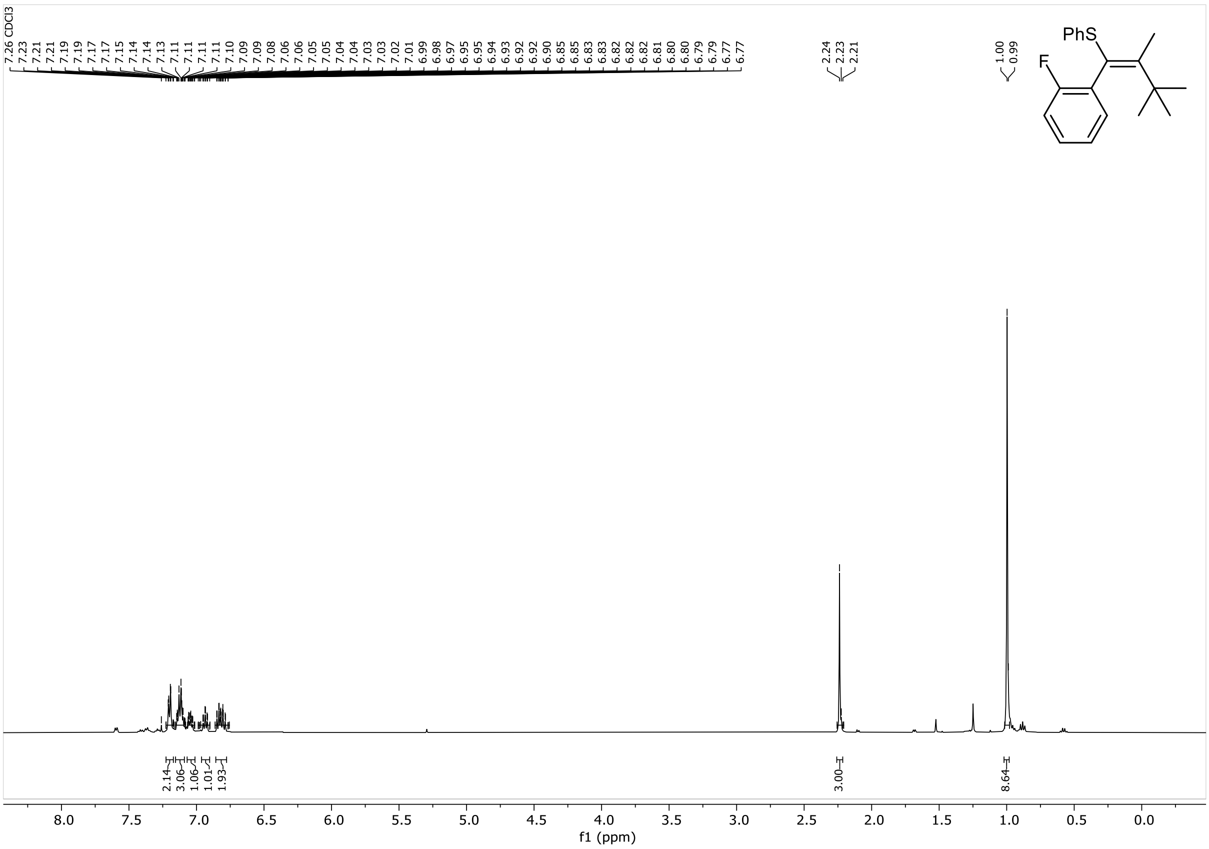

Figure S92. $^{13}C\{^{1}H\}$ NMR spectrum (126 MHz, $CDCl_3$, 298 K) of **3am**

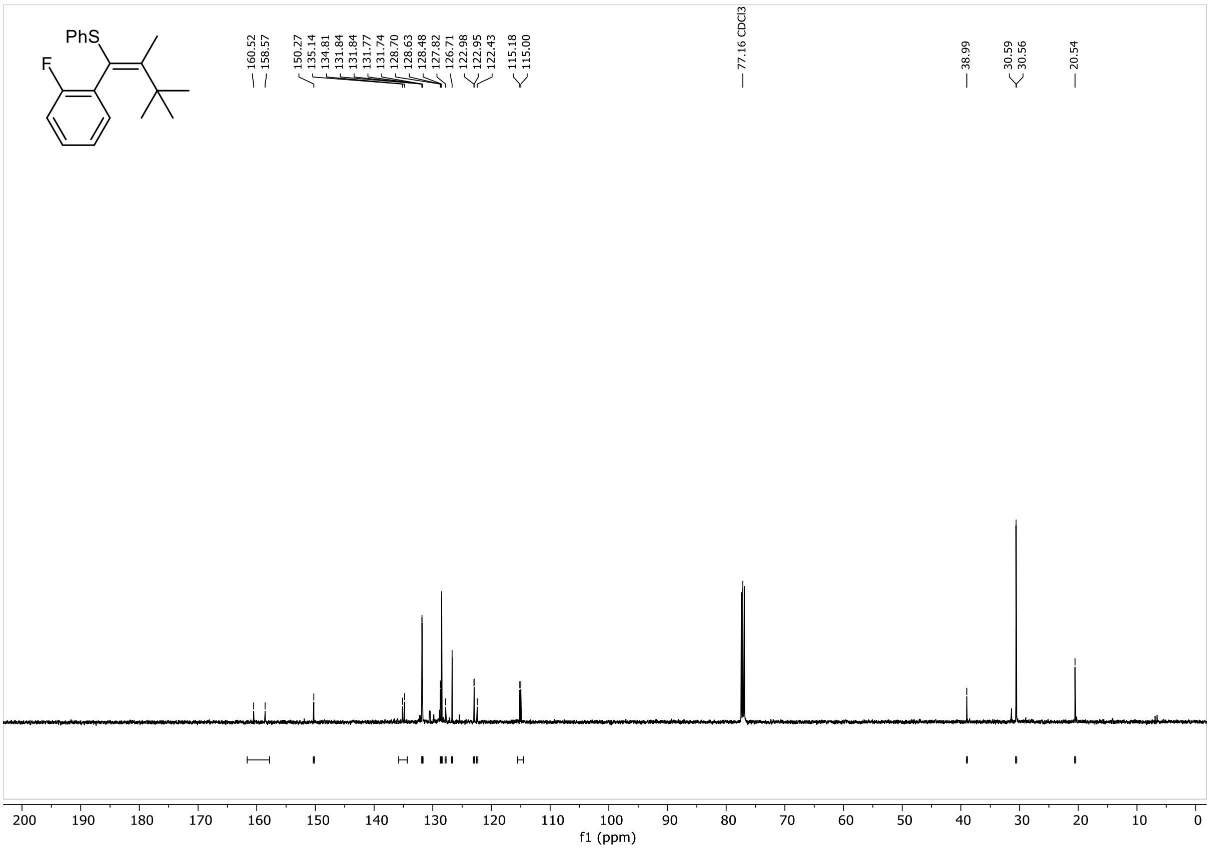

Figure S93. ^{1}H NMR spectrum (500 MHz, $CDCl_3$, 298 K) of *(E)*-(1-(2-chlorophenyl)-2,3,3-trimethylbut-1-en-1-yl)(phenyl)sulfane (**3an**)

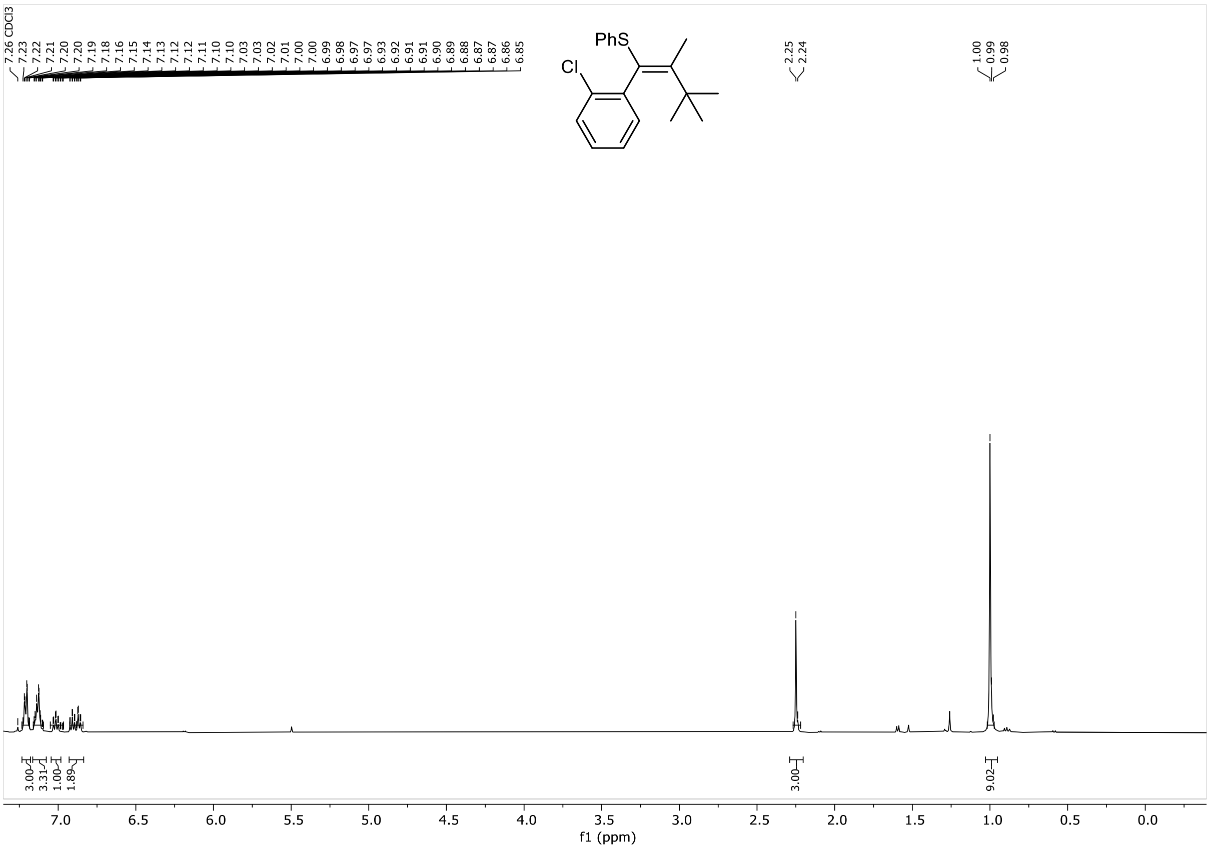

Figure S94. $^{13}C\{^{1}H\}$ NMR spectrum (126 MHz, $CDCl_3$, 298 K) of **3an**

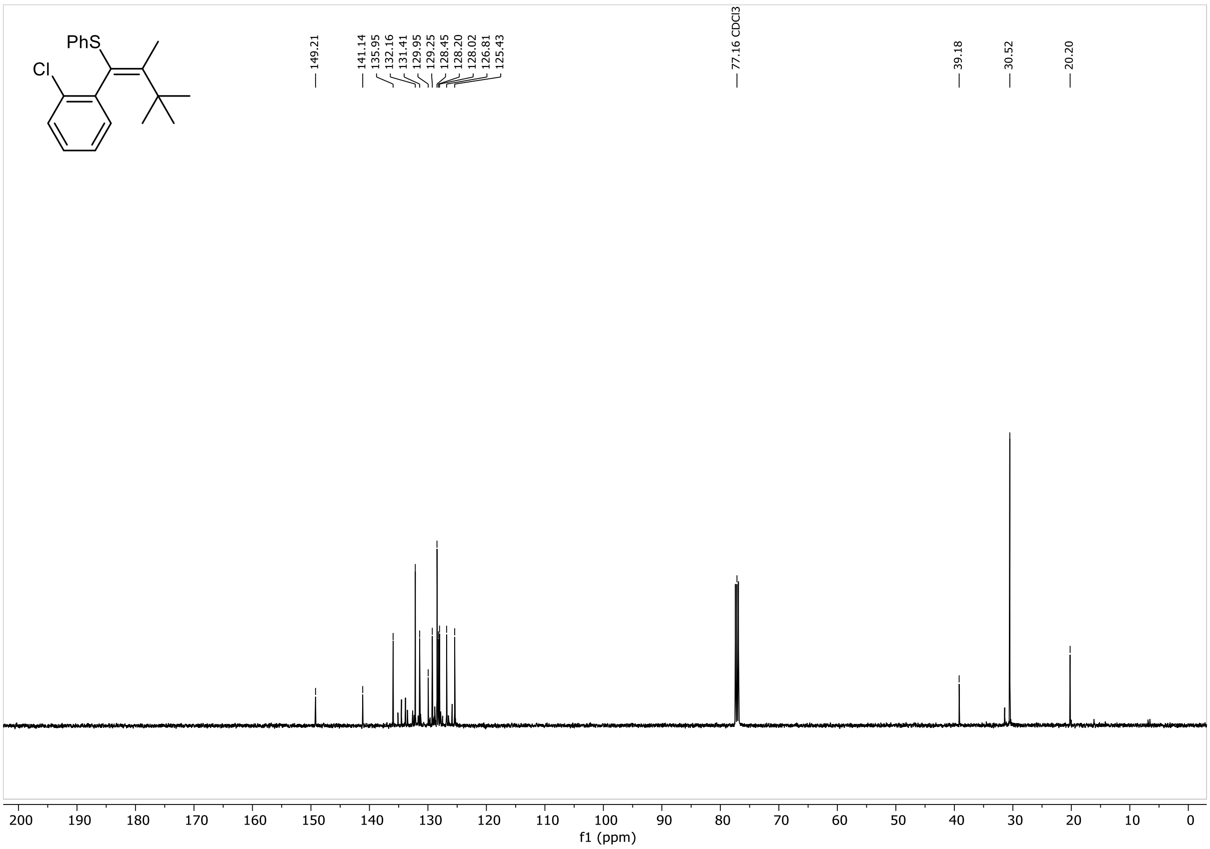

Figure S95. ^{1}H NMR spectrum (500 MHz, $CDCl_3$, 298 K) of (Z)-(3,3-dimethyl-1,2-diphenylbut-1-en-1-yl)(phenyl)sulfane (**3ao**)

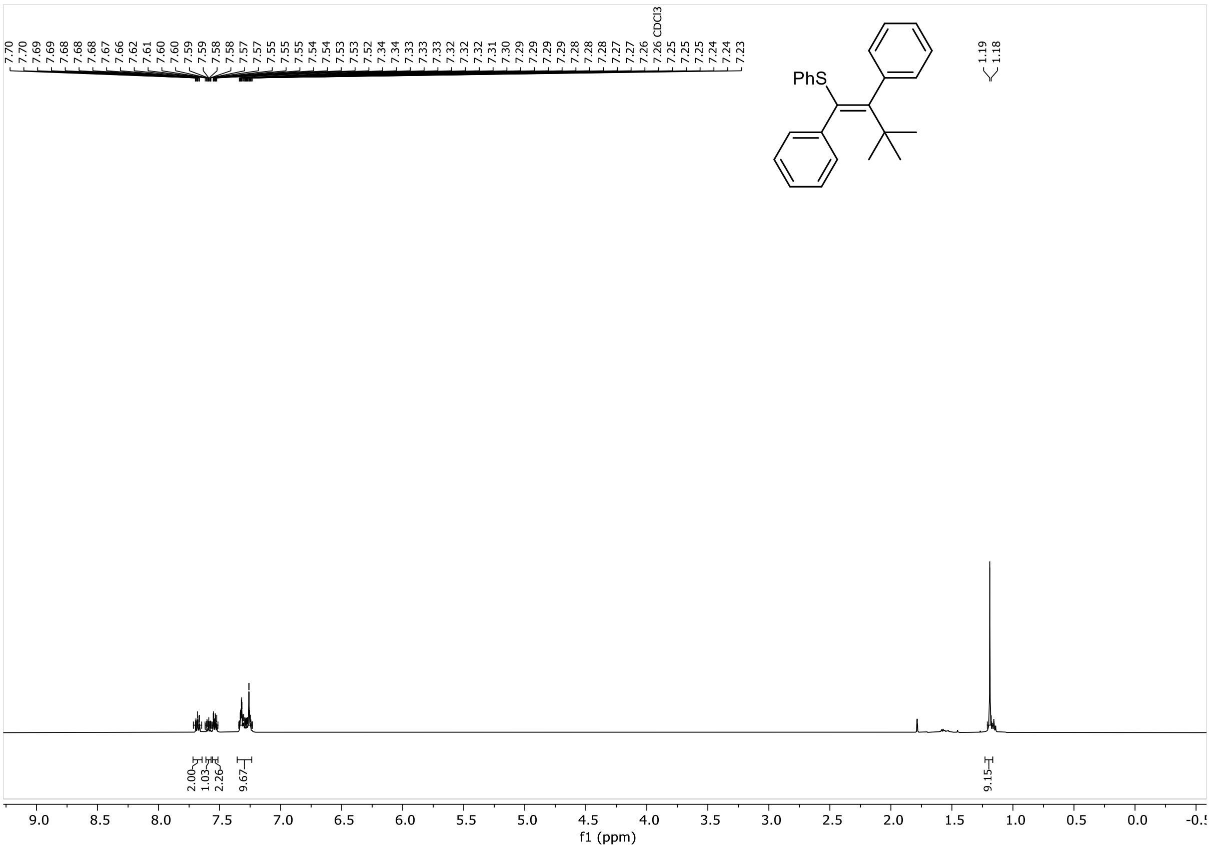

Figure S96. $^{13}C\{^{1}H\}$ NMR spectrum (126 MHz, $CDCl_3$, 298 K) of **3ao**

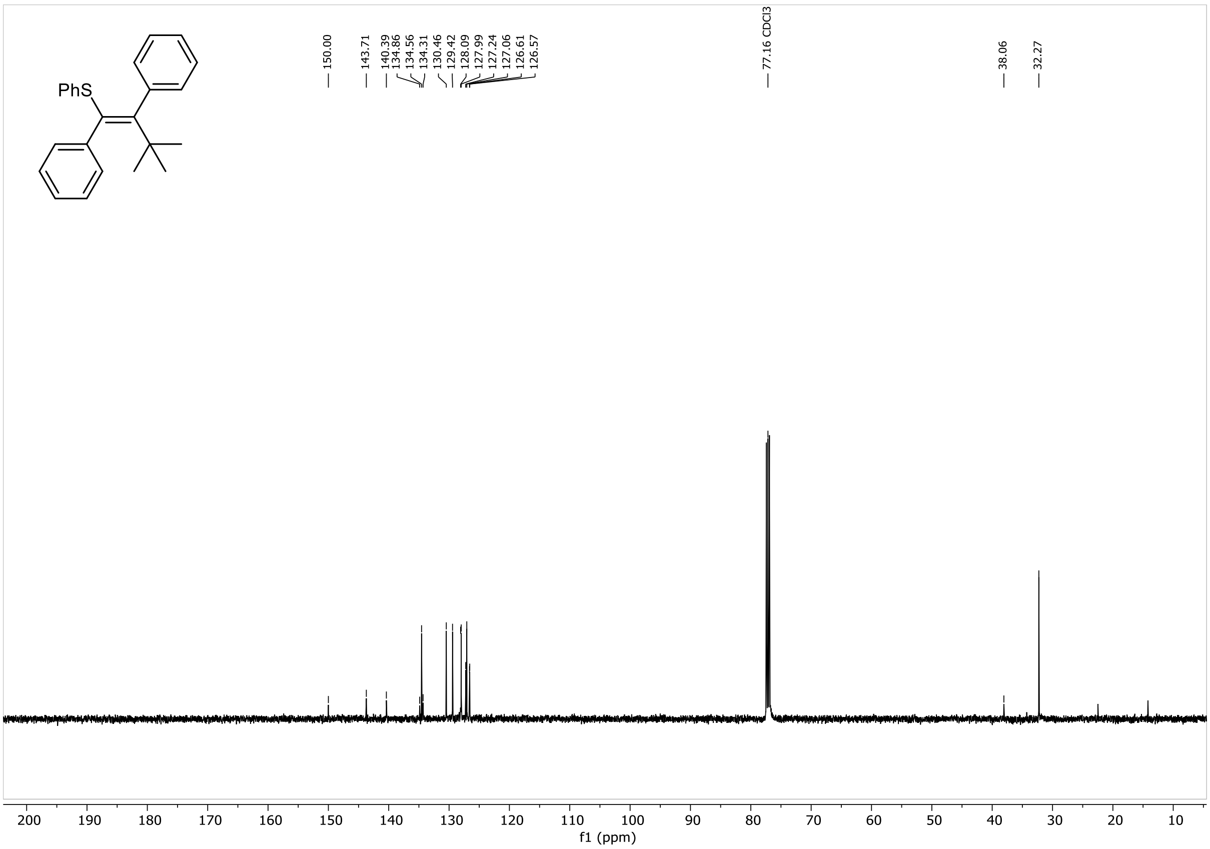

Figure S97. ^{1}H NMR spectrum (500 MHz, $CDCl_3$, 298 K) of *(E)*-(2-Ethyl-3,3-dimethyl-1-(phenylsulfinyl)but-1-en-1-yl)benzene (**12**)

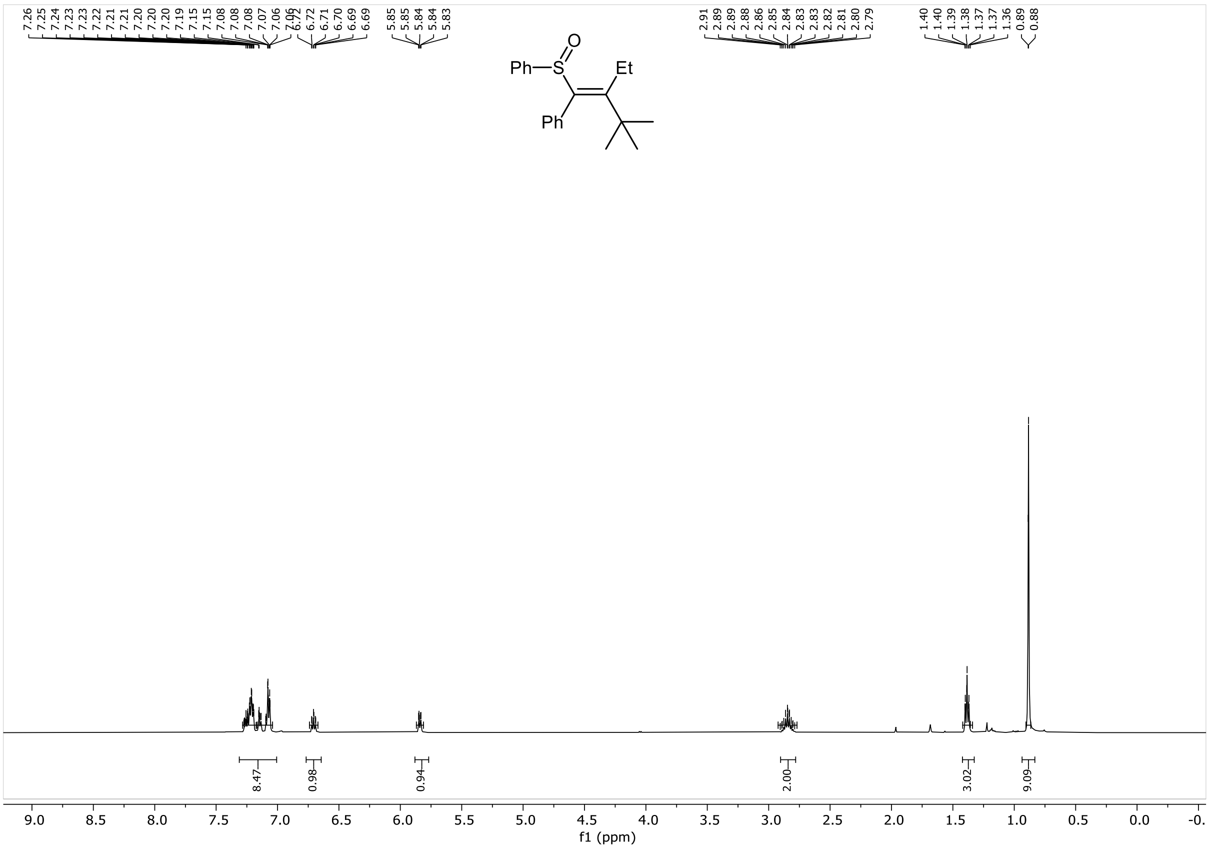

Figure S98. $^{13}C\{^{1}H\}$ NMR spectrum (126 MHz, $CDCl_3$, 298 K) of **12**

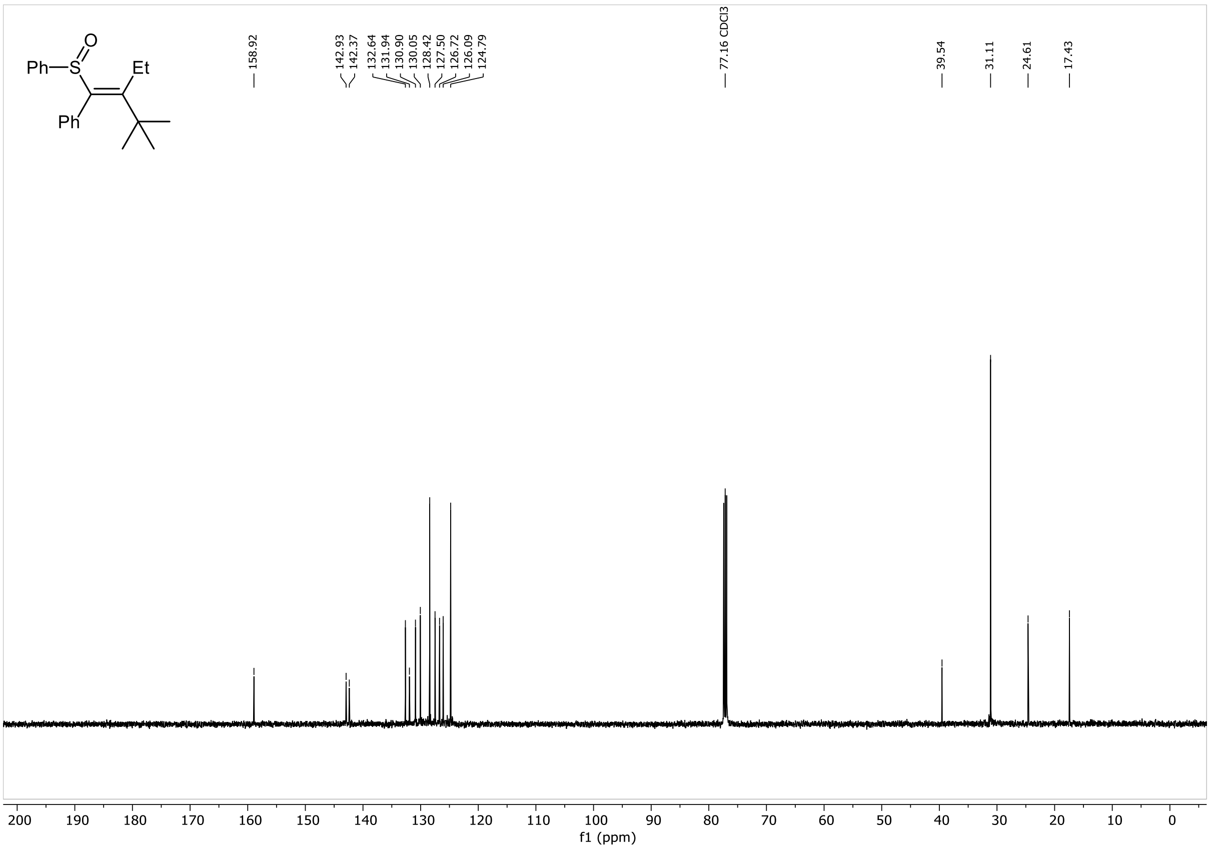

Figure S99. ^{1}H NMR spectrum (500 MHz, $CDCl_3$, 298 K) of *(E)*-(2-ethyl-3,3-dimethyl-1-(phenylsulfonyl)but-1-en-1-yl)benzene (**13**)

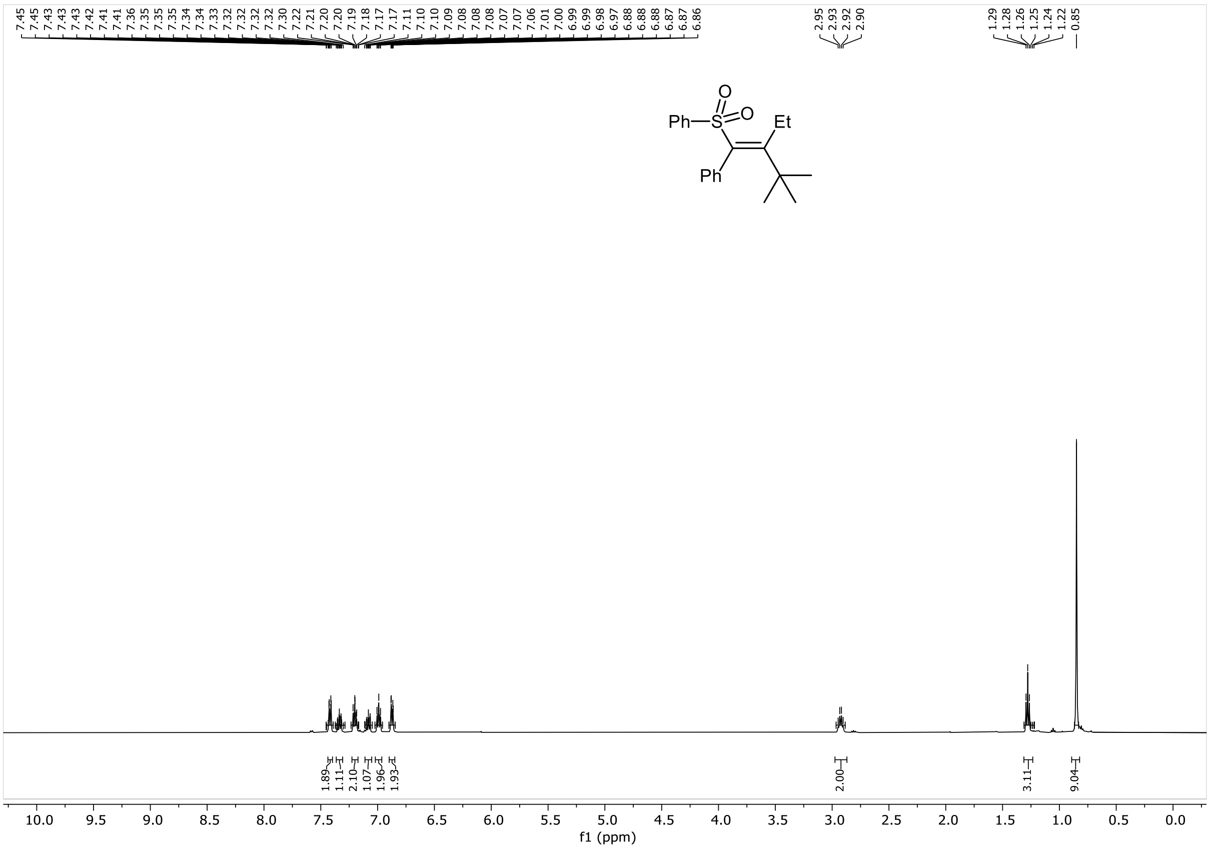

Figure S100. $^{13}C\{^{1}H\}$ NMR spectrum (126 MHz, $CDCl_3$, 298 K) of **13**

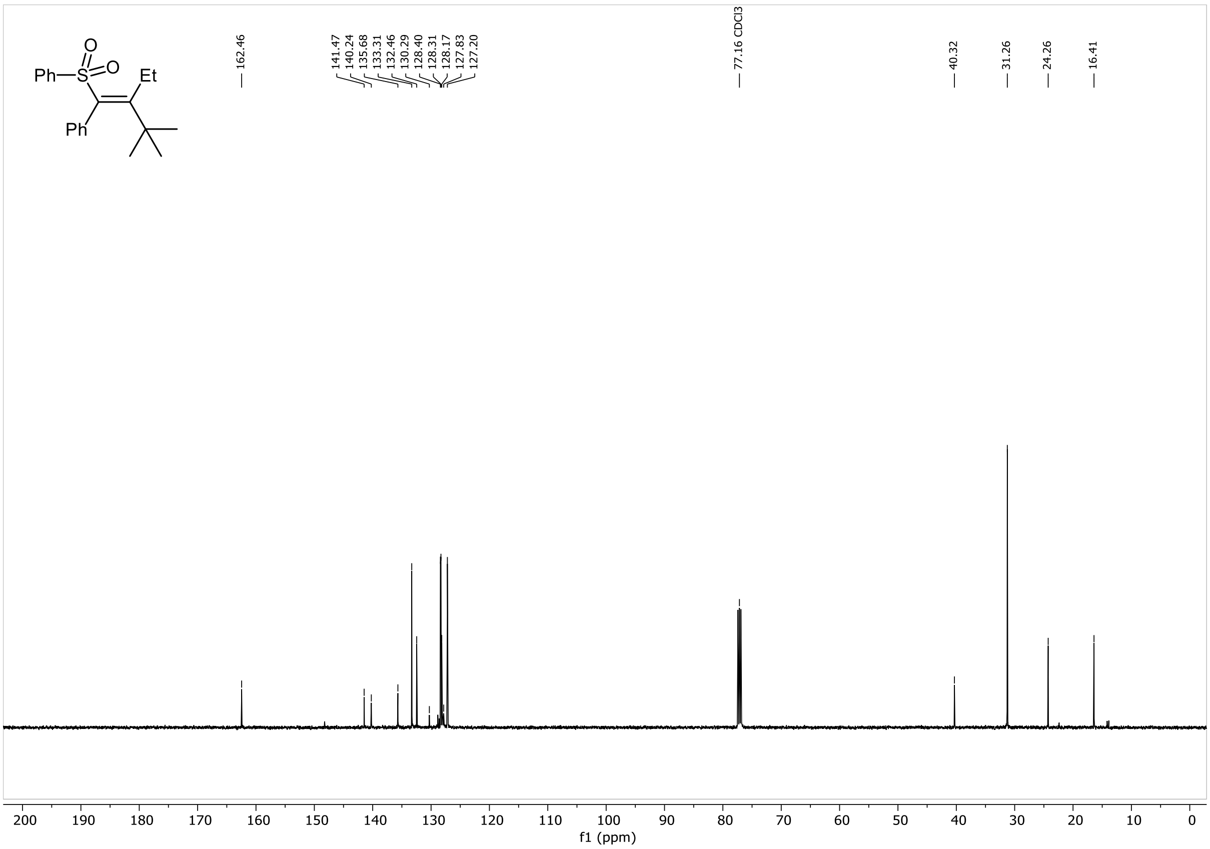

9. References

(S1) Q. Wu, A. Roy, E. Irran, Z. W. Qu, S. Grimme, H. F. T. Klare, and M. Oestreich, *Angew. Chem., Int. Ed.,* 2019, **58**, 17307–17311.

(S2) C. A. Reed, *Acc. Chem. Res.,* 2010, **43**, 121–128.

(S3) R. K. Harris, E. D. Becker, S. M. Cabral De Menezes, R. Goodfellow and P. Granger, *Solid State Nucl. Magn. Reson.,* 2002, **22**, 458–483.

(S4) B. I. P. Smith, N. M. L. Knight, G. J. Knox, D. M. Lindsay, L. C. Paterson, J. Bergare, C. S. Elmore, R. A. Bragg and W. J. Kerr, *Angew. Chem., Int. Ed.,* 2025, **64**, e202417179.

(S5) S. Wu, X. Lei, E. Fan, Z. Sun, *Org. Lett.,* 2018, **20**, 522–525.

(S6) Y. Gong, Z. Zhu, Q. Qian, W. Tong and H. Gong, *Org. Lett.,* 2021, **23**, 1005–1010.

(S7) Y. Chen, H. Qi, N. Chen, D. Ren, J. Xu, and Z. Yang, *J. Org. Chem.,* 2019, **84**, 9044–9050.

(S8) J. -R. Wu, C. -H. Lin and C. F. Lee, *Chem. Commun.,* 2009, **29**, 4450–4452.

(S9) C. Cavedon, A. Madani, P. H. Seeberger and B. Pieber, *Org. Lett.,* 2019, **21**, 5331–5334.

(S10) P. H. Gehrtz, V. Geiger, T. Schmidt, L. Sršan and I. Fleischer, *Org. Lett.,* 2019, **21**, 50–55.

(S11) N. C. Cutress and T. Bruce, *J. Chem. Soc. Perkin Trans. II,* 1974, 263–268.

(S12) M. Sayah and M. G. Organ, *Chem. Eur. J.,* 2011, **17**, 11719–11722.

(S13) D. Babin, J. D. Fourneron, L. M. Harwood, M. Julia, *Tetrahedron,* 1981, **37**, 325–332.

(S14) Z. Wu and D. A. Pratt, *Angew. Chem., Int. Ed.,* 2021, **60**, 15598–15605.

(S15) B. Du, B. Jin and P. Sun, *Org. Lett.,* 2014, **16**, 3032–3035.

(S16) J. Kuzmin, J. Röckl, N. Schwarz, J. Djossou, G. Ahumada, M. Ahlquist and H. Lundberg, *Angew. Chem., Int. Ed.,* 2023, **62**, e202304272.

(S17) A. Singh, S. Gupta and J. M. Khurana, *Org. Prep. Proced. Int.,* 2020, **52**, 110–119.

(S18) L. Rout, P. Saha, S. Jammi and T. Punniyamurthy, *Eur. J. Org. Chem.* 2008, **2008**, 640–643.

(S19) N. Kennedy, P. Liu and T. Cohen, *Angew. Chem., Int. Ed.,* 2016, **55**, 383–386.

(S20) K. Fukumoto, M. Kasa, T. Oya, M. Itazaki and H. Nakazawa, *Organometallics,* 2011, **30**, 3461–3463.

(S21) B. Yu, A. -H. Liu, L. -N. He, B. Li, Z. -F. Diao and Y. -N. Li, *Green Chem.,* 2012, **14**, 957–962.

(S22) H. Zuo, E. Irran, H. F. T. Klare and M. Oestreich, *Angew. Chem., Int. Ed.,* 2024, **63**, e202401599.
